# Genetic Background of Taste Perception, Taste Preferences, and Its Nutritional Implications: A Systematic Review

**DOI:** 10.3389/fgene.2019.01272

**Published:** 2019-12-19

**Authors:** Judit Diószegi, Erand Llanaj, Róza Ádány

**Affiliations:** ^1^MTA-DE Public Health Research Group, Public Health Research Institute, University of Debrecen, Debrecen, Hungary; ^2^Doctoral School of Health Sciences, University of Debrecen, Debrecen, Hungary; ^3^WHO Collaborating Centre on Vulnerability and Health, University of Debrecen, Debrecen, Hungary

**Keywords:** food preference, taste preference, taste threshold, taste sensitivity, genetics, genomics

## Abstract

**Background:** The rise in nutrition-related morbidity and mortality requires public health intervention programs targeting nutritional behavior. In addition to socio-economical, socio-cultural, psychological determinants, taste is one of the main factors that influence food choices. Differences in taste perception and sensitivity may be explained by genetic variations, therefore the knowledge of the extent to which genetic factors influence the development of individual taste preferences and eating patterns is important for public policy actions addressing nutritional behaviors. Our aim was to review genetic polymorphisms accounting for variability in taste and food preferences to contribute to an improved understanding of development of taste and food preferences.

**Methods:** The electronic databases PubMed, Scopus, and Web of Science were searched using MeSH in PubMed and free text terms for articles published between January 1, 2000 and April 13, 2018. The search strategy was conducted following the PRISMA statement. The quality of the included studies was assessed by the validated Q-Genie tool.

**Results:** Following the PRISMA flowchart, finally 103 articles were included in the review. Among the reviewed studies, 43 were rated to have good quality, 47 were rated to have moderate quality, and 13 were rated to have low quality. The majority of the studies assessed the association of genetic variants with the bitter taste modality, followed by articles analyzing the impact of polymorphisms on sweet and fat preferences. The number of studies investigating the association between umami, salty, and sour taste qualities and genetic polymorphisms was limited.

**Conclusions:** Our findings suggest that a significant association exists between TAS2R38 variants (rs713598, rs1726866, rs10246939) and bitter and sweet taste preference. Other confirmed results are related to rs1761667 (CD36) and fat taste responsiveness. Otherwise further research is essential to confirm results of studies related to genetic variants and individual taste sensitivity. This knowledge may enhance our understanding of the development of individual taste and related food preferences and food choices that will aid the development of tailored public health strategy to reduce nutrition-related disease and morbidity.

## Introduction

Globalization related changes resulted in the extremely high prevalence of unhealthy dietary behaviors all over the world especially in low and middle income countries which led to the rise in morbidity (Popkin, 2006; Lachat et al., 2013; Ford et al., 2017) and mortality (Global Burden of Disease Risk Factors Collaborators, 2018) caused by diet-related noncommunicable diseases (NCDs). The unfavorable dietary patterns referred to as the “nutrition transition” can be characterized with an increased consumption of industrially processed foods with high salt, fat, and sugar content and contribute to the development of metabolic abnormalities and consequent NCDs of high public health significance (cardiovascular diseases, type 2 diabetes, and cancer) (Atrup et al., 2008; World Cancer Research Fund, 2018) and *have emerged as the biggest contributor to premature mortality around the world, accounting for 11 million deaths in 2017* (Afshin et al., 2019). Understanding the determinants and drivers of food preferences and food choices is therefore essential to design and implement public health intervention programs targeting nutritional behavior.

Individual food preferences are important predictors of food intake (Drewnowski et al., 1999; Duffy et al., 2009) and are highly influenced by taste perception and taste preference (Glanz et al., 1998; Kearney et al., 2000; Kourouniotis et al., 2016). Taste is listed among the five main values (taste, health, cost, time, and social relationships) in the Food Choice Process Model, which explains the motivations behind food choice decisions (Connors et al., 2001). Sensory perceptions, such as taste sensitivity vary widely among individuals that may partly be explained by genetic polymorphisms located in genes involved in taste perception of the five basic taste qualities and the most recently identified fat taste (Malles, 2010; Running et al., 2015) modality. The magnitude of genetic predisposition to perceived intensity and preference of distinct compounds is provided by family and twin studies, expressed in terms of heritability, i.e., the degree to which genetic differences contribute to individual differences in taste perception and preference. Heritability estimates range from high to moderate for bitter tasting stimuli [0.72, 0.71, 0.34, for 6-n-propylthiouracil (PROP) (Hansen et al., 2006), phenylthiocarbamide (PTC) (Knaapila et al., 2012), and quinine hydrochloride (Hansen et al., 2006), respectively] and moderate for sweet tasting compounds (glucose: h^2^^=^ 0.31, fructose: h^2^^=^ 0.34) (Hwang et al., 2015). Accordingly food preferences that are determined by taste perception are also influenced by genetic factors with similar heritability estimates (dessert foods (0.20), vegetables (0.37–0.54), fruits (0.49–0.53), protein foods (0.48–0.78) (Breen et al., 2006; Fildes et al., 2014; Smith et al., 2016) and the correlation for fat intake (as a percentage of energy intake) was found to be 0.61 for monozygotic twins in a study conducted among subjects of French descent (Pérusse et al., 1988).

The aim of our present study was to review genetic polymorphisms accounting for variability in taste and food preferences to contribute to an improved understanding of the role of genetic polymorphisms in the development of taste and food preferences. Knowledge of the extent to which genetic and environmental factors influence the development of individual taste preferences and eating patterns is important for public policy actions addressing nutritional behavior of populations.

## Methods

### Search Strategy and Eligibility Criteria

Systematic searches were conducted in the electronic databases PubMed, Scopus, and Web of Science for articles published between January 1, 2000 and April 13, 2018 to identify relevant publications. The search strategy was to follow the PRISMA statement. The search terms included controlled terms, e.g., MeSH in PubMed and free text terms. The search was based on a combination of the following keywords: (“taste preference” OR “taste sensitivity” OR “taste threshold” OR “food preference” OR “bitter taste preference” OR “bitter taste sensitivity” OR “bitter taste threshold” OR “sweet taste preference” OR “sweet taste sensitivity” OR “sweet taste threshold” OR “salt taste preference” OR “salt taste sensitivity” OR “salt taste threshold” OR “sour taste preference” OR “sour taste sensitivity” OR “sour taste threshold” OR “umami taste preference” OR “umami taste sensitivity” OR “umami taste threshold” OR “fat taste preference” OR “fat taste sensitivity” OR “fat taste threshold”) AND (“genetics” OR “genomics”). The references of the selected publications were also checked for other potentially eligible studies.

**Figure 1:**
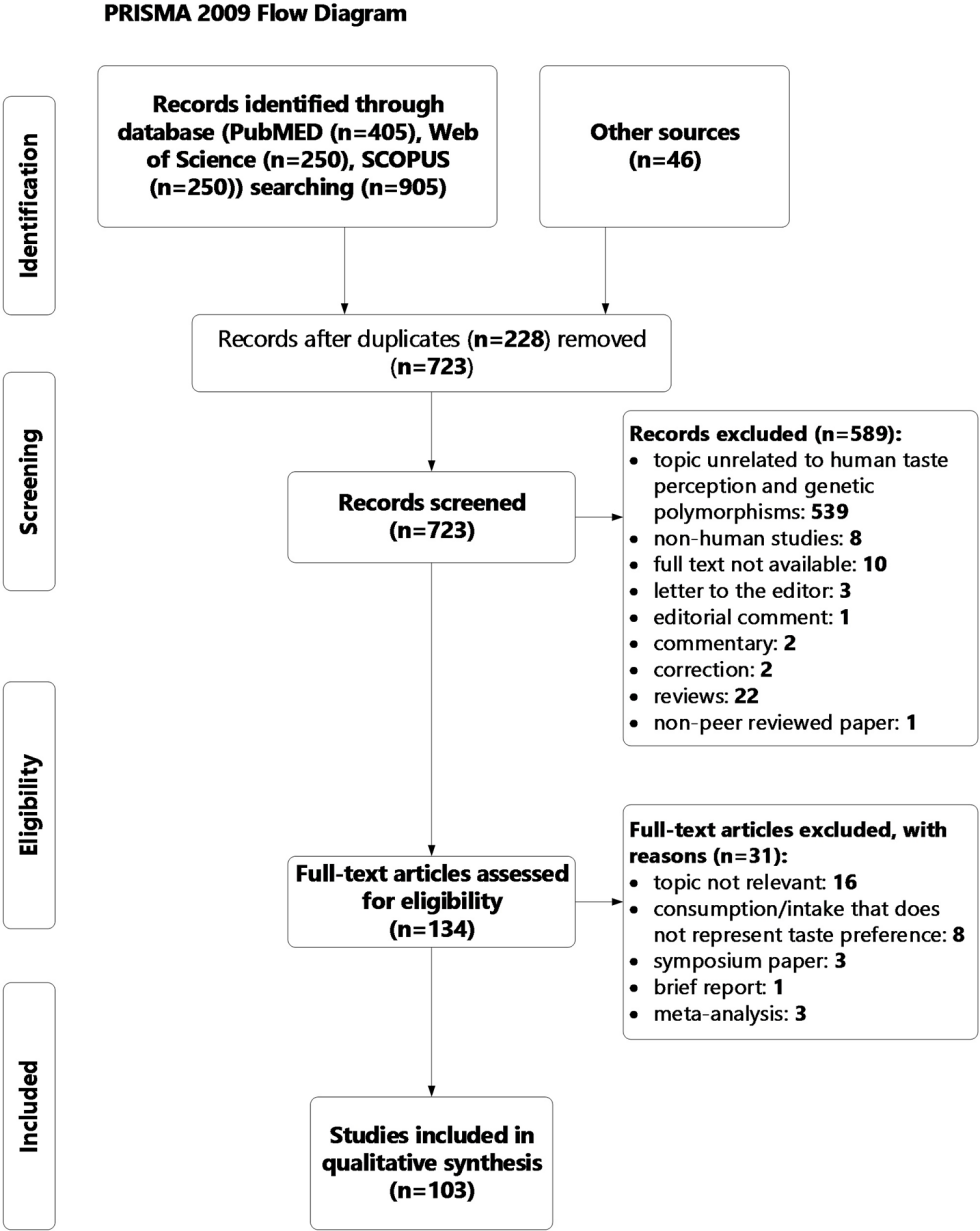
PRISMA flowchart of study selection process.

Studies were excluded: i) that were not written in English; ii) not targeting human subjects; iii); which were not published as peer reviewed in scientific journals; iv) that were not available in full-text format. **Figure 1** summarizes the manuscript selection process.

### Quality Assessment of Primary Studies

The quality of the included studies was assessed independently by two reviewers (JD, EL) using the validated Q-Genie tool. This tool was developed by Sohani et al. (2015) based on the Strengthening the Reporting of Genetic Association Studies (STREGA) and Strengthening the Reporting of Genetic Risk Prediction Studies (GRIPS) and as well as recommendations by Human Molecular Genetics, Diabetologia, Nature Genetics, and individual research groups guidelines. This instrument is composed of 11 items formulated as questions covering the following categories: rationale for study, selection and definition of outcome of interest, selection and comparability of comparison groups, technical classification of the exposure, non-technical classification of the exposure, other source of bias, sample size and power, *a priori* planning of analysis, statistical methods and control for confounding, testing of assumptions and inferences for genetic analysis, and appropriateness of inferences drawn from results. Each item is scored on 7-point Likert Scale ranging from 1 (poor) to 7 (excellent). Each reviewer (JD, EL) generated an overall quality score, after reading and examining each study independently. The quality of studies could be labeled as poor (total scores ≤35 for studies with control groups and scores ≤32 for studies without control groups), moderate (total scores >35 and ≤45 for studies with control groups and scores >32 and ≤40 for studies without control groups), or good (total scores >45 for studies with control groups and scores >40 for studies without control groups) (Sohani et al., 2015). Disagreement between the reviewers on individual items were identified and solved during a consensus meeting. Detailed instructions for using the checklist are provided elsewhere (Sohani et al., 2016).

### Data Extraction

First, duplicates were removed and then all the abstracts of the remaining articles were screened by two independent reviewers (JD, EL). Two authors (JD, EL) extracted data independently from the identified studies.

## Results

### Search Outcomes

In total, 949 publications were identified using PubMed (n = 405), Scopus (n = 250), Web of Science (n = 250), and from reference lists of all relevant articles (n = 46). After the duplicates were removed (n = 228) the abstracts of the remaining articles (n = 723) were screened by two independent reviewers (JD, EL). Any disagreement was resolved by discussion. Studies that did not meet the inclusion criteria (n = 589) were removed, resulting in 134 articles for full-text assessment for eligibility. Out of them 31 were further excluded (**Figure 1** displays the reasons). Finally, 103 articles were included in the review.

### Studies Included in the Review

The majority of the studies assessed analyzed the association of genetic variants with the bitter taste modality (n = 64), followed by articles on the impact of polymorphisms on sweet (n = 28) and fat preferences (n = 22). The number of studies investigating the association between umami, salty, and sour taste perceptions and genetic polymorphisms was limited (n = 6, n = 6, and n = 4, respectively). **Tables 1**–**6** summarize the findings for each modality. Due to the extensive literature and data extracted regarding bitter, sweet, and fat taste qualities, only those genetic associations are presented in **Tables 1**–**3**, where the number of studies with confirmed association was more than one, except only few single publications on sweet perception, where the association could be expected on the basis of the known molecular mechanisms of taste recognition. Genetic variants with only one or no confirmed associations are presented in **Supplementary Tables 1** and **2**. **Tables 4**–**6** include all findings for umami, salty and sour taste modalities, respectively. **Supplementary Tables 3**–**12** include the design, methodology, and study population details of each study included in the review.

**Table 1:** Overview of genetic association studies related to bitter taste preferences.

Gene	SNP	Applied tastant/method	Number of studies with confirmed association	Findings	Reference	Number of studies with no association	Reference
**TAS2R38**	**rs713598**	PTC, PROP, bitterness of wine/alcohol, food habits questionnaire (liking), detection threshold of methimazole, salicin	13 (1)	Homozygotes (P49) had the lowest mean thresholds (i.e., greater sensitivity) to both PTC and PROP. The A49P variant demonstrated a strong association with PTC taster status. The variant alleles were inversely associated with bitterness perception of PROP and wine/alcohol. PP conferred to PROP sensitivity. Associated with broccoli score. PP tasters associated with aversion of bitter vegetables and preference of sweet vegetables. Associated with thioamide and salicin detection threshold	(Kim et al., 2003; Ooi et al., 2010; Wooding et al., 2010; Lucock et al., 2011; Colares-Bento et al., 2012; Allen et al., 2013a; Behrens et al., 2013; Allen et al., 2014; Bering et al., 2014; Keller et al., 2014; Mennella et al., 2014a; Carrai et al., 2017; Risso et al., 2017)	1	(Mennella et al., 2011a) (PROP, mixed population of children, adolescents, and adults)
**TAS2R38**	**rs1726866**	PTC, PROP, bitterness of wine, food habits questionnaire (liking), detection threshold of methimazole, salicin	10 (1)	Homozygotes (V296) had the lowest mean thresholds (i.e., greater sensitivity) to both PTC and PROP. Individuals with “A” rather than a “V,” could perceive the bitterness of PROP increased. The variant alleles were inversely associated with bitterness perception of PROP and wine. Associated with broccoli score. Associated with thioamide and salicin detection threshold	(Kim et al., 2003; Wooding et al., 2010; Lucock et al., 2011; Mennella et al., 2011a; Allen et al., 2013a; Behrens et al., 2013; Bering et al., 2014; Robino et al., 2016; Carrai et al., 2017; Risso et al., 2017)	2	(Duffy et al., 2004a; Timpson et al., 2007) (AceK bitterness, bitterness of alcohol)
**TAS2R38**	**rs10246939**	PROP, bitterness of wine/alcohol, food habits questionnaire (liking), detection threshold of methimazole, salicin	8 (1)	Individuals with a “V” in the last position were more likely to detect bitterness at the lowest concentration compared with subjects with the same diplotype but with an “I” in the last position. The variant alleles were inversely associated with bitterness perception of PROP and wine. Associated with broccoli score. Associated with thioamide and salicin detection threshold	(Lucock et al., 2011; Mennella et al., 2011a; Behrens et al., 2013; Allen et al., 2014; Bering et al., 2014; Ledda et al., 2014; Carrai et al., 2017; Risso et al., 2017)	0	–
**TAS2R38**	**A49P (rs713598), A262V (rs1726866)**	PROP	2	Associated with PROP phenotype	(Timpson et al., 2007; Bering et al., 2014)	0	–
**TAS2R38**	**A49P (rs713598), A262V (rs1726866)**	Cruciferous/*Brassica* vegetable intake (24-hour dietary recall, food record)	1 (1)	Haplotype associated with cruciferous vegetable intake	(Sacerdote et al., 2007)	1 (Popkin, 2006)	(Inoue et al., 2013)
**TAS2R38**	**A49P (rs713598), A262V (rs1726866), V296I (rs10246939)**	PTC, PROP, food habits questionnaire (liking), bitterness of nonglucosinolate-generating vegetables, bitterness of alcohol, liking and bitterness perception of salad rocket, detection threshold of methimazole, salicin	23 (4)	PAV homozygotes possessed a greater sensitivity to PTC compared with AVI. Taster PAV haplotypes inversely correlated with broccoli score and positively associated with PROP perceived bitterness. PAV/PAV subjects rated the glucosinolate-generating vegetables more bitter, than AVI/AVI subjects. Bitterness of ethanol differed significantly among haplotypes. Associated with bitterness perception and scores of salad rocket. Associated with thioamide and salicin detection threshold	(Kim et al., 2003; Duffy et al., 2004a; Mennella et al., 2005; Sandell and Breslin, 2006; Hayes et al., 2008; Duffy et al., 2010; Wooding et al., 2010; Calò C et al., 2011; Lucock et al., 2011; Cabras et al., 2012; Negri et al., 2012; Campbell et al., 2012; Melis et al., 2013; Allen et al., 2014; Bering et al., 2014; Garneau et al., 2014; Robino et al., 2014; Melis et al., 2015; Nolden et al., 2016; Bella et al., 2017; Deshaware and Singhal, 2017; Risso et al., 2017in children also, Behrens et al., 2013)	2	(Feeney et al., 2017)
**TAS2R38**	**A49P (rs713598), A262V (rs1726866), V296I (rs10246939)**	Bitterness of berry juice samples and extracts (bilberry, crowberry)	1 (1)	AVI/AVI subjects rated bitterness, higher than the PAV/PAV subjects	(Laaksonen et al., 2013)	0	–
**TAS2R38**	**A49P (rs713598), A262V (rs1726866), V296I (rs10246939)**	*Brassica* vegetable intake (FFQ)	1 (1)	Associated with consumption of bitter-tasting vegetables (only in children)	(Feeney et al., 2011)	2 (1)	(Gorovic N et al., 2011; Negri et al., 2012)
**TAS2R19**	**rs10772420**	Quinine, detection and recognition thresholds, perceived bitter taste intensities of absinthin, amarogentin, cascarillin, grosheimin, quassin, and quinine, PROP bitterness of unsweetened grapefruit juice	5	Associated with quinine intensity ratings. A allele was associated with more intense quinine perception. Associated with grosheimin detection threshold and intensities (weak, moderate, strong, very strong). Individuals who were homozygous for the Cys299 allele rated grapefruit juice twice as bitter and liked it less as Arg299 homozygotes or heterozygotes.	(Reed et al., 2010; Hayes et al., 2011; Knaapila et al., 2012; Hayes et al., 2015; Roudnitzky et al., 2015)	1	(Bering et al., 2014) (PROP)
**TAS2R19**	**rs1868769**	Quinine, detection and recognition thresholds, perceived bitter taste intensities of absinthin, amarogentin, cascarillin, grosheimin, quassin, and quinine, PROP	2	Associated with quinine intensity ratings. Associated with grosheimin detection threshold, recognition threshold, and weak intensity	(Knaapila et al., 2012; Roudnitzky et al., 2015)	1	(Bering et al., 2014) (PROP)
**TAS2R31 (formerly TAS2R44)**	**rs10845293**	Detection and recognition thresholds, perceived bitter taste intensities of absinthin, amarogentin, cascarillin, grosheimin, quassin, and quinine, saccharin recognition threshold, bitterness of acesulfame potassium	6(1)	Associated with grosheimin detection threshold and intensities (weak, moderate, strong, very strong). Associated with saccharin response. Individuals with at least one TAS2R44-W35 allele were more sensitive to saccharin compared to the group homozygous for the hTAS2R44-R35 allele. Val227 homozygotes reported less bitterness from AceK than the Ala227 homozygotes (heterozygotes intermediate). Association with quinine bitterness	(Pronin et al., 2007; Roudnitzky et al., 2011; Allen et al., 2013a; Allen et al., 2013b; Hayes et al., 2015; Roudnitzky et al., 2015)	1	(Timpson et al., 2007) (PROP)
**TAS2R31 (formerly TAS2R44)**	**rs10772423**	Detection and recognition thresholds, perceived bitter taste intensities of absinthin, amarogentin, cascarillin, grosheimin, quassin, and quinine, bitterness of acesulfame potassium, grapefruit liking	3	Associated with amarogentin intensity (weak), grosheimin detection threshold, recognition threshold, and intensities (weak, moderate, strong, very strong intensity). Val240 homozygotes reported less bitterness from AceK than the Ile240 homozygotes (Val/Ile heterozygotes intermediate). Association with quinine bitterness and grapefruit liking	(Allen et al., 2013b; Hayes et al., 2015; Roudnitzky et al., 2015)	1	(Nolden et al., 2016) (Bitterness of capsaicin, piperine, ethanol)
**TAS2R4**	**rs2234001**	Bitterness of stevioside, bitterness of unsweetened grapefruit juice, instant espresso	2	Bitterness of stevioside positively associated with G allele. Haplotype, allelic variation (TAS2R3, -R4, and -R5) explained variability in coffee bitterness [individuals with one or two copies of the more responsive haplotype (TGAG) experienced twice as much bitterness compared with individuals homozygous for the less responsive haplotype (CCGT), but these haplotypes did not predict coffee liking].	(Hayes et al., 2011; Risso et al., 2017)	4	(Duffy et al., 2004a; Pronin et al., 2007; Timpson et al., 2007; Lucock et al., 2011) (PROP, AceK bitterness)
**TAS2R5**	**rs2227264**	Bitterness of unsweetened grapefruit juice, instant espresso, PROP	2	Haplotype, allelic variation (TAS2R3, -R4, and -R5) explained variability in coffee bitterness [individuals with one or two copies of the more responsive haplotype (TGAG) experienced twice as much bitterness compared with individuals homozygous for the less responsive haplotype (CCGT), the haploblock did not predict coffee liking]. Associated with PROP phenotype	(Hayes et al., 2011; Carrai et al., 2017)	0	–
**TAS2R5**	**rs2234012**	Bitterness of unsweetened grapefruit juice, instant espresso, intake, PROP	2	Haplotype, allelic variation (TAS2R3, -R4, and -R5) explained variability in coffee bitterness [individuals with one or two copies of the more responsive haplotype (TGAG) experienced twice as much bitterness compared with individuals homozygous for the less responsive haplotype (CCGT), the haploblock did not predict coffee liking]. Associated with PROP phenotype	(Hayes et al., 2011; Nolden et al., 2016)	0	–
**TAS2R9**	**rs3741845**	Bitterness of acesulfame potassium, bitterness of capsaicin, piperine, ethanol	2	Ala187 homozygotes reported less bitterness than heterozygotes and the Val187 homozygotes.	(Pronin et al., 2007; Timpson et al., 2007)	2	(Sandell and Breslin, 2006; Timpson et al., 2007) (PROP)
**CA6**	**rs2274333**	PROP	4 (1)	The genotype AA and allele A were more frequent in supertasters, whereas genotype GG and allele G were more frequent in non-tasters. GG *vs.* AA or AG had thresholds that were more than 10-fold higher. Supertasters had a very high frequency of genotype AA and allele A, whereas non-tasters had a higher frequency of genotype GG and allele G. PROP super-tasters had a very high frequency of allele A, whereas non-tasters had a higher frequency of allele G.	(Padiglia et al., 2010; Calò C et al., 2011; Cabras et al., 2012; Melis et al., 2013)	3	(Bering et al., 2014; Feeney and Hayes, 2014; Risso et al., 2017)

Number of low quality studies is presented in parentheses.

PTC, phenylthiocarbamide; PROP, 6-n-propylthiouracil; FFQ, food frequency questionnaire; AceK, acesulfame potassium.

**Table 2:** Overview of genetic association studies related to sweet taste preferences.

Gene	SNP	Applied tastant/method	Number of studies with confirmed association	Findings	Reference	Number of studies with no association	Reference
**TAS1R2**	rs3935570	Sucrose, sugar intake (FFQ)	1	GG or GT *vs.* TT had significantly higher detection thresholds [and lower suprathreshold sensitivity ratings (iAUC)] but only in individuals with BMI ≥ 25. (No effect on sugar consumption.)	(Dias et al., 2015)	0	–
**TAS1R2**	rs12033832	Sucrose, sugar intake (FFQ)	1	Individuals with a BMI ≥ 25: G allele carriers had significantly higher detection and lower suprathreshold sensitivity ratings (iAUC), higher intake of total sugars, sucrose, fructose, and glucose. Individuals with a BMI <25: significantly lower detection thresholds and no effect on suprathreshold taste, lower intake of total sugars, sucrose, fructose, glucose, and lactose	(Dias et al., 2015)	2	(Fushan et al., 2009; Han et al., 2017)
**TAS1R2**	rs35874116	Intake of sweet food (three factor eating questionnaire)	2	CC and CT *vs.* TT associated with higher intake of sweet foods. Overweight Val carriers consumed less sugars, sucrose, fructose, and glucose than Ile homozygotes.	(Eny et al., 2010; Han et al., 2017)	0	–
**TAS1R3**	rs307355	Sucrose	1	Strong association with decreased sucrose AUC scores (reduced taste sensitivity to sucrose associated with T alleles)	(Fushan et al., 2009)	1	(Han et al., 2017)
**TAS1R3**	rs35744813	Sucrose	2	Strong association with decreased sucrose AUC scores (reduced taste sensitivity to sucrose associated with T alleles). Adults with no T alleles preferred a lower concentration of sucrose than did those with one or two T alleles (no association in children).	(Fushan et al., 2009; Mennella et al., 2014b)	2	(Joseph et al., 2016; Han et al., 2017)
**TAS2R38**	rs713598	Intake of sweet tasting foods (3-day, weighed dietary records, test meal)	2	The PP/PA genotype was associated with a higher intake of (energy dense) sweet tasting foods in children. AP or PP children consumed more chocolate chip cookies at the test-meal than children who had the AA genotype.	(Keller et al., 2014; Pawellek et al., 2016)	0	–
**TAS2R38**	rs713598	Sucrose, food preference questionnaire	3	PP children preferred higher concentrations of sucrose in water and beverages containing more sugar than AA children (AP intermediate preference). GG subjects did not prefer sweet foods (dessert and chocolate). P allele more common in children with lower sucrose thresholds	(Lipchock et al., 2012; Joseph et al., 2016; Perna et al., 2018)	1	(Ooi et al., 2010)
**TAS2R38**	rs1726866	Sucrose	1	Children with one or two bitter-sensitive A alleles had lower detection thresholds (more sensitive to the taste of sucrose).	(Joseph et al., 2016)	1	(Timpson et al., 2007) (AceK sweetness)
**TAS2R38**	rs10246939	Sucrose	1	Children with one or two bitter-sensitive V alleles had lower detection thresholds (more sensitive to the taste of sucrose).	(Joseph et al., 2016)	0	–
**TAS2R38**	A49P (rs713598), A262V (rs1726866), V296I (rs10246939)	Berry products liking	1	Majority of PAV/PAV and PAV/AVI children, liked the sweetened, dried bilberries with rather high sugar content.	(Suomela et al., 2012)	0	–
**TAS2R38**	A49P (rs713598), A262V (rs1726866), V296I (rs10246939)	Sweet food intake (FFQ)	1	PAV homozygotic individuals consumed more sweet foods than did the AVI homozygotic subjects.	(Sandell et al., 2014)	0	–

FFQ, food frequency questionnaire; BMI, body mass index; AUC, area under the curve; AceK, acesulfame potassium; iAUC, incremental area under the curve.

Individual ratings of the intensity of suprathreshold solutions were plotted, and the incremental area under the taste sensitivity curve (iAUC) was computed.

**Table 3:** Overview of genetic association studies related to fat taste preferences.

Gene	SNP	Applied tastant/method	Number of studies with confirmed association	Findings	Reference	Number of studies with no association	Reference
**CD36**	rs1761667	Oleic acid threshold	3 (1)	GG *vs.* AA linked to lower threshold for oleic acid. Threshold higher in A-allele (obese) children than in G-allele children.	(Pepino et al., 2012; Melis et al., 2015; Mrizak et al., 2015; Sayed et al., 2015)	1	(Daoudi et al., 2015)
**CD36**	rs1761667	Perceived oiliness, fat content, and creaminess	2	AA *vs.* GA or GG perceived more creaminess (regardless of fat concentration), associated with acceptance of added fats and oils but no differences in perceived oiliness were reported. AA lowest perceived ratings of fat content.	(Keller et al., 2012; Ong et al., 2017)	0	–
**CD36**	rs1527483	Perceived oiliness, fat content, and creaminess	2	C/T or T/T perceived greater creaminess, oiliness, and fat content.	(Keller et al., 2012; Ong et al., 2017)	1	(Melis et al., 2015)
**IZUMO1**	rs838145	Fat intake (FFQ)	2	Tendency toward decreased total fat intake (A allele carriers), (MUFAs, PUFAs, omega-3 fatty acids). A variant associated with lower fat consumption.	(Tanaka et al., 2013; Søberg et al., 2017)	0	–

Number of low quality studies is presented in parentheses.

MUFA, monounsaturated fatty acid; PUFA, polyunsaturated fatty acid.

**Table 4:** Overview of genetic association studies related to umami taste preferences.

Gene	SNP	Applied tastant/method	Number of studies with confirmed association	Findings	Reference	Number of studies with no association	Reference
**TAS1R3**	**rs307377**	Umami	3	Significant associations between allele frequency and recognition threshold for IMP. The mutation was less frequent in tasters than expected. CT subjects rated MPG/L twice as did those with CC genotype.	(Shigemura et al., 2009; Raliou et al., 2009; Chen et al., 2009)	0	–
**TAS1R3**	**rs76755863**	Umami	2	The mutation G13A was associated with non-tasters and hypotasters. For the rare allele doubling of umami taste intensity ratings.	(Raliou et al., 2009; Chen et al., 2009)	0	–
**TAS1R3**	rs111615792	Umami	1	For the rare allele doubling of umami taste intensity ratings.	(Chen et al., 2009)	0	–
**TAS1R1**	rs34160967	Umami	1	Significant associations between genotypes and recognition thresholds for MSG and M+I. The SNP was more frequent in tasters than expected.	(Shigemura et al., 2009; Raliou et al., 2009)	2	(Chen et al., 2009; Risso et al., 2017)
**TAS1R1**	rs41278020	Umami	1	The mutation was more frequent in non-tasters than expected.	(Raliou et al., 2009)	1	(Chen et al., 2009)
**TAS1R1**	rs35118458	Umami	1	The mutation tended to be more frequent in non-tasters.	(Raliou et al., 2009)	0	–
**GRM1**	rs2814863	Umami	1	The mutation tended to be associated with the non-taster phenotype.	(Raliou et al., 2009)	0	–

For SNPs with more than one confirmed association are presented in bold.

IMP, inosine monophosphate; MPG, monopotassium glutamate; MSG, monosodium glutamate; M+I: MSG in the presence of IMP.

**Table 5:** Overview of genetic association studies related to salty taste preferences.

Gene	SNP	Applied tastant/method	Number of studies with confirmed association	Findings	Reference	Number of studies with no association	Reference
**TRPV1**	rs8065080	NaCl	1	CC genotype significantly lower iAUCs	(Dias et al., 2013)	0	–
**SCNN1B**	rs239345	NaCl	1	AA genotype significantly lower iAUCs	(Dias et al., 2013)	0	–
**SCNN1B**	rs3785368	NaCl	1	TT genotype significantly lower iAUCs	(Dias et al., 2013)	0	–
**CA6**	rs3737665	NaCl, KCl	1	The SNP associated with differences in the perceived intensity of NaCl and KCl saltiness	(Feeney and Hayes, 2014)	0	–
**CA6**	rs3765964	NaCl	1	The SNP associated with differences in the perceived intensity of NaCl	(Feeney and Hayes, 2014)	0	–
**CA6**	rs2274333	KCl	1	The SNP associated with differences in KCl saltiness	(Feeney and Hayes, 2014)	0	–
**TAS1R1**	rs17492553	NaCl	1	T (allele, genotype) lower intensities	(Rawal et al., 2013)	0	–
**TAS1R1**	rs34160967	NaCl	1	A (allele, genotype) lower intensities	(Rawal et al., 2013)	0	–
**TAS2R38**	A49P (rs713598), A262V (rs1726866), V296I (rs10246939)	NaCl	1	PAV/PAV higher ratings for saltiness intensity	(Deshaware and Singhal, 2017)	1	(Hayes et al., 2008)

NaCl, sodium chloride; KCl, potassium chloride; iAUC, incremental area under the curve.

Individual ratings of the intensity of suprathreshold solutions were plotted, and the incremental area under the taste sensitivity curve (iAUC) was computed.

**Table 6:** Overview of genetic association studies related to sour taste preferences.

Gene	SNP	Applied tastant/method	Number of studies with confirmed association	Findings	Reference	Number of studies with no association	Reference
**TAS1R1**	rs17492553	Citric acid	1	T (allele, genotype) associated with lower intensities	(Rawal et al., 2013)	0	–
**TAS1R1**	rs34160967	Citric acid	1	A (allele, genotype) associated with lower intensities	(Rawal et al., 2013)	0	–
**TAS2R38**	A49P (rs713598), A262V (rs1726866), V296I (rs10246939)	Citric acid	0	–	–	1	(Hayes et al., 2008)
**TAS2R38**	A49P (rs713598), A262V (rs1726866), V296I (rs10246939)	Sourness of berry juice samples and extracts (bilberry, crowberry)	1 (1)	AVI/AVI rated sourness higher than the PAV/PAV subjects.	(Laaksonen et al., 2013)	0	–
**NA**	rs6466849	Wine sourness	1	Variant allele associated with wine sourness	(Carrai et al., 2017)	0	–

Number of low quality studies is presented in parentheses.

### Quality Assessment of Primary Studies

Among the reviewed studies, 43 (41.75%) were rated to have good quality, 47 (45.63%) were rated to have moderate quality, and 13 (12.62%) were rated to have low quality.

### Bitter Taste Preference

Ever since the discovery of PTC (bitter) taster status in 1931 (Fox, 1932) a variety of studies investigated this taste quality. More recent studies analyzed the related bitter tasting thiourea compound PROP rather than PTC, and other phenotyping approaches include the preference for bitter tasting foods and beverages. Investigations confirmed that three single nucleotide polymorphisms (SNPs) in the coding region of the TAS2R38 gene leading to amino-acid changes account for variation in human bitter taste perception (2728). Although this gene is the most widely studied there are 25 different taste receptor type 2 (T2Rs) genes (Chandrashekar et al., 2000; Guo and Reed, 2001) (Conte et al., 2002) involved in bitter taste perception. Moreover the bitter taste phenotype is a complex trait influenced by other genetic variants as well, such as the salivary carbonic anhydrase VI (CA6) or gustin protein, which has an effect on fungiform papillae density and maintenance (Melis et al., 2013).

As expected, the majority of studies focused on candidate genes and relevant variants, with TAS2R38 the most extensively studied (n = 40) (Kim et al., 2003; Duffy et al., 2004a; Mennella et al., 2005; Sandell and Breslin, 2006; Sacerdote et al., 2007; Timpson et al., 2007; Hayes et al., 2008; Duffy et al., 2010; Ooi et al., 2010; Wooding et al., 2010; Calò C et al., 2011; Feeney et al., 2011; Gorovic N et al., 2011; Lucock et al., 2011; Mennella et al., 2011a; Cabras et al., 2012; Campbell et al., 2012; Colares-Bento et al., 2012; Negri et al., 2012; Allen et al., 2013a; Behrens et al., 2013; Inoue et al., 2013; Laaksonen et al., 2013; Melis et al., 2013; Allen et al., 2014; Bering et al., 2014; Feeney et al., 2014; Garneau et al., 2014; Keller et al., 2014; Ledda et al., 2014; Mennella et al., 2014a; Robino et al., 2014; Melis et al., 2015; Nolden et al., 2016; Bella et al., 2017; Carrai et al., 2017; Deshaware and Singhal, 2017; Feeney et al., 2017; Risso et al., 2017), followed by TAS2R31 (n = 7) (Pronin et al., 2007; Roudnitzky et al., 2011; Allen et al., 2013a; Allen et al., 2013b; Hayes et al., 2015; Roudnitzky et al., 2015; Nolden et al., 2016), TAS2R19 (n = 6) (1835, Reed et al., 2010; Hayes et al., 2011; Roudnitzky et al., 2015), TAS2R4 (n = 6) (Roudnitzky et al., 2011; Allen et al., 2013a; Allen et al., 2014; Bering et al., 2014; Risso et al., 2017), TAS2R5 (n = 3) (Hayes et al., 2011; Nolden et al., 2016; Carrai et al., 2017), and TAS2R9 (n = 2) (Allen et al., 2013a; Allen et al., 2013b) (**Table 1**). The association of rs227433 (CA6) with PROP phenotype was inconclusive (Padiglia et al., 2010; Calò C et al., 2011; Cabras et al., 2012; Melis et al., 2013; Bering et al., 2014; Feeney and Hayes, 2014; Risso et al., 2017) (presented in **Table 1**). The effect of other TAS2R gene polymorphisms were demonstrated by single studies only (presented in **Supplementary Table 1**). The assessment of perceived bitterness of PROP, PTC, quinine, caffeine/coffee, unsweetened grapefruit juice, berry juice samples and extracts, salad rocket, stevioside, thioamide, aloin, salicin, saccharin, methimazole, acesulfame potassium, denatonium benzoate, absinthin, amarogentin, cascarillin, grosheimin, quassin, capsaicin, piperine, gentiobiose, aspartame, rebaudioside A and D, alcohol/wine, and preference for bitter tasting foods and beverages (broccoli, artichoke, chicory, glucosinolate-generating vegetables, coffee, dark chocolate) were applied as phenotyping methods. Publications on consumption of bitter foods and drinks (*Brassica*/cruciferous vegetables, coffee; measured by the food frequency questionnaire, 24-h dietary recall, and the 3-day food record) only appeared among the search results and were considered for further evaluation as preference, if it was supported by background information, that the consumption was based on free choice and not influenced by other factors.

TAS2R38 rs713598, rs1726866, rs10246939 polymorphisms were associated with PTC and PROP phenotypes and with differences in perceived bitterness of bitter tasting vegetables and berries, wine/alcohol, thioamide, and salicin studies (Kim et al., 2003; Ooi et al., 2010; Wooding et al., 2010; Lucock et al., 2011; Mennella et al., 2011a; Colares-Bento et al., 2012; Behrens et al., 2013; Melis et al., 2013; Bering et al., 2014; Keller et al., 2014; Ledda et al., 2014; Mennella et al., 2014a; Robino et al., 2016; Carrai et al., 2017; Risso et al., 2017). In one study, conducted among 7–13 year old Irish children, TAS2R38 genotype had no significant impact on bitter vegetable liking, although the authors suggest that taster children might be more prone to food neophobia, and might associate green vegetables with bitter or unpleasant tastes (Feeney et al., 2014). The influence of these variants on cruciferous/*Brassica* vegetable intake was showed only by two poor quality studies (Sacerdote et al., 2007; Feeney et al., 2011) and three studies (out of which two were rated as low quality studies) found no association with this phenotype (Negri et al., 2012; Inoue et al., 2013).

Genetic variations located in the TAS2R19 gene were associated with perceived bitterness of phytochemicals quinine and grosheimin (Reed et al., 2010; Knaapila et al., 2012; Hayes et al., 2015; Roudnitzky et al., 2015;) and unsweetened grapefruit juice (Reed et al., 2010; Hayes et al., 2015). TAS2R31 variants correlated with grosheimin, amarogentin, saccharin, acesulfame potassium response, quinine bitterness, and grapefruit liking (Roudnitzky et al., 2011; Allen et al., 2013a; Allen et al., 2013b; Hayes et al., 2015; Roudnitzky et al., 2015). However, it is important to highlight, that the phenotypic associations of rs10772420 (TAS2R19) may be due to strong linkage disequilibrium (LD) between TAS2R19 and TAS2R31 SNPs (Hayes et al., 2015).

### Sweet Taste Preference

Confirmed associations were found in case of 41 polymorphisms in 18 genes (presented in **Table 2** and **Supplementary Table 1**). Variants of the TAS2R38 genes were analyzed as a haploblock and individually as well. Sugar intake/consumption (measured by the food frequency or the three-factor eating questionnaire and the 3-day weighted dietary record) was only considered as preference (regardless of the authors’ statement), if consumption was truly associated with food choice. Other phenotyping methods included the measurement of sucrose detection thresholds and intensities and preference for sweet tasting foods. Sweet taste perception is mediated by heterodimers of two G protein-coupled receptors, taste receptor type 1 member 2 (T1R2), and taste receptor type 1 member 3 (T1R3) (Nelson et al., 2001; Li et al., 2002; Zhao GQ et al., 2003).

Recent studies suggest the involvement of the gustducin signaling molecule in bitter, sweet, and umami taste transduction (Glendinning et al., 2005) as well, still the most convincing findings of this review are linked to TAS2R38 gene polymorphisms (rs713598, rs1726866, rs10246939) (Lipchock et al., 2012; Suomela et al., 2012; Keller et al., 2014; Sandell et al., 2014; Joseph et al., 2016; Pawellek et al., 2016; Perna et al., 2018) involved in shaping bitter taster status. Results of candidate gene studies targeting TAS1R2 rs12033832 (Dias et al., 2015) and TAS1R3 polymorphisms were inconclusive (Fushan et al., 2009; Mennella et al., 2014b). TAS1R2 rs3935570 (Dias et al., 2015) and rs35874116 (Eny et al., 2010; Han et al., 2017) were associated with sweet taste preference but with limited number of studies (n = 1 and n = 2, respectively). Several GNAT3 polymorphisms also showed significant associations (Fushan et al., 2009) (**Supplementary Table 2**). The list of other polymorphisms with possible explanatory mechanism, which were confirmed by single studies, consists of genes involved in glucose metabolism, umami perception, metabolic processes, signal transduction and neurotransmission, regulation of energy homeostasis (SLC2A2, TAS1R1, ADIPOQ, ANKK1, DRD2, OPRM1, LEP, LEPR, NPY1, respectively) (Mizuta et al., 2008; Elbers et al., 2009; Eny et al., 2010; Davis et al., 2011; Jabłoński et al., 2013; Rawal et al., 2013; Wakai et al., 2013). Further studies are essential to confirm the results of candidate gene studies and discover the pathomechanistic link between other SNPs and sweet taste perception.

### Fat Taste Preference

The most recently identified primary sensory quality of the gustatory system is oleogustus (Malles, 2010; Running et al., 2015). A total of 24 SNPs in 15 genes were associated with fat taste preference (presented in **Table 3** and **Supplementary Table 1**). The three TAS2R38 SNPs were investigated as a haplotype. If fat intake/consumption (measured by food frequency or diet history questionnaire) was interpreted as preference by authors, only those results were considered as preference where consumption was truly associated with voluntary diet selection.

Other phenotyping methods included the measurement of oleic acid sensory threshold, perception of creaminess, oiliness, fat content, and preference for fatty foods. The most convincing results are related to polymorphisms (rs1761667, rs15227483) in the CD36 gene (Keller et al., 2012; Pepino et al., 2012; Melis et al., 2015; Mrizak et al., 2015; Sayed et al., 2015; Ong et al., 2017). This gene encodes the fatty acid translocase that has strong affinity for dietary long-chain fatty acids (LCFA) (Baillie et al., 1996) and serves as a fat taste receptor (Laugerette et al., 2005). The effect of the rs838145 variant located in the IZUMO sperm-egg fusion 1 gene (IZUMO1) on fat preference was also confirmed by two independent studies (Tanaka et al., 2013; Søberg et al., 2017). Some of the other polymorphisms with only one confirmed association and a possible molecular link to fat sensitivity include genes involved in the regulation of lipolysis and thermogenesis, lipoprotein metabolism, neurotransmission, and signaling regulators (ADRB3, APOA2, OPRM1, RGS6, respectively) (Corella et al., 2007; Davis et al., 2011; Sibbel et al., 2011; Sasaki et al., 2013). Future research is needed to explore the effect of other genetic variants and fat taste perception.

### Umami Taste Preference

Umami taste is mediated by a heterodimer complex of G-protein-coupled receptors, taste receptor type 1 member 1 (T1R1) and taste receptor type 1 member 3 (T1R3) (Nelson et al., 2001; Li et al., 2002; Zhao GQ et al., 2003) interacting with amino acids, such as monosodium glutamate (GMP) and inosine monophosphate (IMP). The effect of metabotropic glutamate receptors mGluR1 and mGluR2 has also been implicated in umami taste perception (Chaudhari and Roper, 2010; Raliou et al., 2011; Yasumatsu et al., 2012). Another candidate gene accounting for differences in umami sensitivity is GNAT3 gene that is co-expressed with TAS1R1 (Ishimaru et al., 2012) and encodes G protein alpha subunit gustducin, a taste signaling molecule involved in G-protein-coupled membrane receptors mediated taste transduction (bitter, sweet, and umami) (Glendinning et al., 2005). The number of studies investigating the association between umami taste preference and genetic variants was limited (n = 4) (presented in **Table 4**) and analyzed four candidate genes. Findings suggest that TAS1R1 and TAS1R3 polymorphisms influence taster status (Raliou et al., 2009; Chen et al., 2009; Shigemura et al., 2009), but further investigation is needed to confirm these results and elucidate the role of SNPs located in candidate genes on individual sensitivity to umami.

### Salty Taste Preference

The source of salty taste found in foods is NaCl. The molecular mechanism of salty taste responsiveness is not clear, but the involvement of epithelial sodium channels (ENaCs) located in taste cell membranes in fungiform papillae and amiloride-sensitive vanilloid receptors (Trpv1) have been hypothesized in salt perception (Heck et al., 1984; Lin et al., 1999; Lyall et al., 2004). In humans there are four ENaC subunits (αβγδ) coded by SCNN1A, SCNN1B, SCNN1G, and SCNN1D genes, respectively. The number of studies investigating the association between salty taste preference and genetic polymorphisms was very low (n = 5) (presented in **Table 5**). Only one study analyzed the association between polymorphisms in putative salt receptors and differences in salt taste perception. Homozygotes of TRPV1 rs8065080 (CC genotype), SCNN1B rs239345 (AA genotype), and rs3737665 (TT genotype) perceived salt solutions as significantly weaker than heterozygotes or other allele homozygotes (Dias et al., 2013). Other findings were related to genes linked to bitter (TAS2R38, CA6) (Hayes et al., 2008; Feeney and Hayes, 2014; Deshaware and Singhal, 2017) and umami taste responsiveness (TAS1R1) (Rawal et al., 2013). Future studies are essential to confirm these findings and analyze the role of receptors involved in other taste perception pathways rather than salt.

### Sour Taste Preference

Sour taste perception is triggered by acidic foods and substances. The exact mechanism behind the sensitivity to this taste quality is not yet fully understood, but recent data suggest the involvement of transient receptor potential channels (TRPs), namely polycystic-kidney disease like (PKDL-like) receptors in the mediation of sour taste (Huang et al., 2006; Huque et al., 2009; Ishimaru Y et al., 2016). The number of studies investigating the association between sour taste quality and genetic polymorphisms was limited (n = 4). Phenotype was defined by using citric acid or the sourness perception of berry products and wine. The findings of reviewed studies were not related to candidate genes (PKD2L1, PKD2L3), rather to genes encoding two receptors involved in bitter and umami perception (Laaksonen et al., 2013; Carrai et al., 2017) (**Table 6**). Exploring the effect of these variants on sour taste perception and subsequent sensitivity and implementing studies targeting candidate genes is a future direction.

## Discussion

To our knowledge—after a few narrative reviews (Kim et al., 2004; Garcia-Bailo et al., 2008; Grimm and Steinle, 2011; Dotson et al., 2012; Hayes et al., 2013; Keller and Adise, 2016; Chamoun et al., 2018) that provided a comprehensive, critical, and objective analyses of the scientific knowledge regarding the genetic implications of food preference at the time of their publications—this is the first systematic review prepared by following the PRISMA guideline and using all the most relevant research databases to explore associations between genetic polymorphisms and taste preferences. Food preferences are shaped during fetal development and eating behavior evolves over time. It is a complex trait, determined by interactions of genetic and environmental factors (Birch, 1999; Ventura and Worobey, 2013). The environmental determinants include *in utero* exposures, early postnatal experiences, parental feeding practices, family (social, economic factors), and the wider contexts of the environment (Gibson and Cooke, 2017). Sensory properties of consumed food is an important determinant of dietary habits and taste has been considered as one of the main drivers of food choices and dietary patterns (Connors et al., 2001; Honkanen and Frewer, 2009; Kourouniotis et al., 2016). Chemical compounds in food activate specialized taste receptors that can be altered by genetic polymorphisms and consequently lead to individual taste variability and preferences. Bitter, sweet, and umami perception is linked to G-protein-coupled receptors (Nelson et al., 2001; Li et al., 2002), whereas salt and sour tastes are mediated by ion channels (Heck et al., 1984; Lin et al., 1999; Lyall et al., 2004; Huang et al., 2006; Huque et al., 2009; Ishimaru Y et al., 2016). The most recently identified fat taste modality is believed to be linked to the fatty acid transporter CD36 (Laugerette et al., 2005). There is growing interest in characterizing taste preference based on genetic variation, as well as the association between taste preference and the prevalence of different risk conditions and major diet-related NCDs. Increased understanding of interplay between taste genetics, nutrition, and diet can contribute to the development of public health strategies to improve population health through the prevention of diet-related NCDs.

The genetic components shaping human taste abilities could be a result of natural selection driven by evolutionary adaption mechanisms to avoid the consumption of plant-based toxic substances (Kalmus, 1950; Hladik and Simmen, 1996; Wooding S et al., 2004; Glendinning et al., 2005; Soranzo et al., 2005). These plant-derived toxins generally have an unpleasant bitter taste (Maga, 1990) and excessively bitter-tasting plants will be rejected by humans (Rouseff, 1990). Bitter-tasting compounds include amino acids and peptides, sulfimides (saccharin), ureas and thioureas (PROP and PTC), esters and lactones, terpenoids, and phenols and polyphenols (McBurney, 1990). The oral sensitivity to thiourea moiety (N-C = S) containing chemicals and related structures in food varies widely among individuals.

The bitter-tasting thiourea moiety is present as glucosinolates in *Brassica* vegetables (Hanschen et al., 2014), but other foods and beverages without the thiourea moiety (grapefruit juice, coffee, alcohol, green tea, and soy products) are also perceived as bitter for sensitive individuals (Gayathri Devi et al., 1997; Lanier et al., 2005; Dinehart et al., 2006; Sandell and Breslin, 2006). The supertaster-taster-non-taster categories (Harris H, 1949; Bartoshuk et al., 1994) are linked to combinations of three functional SNPs (rs713598, rs1726866, rs10246939) of the TAS2R38 gene. The homozygous PAV (proline–alanine–valine) haplotype defines the taster form, while the homozygous AVI (alanine-valine-isoleucine) haplotype specifies the non-taster phenotype and heterozygotes possess intermediate sensitivity to PROP and PTC (Kim et al., 2003; Kim and Drayna, 2005), accounting for 85% of the phenotypic variance in PTC perception (Wooding S et al., 2004; Bufe et al., 2005). According to the *in vitro* assays of Buffet *et al*. (2005) rs713598 has the greatest effect on bitter taste signal transduction, rs1726866 possesses weaker effects, and rs10246939 site has no detectable effect at all (Bufe et al., 2005). The bitter taste modality has been the most extensively studied and the majority of genetic association studies related to the bitter quality focused on TAS2R38 gene polymorphisms (Kim et al., 2003; Mennella et al., 2005; Sandell and Breslin, 2006; Sacerdote et al., 2007; Timpson et al., 2007; Hayes et al., 2008; Duffy et al., 2010; Ooi et al., 2010; Wooding et al., 2010; Calò C et al., 2011; Feeney et al., 2011; Gorovic N et al., 2011; Lucock et al., 2011; Mennella et al., 2011a; Cabras et al., 2012; Campbell et al., 2012; Colares-Bento et al., 2012; Negri et al., 2012; Behrens et al., 2013; Inoue et al., 2013; Laaksonen et al., 2013; Melis et al., 2013; Bering et al., 2014; Garneau et al., 2014; Keller et al., 2014; Ledda et al., 2014; Mennella et al., 2014a; Robino et al., 2014; Melis et al., 2015; Nolden et al., 2016; Robino et al., 2016; Bella et al., 2017; Carrai et al., 2017; Deshaware and Singhal, 2017; Feeney et al., 2017; Risso et al., 2017). Results of the reviewed studies were congruent, supporting the genetic determination of the bitter taster status by TAS2R38 rs713598, rs1726866, and rs10246939 SNPs (Kim et al., 2003; Mennella et al., 2005; Sandell and Breslin, 2006; Sacerdote et al., 2007; Timpson et al., 2007; Hayes et al., 2008; Duffy et al., 2010; Ooi et al., 2010; Wooding et al., 2010; Calò C et al., 2011; Feeney et al., 2011; Gorovic N et al., 2011; Lucock et al., 2011; Mennella et al., 2011a; Cabras et al., 2012; Campbell et al., 2012; Colares-Bento et al., 2012; Negri et al., 2012; Behrens et al., 2013; Inoue et al., 2013; Laaksonen et al., 2013; Bering et al., 2014; Garneau et al., 2014; Keller et al., 2014; Ledda et al., 2014; Mennella et al., 2014a; Robino et al., 2014; Melis et al., 2015; Nolden et al., 2016; Robino et al., 2016; Bella et al., 2017; Carrai et al., 2017; Deshaware and Singhal, 2017; Feeney et al., 2017; Risso et al., 2017).

Much less is known about the effect of the genetic alterations of other taste 2 receptors (TAS2Rs), which proteins also function as bitter taste receptors. Respondents for TAS2R31 receptors (formerly TAS2R44) are compounds with no common chemical substructure (acesulfame K, famotidine, diphenidol) (Meyerhof et al., 2009). Research included in our review (n = 7) focused on two polymorphisms rs10845293 (Ala227Val) and rs10772423 (Val240Ile) located in this gene (Pronin et al., 2007; Roudnitzky et al., 2011; Allen et al., 2013a; Allen et al., 2013b; Hayes et al., 2015; Roudnitzky et al., 2015; Nolden et al., 2016). The Val240Ile SNP was associated with the bitter compounds amarogentin [found in gentian (*Gentiana lutea*) or in *Swertia chirata*] (Keil et al., 2000) and grosheimin [present in artichokes (Cravotto et al., 2005)] intensities, detection and recognition threshold, quinine bitterness and grapefruit liking. Moreover, Val240 homozygotes reported less bitterness from the artificial sweetener acesulfame potassium than the Ile240 homozygotes (Pronin et al., 2007; Roudnitzky et al., 2011; Allen et al., 2013a; Allen et al., 2013b; Hayes et al., 2015; Roudnitzky et al., 2015; Nolden et al., 2016). This latter finding is in accordance with *in vitro* study results, whereas acesulfame K activated TAS2R43 and TAS2R44 at concentrations known to stimulate bitter taste (Kuhn et al., 2004). The same polymorphism showed no association with bitterness of capsaicin, piperine, and ethanol (Nolden et al., 2016). The bitterness perception from capsaicin and piperine is characterized by individual diversities (Green and Hayes, 2004) and the sensitivity to perceived bitterness of alcohol correlates with PROP phenotypes (Lanier et al., 2005), but based on findings of these studies it was not related to TAS2R31 genetic variants (Nolden et al., 2016).

Two polymorphisms of the TAS2R19 gene were investigated by studies (n = 6) included in the review (Reed et al., 2010; Hayes et al., 2011; Knaapila et al., 2012; Bering et al., 2014; Hayes et al., 2015; Roudnitzky et al., 2015). TAS2R19 rs10772420 codes for an arginine-to-cysteine substitution at amino acid 299 (R299C) (Allen et al., 2013a). This variant showed associations with quinine and grosheimin intensity ratings, grosheimin detection threshold, and bitterness perception of grapefruit juice (Reed et al., 2010; Hayes et al., 2011; Knaapila et al., 2012; Hayes et al., 2015; Roudnitzky et al., 2015) and no association with PROP phenotype (Bering et al., 2014). Moreover rs1868769 in the same gene was associated with quinine and grosheimin intensity ratings and detection and recognition threshold (Knaapila et al., 2012; Roudnitzky et al., 2015), but not with PROP phenotype (Bering et al., 2014). However, in *in vitro* studies, naringin, limonin (two main compounds responsible for the bitterness of grapefruit juice), and quinine did not activate TAS2R19 (Meyerhof et al., 2009; Thalmann et al., 2013), accordingly confirmed associations may be due to strong LD between the Arg299Cys (rs10772420) polymorphism, and other SNPs located in nearby TAS2R genes (Allen et al., 2013a; Hayes et al., 2015).

Both polymorphisms T > G rs2227264 and rs2234012 (A > G) SNPs are located in the 5’ untranslated region (5’UTR) of TAS2R5, which region typically contains sequences that regulate translation efficiency or messenger RNA stability (Hayes et al., 2011), that may account for altered protein function and consecutive variation in bitterness perception.

The gustin protein (or carbonic anhydrase VI) is secreted by the parotid, submandibular, and von Ebner glands (Henkin et al., 1975; Piras et al., 2012) and it has been identified as a trophic factor for growth and development of taste buds (Henkin et al., 1999). The rs2274333 SNP causes the amino acid substitution at position Ser90Gly in the protein sequence of carbonic anhydrase VI (Henkin et al., 1975) and is associated with formation and function of fungiform papillae (Barbarossa et al., 2015).

Due to the inconclusive findings related to the gustin gene (Padiglia et al., 2010; Calò C et al., 2011; Cabras et al., 2012; Melis et al., 2013; Bering et al., 2014; Feeney and Hayes, 2014; Risso et al., 2017), and to the low number of studies focusing on TAS2R19, TAS2R31, and TAS2R5 polymorphisms, additional work is needed to determine the effect of these variants on bitter taste perception, but otherwise the confirmed effect of TAS2R38 rs713598, rs1726866, and rs10246939 SNPs shaping bitter taste preference is notable, since studies suggest a relationship between PROP sensitivity and nutritional behavior. In particular, it has been reported that taster status shows an inverse relationship with the acceptance of bitter tasting foods. Greater sensitivity to PROP is associated with lower preference of citrus fruit (Drewnowski A et al., 1998a), Brussels sprouts, cabbage and spinach (Drewnowski et al., 1999), asparagus, and curly kale (Dinehart et al., 2006) and lower overall vegetable (Drewnowski et al., 2000; Kaminski et al., 2000; Yackinous and Guinard, 2002) and fruit consumption. In other investigations, tasters showed lower acceptance of cruciferous, green and raw vegetables and supertasters higher sensitivity to dark chocolate, black coffee, and caffeine solutions (Reed et al., 2010). Since meta-analysis results provide evidence that a higher consumption of fruit and vegetables is associated with a lower risk of all-cause mortality, particularly cardiovascular mortality (Wang et al., 2014), this genetically-determined bitter phenotype is a substantial contributor to shape healthy eating patterns.

Moreover, several studies in human nutrition have suggested that the PROP phenotype may serve as a general marker for oral sensations and food preferences, and influence dietary behavior and nutritional status (Tepper, 2008). Given the nutritional importance of dietary lipids and sugars an extensive research has investigated the impact of PROP taster status on sweet and fat consumption. Most studies focusing on the relationship between taster status and dietary fat perception (Tepper and Nurse, 1997; Kirkmeyer and Tepper, 2003; Duffy et al., 2004b; Prescott et al., 2004; Hayes and Duffy, 2007; Hayes and Duffy, 2008), but not all (Drewnowski et al., 1998b; Drewnowski et al., 2007) reported that taster individuals had a lower ability to distinguish fat content and creaminess in certain fatty foods and gave higher taste intensity ratings for linoleic acid, than non-tasters (Ebba et al., 2012). Moreover, PROP non-tasters possessed preferences for dietary fat (Forrai and Bánkövi, 1994; Tepper and Nurse, 1998; Duffy, 2000; Keller et al., 2002; Hayes and Duffy, 2007) and consumed more servings of discretionary fats and high-energy foods per day compared to tasters (Keller et al., 2002; Tepper et al., 2011). Findings to elucidate the association between PROP taster status and sweet preference and sugar intake were inconclusive. Some studies found that more sensitive individuals to PROP showed lower sweet preference (Looy et al., 1992; Duffy, 2000; Hayes and Duffy, 2007; Yeomans et al., 2007). Other investigators found that sucrose tasted sweeter to tasters (Gent and Bartoshuk, 1983), but some found no link between PROP taster status and hedonic ratings for sweet (Gent and Bartoshuk, 1983; Drewnowski et al., 1997; Drewnowski et al., 2007; Von Atzingen and Silva, 2012) and the consumption sweet beverages (Wijtzes et al., 2017). Accordingly the role of bitter-taster status in shaping dietary preferences is certainly not negligible, but more research is needed to determine its effect on nutrition, besides the intake of bitter-tasting foods. Although the focus of this review was on genetic variants affecting taste, studies examining associations with PROP/PTC were not included despite their strong linkage with TAS2R38 genotype. This may have resulted in some relevant papers not being included in the analyses and discussion.

The signal transduction of sweet taste is linked to heterodimers of two G protein-coupled receptors T1R2 and T1R3) (Pronin et al., 2007; Roudnitzky et al., 2015), which are encoded by genes clustered on chromosome 1 (Liao and Schultz, 2003). TAS1R2 is characterized by an increased level of genetic diversity, furthermore TAS1R3 is more conserved (Kim et al., 2006). Candidate gene studies of sweet preference targeted the polymorphic sites located in T1R2 and T1R3 genes involved in the signal transduction of this taste modality (Fushan et al., 2009; Eny et al., 2010; Mennella et al., 2014b; Dias et al., 2015; Joseph et al., 2016; Han et al., 2017), with results not allowing further conclusions to make, since only the effect of the functional Ile191Val (rs35874116) variation (Dias et al., 2015) and the intronic rs3935570 yielded positive findings (Eny et al., 2010; Han et al., 2017) (**Table 2**). The most convincing results were related to variants in the bitter taste receptor gene (TAS2R38). These polymorphisms were reported to affect the sensory experience of sweet taste, changes in taste sensitivity and preference, and sweet food intake (Lipchock et al., 2012; Suomela et al., 2012; Keller et al., 2014; Sandell et al., 2014; Joseph et al., 2016; Pawellek et al., 2016; Perna et al., 2018) (**Table 2**), with the only exclusion a study by Ooi et al. (2010). The genetically-determined taster phenotype preferred higher sucrose concentrations (Lipchock et al., 2012), had lower detection thresholds (Joseph et al., 2016), and consumed more sweet tasting foods (Suomela et al., 2012; Keller et al., 2014; Sandell et al., 2014; Pawellek et al., 2016; Perna et al., 2018) whereas genetically determined non-taster individuals did not prefer sweet foods (Ooi et al., 2010), despite that the PROP phenotype without underlying genetic investigations showed inconclusive findings with sugar preference and intake in adults (Gent and Bartoshuk, 1983; Looy et al., 1992; Drewnowski et al., 1998b; Drewnowski et al., 1997; Duffy, 2000; Hayes and Duffy, 2007; Yeomans et al., 2007; Von Atzingen and Silva, 2012; Wijtzes et al., 2017), which is probably related to other genetic variants that influence bitter perception, and also in children that may be explained by age-related changes in taste perception and preference, beyond genetic factors (reviewed in Keller and Adise, 2016).

Although less well-studied than bitter sensitivity, variation in sweet taste responsiveness may also influence food preference and intake. It has been demonstrated that a higher preference for sucrose solutions or sweet taste was associated with increased preferences for sweet desserts (Drewnowski et al., 1999), higher habitual intake of sweet foods (Holt et al., 2000; Ashi et al., 2017), an increased consumption of sweet beverages (Mahar and Duizer, 2007), total sugar consumption (Ko et al., 2015), and the sugar content of preferred sugar-rich cereals (Mennella et al., 2011b) and more sensitive individuals tended to have a lower preference for sugar than less sensitive individuals (Looy et al., 1992). As reviewed by Rippe et al. (2016) excessive sugar intake is responsible for the development metabolically based diseases such as obesity, diabetes, and cardiovascular diseases (Rippe and Angelopoulos, 2016), therefore sweet preference has a clearly important role in determining health status.

The two SNPs rs1761667 and rs1527483, located in the CD36 gene, has received much attention in the research of fat taste perception. The fatty acid translocase, coded by the CD36 gene, is involved in the transport of LCFA across cell membranes, which is first step in fat metabolism (Hajri and Abumrad, 2002). CD36 is expressed on taste cells in animals (Fukuwatar et al., 1997; Laugerette et al., 2005) and has been detected in human foliate and circumvallate papillae (Simons et al., 2011; Hochheimer et al., 2014). Results of research, except for one single study (Daoudi et al., 2015) are consistent, namely individuals with the AA genotype of rs1761667 have higher thresholds for lipid taste perception (decrease in sensitivity and consequent higher acceptance of fatty acids) than do those with GG genotypes (Keller et al., 2012; Pepino et al., 2012; Melis et al., 2015; Mrizak et al., 2015; Sayed et al., 2015. Ong et al., 2017). The intronic SNP, rs1527483, which encodes a C/T substitution, was also found to influence fat perception by two studies. Subjects with C/T or T/T genotypes perceived greater fat content of salad dressings and cream crackers, independent of fat concentration (Keller et al., 2012; Ong et al., 2017). Although, creaminess is a complex sensory characteristic consisting of both flavor and textural components, but overall, it is experienced as a positive attribute of fat containing foods (Mela, 1988). Due to the low number studies related to SNP rs1527483 (Keller et al., 2012; Ong et al., 2017) and IZUMO1 rs838145 (Tanaka et al., 2013; Søberg et al., 2017), replication is needed to confirm the influence of these SNPs on fat taste perception.

According to investigations fat hypersensitivity is associated with lower energy and fat intake (Stewart et al., 2010) and high fat food preference with high dietary fat intake (Fisher and Birch, 1995; Ricketts, 1997). Given that adiposity is a critical risk factor in course of the development of insulin resistance and the development of type 2 diabetes (reviewed in Forouhi et al., 2018) and that atherogenic dyslipidemia [low high-density lipoprotein cholesterol (HDL-C), high triglyceride-rich lipoprotein levels] which occurs with low-fat, high carbohydrate diets and increases risk of coronary heart disease (Trumbo et al., 2002), following dietary guidelines with recommended intakes is essential to ensure adequate consumption of total energy, essential fatty acids, and fat-soluble vitamins (Food and Agriculture Organization of the United Nations/World Health Organization, 2010), and prevent cardiovascular diseases and type 2 diabetes.

The number of studies investigating salty (Hayes et al., 2008; Dias et al., 2013; Rawal et al., 2013; Feeney and Hayes, 2014; Deshaware and Singhal, 2017), umami (Raliou et al., 2009; Shigemura et al., 2009; Chen et al., 2009; Risso et al., 2017), and sour (Hayes et al., 2008; Laaksonen et al., 2013; Rawal et al., 2013; Carrai et al., 2017) taste preferences was limited. The effect of these latter two taste modalities on health status is not yet known. Though the health effects, namely the correlation of salt (sodium) intake with blood pressure is clear (reviewed in Farquhar et al., 2015), and research suggest that sensory phenotypes with greater perceived saltiness from solutions liked the solution less (Hayes et al., 2010) and individuals with a preference of high salt concentrations and salty foods were found to consume more salt compared to those who did not prefer salty beverages (Shepherd et al., 1984; Takamura et al., 2014), results of our review allows us no conclusions to make on the genetic background on salt preference.

### Limitations

Several limitations must be considered in interpreting the findings of this systematic review. Many of the results of genetic association studies on different taste modalities have not been replicated, and it is not possible to perform a qualitative synthesis and meta-analysis. Despite a growing body of nutrigenomics research, the overall number of studies available for this specific review was limited. Some studies had relatively small sample sizes, and several of them were conducted by the same research groups. Certain samples and study groups may have overlapped and participants were from similar backgrounds, without a representation of diverse populations or ethnic background. Considering the work carried out by different research teams, important factors such as genotyping method (s), assessment methods, ethnic composition, and genetic variant (s) evaluated, make direct comparison of findings hard and limit the generalizability of some results. Despite the mentioned limitations, this review represents the first systematic effort to compile and discuss studies on genetic background of taste perception, taste preferences, and its nutritional implications.

## Conclusions

Our findings suggest that a significant association exists between TAS2R38 variants (rs713598, rs1726866, rs10246939) and bitter and sweet taste preference. Due to the limited number of studies related to other tastes (salt and sour) further research is needed to assess the possible effect of TAS2R38 genetic variants on these taste modalities. Other confirmed results are related to rs1761667 (CD36) and fat taste responsiveness. Otherwise further research is essential to confirm results of single studies or clarify inconclusive findings related to genetic variants and individual sensitivity of the gustatory pathway.

Since convincing findings of genetic association studies only exists for bitter, fat, and partly for sweet taste preference, highlighting the role of environmental factors of food preferences and dietary choices has great importance in the planning of public health intervention programs. These interventions should be tailored to change the modifiable determinants of poor dietary practices to promote healthy eating. Two major areas should be recognized for nutrition policies and programs focusing on early and environmental exposures. Early exposures include experiences *in utero* and during the lactation and complementary feeding period of infants (reviewed in Beckerman et al., 2017). Research shows that maternal unhealthy food intake during pregnancy and/or lactation increases the preference for high-fat and/or high-sugar diets of the offspring (Muhlhausler et al., 2017). The timing and repeated intake of bitter tasting fruits and vegetables should be the primary focus of the complementary feeding period (reviewed in Beckerman et al., 2017), since sweet and bitter-taste preference can be influenced by early childhood experiences (Bartoshuk and Beauchamp, 1994; Mela, 2001). This is essential since sweet preference is the highest during childhood and it declines with age (reviewed in Hoffman et al., 2016) and the correlation between preferences of preschool children and their consumption patterns is considerably higher than the relationship reported by adults (Birch, 1979). Environmental exposures involve the social environment, such as parents (feeding practices and social and emotional context of food), peers, community (daycare, preschool, school, etc.), media, and other environmental effects (food access and advertisements) (reviewed in Beckerman et al., 2017). Therefore, it is important to strengthen the implementation of the International Code of Marketing of Breast-milk Substitutes (World Health Organization, 2018) and introduce restrictions on marketing of unhealthy foods to children, covering all media, including digital, and to close any regulatory loopholes, as current evidence indicates that child-directed advertising has a major impact on children’s diets (Tatlow-Golden et al., 2016; World Health Organization, 2016; Emond et al., 2019). Building combined and well-coordinated interventions encompassing all these target areas is essential in managing successful nutrition programs (reviewed in Beckerman et al., 2017).

Regardless, it is still important to emphasize that further genetic research is needed to elucidate the effect of genetic variants on food preference and nutritional behavior. This knowledge may enhance our understanding of the development of individual taste and related food preferences and food choices that will aid public health intervention programs targeting unhealthy dietary behaviors.

## Author Contributions

JD conducted the literature search (involved in all the phases of the study selection procedure), performed the quality assessment of primary studies, interpreted the results, and wrote the manuscript. EL was involved in the screening process of abstracts, assessing full-text articles for eligibility, data extraction, and quality assessment of included studies and contributed to drafting the discussion section of the manuscript. RÁ guided the writing of the manuscript and was involved in finalizing it.

## Funding

This work was supported by the GINOP-2.3.2-15-2016-00005 project. The project is co-financed by the European Union under the European Social Fund and European Regional Development Fund, as well as by the Hungarian Academy of Sciences (TK2016-78).

## Conflict of Interest

The authors declare that the research was conducted in the absence of any commercial or financial relationships that could be construed as a potential conflict of interest.
