# Supplementary material for: Genetic Background of Taste Perception, Taste Preferences, and Its Nutritional Implications: A Systematic Review
Source: Front Genet. 2019 Dec 19;10:1272. doi: 10.3389/fgene.2019.01272 (PMC6930899; doi:10.3389/fgene.2019.01272)
Supplement: Supplementary file 1 [file Table_1.docx]

**Supplementary Table 1 Overview of genetic variants associated with bitter, sweet and umami preferences**

Only those genetic polymorphisms are presented in this table, where the association was confirmed by a single study. Number of low quality studies is presented in parentheses.

SNP: single nucleotide polymorphism, PROP: 6-n-propylthiouracil, GWAS: genome-wide association study, AceK: Acesulfame Potassium, FFQ: food frequency questionnaire, DHQ: dietary history questionnaire, AUC: area under the curve, FP: fungiform papillae, CP: circumvallata papillae

**Supplementary Table 1 Overview of genetic variants associated with bitter, sweet and umami preferences**

| **Gene** | **SNP** | **Applied tastant/method** | **Number of studies with confirmed association** |  | **Findings** | **Reference** | **Number of studies with no association** | **Reference** |
| --- | --- | --- | --- | --- | --- | --- | --- | --- |
| **BITTER TASTE** |  |  |  |  |  |  |  |  |
| **DFNA5** | rs73082019 | Food preference questionnaire | 1 |  | Association with preference of dark chocolate (GWAS). | ([1](#_ENREF_1)) | 0 | - |
| **DIRC3-AS1** | rs4141835 | PROP | 1 |  | Associated with PROP intensity ratings (GWAS). | ([2](#_ENREF_2)) | 0 | - |
| **LINC02346** | rs8034691 | Food preference questionnaire | 1 |  | Associated with artichokes liking. (GWAS). | ([1](#_ENREF_1)) | 0 | - |
| **LOC105370401** | rs10137305 | PROP | 1 |  | Associated with PROP intensity ratings (GWAS). | ([3](#_ENREF_3)) | 0 | - |
| **LOC105370401** | rs11623995 | PROP | 1 |  | Associated with PROP intensity ratings (GWAS). | ([3](#_ENREF_3)) | 0 | - |
| **LOC105370401** | rs2331619 | PROP | 1 |  | Associated with PROP intensity ratings (GWAS). | ([3](#_ENREF_3)) | 0 | - |
| **LOC105370401** | rs7144549 | PROP | 1 |  | Associated with PROP intensity ratings (GWAS). | ([3](#_ENREF_3)) | 0 | - |
| **LOC105377448** | rs28849980 | Food preference questionnaire | 1 |  | Associated with artichokes liking (GWAS). | ([1](#_ENREF_1)) | 0 | - |
| **NA** | rs10050951 | Food preference questionnaire | 1 |  | Associated with artichokes liking (GWAS). | ([1](#_ENREF_1)) | 0 | - |
| **NA** | rs12200968 | PROP | 1 |  | Associated with PROP intensity ratings (GWAS). | ([3](#_ENREF_3)) | 0 | - |
| **NA** | rs138369603 | Food preference questionnaire | 1 |  | Associated with chicory liking (GWAS). | ([1](#_ENREF_1)) | 0 | - |
| **NA** | rs145671205 | Food preference questionnaire | 1 |  | Associated with coffee liking (GWAS). | ([1](#_ENREF_1)) | 0 | - |
| **NA** | rs2530184 | Food preference questionnaire | 1 |  | Associated with broccoli liking (GWAS). | ([1](#_ENREF_1)) | 0 | - |
| **NA** | rs374184 | Bitterness of Acesulfame Potassium | 1 |  | Val carriers perceived significantly more bitterness than Ala homozygotes. | ([4](#_ENREF_4)) | 0 | - |
| **NA** | rs6458845 | PROP | 1 |  | Associated with PROP intensity ratings (GWAS). | ([3](#_ENREF_3)) | 0 | - |
| **NA** | rs7746307 | PROP | 1 |  | Associated with PROP intensity ratings (GWAS). | ([3](#_ENREF_3)) | 0 | - |
| **NA** | rs9832668 | Food preference questionnaire | 1 |  | Associated with broccoli liking (GWAS). | ([1](#_ENREF_1)) | 0 | - |
| **OBP2A** | rs2590498 | PROP | 1 (1) |  | AA homozygotes perceived PROP as more bitter than the GG subjects. | ([5](#_ENREF_5)) | 0 | - |
| **PDSS2** | rs2216084 | Coffee consumption | 1 |  | Associated with coffee consumption (GWAS). | ([6](#_ENREF_6)) | 0 | - |
| **PDSS2** | rs6568479 | Coffee consumption | 1 |  | Associated with coffee consumption (GWAS). | ([6](#_ENREF_6)) | 0 | - |
| **PDSS2** | rs6942255 | Coffee consumption | 1 |  | Associated with coffee consumption (GWAS). | ([6](#_ENREF_6)) | 0 | - |
| **PDSS2** | rs7745311 | Coffee consumption | 1 |  | Associated with coffee consumption (GWAS). | ([6](#_ENREF_6)) | 0 | - |
| **PDSS2** | rs7754744 | Coffee consumption | 1 |  | Associated with coffee consumption (GWAS). | ([6](#_ENREF_6)) | 0 | - |
| **PDSS2** | rs9386630 | Coffee consumption | 1 |  | Associated with coffee consumption (GWAS). | ([6](#_ENREF_6)) | 0 | - |
| **PRH1-TAS2R14** | rs1031391 | Quinine | 1 |  | Associated with the perceived intensity of suprathreshold quinine solutions (concentration-dependent) and detection thresholds (GWAS). | ([7](#_ENREF_7)) | 0 | - |
| **PRH1-TAS2R14** | rs8181 | Caffeine | 1 |  | Associated with caffeine detection threshold (GWAS). | ([7](#_ENREF_7)) | 0 | - |
| **PRH1-TAS2R14 (TAS2R20)** | rs10845279 | Quinine | 1 |  | Associated with quinine intensity. | ([8](#_ENREF_8)) | 0 | - |
| **PRH1-TAS2R14 (TAS2R20)** | rs12226920 | Quinine | 1 |  | Associated with quinine intensity. | ([8](#_ENREF_8)) | 0 | - |
| **PRH1-TAS2R14 (TAS2R31)** | rs10772423 | Bitterness of Acesulfame Potassium | 1 |  | Ile homozygotes and heterozygotes perceived greater bitterness compared to the non-functioning Val homozygotes. | ([4](#_ENREF_4)) | 0 | - |
| **PRH1-TAS2R14 (TAS2R43)** | rs68157013 | Bitterness of aloin and saccharin | 1 (1) |  | The most aloin and saccharin sensitive individuals had at least one W35 allele. | ([9](#_ENREF_9)) | 0 | - |
| **PRH1-TAS2R14 (TAS2R46)** | rs2708377 | Bitterness of caffeine | 1 |  | Associated with the perceived bitterness of caffeine (GWAS). | ([7](#_ENREF_7)) | 0 | - |
| **PRH1-TAS2R14 (TAS2R46)** | rs2708377 | PROP, quinine and caffeine intensity, detection threshold | 1 |  | Associated with caffeine detection  threshold (GWAS). | ([7](#_ENREF_7)) | 0 | - |
| **TAS1R1** | rs17492553 | Quinine | 1 |  | TT and CT reported significantly lower intensities than CC individuals (applied to the FP and CP). | ([10](#_ENREF_10)) | 0 | - |
| **TAS1R2** | rs35874116 | Vegetable intake (3-day food record) | 1 |  | Val/Val carriers had a higher daily intake of vegetables. | ([11](#_ENREF_11)) | 0 | - |
| **TAS2R14** | rs3741843 | Detection and recognition thresholds, perceived bitter taste intensities of absinthin, amarogentin, cascarillin, grosheimin, quassin, and quinine | 1 |  | Associated with grosheimin detection threshold. | ([12](#_ENREF_12)) | 0 | - |
| **TAS2R16** | rs846664 | Salicin bitterness recognition threshold | 1 |  | Individuals with at least one derived T-allele at polymorphic site 516 have a higher sensitivity to salicin bitterness compared with individuals homozygous for the ancestral G-allele. | ([13](#_ENREF_13)) | 1 | ([14](#_ENREF_14)) (PROP) |
| **TAS2R16** | rs860170 | Food habits questionnaire (liking), PROP tasting, streptovisine, salicin bitterness | 1 |  | The perception of salicin bitterness was associated with A allele. At the genotypic level, a notable trend can be was observed (bitterness levels: AA> GG). | ([15](#_ENREF_15)) | 1 | ([14](#_ENREF_14)) (PROP) |
| **TAS2R19** | rs12313469 | Detection and recognition thresholds, perceived bitter taste intensities of absinthin, amarogentin, cascarillin, grosheimin, quassin, and quinine | 1 |  | Associated with grosheimin detection threshold and intensities (weak, moderate, strong, very strong). | ([12](#_ENREF_12)) | 0 | - |
| **TAS2R20** | rs11054143 | Detection and recognition thresholds, perceived bitter taste intensities of absinthin, amarogentin, cascarillin, grosheimin, quassin, and quinine | 1 |  | Associated with grosheimin intensities (strong, very strong). | ([12](#_ENREF_12)) | 0 | - |
| **TAS2R20** | rs7135018 | Detection and recognition thresholds, perceived bitter taste intensities of absinthin, amarogentin, cascarillin, grosheimin, quassin, and quinine | 1 |  | Associated with grosheimin intensities (strong, very strong) and quinine detection threshold. | ([12](#_ENREF_12)) | 0 | - |
| **TAS2R20** | rs10845281 | Detection and recognition thresholds, perceived bitter taste intensities of absinthin, amarogentin, cascarillin, grosheimin, quassin, and quinine | 1 |  | Associated with grosheimin intensities (strong, very strong). | ([12](#_ENREF_12)) | 0 | - |
| **TAS2R20** | rs11054142 | Detection and recognition thresholds, perceived bitter taste intensities of absinthin, amarogentin, cascarillin, grosheimin, quassin, and quinine | 1 |  | Associated with grosheimin intensities (strong, very strong). | ([12](#_ENREF_12)) | 0 | - |
| **TAS2R20** | rs12226920 | Detection and recognition thresholds, perceived bitter taste intensities of absinthin, amarogentin, cascarillin, grosheimin, quassin, and quinine | 1 |  | Associated with grosheimin intensities (strong, very strong). | ([12](#_ENREF_12)) | 0 | - |
| **TAS2R20** | rs12226919 | Detection and recognition thresholds, perceived bitter taste intensities of absinthin, amarogentin, cascarillin, grosheimin, quassin, and quinine | 1 |  | Associated with grosheimin intensities (strong, very strong). | ([12](#_ENREF_12)) | 0 | - |
| **TAS2R20** | rs79420812 | Detection and recognition thresholds, perceived bitter taste intensities of absinthin, amarogentin, cascarillin, grosheimin, quassin, and quinine | 1 |  | Associated with grosheimin intensities (strong, very strong). | ([12](#_ENREF_12)) | 0 | - |
| **TAS2R20** | rs10845279 | Detection and recognition thresholds, perceived bitter taste intensities of absinthin, amarogentin, cascarillin, grosheimin, quassin, and quinine | 1 |  | Associated with grosheimin intensities (strong, very strong). | ([12](#_ENREF_12)) | 0 | - |
| **TAS2R20** | rs10845280 | Detection and recognition thresholds, perceived bitter taste intensities of absinthin, amarogentin, cascarillin, grosheimin, quassin, and quinine | 1 |  | Associated with grosheimin intensities (strong, very strong). | ([12](#_ENREF_12)) | 0 | - |
| **TAS2R3** | rs765007 | Bitterness of unsweetened grapefruit juice, instant espresso | 1 |  | Haplotype, allelic variation (TAS2R3, -R4, and -R5) explained variability in coffee bitterness (individuals with 1 or 2 copies of the more responsive haplotype (TGAG) experienced twice as much bitterness compared with individuals homozygous for the less responsive haplotype (CCGT), but these haplotypes did not predict coffee liking. | ([16](#_ENREF_16)) | 0 | - |
| **TAS2R30** | H1 haplotype | Acesulfame Potassium, saccharin, salicin and denatonium benzoate recognition threshold | 1 |  | H1 haplotype associated with responses to saccharin and Acesulfame Potassium. | ([17](#_ENREF_17)) | 0 | - |
| **TAS2R30** | rs2600355 | Detection and recognition thresholds, perceived bitter taste intensities of absinthin, amarogentin, cascarillin, grosheimin, quassin, and quinine | 1 |  | Associated with amarogentin weak intensity, drosheimin detection threshold and intensities (weak, moderate, strong, very strong). | ([12](#_ENREF_12)) | 0 | - |
| **TAS2R30** | rs2599404 | Detection and recognition thresholds, perceived bitter taste intensities of absinthin, amarogentin, cascarillin, grosheimin, quassin, and quinine | 1 |  | Associated with amarogentin weak intensity, drosheimin detection threshold and intensities (weak, moderate, strong, very strong). | ([12](#_ENREF_12)) | 0 | - |
| **TAS2R30-H1, TAS2R45-H1, TAS2R43-D, TAS2R46-H2, TAS2R31-H2** | LRH21111 | Acesulfame Potassium, saccharin, salicin and denatonium benzoate recognition threshold | 1 |  | LRH11D22 (composed of the five single-gene haplotypes associated with low threshold, which were in tight LD: TAS2R30-H1, TAS2R45-H1, TAS2R43-D, TAS2R46-H2 and TAS2R31-H2) was associated with low threshold response (i.e. high sensitivity) to Acesulfame Potassium and saccharin. | ([17](#_ENREF_17)) | 0 | - |
| **TAS2R31 (formerly TAS2R44)** | H2 haplotype | Acesulfame Potassium, saccharin, salicin and denatonium benzoate recognition threshold | 1 |  | H2 haplotype associated with responses to saccharin and Acesulfame Potassium. | ([17](#_ENREF_17)) | 0 | - |
| **TAS2R31 (formerly TAS2R44)** | rs10845294 | Bitterness of Acesulfame Potassium | 1 |  | The SNP mediates the bitterness of AceK. | ([4](#_ENREF_4)) | 1 | (18) (PROP) |
| **TAS2R31 (formerly TAS2R44)** | rs12370363 | Detection and recognition thresholds, perceived bitter taste intensities of absinthin, amarogentin, cascarillin, grosheimin, quassin, and quinine | 1 |  | Associated with grosheimin intensities (strong, very strong). | ([12](#_ENREF_12)) | 0 | - |
| **TAS2R31 (formerly TAS2R44)** | rs10743938 | Detection and recognition thresholds, perceived bitter taste intensities of absinthin, amarogentin, cascarillin, grosheimin, quassin, and quinine | 1 |  | Associated with grosheimin detection threshold, recognition threshold and intensities (weak, moderate). | ([12](#_ENREF_12)) | 0 | - |
| **TAS2R42** | rs1650019 | Detection and recognition thresholds, perceived bitter taste intensities of absinthin, amarogentin, cascarillin, grosheimin, quassin, and quinine | 1 |  | Associated with grosheimin detection threshold. | ([12](#_ENREF_12)) | 0 | - |
| **TAS2R42** | rs1669413 | Detection and recognition thresholds, perceived bitter taste intensities of absinthin, amarogentin, cascarillin, grosheimin, quassin, and quinine | 1 |  | Associated with grosheimin detection threshold. | ([12](#_ENREF_12)) | 0 | - |
| **TAS2R42** | rs1650017 | Detection and recognition thresholds, perceived bitter taste intensities of absinthin, amarogentin, cascarillin, grosheimin, quassin, and quinine | 1 |  | Associated with grosheimin detection threshold. | ([12](#_ENREF_12)) | 0 | - |
| **TAS2R42** | rs1669411 | Detection and recognition thresholds, perceived bitter taste intensities of absinthin, amarogentin, cascarillin, grosheimin, quassin, and quinine | 1 |  | Associated with grosheimin detection threshold. | ([12](#_ENREF_12)) | 0 | - |
| **TAS2R43** | rs71443637 | Detection and recognition thresholds, perceived bitter taste intensities of absinthin, amarogentin, cascarillin, grosheimin, quassin, and quinine | 1 |  | Associated with grosheimin detection threshold and intensities (weak, moderate, strong, very strong). | ([12](#_ENREF_12)) | 0 | - |
| **TAS2R43** | rs68157013 | Salicin recognition threshold | 1 |  | Associated with saccharin response. | ([17](#_ENREF_17)) | 0 | - |
| **TAS2R43** | rs35720106 | Detection and recognition thresholds, perceived bitter taste intensities of absinthin, amarogentin, cascarillin, grosheimin, quassin, and quinine | 1 |  | Associated with grosheimin detection threshold and intensities (weak, moderate, strong, very strong). | ([12](#_ENREF_12)) | 0 | - |
| **TAS2R43** | D haplotype | Acesulfame Potassium, saccharin recognition threshold | 1 |  | Associated with saccharin and Acesulfame Potassium response. | ([17](#_ENREF_17)) | 0 | - |
| **TAS2R43** | Deletion | Detection and recognition thresholds, perceived bitter taste intensities of absinthin, amarogentin, cascarillin, grosheimin, quassin, and quinine | 1 |  | Associated with amarogentin weak intensity, grosheimin detection threshold and intensities (weak, moderate, strong, very strong). | ([12](#_ENREF_12)) | 0 | - |
| **TAS2R43** | rs68157013 | Detection and recognition thresholds, perceived bitter taste intensities of absinthin, amarogentin, cascarillin, grosheimin, quassin, and quinine | 1 |  | Associated with grosheimin detection threshold and intensities (weak, moderate, strong, very strong). | ([12](#_ENREF_12)) | 0 | - |
| **TAS2R43** | rs35720106 | Coffee liking | 1 |  | Associated with coffee liking. | ([19](#_ENREF_18)) | 0 | - |
| **TAS2R43** | rs68157013 | Coffee liking | 1 |  | Associated with coffee liking. | ([19](#_ENREF_18)) | 0 | - |
| **TAS2R43** | rs71443637 | Coffee liking | 1 |  | Associated with coffee liking. | ([19](#_ENREF_18)) | 0 | - |
| **TAS2R45** | rs11535673 | Detection and recognition thresholds, perceived bitter taste intensities of absinthin, amarogentin, cascarillin, grosheimin, quassin, and quinine | 1 |  | Associated with grosheimin detection threshold. and intensities (moderate, strong, very strong). | ([12](#_ENREF_12)) | 0 | - |
| **TAS2R45** | rs3759247 | Detection and recognition thresholds, perceived bitter taste intensities of absinthin, amarogentin, cascarillin, grosheimin, quassin, and quinine | 1 |  | Associated with grosheimin detection threshold. and intensities (moderate, strong, very strong). | ([12](#_ENREF_12)) | 0 | - |
| **TAS2R45** | rs3759245 | Detection and recognition thresholds, perceived bitter taste intensities of absinthin, amarogentin, cascarillin, grosheimin, quassin, and quinine | 1 |  | Associated with grosheimin detection threshold. and very strong intensity. | ([12](#_ENREF_12)) | 0 | - |
| **TAS2R45** | Deletion | Detection and recognition thresholds, perceived bitter taste intensities of absinthin, amarogentin, cascarillin, grosheimin, quassin, and quinine | 1 |  | Associated with grosheimin detection threshold. | ([12](#_ENREF_12)) | 0 | - |
| **TAS2R45** | rs11526470 | Detection and recognition thresholds, perceived bitter taste intensities of absinthin, amarogentin, cascarillin, 1grosheimin, quassin, and quinine | 1 |  | Associated with grosheimin detection threshold and intensities (moderate, strong, very strong). | ([12](#_ENREF_12)) | 0 | - |
| **TAS2R45** | rs28581524 | Detection and recognition thresholds, perceived bitter taste intensities of absinthin, amarogentin, cascarillin, grosheimin, quassin, and quinine | 1 |  | Associated with grosheimin detection threshold. and intensities (moderate, strong, very strong). | ([12](#_ENREF_12)) | 0 | - |
| **TAS2R45** | rs11537119 | Detection and recognition thresholds, perceived bitter taste intensities of absinthin, amarogentin, cascarillin, grosheimin, quassin, and quinine | 1 |  | Associated with grosheimin detection threshold. and intensities (moderate, strong, very strong). | ([12](#_ENREF_12)) | 0 | - |
| **TAS2R45** | H1 haplotype | Acesulfame Potassium, saccharin recognition threshold | 1 |  | TAS2R45-H1 associated with responses to saccharin and Acesulfame Potassium. | ([17](#_ENREF_17)) | 0 | - |
| **TAS2R45** | rs3759244 | Detection and recognition thresholds, perceived bitter taste intensities of absinthin, amarogentin, cascarillin, grosheimin, quassin, and quinine | 1 |  | Associated with grosheimin detection threshold. and intensities (moderate, strong, very strong). | ([12](#_ENREF_12)) | 0 | - |
| **TAS2R46** | rs2708381 | Detection and recognition thresholds, Perceived bitter taste intensities of absinthin, amarogentin, cascarillin, grosheimin, quassin, and quinine | 1 |  | Associated with quinine detection threshold. | ([12](#_ENREF_12)) | 0 | - |
| **TAS2R46** | H2 haplotype | Acesulfame Potassium, saccharin, recognition threshold | 1 |  | TAS2R46-H2 associated with responses to saccharin and Acesulfame Potassium. | ([17](#_ENREF_17)) | 0 | - |
| **TAS2R46** | rs2708380 | Detection and recognition thresholds, Perceived bitter taste intensities of absinthin, amarogentin, cascarillin, grosheimin, quassin, and quinine | 1 |  | Associated with amarogentin weak intensity, grosheimin detection threshold and intensities (weak, moderate, strong, very strong). | ([12](#_ENREF_12)) | 0 | - |
| **TAS2R50** | rs1376251 | Detection and recognition thresholds, Perceived bitter taste intensities of absinthin, amarogentin, cascarillin, grosheimin, quassin, and quinine | 1 |  | Associated with grosheimin strong, very strong intensity. | ([12](#_ENREF_12)) | 0 | - |
| **TAS2R8** | rs1548803 | Quinine | 1 |  | Associated with quinine intensity. | ([8](#_ENREF_8)) | 0 | - |
|  |  |  |  |  |  |  |  |  |
| **TATDN2** | rs146768860 | PROP | 1 |  | Associated with PROP phenotype (GWAS). | ([3](#_ENREF_3)) | 0 | - |
| **TATDN2** | rs2003595 | PROP | 1 |  | Associated with PROP phenotype (GWAS). | ([3](#_ENREF_3)) | 0 | - |
| **TATDN2** | rs2005903 | PROP | 1 |  | Associated with PROP phenotype (GWAS). | ([3](#_ENREF_3)) | 0 | - |
| **TATDN2** | rs2241313 | PROP | 1 |  | Associated with PROP phenotype (GWAS). | ([3](#_ENREF_3)) | 0 | - |
| **TATDN2** | rs2241314 | PROP | 1 |  | Associated with PROP phenotype (GWAS). | ([3](#_ENREF_3)) | 0 | - |
| **TATDN2** | rs2270454 | PROP | 1 |  | Associated with PROP phenotype (GWAS). | ([3](#_ENREF_3)) | 0 | - |
| **TATDN2** | rs56284018 | PROP | 1 |  | Associated with PROP phenotype (GWAS). | ([3](#_ENREF_3)) | 0 | - |
| **TATDN2** | rs78537477 | PROP | 1 |  | Associated with PROP phenotype (GWAS). | ([3](#_ENREF_3)) | 0 | - |
| **TRPV1** | rs224547 | Bitterness of alcohol | 1 |  | Associated with the summary AUC scores for bitterness of alcohol (AA homozygotes having the highest mean area). | ([20](#_ENREF_20)) | 0 | - |
| **TRPV1** | rs4790521 | Bitterness of alcohol | 1 |  | Associated with the summary AUC scores for bitterness of alcohol (CC homozygotes had the highest mean area for bitterness). | ([20](#_ENREF_20)) | 0 | - |
| **ZNF804B** | rs4727180 | PROP | 1 |  | Associated with PROP phenotype. | ([2](#_ENREF_2)) | 0 | - |
| **DFNA5** | rs73082019 | Food preference questionnaire | 1 |  | Association with preference of dark chocolate (GWAS). | ([6](#_ENREF_6)) | 0 | - |
| **DIRC3-AS1** | rs4141835 | PROP | 1 |  | Associated with PROP intensity ratings (GWAS). | ([2](#_ENREF_2)) | 0 | - |

| **SWEET TASTE** |  |  |  |  |  |  |  |
| --- | --- | --- | --- | --- | --- | --- | --- |
| **ADIPOQ** | rs822396 | Confectionery-intake score (FFQ) | 1 | Association with confectionery-intake score, but it did not reach genome-wide significance level (GWAS). | ([21](#_ENREF_21)) | 0 | - |
| **ANKK1** | rs1800497 | Sucrose | 1 | A1 alleles associated with sucrose preference. | ([22](#_ENREF_22)) | 1 | ([23](#_ENREF_23)) |
| **DRD2** | rs6277 | Sugar intake (FFQ) | 1 | Sucrose consumption was associated with genotypes (CC>CT>TT) among men. Consumption of total sugars, sucrose and fructose was associated with genotypes (CT>TT>CC) among women. | ([24](#_ENREF_24)) | 0 | - |
| **FGF21** | rs838133 | Sweet intake (FFQ) | 1 | A-allele increased the odds ratio of the consumption of candy. | ([25](#_ENREF_25)) | 0 | - |
| **GNAT3** | rs6467192 | Sucrose | 1 | Correlation with sucrose AUC scores. | ([26](#_ENREF_26)) | 0 | - |
| **GNAT3** | rs1524600 | Sucrose | 1 | SNP correlated with sucrose AUC scores and haplotypes (combination of high-sensitivity alleles of rs7792845 and rs1524600), associated with higher sensitivity to sucrose. | ([26](#_ENREF_26)) | 0 | - |
| **GNAT3** | rs6467217 | Sucrose | 1 | Correlation with sucrose AUC scores. | ([26](#_ENREF_26)) | 0 | - |
| **GNAT3** | rs6970109 | Sucrose | 1 | Correlation with sucrose AUC scores. | ([26](#_ENREF_26)) | 0 | - |
| **GNAT3** | rs6975345 | Sucrose | 1 | Correlation with sucrose AUC scores. | ([26](#_ENREF_26)) | 0 | - |
| **GNAT3** | rs10242727 | Sucrose | 1 | Correlation with sucrose AUC scores. | ([26](#_ENREF_26)) | 0 | - |
| **GNAT3** | rs6961082 | Sucrose | 1 | Correlation with sucrose AUC scores. | ([26](#_ENREF_26)) | 0 | - |
| **GNAT3** | rs6979450 | Sucrose | 1 | Correlation with sucrose AUC scores. | ([26](#_ENREF_26)) | 0 | - |
| **GNAT3** | rs7776757 | Sucrose | 1 | Correlation with sucrose AUC scores. | ([26](#_ENREF_26)) | 0 | - |
| **LEP** | rs2167270 | Sweet preference questionnaire | 1 | Association with sweet preference. | ([27](#_ENREF_27)) | 0 | - |
| **LEPR** | rs1137100 | Sweet preference questionnaire | 1 | Association with sweet preference. | ([27](#_ENREF_27)) | 1 | ([28](#_ENREF_28)) |
| **LOC107986812** | rs7792845 | Sucrose | 1 | Correlation with sucrose AUC scores. | ([26](#_ENREF_26)) | 1 | ([29](#_ENREF_29) children) |
| **LOC107986812** | rs940541 | Sucrose | 1 | Correlation with sucrose AUC scores. | ([26](#_ENREF_26)) | 0 | - |
| **LOC107986812** | rs1107660 | Sucrose | 1 | Correlation with sucrose AUC scores. | ([26](#_ENREF_26)) | 0 | - |
| **LOC107986812** | rs1107657 | Sucrose | 1 | Correlation with sucrose AUC scores. | ([26](#_ENREF_26)) | 0 | - |
| **LOC107986812** | rs2012380 | Sucrose | 1 | Correlation with sucrose AUC scores. | ([26](#_ENREF_26)) | 0 | - |
| **NA** | rs17724320 | Intake of mono and disaccharides (FFQ) | 1 | Dose-response relationship with mono- and disaccharide intake as for total carbohydrates for the derived allele. | ([30](#_ENREF_30)) | 0 | - |
| **NPY1R** | rs11100489 | Intake of mono and disaccharides (FFQ) | 1 | Dose-response relationship with mono- and disaccharide intake as for total carbohydrates for the derived allele. | ([30](#_ENREF_30)) | 0 | - |
| **NPY1R** | rs12507653 | Intake of mono and disaccharides (FFQ) | 1 | AA or AT vs. TT men consumed less mono- and disaccharides. | ([30](#_ENREF_30)) | 0 | - |
| **NPY1R** | rs4234955 | Intake of mono and disaccharides (FFQ) | 1 | Dose-response relationship with mono- and disaccharide intake as for total carbohydrates for the derived allele. | ([30](#_ENREF_30)) | 0 | - |
| **NPY2R** | rs12507396 | Intake of mono and disaccharides (FFQ) | 1 | Dose-response relationship with mono- and disaccharide intake as for total carbohydrates for the derived allele. | ([30](#_ENREF_30)) | 0 | - |
| **OPRM1** | rs1799971 | Food preference questionnaire | 1 | GG associated with stronger sweet preference. | ([31](#_ENREF_31)) | 0 | - |
| **OPRM1** | rs495491 | Food preference questionnaire | 1 | GA reported a lower sweet preference. The A–C haplotype (rs495491, rs563649) was associated with stronger sweet preference than the other two common haplotypes. | ([31](#_ENREF_31)) | 0 | - |
| **OPRM1** | rs563649 | Food preference questionnaire | 1 | The A–C haplotype (rs495491, rs563649) was associated with stronger sweet preference than the other two common haplotypes. | ([31](#_ENREF_31)) | 0 | - |
| **OXTR** | rs2268494 | Food preference questionnaire | 1 | AA or AT vs. TT associated with stronger preference for sweet and fatty foods. | ([32](#_ENREF_32)) | 0 | - |
| **SLC2A2** | rs5400 | Sugar intake (FFQ) | 1 | Intake of sugars, sucrose, fructose, glucose, sweetened beverages was greater among carriers of the Ile allele compared with those with the Thr/Thr genotype. | ([33](#_ENREF_33)) | 1 |  |
| **TAS1R1** | rs17492553 | Sucrose | 1 | TT associated with lower intensities than CC (regional application on FP). TT, CT reported lower intensities than CC (regional application on CP). | ([10](#_ENREF_10)) | 0 | - |
| **TAS1R1** | rs34160967 | Sucrose | 1 | AA/AG associated with lower intensities than GG homozygotes (regional application on CP). | ([10](#_ENREF_10)) | 0 | - |
| **TRPV1** | rs8065080 | Sweet taste preference | 1 | Minor allele positively associated with sweet preference. | ([34](#_ENREF_34)) | 0 | - |
| **TRPV1** | rs161364 | Sweet taste preference | 1 | Minor allele positively associated with sweet preference. | ([34](#_ENREF_34)) | 0 | - |

| **FAT TASTE** |  |  |  |  |  |  |  |
| --- | --- | --- | --- | --- | --- | --- | --- |
| **ADRB3** | rs4994 | Intake of lipids (FFQ) | 1 | T/C; TT vs. CC/TC linked to higher lipid intakes, including fatty acids and cholesterol. | ([35](#_ENREF_35)) | 0 | - |
| **APOA2** | rs5082 | Fat intake (DHQ) | 1 | CC vs TC/TT linked to higher total fat intake expressed as percentage of daily energy intake. | ([36](#_ENREF_36)) | 0 | - |
| **OPRM1** | rs1799971 | Food preference questionnaire | 1 | GG stronger fat preferences. | ([31](#_ENREF_31)) | 0 | - |
| **OPRM1** | rs495491 | Food preference questionnaire | 1 | GG vs. GA and AA reported lower fat preference. | ([31](#_ENREF_31)) | 0 | - |
| **RGS6** | rs847330 | Fat intake (FFQ) | 1 | Associated with the frequency of servings of fats/oils/sweets and modestly associated with total fat and saturated fat intake. | ([37](#_ENREF_37)) | 0 | - |
| **RGS6** | rs847328 | Fat intake (FFQ) | 1 | Associated with the frequency of servings of fats/oils/sweets. | ([37](#_ENREF_37)) | 0 | - |
| **RGS6** | rs769148 | Fat intake (FFQ) | 1 | Associated with the frequency of servings of fats/oils/sweets. | ([37](#_ENREF_37)) | 0 | - |
| **AGT** | rs699 | Intake of lipids (FFQ) | 1 | MM/MT vs. TT associated with higher intake of total lipids. | ([35](#_ENREF_35)) | 0 | - |
| **BPNT1** | rs6661761 | Food preference (categories: vegetables, fatty, dairy and bitter) | 1 | Association with oil or butter on bread liking (GWAS). | ([6](#_ENREF_6)) | 0 | - |
| **CNR1** | rs1049353 | Fat intake (FFQ) | 1 | The SNP inversely associated with the intake of dietary cholesterol and saturated fats. | ([38](#_ENREF_38)) | 0 | - |
| **CNTN5** | rs140738262 | Food preference (categories: vegetables, fatty, dairy and bitter) | 1 | Association with bacon liking (GWAS). | ([6](#_ENREF_6)) | 0 | - |
| **FGF21** | rs838133 | Fat intake (FFQ) | 1 | Tendency toward decreased total fat intake (A allele carriers), (MUFAs, PUFAs, omega-3 fatty acids). | ([25](#_ENREF_25)) | 0 | - |
| **OXTR** | rs2268494 | Food preference questionnaire | 1 | A allele carriers stronger preference for fatty foods. | ([32](#_ENREF_32)) | 0 | - |
| **RGS6** | rs847354 | Fat intake (FFQ) | 1 | Modestly associated with intake frequency of fats/oils/sweets, total fat and saturated fat intake. | ([37](#_ENREF_37)) | 0 | - |
| **RGS6** | rs2239247 | Fat intake (FFQ) | 1 | Associated with intake frequency of fats/oils/ sweets (servings/day). | ([37](#_ENREF_37)) | 0 | - |
| **RGS6** | rs1402064 | Fat intake (FFQ) | 1 | Associated with the intake frequency of servings of fats/oils/sweets and total fat intake, saturated fat intake, percent of calories from fat. | ([37](#_ENREF_37)) | 0 | - |
| **SH2B1** | rs7498665 | Fat intake (FFQ) | 1 | Associated with fat and saturated fat intakes. | ([39](#_ENREF_39)) | 0 | - |
| **TAS2R38** | A49P (rs713598), A262V (rs1726866), V296I (rs10246939) | Oleic acid threshold | 1 | In interaction with rs1761667 and rs1527483. | ([40](#_ENREF_40)) | 0 | - |
