# Supplementary material for: Genetic Background of Taste Perception, Taste Preferences, and Its Nutritional Implications: A Systematic Review
Source: Front Genet. 2019 Dec 19;10:1272. doi: 10.3389/fgene.2019.01272 (PMC6930899; doi:10.3389/fgene.2019.01272)
Supplement: Supplementary file 2 [file Table_2.docx]

**Supplementary Table 2 Overview of genetic variants with no confirmed associations related to taste preferences**

SNP: single nucleotide polymorphism, PROP: 6-n-propylthiouracil, FFQ: food frequency questionnaire, SQFFQ: semi-quantitative food frequency questionnaire, AceK: Acesulfame Potassium

**Supplementary Table 2 Overview of genetic variants with no confirmed associations related to taste preferences**

| **Gene** | **SNP** | **Applied tastant/method** | **Number of studies with confirmed association** | **Number of studies with no association (number of low quality studies, if applicable)** | **Reference** |
| --- | --- | --- | --- | --- | --- |
| **BITTER TASTE** |  |  |  |  |  |
| **CA4** | rs9905484 | Quinine | 0 | 1 | ([8](#_ENREF_8)) |
| **CA6** | rs12748400 | PROP | 0 | 1 | ([41](#_ENREF_41)) |
| **CA6** | rs17032907 | PROP | 0 | 1 | ([41](#_ENREF_41)) |
| **CA6** | rs2274327 | PROP | 0 | 1 | ([41](#_ENREF_41)) |
| **CA6** | rs2274328 | PROP | 0 | 1 | ([41](#_ENREF_41)) |
| **CA6** | rs2274333 | Quinine | 0 | 1 | ([8](#_ENREF_8)) |
| **CA6** | rs2274334 | PROP | 0 | 1 | ([41](#_ENREF_41)) |
| **CA6** | rs3737665 | PROP | 0 | 1 | ([41](#_ENREF_41)) |
| **CA6** | rs3765964 | PROP | 0 | 1 | ([41](#_ENREF_41)) |
| **CA6** | rs3765965 | PROP | 0 | 1 | ([41](#_ENREF_41)) |
| **CA6** | rs3765967 | PROP | 0 | 1 | ([41](#_ENREF_41)) |
| **CA6** | rs3765968 | PROP | 0 | 1 | ([41](#_ENREF_41)) |
| **CA6** | rs7545200 | PROP | 0 | 1 | ([41](#_ENREF_41)) |
| **CASC6** | rs10485099 | Quinine | 0 | 1 | ([8](#_ENREF_8)) |
| **CD36** | rs1527483 | PROP | 0 | 1 | ([40](#_ENREF_40)) |
| **CD36** | rs1761667 | PROP | 0 | 1 | ([40](#_ENREF_40)) |
| **CDYL2** | rs13333767 | Quinine | 0 | 1 | ([8](#_ENREF_8)) |
| **FFAR1** | rs2301151 | Quinine | 0 | 1 | ([8](#_ENREF_8)) |
| **GNAT3** | rs1524600 | Quinine | 0 | 1 | ([8](#_ENREF_8)) |
| **INTS4** | rs3819256 | Quinine | 0 | 1 | ([8](#_ENREF_8)) |
| **KCNJ5** | rs4937384 | Quinine | 0 | 1 | ([8](#_ENREF_8)) |
| **LOC105377272** | rs4127802 | Quinine | 0 | 1 | ([8](#_ENREF_8)) |
| **LOC107986812** | rs7792845 | Quinine | 0 | 1 | ([8](#_ENREF_8)) |
| **NA** | rs10966900 | Quinine | 0 | 1 | ([8](#_ENREF_8)) |
| **NA** | rs1308724 | Bitterness of Acesulfame Potassium, quinine | 0 | 3 | ([4](#_ENREF_4), 18, 8) |
| **NA** | rs4481887 | Quinine | 0 | 1 | ([8](#_ENREF_8)) |
| **NA** | rs846672 | Quinine | 0 | 1 | ([8](#_ENREF_8)) |
| **OR11H7** | rs1953558 | Quinine | 0 | 1 | ([8](#_ENREF_8)) |
| **OR7D4** | rs5020278 | Quinine | 0 | 1 | ([8](#_ENREF_8)) |
| **OR7D4** | rs61729907 | Quinine | 0 | 1 | ([8](#_ENREF_8)) |
| **PRH1-TAS2R14 (TAS2R31)** | rs10845293 | PROP | 0 | 1 | ([14](#_ENREF_14)) |
| **PRH1-TAS2R14 (TAS2R50)** | rs10772397 | Quinine | 0 | 1 | ([8](#_ENREF_8)) |
| **SCNN1D** | rs586965 | Quinine | 0 | 1 | ([8](#_ENREF_8)) |
| **SRPK2** | rs1204064 | PROP | 0 | 1 | ([14](#_ENREF_14)) |
| **TAS1R1** | rs34160967 | Quinine | 0 | 1 | ([10](#_ENREF_10)) |
| **TAS1R1** | rs34160967 | Quinine | 0 | 1 | ([8](#_ENREF_8)) |
| **TAS1R2** | rs35874116 | Quinine | 0 | 1 | ([8](#_ENREF_8)) |
| **TAS1R2** | rs9701796 | Quinine | 0 | 1 | ([8](#_ENREF_8)) |
| **TAS1R3** | rs111615792 | Quinine | 0 | 1 | ([8](#_ENREF_8)) |
| **TAS1R3** | rs307355 | Quinine | 0 | 1 | ([8](#_ENREF_8)) |
| **TAS1R3** | rs307377 | Quinine | 0 | 1 | ([8](#_ENREF_8)) |
| **TAS1R3** | rs35744813 | Quinine | 0 | 1 | ([8](#_ENREF_8)) |
| **TAS1R3** | rs76755863 | Quinine | 0 | 1 | ([8](#_ENREF_8)) |
| **TAS2R1** | rs2234233 | Quinine | 0 | 1 | ([8](#_ENREF_8)) |
| **TAS2R13** | rs1015443 | Bitterness of capsaicin, piperine, ethanol | 0 | 1 | (41) |
| **TAS2R13** | rs1015443 | PROP, bitterness of alcohol | 0 | 2 | ([14](#_ENREF_14), [20](#_ENREF_20)) |
| **TAS2R16** | rs2233989 | PROP | 0 | 1 | ([14](#_ENREF_14)) |
| **TAS2R16** | rs978739 | PROP | 0 | 1 | ([14](#_ENREF_14)) |
| **TAS2R19** | rs12578654 | PROP | 0 | 1 | ([14](#_ENREF_14)) |
| **TAS2R19** | rs4763235 | PROP | 0 | 1 | ([14](#_ENREF_14)) |
| **TAS2R3** | rs2270009 | Quinine | 0 | 1 | ([8](#_ENREF_8)) |
| **TAS2R3** | rs765007 | Bitterness of capsaicin, piperine, ethanol threshold | 0 | 1 | ([19](#_ENREF_19)) |
| **TAS2R39** | rs4726600 | Quinine | 0 | 1 | ([8](#_ENREF_8)) |
| **TAS2R4** | rs2233998 | PROP, bitterness of capsaicin, piperine, ethanol | 0 | 2 | (41, [14](#_ENREF_14)) |
| **TAS2R4** | rs2234002 | Bitterness of capsaicin, piperine, ethanol | 0 | 1 | (41) |
| **TAS2R60** | rs4595035 | Quinine | 0 | 1 | ([8](#_ENREF_8)) |
| **TRPA1** | rs7827617 | Quinine | 0 | 1 | ([8](#_ENREF_8)) |
| **TRPA1** | rs11988795 | Quinine | 0 | 1 | ([8](#_ENREF_8)) |
| **TRPM5** | rs2301699 | Quinine | 0 | 1 | ([8](#_ENREF_8)) |
| **TRPM8** | rs7593557 | Quinine | 0 | 1 | ([8](#_ENREF_8)) |
| **TRPV1** | rs4790522 | Quinine | 0 | 1 | ([8](#_ENREF_8)) |
| **TRPV1** | rs8065080 | Quinine | 0 | 1 | ([8](#_ENREF_8)) |
| **SWEET TASTE** |  |  |  |  |  |
| **TAS1R2** | rs35874116 | Sucrose | 0 | 1 | ([4](#_ENREF_42)3) |
| **TAS2R31** | rs10772423 | Intensity ratings (test samples: sucrose, gentiobiose, aspartame, rebaudioside A and D, AceK sweetness) | 0 | 2 | ([4](#_ENREF_4), 18) |
| **TAS2R4** | rs2234001 | Intensity ratings (test samples: sucrose, gentiobiose, aspartame, rebaudioside A and D) | 0 | 1 | ([4](#_ENREF_4)) |
| **TAS2R9** | rs3741845 | AceK sweetness | 0 | 1 | (18) |
| **FAT TASTE** |  |  |  |  |  |
| **ADIPOQ-AS1** | rs2241766 | Fat intake (7-day food records) | 0 | 1 | ([4](#_ENREF_43)4) |
| **BDNF-AS** | rs1488830 | Fat intake (FFQ) | 0 | 1 | ([39](#_ENREF_39)) |
| **BDNF-AS** | rs925946 | Fat intake (FFQ) | 0 | 1 | ([39](#_ENREF_39)) |
| **CAV3** | rs237878 | Food preference questionnaire | 0 | 1 | ([32](#_ENREF_32)) |
| **CD36** | rs1984112 | Fat consumption (FFQ) | 0 | 1 | ([4](#_ENREF_44)5) |
| **CD36** | rs1984112 | Ratings of perceived oiliness, fat content, and creaminess | 0 | 1 | ([4](#_ENREF_45)6) |
| **CD36** | rs1527479 | Fat consumption (FFQ) | 0 | 1 | ([4](#_ENREF_44)5) |
| **CD36** | rs1049673 | Ratings of perceived oiliness, fat content, and creaminess | 0 | 1 | ([4](#_ENREF_45)6) |
| **CD36** | rs3840546 | Ratings of perceived oiliness, fat content, and creaminess | 0 | 1 | ([4](#_ENREF_45)6) |
| **FTO** | rs1121980 | Fat intake (FFQ) | 0 | 1 | ([39](#_ENREF_39)) |
| **FTO** | rs17782313 | Fat intake (FFQ) | 0 | 1 | ([39](#_ENREF_39)) |
| **GHRL** | rs696217 | Fat intake (7-day food records) | 0 | 1 | ([28](#_ENREF_28)) |
| **LEPR** | rs1137101 | Fat intake (7-day food records) | 0 | 1 | ([28](#_ENREF_28)) |
| **LOC105375494** | rs7799039 | Fat intake (7-day food records) | 0 | 1 | ([28](#_ENREF_28)) |
| **LOC105378797** | rs2568958 | Fat intake (FFQ) | 0 | 1 | ([39](#_ENREF_39)) |
| **MTCH2** | rs10838738 | Fat intake (FFQ) | 0 | 1 | ([39](#_ENREF_39)) |
| **NA** | rs17700633 | Fat intake (FFQ) | 0 | 1 | ([39](#_ENREF_39)) |
| **NA** | rs6548238 | Fat intake (FFQ) | 0 | 1 | ([39](#_ENREF_39)) |
| **NA** | rs10938397 | Fat intake (FFQ) | 0 | 1 | ([39](#_ENREF_39)) |
| **NA** | rs368794 | Fat intake (FFQ) | 0 | 1 | ([39](#_ENREF_39)) |
| **NA** | rs7647305 | Fat intake (FFQ) | 0 | 1 | ([39](#_ENREF_39)) |
| **NA** | rs2844479 | Fat intake (FFQ) | 0 | 1 | ([39](#_ENREF_39)) |
| **OPRM1** | rs510769 | Food preference questionnaire | 0 | 1 | ([31](#_ENREF_31)) |
| **OPRM1** | rs563649 | Food preference questionnaire | 0 | 1 | ([31](#_ENREF_31)) |
| **OPRM1** | rs675026 | Food preference questionnaire | 0 | 1 | ([31](#_ENREF_31)) |
| **OPRM1** | rs9322447 | Food preference questionnaire | 0 | 1 | ([31](#_ENREF_31)) |
| **OPRM1** | rs558948 | Food preference questionnaire | 0 | 1 | ([31](#_ENREF_31)) |
| **OXTR** | rs53576 | Food preference questionnaire | 0 | 1 | ([32](#_ENREF_32)) |
| **OXTR** | rs2268493 | Food preference questionnaire | 0 | 1 | ([32](#_ENREF_32)) |
| **OXTR** | rs237885 | Food preference questionnaire | 0 | 1 | ([32](#_ENREF_32)) |
| **OXTR** | rs2254298 | Food preference questionnaire | 0 | 1 | ([32](#_ENREF_32)) |
| **OXTR** | rs2268498 | Food preference questionnaire | 0 | 1 | ([32](#_ENREF_32)) |
| **POMC** | rs3754860 | Fat intake (7-day food records) | 0 | 1 | ([28](#_ENREF_28)) |
| **POMC** | rs1009388 | Fat intake (7-day food records) | 0 | 1 | ([28](#_ENREF_28)) |
| **RGS6** | rs860195 | Fat intake (FFQ) | 0 | 1 | ([37](#_ENREF_37)) |
| **RGS6** | rs2283394 | Fat intake (FFQ) | 0 | 1 | ([37](#_ENREF_37)) |
| **RGS6** | rs847352 | Fat intake (FFQ) | 0 | 1 | ([37](#_ENREF_37)) |
| **RGS6** | rs2239250 | Fat intake (FFQ) | 0 | 1 | ([37](#_ENREF_37)) |
| **RGS6** | rs8018927 | Fat intake (FFQ) | 0 | 1 | ([37](#_ENREF_37)) |
| **RGS6** | rs7147236 | Fat intake (FFQ) | 0 | 1 | ([37](#_ENREF_37)) |
| **RGS6** | rs2238199 | Fat intake (FFQ) | 0 | 1 | ([37](#_ENREF_37)) |
| **RGS6** | rs2681749 | Fat intake (FFQ) | 0 | 1 | ([37](#_ENREF_37)) |
| **RGS6** | rs12892244 | Fat intake (FFQ) | 0 | 1 | ([37](#_ENREF_37)) |
| **RGS6** | rs847334 | Fat intake (FFQ) | 0 | 1 | ([37](#_ENREF_37)) |
| **RGS6** | rs2239227 | Fat intake (FFQ) | 0 | 1 | ([37](#_ENREF_37)) |
| **RGS6** | rs4903013 | Fat intake (FFQ) | 0 | 1 | ([37](#_ENREF_37)) |
| **RGS6** | rs10149207 | Fat intake (FFQ) | 0 | 1 | ([37](#_ENREF_37)) |
| **RGS6** | rs6574069 | Fat intake (FFQ) | 0 | 1 | ([37](#_ENREF_37)) |
| **RGS6** | rs2239223 | Fat intake (FFQ) | 0 | 1 | ([37](#_ENREF_37)) |
| **RGS6** | rs2239219 | Fat intake (FFQ) | 0 | 1 | ([37](#_ENREF_37)) |
| **RGS6** | rs10149848 | Fat intake (FFQ) | 0 | 1 | ([37](#_ENREF_37)) |
| **TAS2R38** | rs713598 | Fat intake (test meal) (children) | 0 | 1 | ([4](#_ENREF_46)7) |
| **TAS2R38** | A49P (rs713598), V296I (rs10246939) | Fat intake (3-day food record) | 0 | 1 | ([4](#_ENREF_47)8) |
| **TRPV1** | rs161364 | Preference for oiliness, fat intake (SQFFQ) | 0 | 1 | ([34](#_ENREF_34)) |
| **TRPV1** | rs8065080 | Preference for oiliness, fat intake (SQFFQ) | 0 | 1 | ([34](#_ENREF_34)) |
| **UMAMI TASTE** |  |  |  |  |  |
| **TAS1R3** | rs3813210 | Umami | 0 | 2 | ([4](#_ENREF_48)9, 50) |
| **TAS1R3** | rs35424002 | Umami | 0 | 1 | ([15](#_ENREF_15)) |
| **GNAT3** | rs6467192 | Umami | 0 | 1 | ([15](#_ENREF_15)) |

**REFERENCES**

1. Pirastu N, Kooyman M, Traglia M, Robino A, Willems SM, Pistis G, et al. A Genome-wide association study in isolated populations reveals new genes associated to common food likings. Rev Endocr Metab Disord (2016) 17:209-19. doi: 10.1007/s11154-016-9354-3.

2. Reed DR, Zhu G, Breslin PAS, et al. The perception of quinine taste intensity is associated with common genetic variants in a bitter receptor cluster on chromosome 12. Human Molecular Genetics. 2010;19(21):4278-4285

3. Robino A. (2014) Genetic variation in taste perception and its role in food liking and health status. [PhD thesis]. [Triest (Italy)]: University of Triest. Available at: https://www.openstarts.units.it/bitstream/10077/9988/1/Robino_phd.pdf (Accessed November 1, 2018)

4. Allen AL, McGeary JE, Hayes JE. Rebaudioside A and Rebaudioside D bitterness do not covary with Acesulfame K bitterness or polymorphisms in TAS2R9 and TAS2R31. Chemosens Percept (2013) 6:3. doi: 10.1007/s12078-013-9149-9.

5. Tomassini Barbarossa I, Ozdener MH, Melis M, Love-Gregory L, Mitreva M, Abumrad NA, et al. Variant in a common odorant-binding protein gene is associated with bitter sensitivity in people. Behav Brain Res (2017) 329:200-4. doi: 10.1016/j.bbr.2017.05.015.

6. Pirastu N, Kooyman M, Robino A, van der Spek A, Navarini L, Najaf A, et al. Non-additive genome-wide association scan reveals a new gene associated with habitual coffee consumption. Sci Rep (2016) 6:31590. doi: 10.1038/srep31590

7. Ledda M, Kutalik Z, Souza Destito MC, Souza MM, Cirillo CA, Zamboni A, et al. GWAS of human bitter taste perception identifies new loci and reveals additional complexity of bitter taste genetics. Hum Mol Genet (2104) 23:259-67. doi: 10.1093/hmg/ddt404.

8. Knaapila A, Hwang LD, Lysenko A, Duke FF, Fesi B, Khoshnevisan A, et al. Genetic analysis of chemosensory traits in human twins. Chem Senses (2012) 37:869-81. doi: 10.1093/chemse/bjs070.

9. Pronin AN, Xu H, Tang H, Zhang L, Li Q, Li X. Specific alleles of B

bitter receptor genes influence human sensitivity to the bitterness of aloin and saccharin.. Curr Biol (2007) 17:1403-8.

10. Rawal S, Hayes JE, Wallace MR, Bartoshuk LM, Duffy VB. Do polymorphisms in the TAS1R1 gene contribute to broader differences in human taste intensity? Chem Senses (2013) 38:719-28. doi: 10.1093/chemse/bjt040.

11. Ramos-Lopez O, Panduro A, Martinez-Lopez E, Roman S. Sweet taste receptor TAS1R2 polymorphism (Val191Val) is associated with a higher carbohydrate intake and hypertriglyceridemia among the population of West Mexico. Nutrients (2016) 8:101. doi: [10.3390/nu8020101]

12. Roudnitzky N, Behrens M,  Engel A, Kohl S, Thalmann S, Hübner S, et al. Receptor polymorphism and genomic structure interact to shape bitter taste perception. PLoS Genet (2015) 11:e1005530. doi: 10.1371/journal.pgen.1005530.

13. Campbell MC, Ranciaro A, Zinshteyn D, Rawlings-Goss R, Hirbo J, Thompson S, et al. Origin and differential selection of allelic variation at TAS2R16 associated with salicin bitter taste sensitivity in Africa. Mol Biol Evol (2014) 31:288-302. doi: 10.1093/molbev/mst211.

14. Bering AB, Pickering G, Liang P. TAS2R38 Single nucleotide polymorphisms are associated with PROP—but not thermal—tasting: a pilot study. Chem Percept (2014) 7:23-30. doi.org/10.1007/s12078-013-9160-115.

15. Risso DS, Giuliani C, Antinucci M, Morini G, Garagnani P, Tofanelli S, et al. A bio-cultural approach to the study of food choice: The contribution of taste genetics, population and culture. Appetite (2017) 114:240-47. doi: 10.1016/j.appet.2017.03.046.

16. Hayes JE, Wallace MR, Knopik VS, Herbstman DM, Bartoshuk LM, Duffy VB. Allelic variation in TAS2R bitter receptor genes associates with variation in sensations from and ingestive behaviors toward common bitter beverages in adults. Chem Senses (2011) 36:311-9. doi: 10.1093/chemse/bjq132.

17. Roudnitzky N, Bufe B, Thalmann S, Kuhn C, Gunn HC, Xing C, et al. Genomic, genetic and functional dissection of bitter taste responses to artificial sweeteners. Hum Mol Genet (2011) 20:3437-49. doi: 10.1093/hmg/ddr252.

18. Allen AL, McGeary JE, Knopik VS, Hayes JE. Bitterness of the non-nutritive sweetener acesulfame potassium varies with polymorphisms in TAS2R9 and TAS2R31. Chem Senses (2013) 38:379-89. doi: 10.1093/chemse/bjt017.

19. Pirastu N, Kooyman M, Traglia M, Robino A, Willems SM, Pistis G, et al. Association analysis of bitter receptor genes in five isolated populations identifies a significant correlation between TAS2R43 variants and coffee liking. PLoS One (2014) 9:e92065. doi.org/10.1371/journal.pone.0092065

20. Allen AL, McGeary J, Hayes JE. Polymorphisms in TRPV1 and TAS2Rs associate with sensations from sampled ethanol. Alc Clin Exp Res (2014) 38:2550-60. doi: 10.1111/acer.12527.

21. Wakai K, Matsuo K, Matsuda F, Yamada R, Takahashi M, Kawaguchi T, et al. Genome-wide association study of genetic factors related to confectionery intake: potential roles of the ADIPOQ gene. Obesity (Silver Spring, Md) (2013) 21:2413-9. doi: 10.1002/oby.20316.

22. Jablonski M, Jasiewicz A, Kucharska-Mazur J, Samochowiec J, Bienkowski P, Mierzejewski P, et al. The effect of selected polymorphisms of the dopamine receptor gene DRD2 and the ANKK-1 on the preference of concentrations of sucrose solutions in men with alcohol dependence. Psychiatr Danub (2013) 25:371-8.

23. Rudenga KJ, Small DM. Ventromedial prefrontal cortex response to concentrated sucrose reflects liking rather than sweet quality coding. Chem Senses (2013) 38:585-94. doi: 10.1093/chemse/bjt029..

24. Eny KM, Corey PN, El-Sohemy A. Dopamine D2 receptor genotype (C957T) and habitual consumption of sugars in a free-living population of men and women. J Nutrigenet Nutrigenomics (2009) 2:235-42. doi: 10.1159/000276991.

25. Søberg S, Sandholt CH, Jespersen NZ, Toft U, Madsen AL, von Holstein-Rathlou S, et al. FGF21 is a sugar-induced hormone associated with sweet intake and preference in humans. Cell Metab (2017) 25:1045-53.e6. doi: 10.1016/j.cmet.2017.04.009.

26. Fushan AA, Simons CT, Slack JP, Drayna D. Association between common variation in genes encoding sweet taste signaling components and human sucrose perception. Chem Senses (2010) 35:579-92. doi: [10.1093/chemse/bjq063]

27. Mizuta E, Kokubo Y, Yamanaka I, Miyamoto Y, Okayama A, Yoshimasa Y, et al. Leptin gene and leptin receptor gene polymorphisms are associated with sweet preference and obesity. Hypertens Res (2008) 31:1069-77. doi: 10.1291/hypres.31.1069.

28. Bienertova-Vasku J, Bienert P, Tomandl J, Forejt M, Vavrina M, Kudelkova J, et al. No association of defined variability in leptin, leptin receptor, adiponectin, proopiomelanocortin and ghrelin gene with food preferences in the Czech population. Nutr Neurosci (2008) 11:2-8. doi: 10.1179/147683008X301379.

29. Joseph PV, Reed DR, Mennella JA. Individual differences among children in sucrose detection thresholds: relationship with age, gender, and bitter taste genotype. Nurs Res (2016) 65:3-12. doi: 10.1097/NNR.0000000000000138.

30. Elbers CC, de Kovel CGF, van der Schouw YT, Meijboom JR, Bauer F, Grobbee DE, et al. Variants in neuropeptide Y receptor 1 and 5 are associated with nutrient-specific food intake and are under recent selection in Europeans. PLos One (2009) 4:e7070. doi: 10.1371/journal.pone.0007070.

31. Davis C, Zai C, Levitan RD, Kaplan AS, Carter JC, Reid-Westoby C, et al. Opiates, overeating and obesity: a psychogenetic analysis. Int J Obes (Lond) (2011) 35:1347-54. doi: 10.1038/ijo.2010.276..

32. Davis C, Patte K, Zai C, Kennedy JL. Polymorphisms of the oxytocin receptor gene and overeating: the intermediary role of endophenotypic risk factors. Nutr Diab (2017) 7:e279. doi: 10.1038/nutd.2017.24.

33. Eny KM, Wolever TM, Fontaine-Bisson B, El-Sohemy A. Genetic variant in the glucose transporter type 2 is associated with higher intakes of sugars in two distinct populations. Physiol Genomics (2008) 33:355-60. doi: 10.1152/physiolgenomics.00148.2007.

34. Park S, Zhang X, Lee NR, Jin HS. TRPV1 gene polymorphisms are associated with type 2 diabetes by their interaction with fat consumption in the Korean Genome Epidemiology Study. J Nutrigenet Nutrigenomics (2016) 9:47-61. doi: 10.1159/000446499.

35. Sasaki M, Yamada K, Namba H, Yoshinaga M, Du D, Uehara Y. Angiotensinogen gene polymorphisms and food-intake behavior in young, normal female subjects in Japan. Nutrition (2013) 29:60-5. doi: 10.1016/j.nut.2012.03.013.

36. Corella D, Arnett DK, Tsai MY, Kabagambe EK, Peacock JM, Hixson JE, et al. The -256T>C polymorphism in the apolipoprotein A-II gene promoter is associated with body mass index and food intake in the genetics of lipid lowering drugs and diet network study. Clin Chem (2007) 53:1144 –52.

37. Sibbel SP, Talbert ME, Bowden DW, Haffner SM, Taylor KD, Chen YD, et al. RGS6 variants are associated with dietary fat intake in Hispanics: the IRAS Family Study. Obesity (Silver Spring, Md) (2011) 19:1433-38. doi: 10.1038/oby.2010.333.

38. Caruso MG, Gazzerro P, Notarnicola M, Cisternino AM, Guerra V, Misciagna G, et al. Cannabinoid type 1 receptor gene polymorphism and macronutrient intake. J. Nutrigenet. Nutrigenomics (2012) 5:305-13. doi: 10.1159/000343563..

39. Bauer F, Elbers CC, Adan RA, Loos RJ, Onland-Moret NC, Grobbee DE, et al. Obesity genes identified in genome-wide association studies are associated with adiposity measures and potentially with nutrient-specific food preference. Am J Clin Nutr (2009) 90:951-9. doi: 10.3945/ajcn.2009.27781.

40. Melis M, Sollai G, Muroni P, Crnjar R, Barbarossa IT. Associations between orosensory perception of oleic acid, the common single nucleotide polymorphisms (rs1761667 and rs1527483) in the CD36 gene, and 6-n-propylthiouracil (PROP) tasting. Nutrients (2015) 7:2068-84. doi: 10.3390/nu7032068.

41. Nolden AA, McGeary JE, Hayes JE. Differential bitterness in capsaicin, piperine, and ethanol associates with polymorphisms in multiple bitter taste receptor genes. Physiol Behav (2016) 156:117-27. doi: 10.1016/j.physbeh.2016.01.017.42. Feeney EL, Hayes JE. Exploring associations between taste perception, oral anatomy and polymorphisms in the carbonic anhydrase (gustin) gene CA6. Physiol Behav (2014) 128:148-54. doi: 10.1016/j.physbeh.2014.02.013.

43. Fushan AA, Simons CT, Slack JP, Manichaikul A, Drayna D. Allelic polymorphism within the TAS1R3 promoter is associated with human taste sensitivity to sucrose. Curr Biol (2009) 19:1288-93. doi: 10.1016/j.cub.2009.06.015.

44. Bienertova-Vasku J, Bienert P, Tomandl J, Forejt M, Vasku A. Relation between adiponectin 45 T/G polymorphism and dietary composition in the Czech population. Diabetes Res Clin Pract (2009) 84:329-31. doi: 10.1016/j.diabres.2009.02.023.

45. Jayewardene AF, Mavros Y, Hancock DP, Gwinn T, Rooney KB. Associations between CD36 gene polymorphisms, fat tolerance and oral fat preference in a young-adult population. Eur J Clin Nutr (2016) 70:1325-31. doi: 10.1038/ejcn.2016.132.

46. Keller KL, Liang LC, Sakimura J, May D, van Belle C, Breen C, et al. Common variants in the CD36 gene are associated with oral fat perception, fat preferences, and obesity in African Americans. Obesity (Silver Spring) (2012) 20:1066-73. doi: 10.1038/oby.2011.374.

47. Keller KL, Olsen A, Cravener TL, Bloom R, Chung WK, Deng L, et al. Bitter taste phenotype and body weight predict children's selection of sweet and savory foods at a palatable test-meal. Appetite (2014) 77:113-21. doi: 10.1016/j.appet.2014.02.019.

48. Inoue H, Yamakawa-Kobayashi K, Suzuki Y, Nakano T, Hayashi H, Kuwano T. A case study on the association of variation of bitter-taste receptor gene TAS2R38 with the height, weight and energy intake in Japanese female college students. J Nutr Sci Vitaminol (Tokyo) (2013) 59:16-21.

49. Shigemura N, Shirosaki S, Sanematsu K, Yoshida R, Ninomiya Y. Genetic and molecular basis of individual differences in human umami taste perception. PLoS One (2009) 4:e6717. doi.org/10.1371/journal.pone.00067172.

50. Chen QY, Alarcon S, Tharp A, Ahmed OM, Estrella NL, Greene TA, et al. Perceptual variation in umami taste and polymorphisms in TAS1R taste receptor genes. Am J Clin Nutr (2009) 90:770S-9S. doi: [10.3945/ajcn.2009.27462N].
