# Supplementary material for: Genetic Background of Taste Perception, Taste Preferences, and Its Nutritional Implications: A Systematic Review
Source: Front Genet. 2019 Dec 19;10:1272. doi: 10.3389/fgene.2019.01272 (PMC6930899; doi:10.3389/fgene.2019.01272)
Supplement: Supplementary file 3 [file Table_3.docx]

**Supplementary Table 3 Study characteristics of genetic association studies related to bitter taste preferences**

SNP: single nucleotide polymorphism, PROP: 6-n-propylthiouracil, PTC: phenylthiocarbamide, GWAS: genome-wide association study, AceK: Acesulfame Potassium, FP: fungiform papillae, CP: circumvallata papillae, LMS: labeled magnitude scale, EtOH: ethanol, RebA: rebaudioside A, RebD: rebaudioside D,

**Supplementary Table 3 Study characteristics of genetic association studies related to bitter taste preferences**

| **Gene** | **SNP** | **Discovery method** | **Phenotype assessment method** | **Study population characteristics** | **Findings** | **Reference** |
| --- | --- | --- | --- | --- | --- | --- |
| **TAS2R38** | rs713598 | Candidate | Food frequency questionaire | 255 female outpatients aged 60 years or older | C allele carriers, responsible for conferring sensitivity to the PTC taste, exhibited reduced consumption of specific bitter taste foods. | (1) |
| **TAS2R38** | rs713598 | Candidate | PROP tasting | Participants (n = 202; 116 females and 86 males) were volunteers from a gastroenterology practice (Gosford, NSW) undergoing colonoscopy as a screening protocol for colonic pathology: Subjects were between 40 and 89 years of age at time of colonoscopy (overall mean age 63.2; mean age adenoma group 65.7; mean age control group 62.6). | TAS2R38 diplotype predicted bitter taste (PROP) phenotype and red cell folate status consistent with the diplotype that has the broadest range of bitter perception (AVI/PAV) also possessing the highest average red cell folate value. | (2) |
| **TAS2R38** | rs713598 | Candidate | PROP and thermal tasting | 53 participants were recruited from the students, staff, and faculty of Brock University, and comprised of 36 females and 17 males with a mean age of 26.5 years ±9.7 SD, | TAS2R38 SNPs rs713598, rs1726866, and rs10246939 associate with PROP responsiveness. | (3) |
| **TAS2R38** | rs713598 | Candidate | Goitrin, PTC, and PROP, along with a chemically unrelated compound, salicin | 50 unrelated Caucasian subjects were recruited for phenotyping and genetic analysis | Goitrin responses were associated with TAS2R38, but the same variations accounted for a smaller proportion of variance in goitrin response than for PROP and PTC. | (4) |
| **TAS2R38** | rs713598 | Candidate | PROP tasting | A sample of 599 individuals recruited in two different European countries: 299 at the Institute of Experimental Medicine, Czech Academy of Sciences, Prague, Czech Republic and 300 at the Biology Department, Pisa University, Italy. Volunteers were enrolled to obtain two groups with similar mean age (40 ± 15 years), and an equal male: female ratio. | TAS2R38 variants were associated with bitter taste sensitivity. | (5) |
| **TAS2R38** | rs713598 | Candidate | Phenylthiocarbamade tasting | Different adult samples | Variants of TAS2R38 associated with bitter taste sensitivity | (6) |
| **TAS2R38** | rs713598 | Candidate | EtOH tasting | 91 individuals of European (58 women and 35 men) with a mean age of 25 (±0.69 SEM) years. | TAS2R38 SNPs rs713598, rs1726866, and rs10246939 formed a haplotype, and were associated with bitterness. | (7) |
| **TAS2R38** | rs713598 | Candidate | PROP tasting | 108 participants (34 men) of European ancestry, with a mean age of 27.4 (±8.1 SD) years | TAS2R31 SNPs were associated with acesulfame potassium bitterness and TAS2R9 SNPs predicted additional variation in acesulfame potassium bitterness. | (8) |
| **TAS2R38** | rs713598 | Candidate | PROP tasting | 79 children aged 4-6-year-olds from diverse ethnicities. | 16 (21.1%) of the children had the GG genotype at rs713598 (bitter insensitive homozygotes), 32 (42%) were CG (bitter sensitive heterozygotes), and 28 (37%) were CC (bitter sensitive homozygotes). | (9) |
| **TAS2R38** | rs713598 | Candidate | Five food-grade bitter compounds (quinine, denatonium benzoate, caffeine, PROP, urea) with forced-choice method of paired comparisons | A racially diverse group of 154 children (3-10 years old) and their mothers (N = 118) | Bitterness of PROP was not reduced by either blocker in either age group regardless of TAS2R38 genotype. | (10) |
| **TAS2R38** | rs713598 | Candidate | Food habits questionnaire and bitter compounds tasting (salicin, PROP and stevioside) using LMS. | An overall number of 183 individuals were recruited and enrolled in the study (81 females and 102 males with an average age of 42.71 ± 15.89). Most of subjects (n=111) were Italians, with the remaining subjects coming from the Maghreb region (N=18), Sri Lanka (n=26) and Northern Europe (n=28) but recruited in Italy. | Rs713598 was associated with Broccoli score. | (11) |
| **TAS2R38** | rs713598 | Candidate | Food liking questionnaire | 215 subjects, 53.5 females, aged 10 to 76 years old with the mean age of 21.3 ± 10.4 years old. Chinese (48.9%), followed by Malays (27.5%) and Indians (22.7%). | Frequency of rs713598 non-tasters is very low among the 3 major ethnic groups of the Malaysian population, and that this SNP is not a suitable predictor of body indices and food selection for the population. | (12) |
| **TAS2R38** | rs713598 | Candidate | PTC, PROP, goitrin, methimazole, sinigrin, and salicin tasting | 56 subjects (38 female, 18 male, mean age = 41.6). | Association with bitter threshold were significant for rs713598 | (13) |
| **TAS2R38** | rs1726866 | Candidate | Phenylthiocarbamade tasting | Different adult samples | Rs1726866 was associated with bitter taste sensitivity. | (6) |
| **TAS2R38** | rs1726866 | Candidate | PROP and PTC tasting | 980 individuals, of which 448 were children (241 females, 207 males), 100 adolescents (55 females, 45 males), and 432 adults (425 females, 7 males). The majority of the adult subjects (N = 345) were the mothers of the children or adolescent participants. Children and adolescents ranged in age from 3 to 19 years (mean 7 ± 2) and adults from 20 to 55 years (mean 34 ± 7). Race/ethnicity was assigned by maternal (or adult) report according to standard US Census categories. | Human PROP sensitivity is determined by the combination of each of these 3 polymorphisms within the TAS2R38 gene. | (14) |
| **TAS2R38** | rs1726866 | Candidate | PROP tasting | Participants (n = 202; 116 females and 86 males) were volunteers from a gastroenterology practice (Gosford, NSW) undergoing colonoscopy as a screening protocol for colonic pathology: Subjects were between 40 and 89 years of age at time of colonoscopy (overall mean age 63.2; mean age adenoma group 65.7; mean age control group 62.6);. | TAS2R38 diplotype predicted bitter taste (PROP) phenotype and red cell folate status consistent with the diplotype that has the broadest range of bitter perception (AVI/PAV) also possessing the highest average red cell folate value. | (2) |
| **TAS2R38** | rs1726866 | Candidate | PROP and Acelsulfame K tasting | 108 participants (34 men) of European ancestry, with a mean age of 27.4 (±8.1 SD) years | TAS2R31 SNPs were associated with acesulfame potassium bitterness and TAS2R9 SNPs predicted additional variation in acesulfame potassium bitterness. | (8) |
| **TAS2R38** | rs1726866 | Candidate | A food liking questionnaire comprised of 45 different foods was administered for subjects to rate their liking of each item on a 9-point scale ranging from “like extremely” (score 9) to “dislike extremely” (score 1). The option “never tried” was also included. | A total of 649 subjects were recruited from six villages located in Northeast Italy, due to geographical, historical, linguistic and/or cultural factors, these villages show evidence of isolation. | PROP bitter taste perception and food liking are associated with rs1726866. | (15) |
| **TAS2R38** | rs1726866 | Candidate | PROP and thermal tasting | 53 participants were recruited from the students, staff, and faculty of Brock University, and comprised of 36 females and 17 males with a mean age of 26.5 years ±9.7 SD, | TAS2R38 SNPs rs713598, rs1726866, and rs10246939 associate with PROP responsiveness. | (3) |
| **TAS2R38** | rs1726866 | Candidate | Goitrin, PTC, and PROP, along with a chemically unrelated compound, salicin | 50 unrelated Caucasian subjects were recruited for phenotyping and genetic analysis | Goitrin responses were associated with TAS2R38, but the same variations accounted for a smaller proportion of variance in goitrin response than for PROP and PTC. | (4) |
| **TAS2R38** | rs1726866 | Candidate | PROP tasting | A sample of 599 individuals recruited in two different European countries: 299 at the Institute of Experimental Medicine, Czech Academy of Sciences, Prague, Czech Republic and 300 at the Biology Department, Pisa University, Italy. Volunteers were enrolled to obtain two groups with similar mean age (40 ± 15 years), and an equal male: female ratio. | TAS2R38 variants were associated with bitter taste sensitivity. | (5) |
| **TAS2R38** | rs1726866 | Candidate | Food habits questionnaire and bitter compounds tasting (salicin, PROP and stevioside) using LMS. | An overall number of 183 individuals were recruited and enrolled in the study (81 females and 102 males with an average age of 42.71 ± 15.89). Most of subjects (n=111) were Italians, with the remaining subjects coming from the Maghreb region (N=18), Sri Lanka (n=26) and Northern Europe (n=28) but recruited in Italy. | Rs1726866 was associated with Broccoli score. | (11) |
| **TAS2R38** | rs1726866 | Candidate | PTC, PROP, goitrin, methimazole, sinigrin, and salicin tasting | 56 subjects (38 female, 18 male, mean age = 41.6). | Association with bitter threshold was significant for rs1726866. | (13) |
| **TAS2R38** | rs10246939 | Candidate | PROP and PTC tasting | 980 individuals, of which 448 were children (241 females, 207 males), 100 adolescents (55 females, 45 males), and 432 adults (425 females, 7 males). The majority of the adult subjects (N = 345) were the mothers of the children or adolescent participants. Children and adolescents ranged in age from 3 to 19 years (mean 7 ± 2) and adults from 20 to 55 years (mean 34 ± 7). Race/ethnicity was assigned by maternal (or adult) report according to standard US Census categories. | Human PROP sensitivity is determined by the combination of each of these 3 polymorphisms within the TAS2R38 gene. | (14) |
| **TAS2R38** | rs10246939 | Candidate | PROP tasting | Participants (n = 202; 116 females and 86 males) were volunteers from a gastroenterology practice (Gosford, NSW) undergoing colonoscopy as a screening protocol for colonic pathology: Subjects were between 40 and 89 years of age at time of colonoscopy (overall mean age 63.2; mean age adenoma group 65.7; mean age control group 62.6);. | TAS2R38 diplotype predicted bitter taste (PROP) phenotype and red cell folate status consistent with the diplotype that has the broadest range of bitter perception (AVI/PAV) also possessing the highest average red cell folate value. | (2) |
| **TAS2R38** | rs10246939 | Candidate | EtOH tasting | 91 individuals of European (58 women and 35 men) with a mean age of 25 (±0.69 SEM) years. | TAS2R38 SNPs rs713598, rs1726866, and rs10246939 formed a haplotype, and were associated with bitterness. | (7) |
| **TAS2R38** | rs10246939 | Candidate | PROP and thermal tasting | 53 participants were recruited from the students, staff, and faculty of Brock University, and comprised of 36 females and 17 males with a mean age of 26.5 years ±9.7 SD, | TAS2R38 SNPs rs713598, rs1726866, and rs10246939 associate with PROP responsiveness. | (3) |
| **TAS2R38** | rs10246939 | Candidate | PROP tasting | A sample of 599 individuals recruited in two different European countries: 299 at the Institute of Experimental Medicine, Czech Academy of Sciences, Prague, Czech Republic and 300 at the Biology Department, Pisa University, Italy. Volunteers were enrolled to obtain two groups with similar mean age (40 ± 15 years), and an equal male: female ratio. | Rs10246939 was associated with bitter taste sensitivity. | (5) |
| **TAS2R38** | rs10246939 | GWAS | Individual taste detection thresholds were determined for 13 tested compounds with staircase method. | 607 healthy adult (503 subjects for the discovery panel and 104 for the replication panel) subjects between 18 and 45 years old recruited from the general population of Sao Paulo in Brazil. | Rs10246939 influences bitter taste sensitivity. | (16) |
| **TAS2R38** | rs10246939 | Candidate | Food habits questionnaire and bitter compounds tasting (salicin, PROP and stevioside) using LMS. | An overall number of 183 individuals were recruited and enrolled in the study (81 females and 102 males with an average age of 42.71 ± 15.89). Most of subjects (n=111) were Italians, with the remaining subjects coming from the Maghreb region (N=18), Sri Lanka (n=26) and Northern Europe (n=28) but recruited in Italy. | Rs10246939 was associated with Broccoli score. | (11) |
| **TAS2R38** | rs10246939 | Candidate | PTC, PROP, goitrin, methimazole, sinigrin, and salicin tasting | 56 subjects (38 female, 18 male, mean age = 41.6). | Association with bitter threshold was significant for rs10246939. | (13) |
| **TAS2R38** | rs713598 (A49P) | Candidate | PROP and thermal tasting | 53 participants were recruited from the students, staff, and faculty of Brock University, and comprised of 36 females and 17 males with a mean age of 26.5 years ±9.7 SD, | TAS2R38 SNPs rs713598, rs1726866, and rs10246939 associate with PROP responsiveness. | (3) |
| **TAS2R38** | rs713598 (A49P)  rs1726866 (A262V) | Candidate | PROP tasting | 4795 children | Variants in TAS2R38 are associated with intermediate biter tasting ability. | (17) |
| **TAS2R38** | rs713598 (A49P)  rs1726866 (A262V) | Candidate | Cruciferous/Brassica vegetable intake (24-hour dietary recall) | 634 volunteers recruited (1992-1998) from the Italian arm of the EPIC study. | Haplotype associated with cruciferous vegetable intake. | (18) |
| **TAS2R38** | rs713598 (A49P)  rs1726866 (A262V)  rs10246939  (V296I) | Candidate | Phenylthiocarbamade tasting | Different adult samples | Rs1726866 was associated with bitter taste sensitivity. | (6) |
| **TAS2R38** | rs713598 (A49P)  rs1726866 (A262V)  rs10246939  (V296I) | Candidate | PROP tasting | 63 volunteers (21 males, 42 females, age 25±3 y) | PROP super-tastings was strongly associated with the ‘taster’ variant (PAV haplotype) of TAS2R38. | (19) |
| **TAS2R38** | rs713598 (A49P)  rs1726866 (A262V)  rs10246939  (V296I) | Candidate | PROP tasting | A sample of 198, recruited via posters and word of mouth for a laboratory study of the relationship between variation in oral sensation and dietary behaviors. | Variability in the receptor expression may explain attenuated bitterness– FP relationships. PROP bitterness does associate with heightened taste sensations (i.e., supertasting), but this is not due to TAS2R38 polymorphisms. | (20) |
| **TAS2R38** | rs713598 (A49P)  rs1726866 (A262V)  rs10246939  (V296I) | Candidate | PROP tasting | Eighty-four subjects (53 women and 31 men) with an age (mean SD) of 36 13 years (range, 21–59 years). | Taste genetic effects on alcohol intake and PROP bitterness serves as a marker of these effects. | (21) |
| **TAS2R38** | rs713598 (A49P)  rs1726866 (A262V)  rs10246939  (V296I) | Candidate | Food liking scale and 3-dietary reacord | 7–13 year old Irish children (n = 525). | PROP ratings were correlated but did not affect cruciferous vegetable liking or intake. | (22) |
| **TAS2R38** | rs713598 (A49P)  rs1726866 (A262V)  rs10246939  (V296I) | Candidate | PROP tasting | Participants (n = 202; 116 females and 86 males) were volunteers from a gastroenterology practice (Gosford, NSW) undergoing colonoscopy as a screening protocol for colonic pathology: Subjects were between 40 and 89 years of age at time of colonoscopy (overall mean age 63.2; mean age adenoma group 65.7; mean age control group 62.6);. | TAS2R38 diplotype predicted bitter taste (PROP) phenotype and red cell folate status consistent with the diplotype that has the broadest range of bitter perception (AVI/PAV) also possessing the highest average red cell folate value. | (2) |
| **TAS2R38** | rs713598 (A49P)  rs1726866 (A262V)  rs10246939  (V296I) | Candidate | PROP and thermal tasting | 53 participants were recruited from the students, staff, and faculty of Brock University, and comprised of 36 females and 17 males with a mean age of 26.5 years ±9.7 SD, | TAS2R38 SNPs rs713598, rs1726866, and rs10246939 associate with PROP responsiveness. | (3) |
| **TAS2R38** | rs713598 (A49P)  rs1726866 (A262V)  rs10246939  (V296I) | Candidate | PROP tasting | 76 participants (29 males, 47 females, age 25±3 y) Caucasians from Italy. | Variation in body weight larger than 5 kg over the previous 3 months was asociated with selected SNPs. | (23) |
| **TAS2R38** | rs713598 (A49P)  rs1726866 (A262V)  rs10246939  (V296I) | Candidate | Suprathreshold method with 6-propyl-2-thiouracil | A total of 320 individuals: -120 healthy adult volunteers (medical students) - 44 healthy children - 54 children affected by a variety of functional disturbances - 92 mother–child dyads, including 41 of the 44 healthy and 51 of the 54 affected children - 44 unselected healthy children, with their mothers, were recruited consecutively at a well-baby clinic in the field; 54 children affected by a variety of disturbances were recruited from outpatients in the Department of Pediatrics | Greater sensitivity to 6-propyl-2-thiouracil predicts lower preferences for vegetables in children, showing an appreciable effect of the genetic predisposition on food choices, but in the present study it was not clear. | (24) |
| **TAS2R38** | rs713598 (A49P)  rs1726866 (A262V)  rs10246939  (V296I) | Candidate | PROP tasting | 63 non-smoking Caucasian healthy, young subjects (22 males, 42 females, age 25 ± 3 y) from Sardinia, Italy were recruited at the local University. They had a normal body mass index (BMI) ranging from 18.6 to 25.3 kg/m2 and showed no variation of body weight larger than 5 kg over the previous 3 months. | Associated with PROP sensitivity | (25) |
| **TAS2R38** | rs713598 (A49P)  rs1726866 (A262V)  rs10246939  (V296I) | Candidate | PTC tasting | 611 individuals originating from 57 populations in Africa that practice diverse modes of subsistence and a comparative set of 132 non-Africans from the Middle East, Europe, South Asia, East Asia, and the Americas595 individuals from 74 African populations and in 94 non-Africans from 11 populations | There is a genotype–phenotype association variation at other TAS2R bitter taste loci in diverse populations. Polymorphism at TAS2R16 influences salicin recognition. | (26) |
| **TAS2R38** | rs713598 (A49P)  rs1726866 (A262V)  rs10246939  (V296I) | Candidate | Food habits questionnaire and bitter compounds tasting (salicin, PROP and stevioside) using LMS. | An overall number of 183 individuals were recruited and enrolled in the study (81 females and 102 males with an average age of 42.71 ± 15.89). Most of subjects (n=111) were Italians, with the remaining subjects coming from the Maghreb region (N=18), Sri Lanka (n=26) and Northern Europe (n=28) but recruited in Italy. | Rs10246939 was associated with Broccoli score. | (11) |
| **TAS2R38** | rs713598 (A49P)  rs1726866 (A262V)  rs10246939  (V296I) | Candidate | Goitrin, PTC, and PROP, along with a chemically unrelated compound, salicin | 50 unrelated Caucasian subjects were recruited for phenotyping and genetic analysis | Goitrin responses were associated with TAS2R38, but the same variations accounted for a smaller proportion of variance in goitrin response than for PROP and PTC. | (4) |
| **TAS2R38** | rs713598 (A49P)  rs1726866 (A262V)  rs10246939  (V296I) | Candidate | PROP tasting and Food Frequency Questionnaire (59 items) | 393 individuals (212 males, 181 females) between 19–55 years of age were selected as a convenience sample from 4 geographical regions of India. | Food preferences did not significantly (p > 0.05) correlate with TAS2R38 diplotypes or PROP phenotypes. | (27) |
| **TAS2R38** | rs713598 (A49P)  rs1726866 (A262V)  rs10246939  (V296I) | Candidate | PROP tasting and Food Preferences and Mothers’ Perception of Child Temperament. | A racially and ethnically diverse sample of 143 children and their mothers. | Variations in a taste receptor gene accounted for a major portion of individual differences in PROP bitterness perception in both children and adults. | (28) |
| **TAS2R38** | rs713598 (A49P)  rs1726866 (A262V)  rs10246939  (V296I) | Candidate | PROP tasting | 64 non-smoking Caucasian subjects (23 males, 41 females, age 27.6 ± 0.85 years) from Sardinia, Italy were recruited according to standard procedures. | Direct association between orosensory perception of oleic acid and PROP tasting or rs1761667 polymorphism of CD36. | (29) |
| **TAS2R38** | rs713598 (A49P)  rs1726866 (A262V)  rs10246939  (V296I) | Candidate | Quinine hydrochloride and PROP tasting. Vegetable intake was assessed using two complementary but distinct methods: (a) five 1-day, non-consecutive food records, and (b) validated food frequency questionnaire | 59 reportedly healthy individuals (28 females, 31 males; mean=26.14±0.55 SEM) for whom blood samples were available for genotyping the TAS2R38 gene. | Genetic variation in taste, measured by multiple phenotypes or TAS2R38 genotype, can explain differences in overall consumption of vegetables, and this was not restricted to vegetables that are predominantly bitter | (30) |
| **TAS2R38** | rs713598 (A49P)  rs1726866 (A262V)  rs10246939  (V296I) | Candidate | PROP and PTC tasting | 94 healthy, non-smoking participants | Sensitivity to these bitter chemicals are partially controlled by variation in the TAS2R38 gene. | (31) |
| **TAS2R38** | rs713598 (A49P)  rs1726866 (A262V)  rs10246939  (V296I) | Candidate | PROP tasting and 79-item food liking questionnaire | 496 subjects participated in the study (206 males and 290 females), coming from 20 different communities of six countries | Population-based approach utilizing distance matrices is a useful technique for detecting PROP-related differences in food liking and can be applied to other taste phenotypes. | (32) |
| **TAS2R38** | rs713598 (A49P)  rs1726866 (A262V)  rs10246939  (V296I) | Candidate | PROP and PTC tasting | Healthy (self-reported) males (n=19) and females (n=16). The age range of subjects was 18-66 y in PAV/PAV, 19-61 in AVI/AVI, and 25-59 y in PAV/AVI group. | Genotypes of hTAS2R38 specifically determine humans' bitterness perception of plants that synthesize glucosinolates, a class of anti-thyroid compounds that also contain the thiourea moiet | (33) |
| **TAS2R38** | rs713598 (A49P)  rs1726866 (A262V)  rs10246939  (V296I) | Candidate | Capsaicin, piperine, and ethanol. | 106 participants (40 men) with a mean age of 25.2 ± 0.63 (SEM). Caucasian ancestry (n = 69), followed by Asian (n = 15) and African American (n = 1); 11 participants did not disclose their ancestry. | For TAS2R38, PAV homozygotes perceived greater bitterness from capsaicin and ethanol, compared to heterozygotes and AVI homozygotes. | (34) |
| **TAS2R38** | rs713598 (A49P)  rs1726866 (A262V)  rs10246939  (V296I) | Candidate | Eruca sativa (“salad rocket”) | 91 consenting individuals, who were recruited from in and around the University of Reading, over 18 years of age and non-smokers. | Bitter perception did not significantly influence liking of accessions, despite PAV/PAV ‘supertasters’ scoring higher for this attribute. | (35) |
| **TAS2R38** | rs713598 (A49P)  rs1726866 (A262V)  rs10246939  (V296I) | Candidate | PTC, PROP, goitrin, methimazole, sinigrin, and salicin tasting | 56 subjects (38 female, 18 male, mean age = 41.6). | Association with bitter threshold was significant for rs10246939. | (13) |
| **TAS2R38** | rs713598 (A49P)  rs1726866 (A262V)  rs10246939  (V296I) | Candidate | Wild bilberries juice tasting and Food Choice Questionnaire | 41 voluntary subjects (32 females and 9 males) between ages 20 and 60 (low 20–30, n = 13; medium 31–40, n = 16; high 41–60, n = 12). | hTAS2R38 PAV homozygotes liked berry extracts less than AVI homozygotes. | (36) |
| **TAS2R38** | rs713598 (A49P)  rs1726866 (A262V)  rs10246939  (V296I) | Candidate | Brassica vegetable intake (FFQ) | 7–13 year old Irish children (n = 525) | Associated with consumption of bitter tasting vegetables intake (only in children | (37) |
| **TAS2R19** | rs10772420 | Candidate | Quinine hydrochloride and PTC tasting | 502 monozygotic (251 not genetically identical) and 70 dyzigotic (35 not genetically identical) twin pairs. | Associated with quinine ratings. | (38) |
| **TAS2R19** | rs10772420 | Candidate | Quinine tasting | 246 subjects of which 99 men, aged 18–45 years. | Associated with quinine tasting. | (39) |
| **TAS2R19** | rs10772420 | GWAS | Quinine HCl, caffeine, sucrose octaacetate and Propylthiouracil | For the GWAS study 1457 subjects (671 males and 786 females) were recruited, aged 11-25 years old, from Australia. The replication study included 73 subjects from USA (16 males and 57 females) aged 21 – 82 years old. | Associated with quinine tasting. | (40) |
| **TAS2R19** | rs10772420 | Candidate | Absinthin, amarogentin, cascarillin, grosheimin, quassin, and quinine tasting. | 48 unrelated Caucasian subjects: 39 women, 9 men; age range 21-59 years, mean age = 30.6 years. | Complex associations dependent on linkage between several high- and low-sensitivity alleles. | (41) |
| **TAS2R19** | rs10772420 | Candidate | Instant espresso tasting and intake frequency of bitter food items. | A sample of 198, recruited via posters and word of mouth for a laboratory study of the relationship between variation in oral sensation and dietary behaviors. | SNP appears to influence the sensations, liking, or intake of common bitter-tasting beverages | (42) |
| **TAS2R19** | rs1868769 | Candidate | Quinine hydrochloride and PTC tasting | 502 monozygotic (251 not genetically identical) and 70 dyzigotic (35 not genetically identical) twin pairs. | Associated with quinine ratings. | (38) |
| **TAS2R19** | rs10772420 | Candidate | Absinthin, amarogentin, cascarillin, grosheimin, quassin, and quinine tasting. | 48 unrelated Caucasian subjects: 39 women, 9 men; age range 21-59 years, mean age = 30.6 years. | Complex associations dependent on linkage between several high- and low-sensitivity alleles. | (41) |
| **TAS2R31 (formerly known as TAS2R44)** | rs10845293 | Candidate | Absinthin, amarogentin, cascarillin, grosheimin, quassin, and quinine tasting. | 48 unrelated Caucasian subjects: 39 women, 9 men; age range 21-59 years, mean age = 30.6 years. | Complex associations dependent on linkage between several high- and low-sensitivity alleles. | (41) |
| **TAS2R31 (formerly known as TAS2R44)** | rs10845293 | Candidate | Bitter perception of saccharin and acesulfame K | 60 unrelated caucasians: 41 women, 19 men; age range 20–62 years, mean age 32.3 years, SD = 11.4. | Associated with sacharin and acesulfame K tasting. | (43) |
| **TAS2R31 (formerly known as TAS2R44)** | rs10845293 | Candiadte | PTC, aloin and and sacharin tasting | 55 unrelated volunteers (20–51 year old) of mostly Caucasian and Asian origin. | Associated with response to compounds. | (44) |
| **TAS2R31 (formerely known as TAS2R44)** | rs10845293 | Candidate | AceK, RebA and RebD. | 122 participants (44 men), with a mean age 27.7±7.89 years | Explains AceK Bitterness but not Bitterness of RebA and RebD. | (45) |
| **TAS2R31 (formerly known as TAS2R44)** | rs10845293 | Candidate | PROP tasting and Acesulfame K | 108 participants (34 men) of European ancestry, with a mean age of 27.4 (±8.1 SD) years | Associated with bitterness. | (8) |
| **TAS2R31 (formerly known as TAS2R44)** | rs10845293 | Candidate | Quinine tasting | 246 subjects of which 99 men, aged 18–45 years. | Associated with quinine tasting. | (39) |
| **TAS2R31 (formerly known as TAS2R44)** | rs10772423 | Candidate | Absinthin, amarogentin, cascarillin, grosheimin, quassin, and quinine tasting. | 48 unrelated Caucasian subjects: 39 women, 9 men; age range 21-59 years, mean age = 30.6 years. | Complex associations dependent on linkage between several high- and low-sensitivity alleles. | (41) |
| **TAS2R31 (formerly known as TAS2R44)** | rs10772423 | Candidate | AceK, RebA and RebD. | 122 participants (44 men), with a mean age 27.7±7.89 years | Explains AceK Bitterness but not Bitterness of RebA and RebD. | (45) |
| **TAS2R31 (formerly known as TAS2R44)** | rs10772423 | Candidate | Quinine tasting | 246 subjects of which 99 men, aged 18–45 years. | Associated with quinine tasting. | (39) |
| **TAS2R4** | rs22304001 | Candidate | Food habits questionnaire and bitter compounds tasting (salicin, PROP and stevioside) using LMS. | An overall number of 183 individuals were recruited and enrolled in the study (81 females and 102 males with an average age of 42.71 ± 15.89). Most of subjects (n=111) were Italians, with the remaining subjects coming from the Maghreb region (N=18), Sri Lanka (n=26) and Northern Europe (n=28) but recruited in Italy. | Associated with Broccoli score. | (11) |
| **TAS2R4** | rs22304001 | Candidate | Bitter perception of saccharin and acesulfame K | 60 unrelated caucasians: 41 women, 19 men; age range 20–62 years, mean age 32.3 years, SD = 11.4. | Associated with sacharin and acesulfame K tasting. | (43) |
| **TAS2R5** | rs2227264 | Candidate | Instant espresso tasting and intake frequency of bitter food items. | A sample of 198, recruited via posters and word of mouth for a laboratory study of the relationship between variation in oral sensation and dietary behaviors. | SNP appears to influence the sensations, liking, or intake of common bitter-tasting beverages | (42) |
| **TAS2R5** | rs2227264 | Candidate | PROP tasting | A sample of 599 individuals recruited in two different European countries: 299 at the Institute of Experimental Medicine, Czech Academy of Sciences, Prague, Czech Republic and 300 at the Biology Department, Pisa University, Italy. Volunteers were enrolled to obtain two groups with similar mean age (40 ± 15 years), and an equal male: female ratio. | Associated with bitter taste sensitivity. | (5) |
| **TAS2R5** | rs2234012 | Candidate | Instant espresso tasting and intake frequency of bitter food items. | A sample of 198, recruited via posters and word of mouth for a laboratory study of the relationship between variation in oral sensation and dietary behaviors. | SNP appears to influence the sensations, liking, or intake of common bitter-tasting beverages | (42) |
| **TAS2R5** | rs2234012 | Candidate | Capsaicin, piperine, and ethanol. | 106 participants (40 men) with a mean age of 25.2 ± 0.63 (SEM). Caucasian ancestry (n = 69), followed by Asian (n = 15) and African American (n = 1); 11 participants did not disclose their ancestry. | CCCAGT homozygotes rated the greatest bitterness, compared to heterozygotes and TTGGAG homozygotes, for both ethanol and capsaicin. | (34) |
| **TAS2R9** | rs3741845 | Candidate | AceK, RebA and RebD. | 122 participants (44 men), with a mean age 27.7±7.89 years | Explains AceK Bitterness but not Bitterness of RebA and RebD. | (45) |
| **TAS2R9** | rs3741845 | Candidate | PROP tasting and Acesulfame K | 108 participants (34 men) of European ancestry, with a mean age of 27.4 (±8.1 SD) years | Associated with bitterness. | (8) |
| **CA6** | rs2274333 | Candidate | PROP tasting | 75 volunteers (28 men and 47 women; mean plusmn SEM age: 25 plusmn 3 y) | Associated with PROP sensitivity. | (46) |
| **CA6** | rs2274333 | Candidate | PROP tasting | 63 non-smoking Caucasian healthy, young subjects (22 males, 42 females, age 25 ± 3 y) from Sardinia, Italy were recruited at the local University. They had a normal body mass index (BMI) ranging from 18.6 to 25.3 kg/m2 and showed no variation of body weight larger than 5 kg over the previous 3 months. | A gustin (CA6) gene polymorphism, affects PROP tasting by acting on the density and maintenance of fungiform papillae, and that between the two protein iso-forms that result from this polymorphism, gustin 90Ser exhibits full functional activity, compared to the gustin 90Gly iso-form. | (25) |
| **CA6** | rs2274333 | Candidate | PROP tasting | 76 participants (29 males, 47 females, age 25±3 y) Caucasians from Italy. | Variation in body weight larger than 5 kg over the previous 3 months was asociated. | (23) |
| **CA6** | rs2274333 | Candidate | PROP tasting | 63 volunteers (21 males, 42 females, age 25±3 y) | PROP super-tastings was strongly associated. | (19) |

| **DFNA5** | rs73082019 | GWAS | Food preference questionnaire | 4611 individuals | Association with preference of dark chocolate. | (47) |
| --- | --- | --- | --- | --- | --- | --- |
| **DIRC3-AS1** | rs4141835 | GWAS | PROP intensity measurement | For the discovery sample, participants were a subset of adolescent and young adult twins and their singleton siblings who have participated in previous studies of the genetics of skin moles and cognition. The sample for which taste sensitivity results were available consisted of females and males and included monozygotic (MZ) and dizygotic (DZ) twin pairs and their siblings. For the replication sample, experimenters recruited and tested participants at an annual convention of twins, Twins Days Festival, in Twinsburg, OH, USA. Testing occurred at the 2009 festival in August. Characteristic: Discovery: n=1457, males/females 671/786, age (mean + standard deviation): 18 + 2, age range (years) 11–25; Replication: n=73,  males/females 16/57,  age (mean + standard deviation) 42 + 17,  age range (years): 21–82. | Associated with PROP intensity ratings. | (48) |
| **LINC02346** | rs8034691 | GWAS | Food preference questionnaire | 4611 individuals | Associated with artichokes liking. | (47) |
| **LOC105370401** | rs10137305 | GWAS | PROP intensity measurement | This study includes 3500 participants coming from three different Italian populations. Due to geographical, historical, linguistic and/or cultural factors, these populations showed evidences of genetic isolation. Also this study included total of 496 subjects participated in the study (206 males and 290 females), coming from 20 different communities of six countries in the Caucasus and Central Asia: Georgia, Armenia, Azerbaijan, Uzbekistan, Kazakhstan and | Associated with PROP intensity ratings. | (49) |
| **LOC105370401** | rs11623995 | GWAS | PROP intensity measurement | This study includes 3500 participants coming from three different Italian populations. Due to geographical, historical, linguistic and/or cultural factors, these populations showed evidences of genetic isolation. Also this study included total of 496 subjects participated in the study (206 males and 290 females), coming from 20 different communities of six countries in the Caucasus and Central Asia: Georgia, Armenia, Azerbaijan, Uzbekistan, Kazakhstan and | Associated with PROP intensity ratings. | (49) |
| **LOC105370401** | rs2331619 | GWAS | PROP intensity measurement | This study includes 3500 participants coming from three different Italian populations. Due to geographical, historical, linguistic and/or cultural factors, these populations showed evidences of genetic isolation. Also this study included total of 496 subjects participated in the study (206 males and 290 females), coming from 20 different communities of six countries in the Caucasus and Central Asia: Georgia, Armenia, Azerbaijan, Uzbekistan, Kazakhstan and | Associated with PROP intensity ratings. | (49) |
| **LOC105370401** | rs7144549 | GWAS | PROP intensity measurement | This study includes 3500 participants coming from three different Italian populations. Due to geographical, historical, linguistic and/or cultural factors, these populations showed evidences of genetic isolation. Also this study included total of 496 subjects participated in the study (206 males and 290 females), coming from 20 different communities of six countries in the Caucasus and Central Asia: Georgia, Armenia, Azerbaijan, Uzbekistan, Kazakhstan and | Associated with PROP intensity ratings. | (49) |
| **LOC105377448** | rs28849980 | GWAS | Food preference questionnaire | 4611 individuals | Associated with artichokes liking. | (47) |
| **NA** | rs10050951 | GWAS | Food preference questionnaire | 4611 individuals | Associated with artichokes liking. | (47) |
| **NA** | rs12200968 |  | PROP intensity measurement | This study includes 3500 participants coming from three different Italian populations. Due to geographical, historical, linguistic and/or cultural factors, these populations showed evidences of genetic isolation. Also this study included total of 496 subjects participated in the study (206 males and 290 females), coming from 20 different communities of six countries in the Caucasus and Central Asia: Georgia, Armenia, Azerbaijan, Uzbekistan, Kazakhstan and | Associated with PROP intensity ratings. | (49) |
| **NA** | rs138369603 | GWAS | Food preference questionnaire | 4611 individuals | Associated with chicory liking. | (47) |
| **NA** | rs145671205 | GWAS | Food preference questionnaire | 4611 individuals | Associated with coffee liking. | (47) |
| **NA** | rs2530184 | GWAS | Food preference questionnaire | 4611 individuals | Associated with broccoli liking. | (47) |
| **NA** | rs374184 | Candidate | Bitterness of Acesulfame Potassium | Intensity ratings (test samples: sucrose, gentiobiose, aspartame, rebaudioside A and D). Participants reported perceived intensity of the test stimuli by rating on a general Labeled Magnitude Scale (gLMS). The gLMS anchors are 0 (‘no sensation’) to 100 (’the strongest imaginable sensation of any kind’), with descriptors at 1.4 (‘barely detectable’), 6 (‘weak’), 17 (‘moderate’), 35 (‘strong’) and 51 (‘very strong’). | Val carriers perceived significantly more bitterness than Ala homozygotes. | (45) |
| **NA** | rs6458845 | GWAS | PROP intensity measurement | This study includes 3500 participants coming from three different Italian populations. Due to geographical, historical, linguistic and/or cultural factors, these populations showed evidences of genetic isolation. Also this study included total of 496 subjects participated in the study (206 males and 290 females), coming from 20 different communities of six countries in the Caucasus and Central Asia: Georgia, Armenia, Azerbaijan, Uzbekistan, Kazakhstan and | Associated with PROP intensity ratings. | (49) |
| **NA** | rs7746307 | GWAS | PROP intensity measurement | This study includes 3500 participants coming from three different Italian populations. Due to geographical, historical, linguistic and/or cultural factors, these populations showed evidences of genetic isolation. Also this study included total of 496 subjects participated in the study (206 males and 290 females), coming from 20 different communities of six countries in the Caucasus and Central Asia: Georgia, Armenia, Azerbaijan, Uzbekistan, Kazakhstan and | Associated with PROP intensity ratings. | (49) |
| **NA** | rs9832668 |  | Food preference questionnaire | 4611 individuals, | Associated with broccoli liking (GWAS). | (47) |
| **OBP2A** | rs2590498 | Candiddate | PROP bitterness | Ninety-six non-smoking, Caucasian healthy, young subjects (27.2 (SD 6.5) years of age, 22.0 (SD 2.3) kg/m^2^, 55 females) from Sardinia, Italy | AA homozygotes perceived PROP as bitterer than the GG subjects. | (50) |
| **PDSS2** | rs2216084 | GWAS | Coffee consumption | Samples from two isolated Italian populations participating to the INGI consortium were included. In particular the inidviduals involved were 370 individuals from INGI-CARL, a population coming from Carlantino, a small village located in Puglia (Southern Italy), and 843 defined as INGI-FVG, making reference to 6 villages situated in the Friuli Venezia Region in North-Eastern Italy for a total of 1207 samples. For replication the samples coming from the Erasmus Rucphen Family (ERF) study were used, a cross-sectional cohort including 3,000 living descendants of 22 couples who had at least 6 children baptized in the community church around 1850–1900; 1731 samples were used from this study. | Associated with coffee consumption. | (51) |
| **PDSS2** | rs6568479 | GWAS | Coffee consumption | Samples from two isolated Italian populations participating to the INGI consortium were included. In particular the inidviduals involved were 370 individuals from INGI-CARL, a population coming from Carlantino, a small village located in Puglia (Southern Italy), and 843 defined as INGI-FVG, making reference to 6 villages situated in the Friuli Venezia Region in North-Eastern Italy for a total of 1207 samples. For replication the samples coming from the Erasmus Rucphen Family (ERF) study were used, a cross-sectional cohort including 3,000 living descendants of 22 couples who had at least 6 children baptized in the community church around 1850–1900; 1731 samples were used from this study. | Associated with coffee consumption. | (51) |
| **PDSS2** | rs6942255 | GWAS | Coffee consumption | Samples from two isolated Italian populations participating to the INGI consortium were included. In particular the inidviduals involved were 370 individuals from INGI-CARL, a population coming from Carlantino, a small village located in Puglia (Southern Italy), and 843 defined as INGI-FVG, making reference to 6 villages situated in the Friuli Venezia Region in North-Eastern Italy for a total of 1207 samples. For replication the samples coming from the Erasmus Rucphen Family (ERF) study were used, a cross-sectional cohort including 3,000 living descendants of 22 couples who had at least 6 children baptized in the community church around 1850–1900; 1731 samples were used from this study. | Associated with coffee consumption. | (51) |
| **PDSS2** | rs7745311 | GWAS | Coffee consumption | Samples from two isolated Italian populations participating to the INGI consortium were included. In particular the inidviduals involved were 370 individuals from INGI-CARL, a population coming from Carlantino, a small village located in Puglia (Southern Italy), and 843 defined as INGI-FVG, making reference to 6 villages situated in the Friuli Venezia Region in North-Eastern Italy for a total of 1207 samples. For replication the samples coming from the Erasmus Rucphen Family (ERF) study were used, a cross-sectional cohort including 3,000 living descendants of 22 couples who had at least 6 children baptized in the community church around 1850–1900; 1731 samples were used from this study. | Associated with coffee consumption. | (51) |
| **PDSS2** | rs7754744 | GWAS | Coffee consumption | Samples from two isolated Italian populations participating to the INGI consortium were included. In particular the inidviduals involved were 370 individuals from INGI-CARL, a population coming from Carlantino, a small village located in Puglia (Southern Italy), and 843 defined as INGI-FVG, making reference to 6 villages situated in the Friuli Venezia Region in North-Eastern Italy for a total of 1207 samples. For replication the samples coming from the Erasmus Rucphen Family (ERF) study were used, a cross-sectional cohort including 3,000 living descendants of 22 couples who had at least 6 children baptized in the community church around 1850–1900; 1731 samples were used from this study. | Associated with coffee consumption. | (51) |
| **PDSS2** | rs9386630 | GWAS | Coffee consumption | Samples from two isolated Italian populations participating to the INGI consortium were included. In particular the inidviduals involved were 370 individuals from INGI-CARL, a population coming from Carlantino, a small village located in Puglia (Southern Italy), and 843 defined as INGI-FVG, making reference to 6 villages situated in the Friuli Venezia Region in North-Eastern Italy for a total of 1207 samples. For replication the samples coming from the Erasmus Rucphen Family (ERF) study were used, a cross-sectional cohort including 3,000 living descendants of 22 couples who had at least 6 children baptized in the community church around 1850–1900; 1731 samples were used from this study. | Associated with coffee consumption. | (51) |
| **PRH1-TAS2R14** | rs1031391 | GWAS | Quinine | 607 healthy adult (503 subjects for the discovery panel and 104 for the replication panel) subjects between 18 and 45 years old recruited from the general population of Sao Paulo in Brazil. | Associated with the perceived intensity of suprathreshold quinine solutions (concentration-dependent) and detection thresholds. | (52) |
| **PRH1-TAS2R14** | rs8181 | GWAS | Caffeine | 607 healthy adult (503 subjects for the discovery panel and 104 for the replication panel) subjects between 18 and 45 years old recruited from the general population of Sao Paulo in Brazil. | Associated with caffeine detection threshold. | (52) |
| **PRH1-TAS2R14 (TAS2R20)** | rs10845279 | Candidate | Quinine | 286 twin pairs | Associated with quinine intensity. | (38) |
| **PRH1-TAS2R14 (TAS2R20)** | rs12226920 | Candidate | Quinine | 286 twin pairs | Associated with quinine intensity. | (38) |
| **PRH1-TAS2R14 (TAS2R31)** | rs10772423 | Candidate | Bitterness of Acesulfame Potassium | Intensity ratings (test samples: sucrose, gentiobiose, aspartame, rebaudioside A and D). Participants reported perceived intensity of the test stimuli by rating on a general Labeled Magnitude Scale (gLMS). The gLMS anchors are 0 (‘no sensation’) to 100 (’the strongest imaginable sensation of any kind’), with descriptors at 1.4 (‘barely detectable’), 6 (‘weak’), 17 (‘moderate’), 35 (‘strong’) and 51 (‘very strong’). | Ile homozygotes and heterozygotes perceived greater bitterness compared to the non-functioning Val homozygotes. | (45) |
| **PRH1-TAS2R14 (TAS2R43)** | rs68157013 | Candidate | Bitterness of aloin and saccharin | 55 unrelated volunteers (20–51 year old) of mostly Caucasian and Asian origin. | The most aloin and saccharin sensitive individuals had at least one W35 allele. | (44) |
| **PRH1-TAS2R14 (TAS2R46)** | rs2708377 | GWAS | Bitterness of caffeine | 607 healthy adult (503 subjects for the discovery panel and 104 for the replication panel) subjects between 18 and 45 years old recruited from the general population of Sao Paulo in Brazil. | Associated with the perceived bitterness of caffeine. | (52) |
| **PRH1-TAS2R14 (TAS2R46)** | rs2708377 | GWAS | PROP, quinine and caffeine intensity, detection threshold | 607 healthy adult (503 subjects for the discovery panel and 104 for the replication panel) subjects between 18 and 45 years old recruited from the general population of Sao Paulo in Brazil. | Associated with caffeine detection threshold. | (52) |
| **TAS1R1** | rs17492553 | Candidate | Quinine | 92 adults, primarily of European ancestry (84.8%), female (76%), and middle aged (mean 40.9±12.2 SD). Other ethnicities represented in the sample were Black (5.4%), Hispanic or Latino (5.4%), Asian (3.3%), and other (1.1%). | TT and CT reported significantly lower intensities than CC individuals (applied to the FP and CP). | (53) |
| **TAS1R2** | rs35874116 | Candidate | Vegetable intake (3-day food record) | 441 unrelated subjects. | Val/Val carriers had a higher daily intake of vegetables. | (54) |
| **TAS2R14** | rs3741843 | Candidate | Detection and recognition thresholds, perceived bitter taste intensities of absinthin, amarogentin, cascarillin, grosheimin, quassin, and quinine | 48 unrelated Caucasian subjects: 39 women, 9 men; age range 21-59 years, mean age = 30.6 years. | Associated with grosheimin detection threshold. | (41) |
| **TAS2R16** | rs846664 | Candidate | Salicin bitterness recognition threshold | 595 individuals from 74 African populations and in 94 non-Africans from 11 populations. | Individuals with at least one derived T-allele at polymorphic site 516 have a higher sensitivity to salicin bitterness compared with individuals homozygous for the ancestral G-allele. | (55) |
| **TAS2R16** | rs860170 | Candidate | Food habits questionnaire (liking), PROP tasting, streptovisine, salicin bitterness | An overall number of 183 individuals (belonging to four different geographical regions and highly diversiﬁed in terms of both genetic background and food habits: Italy, North Europe, Maghreb and Sri Lanka.) were recruited and enrolled in the study. Subjects (81 females and 102 males with an average age of 42.71 ± 15.89) did not report any food allergies, were not following any prescribed diet or using drugs that might interfere with taste perception. Most of the participants (N=111) were Italians, with the remaining subjects coming from the Maghreb region (N =18), Sri Lanka (N =26) and Northern Europe (N=28) but recruited in Italy. | The perception of salicin bitterness was associated with A allele. At the genotypic level, a notable trend can be was observed (bitterness levels: AA> GG). | (11) |
| **TAS2R19** | rs12313469 | Candidate | Detection and recognition thresholds, perceived bitter taste intensities of absinthin, amarogentin, cascarillin, grosheimin, quassin, and quinine | 48 unrelated Caucasian subjects: 39 women, 9 men; age range 21-59 years, mean age = 30.6 years. | Associated with grosheimin detection threshold and intensities (weak, moderate, strong, very strong). | (41) |
| **TAS2R20** | rs11054143 | Candidate | Detection and recognition thresholds, perceived bitter taste intensities of absinthin, amarogentin, cascarillin, grosheimin, quassin, and quinine | 48 unrelated Caucasian subjects: 39 women, 9 men; age range 21-59 years, mean age = 30.6 years. | Associated with grosheimin intensities (strong, very strong). | (41) |
| **TAS2R20** | rs7135018 | Candidate | Detection and recognition thresholds, perceived bitter taste intensities of absinthin, amarogentin, cascarillin, grosheimin, quassin, and quinine | 48 unrelated Caucasian subjects: 39 women, 9 men; age range 21-59 years, mean age = 30.6 years. | Associated with grosheimin intensities (strong, very strong) and quinine detection threshold. | (41) |
| **TAS2R20** | rs10845281 | Candidate | Detection and recognition thresholds, perceived bitter taste intensities of absinthin, amarogentin, cascarillin, grosheimin, quassin, and quinine | 48 unrelated Caucasian subjects: 39 women, 9 men; age range 21-59 years, mean age = 30.6 years. | Associated with grosheimin intensities (strong, very strong). | (41) |
| **TAS2R20** | rs11054142 | Candidate | Detection and recognition thresholds, perceived bitter taste intensities of absinthin, amarogentin, cascarillin, grosheimin, quassin, and quinine | 48 unrelated Caucasian subjects: 39 women, 9 men; age range 21-59 years, mean age = 30.6 years. | Associated with grosheimin intensities (strong, very strong). | (41) |
| **TAS2R20** | rs12226920 | Candidate | Detection and recognition thresholds, perceived bitter taste intensities of absinthin, amarogentin, cascarillin, grosheimin, quassin, and quinine | 48 unrelated Caucasian subjects: 39 women, 9 men; age range 21-59 years, mean age = 30.6 years. | Associated with grosheimin intensities (strong, very strong). | (41) |
| **TAS2R20** | rs12226919 | Candidate | Detection and recognition thresholds, perceived bitter taste intensities of absinthin, amarogentin, cascarillin, grosheimin, quassin, and quinine | 48 unrelated Caucasian subjects: 39 women, 9 men; age range 21-59 years, mean age = 30.6 years. | Associated with grosheimin intensities (strong, very strong). | (41) |
| **TAS2R20** | rs79420812 | Candidate | Detection and recognition thresholds, perceived bitter taste intensities of absinthin, amarogentin, cascarillin, grosheimin, quassin, and quinine | 48 unrelated Caucasian subjects: 39 women, 9 men; age range 21-59 years, mean age = 30.6 years. | Associated with grosheimin intensities (strong, very strong). | (41) |
| **TAS2R20** | rs10845279 | Candidate | Detection and recognition thresholds, perceived bitter taste intensities of absinthin, amarogentin, cascarillin, grosheimin, quassin, and quinine | 48 unrelated Caucasian subjects: 39 women, 9 men; age range 21-59 years, mean age = 30.6 years. | Associated with grosheimin intensities (strong, very strong). | (41) |
| **TAS2R20** | rs10845280 | Candidate | Detection and recognition thresholds, perceived bitter taste intensities of absinthin, amarogentin, cascarillin, grosheimin, quassin, and quinine | 48 unrelated Caucasian subjects: 39 women, 9 men; age range 21-59 years, mean age = 30.6 years. | Associated with grosheimin intensities (strong, very strong). | (41) |
| **TAS2R3** | rs765007 | Candidate | Bitterness of unsweetened grapefruit juice, instant espresso | A sample of 198, recruited via posters and word of mouth for a laboratory study of the relationship between variation in oral sensation and dietary behaviors. | Haplotype, allelic variation (TAS2R3, -R4, and -R5) explained variability in coffee bitterness (individuals with 1 or 2 copies of the more responsive haplotype (TGAG) experienced twice as much bitterness compared with individuals homozygous for the less responsive haplotype (CCGT), but these haplotypes did not predict coffee liking. | (42) |
| **TAS2R30** | H1 haplotype |  | Acesulfame Potassium, saccharin, salicin and denatonium benzoate recognition threshold | 60 unrelated caucasians: 41 women, 19 men; age range 20–62 years, mean age 32.3 years, SD = 11.4. | H1 haplotype associated with responses to saccharin and Acesulfame Potassium. | (43) |
| **TAS2R30** | rs2600355 | Candidate | Detection and recognition thresholds, perceived bitter taste intensities of absinthin, amarogentin, cascarillin, grosheimin, quassin, and quinine | 48 unrelated Caucasian subjects: 39 women, 9 men; age range 21-59 years, mean age = 30.6 years. | Associated with amarogentin weak intensity, drosheimin detection threshold and intensities (weak, moderate, strong, very strong). | (41) |
| **TAS2R30** | rs2599404 | Candidate | Detection and recognition thresholds, perceived bitter taste intensities of absinthin, amarogentin, cascarillin, grosheimin, quassin, and quinine | 48 unrelated Caucasian subjects: 39 women, 9 men; age range 21-59 years, mean age = 30.6 years. | Associated with amarogentin weak intensity, drosheimin detection threshold and intensities (weak, moderate, strong, very strong). | (41) |
| **TAS2R30-H1, TAS2R45-H1, TAS2R43-D, TAS2R46-H2, TAS2R31-H2** | LRH21111 | Candidate | Bitter perception of saccharin and acesulfame K | 60 unrelated caucasians: 41 women, 19 men; age range 20–62 years, mean age 32.3 years, SD = 11.4. | LRH11D22 (composed of the five single-gene haplotypes associated with low threshold, which were in tight LD: TAS2R30-H1, TAS2R45-H1, TAS2R43-D, TAS2R46-H2 and TAS2R31-H2) was associated with low threshold response (i.e. high sensitivity) to Acesulfame Potassium and saccharin. | (43) |
| **TAS2R31 (formerly TAS2R44)** | H2 haplotype | Candidate | Acesulfame Potassium, saccharin, salicin and denatonium benzoate recognition threshold | 60 unrelated caucasians: 41 women, 19 men; age range 20–62 years, mean age 32.3 years, SD = 11.4. | H2 haplotype associated with responses to saccharin and Acesulfame Potassium. | (43) |
| **TAS2R31 (formerly TAS2R44)** | rs10845294 | Candidate | Bitterness of Acesulfame Potassium | Intensity ratings (test samples: sucrose, gentiobiose, aspartame, rebaudioside A and D). Participants reported perceived intensity of the test stimuli by rating on a general Labeled Magnitude Scale (gLMS). The gLMS anchors are 0 (‘no sensation’) to 100 (’the strongest imaginable sensation of any kind’), with descriptors at 1.4 (‘barely detectable’), 6 (‘weak’), 17 (‘moderate’), 35 (‘strong’) and 51 (‘very strong’). | The SNP mediates the bitterness of AceK. | (45) |
| **TAS2R31 (formerly TAS2R44)** | rs12370363 | Candidate | Detection and recognition thresholds, perceived bitter taste intensities of absinthin, amarogentin, cascarillin, grosheimin, quassin, and quinine | 48 unrelated Caucasian subjects: 39 women, 9 men; age range 21-59 years, mean age = 30.6 years. | Associated with grosheimin intensities (strong, very strong). | (41) |
| **TAS2R31 (formerly TAS2R44)** | rs10743938 | Candidate | Detection and recognition thresholds, perceived bitter taste intensities of absinthin, amarogentin, cascarillin, grosheimin, quassin, and quinine | 48 unrelated Caucasian subjects: 39 women, 9 men; age range 21-59 years, mean age = 30.6 years. | Associated with grosheimin detection threshold, recognition threshold and intensities (weak, moderate). | (41) |
| **TAS2R42** | rs1650019 | Candidate | Detection and recognition thresholds, perceived bitter taste intensities of absinthin, amarogentin, cascarillin, grosheimin, quassin, and quinine | 48 unrelated Caucasian subjects: 39 women, 9 men; age range 21-59 years, mean age = 30.6 years. | Associated with grosheimin detection threshold. | (41) |
| **TAS2R42** | rs1669413 | Candidate | Detection and recognition thresholds, perceived bitter taste intensities of absinthin, amarogentin, cascarillin, grosheimin, quassin, and quinine | 48 unrelated Caucasian subjects: 39 women, 9 men; age range 21-59 years, mean age = 30.6 years. | Associated with grosheimin detection threshold. | (41) |
| **TAS2R42** | rs1650017 | Candidate | Detection and recognition thresholds, perceived bitter taste intensities of absinthin, amarogentin, cascarillin, grosheimin, quassin, and quinine | 48 unrelated Caucasian subjects: 39 women, 9 men; age range 21-59 years, mean age = 30.6 years. | Associated with grosheimin detection threshold. | (41) |
| **TAS2R42** | rs1669411 | Candidate | Detection and recognition thresholds, perceived bitter taste intensities of absinthin, amarogentin, cascarillin, grosheimin, quassin, and quinine | 48 unrelated Caucasian subjects: 39 women, 9 men; age range 21-59 years, mean age = 30.6 years. | Associated with grosheimin detection threshold. | (41) |
| **TAS2R43** | rs71443637 | Candidate | Detection and recognition thresholds, perceived bitter taste intensities of absinthin, amarogentin, cascarillin, grosheimin, quassin, and quinine | 48 unrelated Caucasian subjects: 39 women, 9 men; age range 21-59 years, mean age = 30.6 years. | Associated with grosheimin detection threshold and intensities (weak, moderate, strong, very strong). | (41) |
| **TAS2R43** | rs68157013 | Candidate | Salicin recognition threshold | 60 unrelated caucasians: 41 women, 19 men; age range 20–62 years, mean age 32.3 years, SD = 11.4. | Associated with saccharin response. | (43) |
| **TAS2R43** | rs35720106 | Candidate | Detection and recognition thresholds, perceived bitter taste intensities of absinthin, amarogentin, cascarillin, grosheimin, quassin, and quinine | 48 unrelated Caucasian subjects: 39 women, 9 men; age range 21-59 years, mean age = 30.6 years. | Associated with grosheimin detection threshold and intensities (weak, moderate, strong, very strong). | (41) |
| **TAS2R43** | D haplotype | Candidate | Acesulfame Potassium, saccharin recognition threshold | 60 unrelated caucasians: 41 women, 19 men; age range 20–62 years, mean age 32.3 years, SD = 11.4. | Associated with saccharin and Acesulfame Potassium response. | (43) |
| **TAS2R43** | Deletion | Candidate | Detection and recognition thresholds, perceived bitter taste intensities of absinthin, amarogentin, cascarillin, grosheimin, quassin, and quinine | 48 unrelated Caucasian subjects: 39 women, 9 men; age range 21-59 years, mean age = 30.6 years. | Associated with amarogentin weak intensity, grosheimin detection threshold and intensities (weak, moderate, strong, very strong). | (41) |
| **TAS2R43** | rs68157013 | Candidate | Detection and recognition thresholds, perceived bitter taste intensities of absinthin, amarogentin, cascarillin, grosheimin, quassin, and quinine | 48 unrelated Caucasian subjects: 39 women, 9 men; age range 21-59 years, mean age = 30.6 years. | Associated with grosheimin detection threshold and intensities (weak, moderate, strong, very strong) . | (41) |
| **TAS2R43** | rs35720106 | Candidate | Coffee liking | 402 individuals come from INGI-CARL a population coming from Carlantino, a small village located in Puglia (Southern Italy); 749 are defined as INGI-FVG, making reference to 6 villages situated in the Friuli Venezia Region in North-Eastern Italy and finally 1160 come from INGI-VB, i.e. a population coming from the Val Borbera Valley in North-Western Italy. The Erasmus Rucphen Family (ERF) study is a cross-sectional cohort including 3,000 living descendants of 22 couples who had at least 6 children baptized in the community church around 1850-1900; 1310 samples were used from this study. Finally, Silk Road (SR) is a cohort of ∼1000 individuals resulting from the sampling of 20 communities coming from 5 nations (Armenia, Azerbaijan, Georgia, Uzbekistan, Tajikistan and Kazakhstan) located along the Silk Road. | Associated with coffee liking. | (56) |
| **TAS2R43** | rs68157013 | Candidate | Coffee liking | 402 individuals come from INGI-CARL a population coming from Carlantino, a small village located in Puglia (Southern Italy); 749 are defined as INGI-FVG, making reference to 6 villages situated in the Friuli Venezia Region in North-Eastern Italy and finally 1160 come from INGI-VB, i.e. a population coming from the Val Borbera Valley in North-Western Italy. The Erasmus Rucphen Family (ERF) study is a cross-sectional cohort including 3,000 living descendants of 22 couples who had at least 6 children baptized in the community church around 1850-1900; 1310 samples were used from this study. Finally, Silk Road (SR) is a cohort of ∼1000 individuals resulting from the sampling of 20 communities coming from 5 nations (Armenia, Azerbaijan, Georgia, Uzbekistan, Tajikistan and Kazakhstan) located along the Silk Road. | Associated with coffee liking. | (56) |
| **TAS2R43** | rs71443637 | Candidate | Coffee liking | 402 individuals come from INGI-CARL a population coming from Carlantino, a small village located in Puglia (Southern Italy); 749 are defined as INGI-FVG, making reference to 6 villages situated in the Friuli Venezia Region in North-Eastern Italy and finally 1160 come from INGI-VB, i.e. a population coming from the Val Borbera Valley in North-Western Italy. The Erasmus Rucphen Family (ERF) study is a cross-sectional cohort including 3,000 living descendants of 22 couples who had at least 6 children baptized in the community church around 1850-1900; 1310 samples were used from this study. Finally, Silk Road (SR) is a cohort of ∼1000 individuals resulting from the sampling of 20 communities coming from 5 nations (Armenia, Azerbaijan, Georgia, Uzbekistan, Tajikistan and Kazakhstan) located along the Silk Road. | Associated with coffee liking. | (56) |
| **TAS2R45** | rs11535673 | Candidate | Absinthin, amarogentin, cascarillin, grosheimin, quassin, and quinine tasting. | 48 unrelated Caucasian subjects: 39 women, 9 men; age range 21-59 years, mean age = 30.6 years. | Associated with grosheimin detection threshold. and intensities (moderate, strong, very strong). | (41) |
| **TAS2R45** | rs3759247 | Candidate | Absinthin, amarogentin, cascarillin, grosheimin, quassin, and quinine tasting. | 48 unrelated Caucasian subjects: 39 women, 9 men; age range 21-59 years, mean age = 30.6 years. | Associated with grosheimin detection threshold. and intensities (moderate, strong, very strong). | (41) |
| **TAS2R45** | rs3759245 | Candidate | Absinthin, amarogentin, cascarillin, grosheimin, quassin, and quinine tasting. | 48 unrelated Caucasian subjects: 39 women, 9 men; age range 21-59 years, mean age = 30.6 years. | Associated with grosheimin detection threshold. and very strong intensity. | (41) |
| **TAS2R45** | Deletion | Candidate | Absinthin, amarogentin, cascarillin, grosheimin, quassin, and quinine tasting. | 48 unrelated Caucasian subjects: 39 women, 9 men; age range 21-59 years, mean age = 30.6 years. | Associated with grosheimin detection threshold. | (41) |
| **TAS2R45** | rs11526470 | Candidate | Absinthin, amarogentin, cascarillin, grosheimin, quassin, and quinine tasting. | 48 unrelated Caucasian subjects: 39 women, 9 men; age range 21-59 years, mean age = 30.6 years. | Associated with grosheimin detection threshold and intensities (moderate, strong, very strong). | (41) |
| **TAS2R45** | rs28581524 | Candidate | Absinthin, amarogentin, cascarillin, grosheimin, quassin, and quinine tasting. | 48 unrelated Caucasian subjects: 39 women, 9 men; age range 21-59 years, mean age = 30.6 years. | Associated with grosheimin detection threshold. and intensities (moderate, strong, very strong). | (41) |
| **TAS2R45** | rs11537119 | Candidate | Absinthin, amarogentin, cascarillin, grosheimin, quassin, and quinine tasting. | 48 unrelated Caucasian subjects: 39 women, 9 men; age range 21-59 years, mean age = 30.6 years. | Associated with grosheimin detection threshold. and intensities (moderate, strong, very strong). | (41) |
| **TAS2R45** | H1 haplotype |  | Acesulfame Potassium, saccharin recognition threshold |  | TAS2R45-H1 associated with responses to saccharin and Acesulfame Potassium. | (57) |
| **TAS2R45** | rs3759244 | Candidate | Absinthin, amarogentin, cascarillin, grosheimin, quassin, and quinine tasting. | 48 unrelated Caucasian subjects: 39 women, 9 men; age range 21-59 years, mean age = 30.6 years. | Associated with grosheimin detection threshold. and intensities (moderate, strong, very strong). | (41) |
| **TAS2R46** | rs2708381 | Candidate | Absinthin, amarogentin, cascarillin, grosheimin, quassin, and quinine tasting. | 48 unrelated Caucasian subjects: 39 women, 9 men; age range 21-59 years, mean age = 30.6 years. | Associated with quinine detection threshold. | (41) |
| **TAS2R46** | H2 haplotype | Candidate | Acesulfame Potassium, saccharin, recognition threshold | 60 unrelated caucasians: 41 women, 19 men; age range 20–62 years, mean age 32.3 years, SD = 11.4. | TAS2R46-H2 associated with responses to saccharin and Acesulfame Potassium. | (43) |
| **TAS2R46** | rs2708380 | Candidate | Absinthin, amarogentin, cascarillin, grosheimin, quassin, and quinine tasting. | 48 unrelated Caucasian subjects: 39 women, 9 men; age range 21-59 years, mean age = 30.6 years. | Associated with amarogentin weak intensity, grosheimin detection threshold and intensities (weak, moderate, strong, very strong). | (41) |
| **TAS2R50** | rs1376251 | Candidate | Absinthin, amarogentin, cascarillin, grosheimin, quassin, and quinine tasting. | 48 unrelated Caucasian subjects: 39 women, 9 men; age range 21-59 years, mean age = 30.6 years. | Associated with grosheimin strong, very strong intensity. | (41) |
| **TAS2R8** | rs1548803 | Candidate | Quinine | 286 twin pairs | Associated with quinine intensity. | (38) |
| **TATDN2** | rs146768860 | GWAS | PROP intensity measurement | This study includes 3500 participants coming from three different Italian populations. Due to geographical, historical, linguistic and/or cultural factors, these populations showed evidences of genetic isolation. Also this study included total of 496 subjects participated in the study (206 males and 290 females), coming from 20 different communities of six countries in the Caucasus and Central Asia: Georgia, Armenia, Azerbaijan, Uzbekistan, Kazakhstan and | Associated with PROP phenotype. | (49) |
| **TATDN2** | rs2003595 | GWAS | PROP intensity measurement | This study includes 3500 participants coming from three different Italian populations. Due to geographical, historical, linguistic and/or cultural factors, these populations showed evidences of genetic isolation. Also this study included total of 496 subjects participated in the study (206 males and 290 females), coming from 20 different communities of six countries in the Caucasus and Central Asia: Georgia, Armenia, Azerbaijan, Uzbekistan, Kazakhstan and | Associated with PROP phenotype. | (49) |
| **TATDN2** | rs2005903 | GWAS | PROP intensity measurement | This study includes 3500 participants coming from three different Italian populations. Due to geographical, historical, linguistic and/or cultural factors, these populations showed evidences of genetic isolation. Also this study included total of 496 subjects participated in the study (206 males and 290 females), coming from 20 different communities of six countries in the Caucasus and Central Asia: Georgia, Armenia, Azerbaijan, Uzbekistan, Kazakhstan and | Associated with PROP phenotype. | (49) |
| **TATDN2** | rs2241313 | GWAS | PROP intensity measurement | This study includes 3500 participants coming from three different Italian populations. Due to geographical, historical, linguistic and/or cultural factors, these populations showed evidences of genetic isolation. Also this study included total of 496 subjects participated in the study (206 males and 290 females), coming from 20 different communities of six countries in the Caucasus and Central Asia: Georgia, Armenia, Azerbaijan, Uzbekistan, Kazakhstan and | Associated with PROP phenotype. | (49) |
| **TATDN2** | rs2241314 | GWAS | PROP intensity measurement | This study includes 3500 participants coming from three different Italian populations. Due to geographical, historical, linguistic and/or cultural factors, these populations showed evidences of genetic isolation. Also this study included total of 496 subjects participated in the study (206 males and 290 females), coming from 20 different communities of six countries in the Caucasus and Central Asia: Georgia, Armenia, Azerbaijan, Uzbekistan, Kazakhstan and | Associated with PROP phenotype. | (49) |
| **TATDN2** | rs2270454 | GWAS | PROP intensity measurement | This study includes 3500 participants coming from three different Italian populations. Due to geographical, historical, linguistic and/or cultural factors, these populations showed evidences of genetic isolation. Also this study included total of 496 subjects participated in the study (206 males and 290 females), coming from 20 different communities of six countries in the Caucasus and Central Asia: Georgia, Armenia, Azerbaijan, Uzbekistan, Kazakhstan and | Associated with PROP phenotype. | (49) |
| **TATDN2** | rs56284018 | GWAS | PROP | This study includes 3500 participants coming from three different Italian populations. Due to geographical, historical, linguistic and/or cultural factors, these populations showed evidences of genetic isolation. Also this study included total of 496 subjects participated in the study (206 males and 290 females), coming from 20 different communities of six countries in the Caucasus and Central Asia: Georgia, Armenia, Azerbaijan, Uzbekistan, Kazakhstan and | Associated with PROP phenotype. | (49) |
| **TATDN2** | rs78537477 | GWAS | PROP | This study includes 3500 participants coming from three different Italian populations. Due to geographical, historical, linguistic and/or cultural factors, these populations showed evidences of genetic isolation. Also this study included total of 496 subjects participated in the study (206 males and 290 females), coming from 20 different communities of six countries in the Caucasus and Central Asia: Georgia, Armenia, Azerbaijan, Uzbekistan, Kazakhstan and | Associated with PROP phenotype. | (49) |
| **TRPV1** | rs224547 | Candidate | Bitterness of alcohol | 91 individuals of European (58 women and 35 men) with a mean age of 25 (±0.69 SEM) years. | Associated with the summary AUC scores for bitterness of alcohol (AA homozygotes having the highest mean area). | (7) |
| **TRPV1** | rs4790521 | Candidate | Bitterness of alcohol | 91 individuals of European (58 women and 35 men) with a mean age of 25 (±0.69 SEM) years. | Associated with the summary AUC scores for bitterness of alcohol (CC homozygotes had the highest mean area for bitterness). | (7) |
| **ZNF804B** | rs4727180 | GWAS | PROP intensity measurement | For the discovery sample, participants were a subset of adolescent and young adult twins and their singleton siblings who have participated in previous studies of the genetics of skin moles and cognition. The sample for which taste sensitivity results were available consisted of females and males and included monozygotic (MZ) and dizygotic (DZ) twin pairs and their siblings. For the replication sample, experimenters recruited and tested participants at an annual convention of twins, Twins Days Festival, in Twinsburg, OH, USA. Testing occurred at the 2009 festival in August. Characteristic: Discovery: n=1457, males/females 671/786, age (mean + standard deviation): 18 + 2, age range (years) 11–25; Replication: n=73,  males/females 16/57,  age (mean + standard deviation) 42 + 17,  age range (years): 21–82. | Associated with PROP phenotype. | (48) |
| **DFNA5** | rs73082019 | GWAS | Food preference questionnaire | Participants were enrolled with written informed consent under National | Association with preference of dark chocolate (GWAS). | (51) |
| **DIRC3-AS1** | rs4141835 | GWAS | PROP intensity measurement | For the discovery sample, participants were a subset of adolescent and young adult twins and their singleton siblings who have participated in previous studies of the genetics of skin moles and cognition. The sample for which taste sensitivity results were available consisted of females and males and included monozygotic (MZ) and dizygotic (DZ) twin pairs and their siblings. For the replication sample, experimenters recruited and tested participants at an annual convention of twins, Twins Days Festival, in Twinsburg, OH, USA. Testing occurred at the 2009 festival in August. Characteristic: Discovery: n=1457, males/females 671/786, age (mean + standard deviation): 18 + 2, age range (years) 11–25; Replication: n=73,  males/females 16/57,  age (mean + standard deviation) 42 + 17,  age range (years): 21–82. | Associated with PROP intensity ratings. | (48) |

**Supplementary Table 4 Study characteristics of genetic association studies related to bitter taste preferences (no associations)**

PROP: 6-n-propylthiouracil, AceK: Acesulfame Potassium, gLMS: generalized Labeled Magnitude Scale, pMT: PROP medium-taster, pNT: PROP non-taster, pST: PROP super-taster

**Supplementary Table 4 Study characteristics of genetic association studies related to bitter taste preferences (no associations)**

| **Gene** | **SNP** | **Discovery method** | **Phenotype assessment method** | **Study population characteristics** | **Reference (no association)** |
| --- | --- | --- | --- | --- | --- |
| **CA4** | rs9905484 | Candidate | Bitterness of quinine was rated. | A total of 600 subjects were enrolled in this study, but 28 were eliminated from the analysis because they (or their co-twin) failed to follow instructions or had a history of alcohol dependence. The remaining 572 subjects were, on average, middle age (range 21–82 years), and most were Caucasian and female. | (58) |
| **CA6** | rs12748400 | Candidate | PROP intensity was rated on a gLMS. | 243 reportedly healthy participants (146 women), aged 18–45 were recruited from the Pennsylvania State University campus and surrounding area. | (59) |
| **CA6** | rs17032907 | Candidate | PROP intensity was rated on a gLMS. | 243 reportedly healthy participants (146 women), aged 18–45 were recruited from the Pennsylvania State University campus and surrounding area. | (59) |
| **CA6** | rs2274327 | Candidate | PROP intensity was rated on a gLMS. | 243 reportedly healthy participants (146 women), aged 18–45 were recruited from the Pennsylvania State University campus and surrounding area. | (59) |
| **CA6** | rs2274328 | Candidate | PROP intensity was rated on a gLMS. | 243 reportedly healthy participants (146 women), aged 18–45 were recruited from the Pennsylvania State University campus and surrounding area. | (59) |
| **CA6** | rs2274333 | Candidate | Bitterness of quinine was rated. | A total of 600 subjects were enrolled in this study, but 28 were eliminated from the analysis because they (or their co-twin) failed to follow instructions or had a history of alcohol dependence. The remaining 572 subjects were, on average, middle age (range 21–82 years), and most were Caucasian and female. | (58) |
| **CA6** | rs2274334 | Candidate | PROP intensity was rated on a gLMS. | 243 reportedly healthy participants (146 women), aged 18–45 were recruited from the Pennsylvania State University campus and surrounding area. | (59) |
| **CA6** | rs3737665 | Candidate | PROP intensity was rated on a gLMS. | 243 reportedly healthy participants (146 women), aged 18–45 were recruited from the Pennsylvania State University campus and surrounding area. | (59) |
| **CA6** | rs3765964 | Candidate | PROP intensity was rated on a gLMS. | 243 reportedly healthy participants (146 women), aged 18–45 were recruited from the Pennsylvania State University campus and surrounding area. | (59) |
| **CA6** | rs3765965 | Candidate | PROP intensity was rated on a gLMS. | 243 reportedly healthy participants (146 women), aged 18–45 were recruited from the Pennsylvania State University campus and surrounding area. | (59) |
| **CA6** | rs3765967 | Candidate | PROP intensity was rated on a gLMS. | 243 reportedly healthy participants (146 women), aged 18–45 were recruited from the Pennsylvania State University campus and surrounding area. | (59) |
| **CA6** | rs3765968 | Candidate | PROP intensity was rated on a gLMS. | 243 reportedly healthy participants (146 women), aged 18–45 were recruited from the Pennsylvania State University campus and surrounding area. | (59) |
| **CA6** | rs7545200 | Candidate | PROP intensity was rated on a gLMS. | 243 reportedly healthy participants (146 women), aged 18–45 were recruited from the Pennsylvania State University campus and surrounding area. | (59) |
| **CASC6** | rs10485099 | Candidate | Bitterness of quinine was rated. | A total of 600 subjects were enrolled in this study, but 28 were eliminated from the analysis because they (or their co-twin) failed to follow instructions or had a history of alcohol dependence. The remaining 572 subjects were, on average, middle age (range 21–82 years), and most were Caucasian and female. | (58) |
| **CD36** | rs1527483 | Candidate | Participants were assessed for PROP taster status (three-solution test: the taste intensity rating for three suprathreshold PROP (0.032, 0.32, and 3.2 mmol/L) and sodium chloride (NaCl; 0.01, 0.1, 1.0 mol/L) solutions was collected in each subject, by using the Labeled Magnitude Scale , which gave subjects the freedom to rate the PROP bitterness relative to the “strongest imaginable” oral stimulus they had ever experienced in their life. Each stimulation was followed by oral rinsing with spring water. The order of presentation of the taste stimuli (PROP or NaCl) in 10 mL samples was reversed in the two sessions. Concentrations were tested in a random order, and the interstimulus interval was set at 60 s. The mean rating of the two replicates was calculated, and functions of perceived taste intensity for PROP and NaCl for each subject were generated from the results. Subjects who gave lower intensity ratings to PROP than to NaCl were classified as PROP non-tasters, those who gave similar ratings to the two stimuli were classified as medium tasters, and those who gave higher ratingsto PROP than to NaCl were classified as super-tasters.) | Sixty-four non-smoking Caucasian subjects (23 males, 41 females, age 27.6 ± 0.85 years) from Sardinia, Italy were recruited according to standard procedures. All were normal weight with a body mass index (BMI) ranging from 18.6 to 25.3 kg/m2 , had maintained a stable weight in the previous. 3 months, and did not follow a diet or take medications that might interfere with taste function. Subjects with extreme scores for restraint and/or disinhibition and/or perceived hunger, assessed by theThree-Factor Eating Questionnaire, were excluded from the study. 64 tested for PROP sensitivity, 36 for tested for sensitivity to oleic acid esterified with glycerol (triolein). | (60) |
| **CD36** | rs1761667 | Candidate | Participants were assessed for PROP taster status (three-solution test: the taste intensity rating for three suprathreshold PROP (0.032, 0.32, and 3.2 mmol/L) and sodium chloride (NaCl; 0.01, 0.1, 1.0 mol/L) solutions was collected in each subject, by using the Labeled Magnitude Scale , which gave subjects the freedom to rate the PROP bitterness relative to the “strongest imaginable” oral stimulus they had ever experienced in their life. Each stimulation was followed by oral rinsing with spring water. The order of presentation of the taste stimuli (PROP or NaCl) in 10 mL samples was reversed in the two sessions. Concentrations were tested in a random order, and the interstimulus interval was set at 60 s. The mean rating of the two replicates was calculated, and functions of perceived taste intensity for PROP and NaCl for each subject were generated from the results. Subjects who gave lower intensity ratings to PROP than to NaCl were classified as PROP non-tasters, those who gave similar ratings to the two stimuli were classified as medium tasters, and those who gave higher ratingsto PROP than to NaCl were classified as super-tasters.) | Sixty-four non-smoking Caucasian subjects (23 males, 41 females, age 27.6 ± 0.85 years) from Sardinia, Italy were recruited according to standard procedures. All were normal weight with a body mass index (BMI) ranging from 18.6 to 25.3 kg/m2, had maintained a stable weight in the previous. 3 months, and did not follow a diet or take medications that might interfere with taste function. Subjects with extreme scores for restraint and/or disinhibition and/or perceived hunger, assessed by theThree-Factor Eating Questionnaire, were excluded from the study. 64 tested for PROP sensitivity, 36 for tested for sensitivity to oleic acid esterified with glycerol (triolein). | (60) |
| **CDYL2** | rs13333767 | Candidate | Bitterness of quinine was rated. | A total of 600 subjects were enrolled in this study, but 28 were eliminated from the analysis because they (or their co-twin) failed to follow instructions or had a history of alcohol dependence. The remaining 572 subjects were, on average, middle age (range 21–82 years), and most were Caucasian and female. | (58) |
| **FFAR1** | rs2301151 | Candidate | Bitterness of quinine was rated. | A total of 600 subjects were enrolled in this study, but 28 were eliminated from the analysis because they (or their co-twin) failed to follow instructions or had a history of alcohol dependence. The remaining 572 subjects were, on average, middle age (range 21–82 years), and most were Caucasian and female. | (58) |
| **GNAT3** | rs1524600 | Candidate | Bitterness of quinine was rated. | A total of 600 subjects were enrolled in this study, but 28 were eliminated from the analysis because they (or their co-twin) failed to follow instructions or had a history of alcohol dependence. The remaining 572 subjects were, on average, middle age (range 21–82 years), and most were Caucasian and female. | (58) |
| **INTS4** | rs3819256 | Candidate | Bitterness of quinine was rated. | A total of 600 subjects were enrolled in this study, but 28 were eliminated from the analysis because they (or their co-twin) failed to follow instructions or had a history of alcohol dependence. The remaining 572 subjects were, on average, middle age (range 21–82 years), and most were Caucasian and female. | (58) |
| **KCNJ5** | rs4937384 | Candidate | Bitterness of quinine was rated. | A total of 600 subjects were enrolled in this study, but 28 were eliminated from the analysis because they (or their co-twin) failed to follow instructions or had a history of alcohol dependence. The remaining 572 subjects were, on average, middle age (range 21–82 years), and most were Caucasian and female. | (58) |
| **LOC105377272** | rs4127802 | Candidate | Bitterness of quinine was rated. | A total of 600 subjects were enrolled in this study, but 28 were eliminated from the analysis because they (or their co-twin) failed to follow instructions or had a history of alcohol dependence. The remaining 572 subjects were, on average, middle age (range 21–82 years), and most were Caucasian and female. | (58) |
| **LOC107986812** | rs7792845 | Candidate | Bitterness of quinine was rated. | A total of 600 subjects were enrolled in this study, but 28 were eliminated from the analysis because they (or their co-twin) failed to follow instructions or had a history of alcohol dependence. The remaining 572 subjects were, on average, middle age (range 21–82 years), and most were Caucasian and female. | (58) |
| **NA** | rs10966900 | Candidate | Bitterness of quinine was rated. | A total of 600 subjects were enrolled in this study, but 28 were eliminated from the analysis because they (or their co-twin) failed to follow instructions or had a history of alcohol dependence. The remaining 572 subjects were, on average, middle age (range 21–82 years), and most were Caucasian and female. | (58) |
| **NA** | rs1308724 | Candidate | Intensity ratings (test samples: sucrose, gentiobiose, aspartame, rebaudioside A and D). Participants reported perceived intensity of the test stimuli by rating on a general Labeled Magnitude Scale (gLMS). The gLMS anchors are 0 (‘no sensation’) to 100 (’the strongest imaginable sensation of any kind’), with descriptors at 1.4 (‘barely detectable’), 6 (‘weak’), 17 (‘moderate’), 35 (‘strong’) and 51 (‘very strong’). | Here, we present data from a follow-up study to a larger project on the genetics of oral sensation (Project GIANT-CS). Participants who had completed the main study and had been genotyped were invited to return to our laboratory to taste a variety of tastants not originally included in the main study, including multiple non-nutritive sweeteners and disaccharides. 122 participants returned to complete the follow-up study. The study was conducted in the Sensory Evaluation Center at the Pennsylvania State University in individual testing test booths under white light. After participants were re-consented in writing for the follow-up study, they participated in a 3- minute training session in a multifunction space in our facility prior to entering the test booths. In isolated testing booths, participants rated bitter, sweet, and metallic sensations on a computerized general Labeled Magnitude Scale (gLMS). Participants were screened prior to the start of the main study. Eligibility criteria included: between 18–45 years old, not pregnant or breastfeeding, non-smoker (had not smoked in the last 30 days), no known defects of smell or taste, no lip, cheek or tongue piercings, no history of any condition involving chronic pain, not currently taking any prescription pain medication, no reported history of choking or difficulty swallowing and no history of thyroid disease. Here, we report data from 122 participants (44 men), with a mean age 27.7 (±7.89) years. Self-reported race and ethnicity was collected based on criteria provided by the 1997 OMB Directive 15. This population is largely of European ancestry (n=88), with marginal representation from other ancestry, African (n=2) and Asian (n=21), with 9 individuals choosing to not disclose their ancestry. | (61) |
|  |  | Candidate | A general labeled magnitude scale (gLMS) was used to collect perceived intensity of suprathreshold stimuli (AceK sweetness). This scale ranges from 0 (“no sensation”) to 100 (“the strongest imaginable sensation of any kind”), with intermediate descriptors at 1.4 (“barely detectable”), 6 (“weak”), 17 (“moderate”), 35 (“strong”), and 51 (“very strong”). | Eligibility criteria included the following: between 18 and 45 years old; not pregnant or breastfeeding; nonsmoker (had not smoked in the last 30 days); no known defects of smell or taste; no lip, cheek, or tongue piercings; no history Bitterness of Acesulfame Potassium Varies With TAS2R Polymorphisms 381 of any condition involving chronic pain; not currently taking any prescription pain medication; no reported history of choking or difficulty swallowing; and no history of thyroid disease. Participants also needed to be willing to provide a DNA sample via saliva. DNA samples were available from 147 participants. Race and ethnicity was self-reported using categories provided by the 1997 OMB Directive 15. To minimize potential population stratification, which can potentially cause false negatives and false positives in gene association studies (Hamer and Sirota 2000), individuals with Asian (n = 18), African (n = 5), or unknown (n = 15) ancestry were excluded from the present analyses. Thus, we report data from 108 participants (34 men) of European ancestry, with a mean age of 27.4 (±8.1 SD) years. Results were not substantively different in the mixed ancestry sample, but we report only the results for the European–American participants to facilitate interpretation of the linkage disequilibrium (LD) plots. | (8) |
|  |  | Candidate | Bitterness of quinine was rated. | A total of 600 subjects were enrolled in this study, but 28 were eliminated from the analysis because they (or their co-twin) failed to follow instructions or had a history of alcohol dependence. The remaining 572 subjects were, on average, middle age (range 21–82 years), and most were Caucasian and female. | (38) |
| **NA** | rs4481887 | Candidate | Bitterness of quinine was rated. | A total of 600 subjects were enrolled in this study, but 28 were eliminated from the analysis because they (or their co-twin) failed to follow instructions or had a history of alcohol dependence. The remaining 572 subjects were, on average, middle age (range 21–82 years), and most were Caucasian and female. | (58) |
| **NA** | rs846672 | Candidate | Bitterness of quinine was rated. | A total of 600 subjects were enrolled in this study, but 28 were eliminated from the analysis because they (or their co-twin) failed to follow instructions or had a history of alcohol dependence. The remaining 572 subjects were, on average, middle age (range 21–82 years), and most were Caucasian and female. | (58) |
| **OR11H7** | rs1953558 | Candidate | Bitterness of quinine was rated. | A total of 600 subjects were enrolled in this study, but 28 were eliminated from the analysis because they (or their co-twin) failed to follow instructions or had a history of alcohol dependence. The remaining 572 subjects were, on average, middle age (range 21–82 years), and most were Caucasian and female. | (58) |
| **OR7D4** | rs5020278 | Candidate | Bitterness of quinine was rated. | A total of 600 subjects were enrolled in this study, but 28 were eliminated from the analysis because they (or their co-twin) failed to follow instructions or had a history of alcohol dependence. The remaining 572 subjects were, on average, middle age (range 21–82 years), and most were Caucasian and female. | (58) |
| **OR7D4** | rs61729907 | Candidate | Bitterness of quinine was rated. | A total of 600 subjects were enrolled in this study, but 28 were eliminated from the analysis because they (or their co-twin) failed to follow instructions or had a history of alcohol dependence. The remaining 572 subjects were, on average, middle age (range 21–82 years), and most were Caucasian and female. | (58) |
| **PRH1-TAS2R14 (TAS2R31)** | rs10845293 | Candidate | Responsiveness to a 3.2-mM solution of PROP (MP Biomedicals, OH, USA) was measured using a generalized labeled magnitude scale (gLMS), employing the method described in Bajec and Pickering.. PTS categories were defined based on the cutoff values used by Porubcan and Vickers (2005) and Bajec and Pickering (2010): pNTs, <10.9 mm; pMTs, 10.9–61.5 mm; pSTs, >61.5 mm. | Fifty-three participants were recruited from the students, staff, and faculty of Brock University, and comprised of 36 females and 17 males with a mean age of 26.5 years ±9.7 SD, all of whom were nonsmokers. | (62) |
| **PRH1-TAS2R14 (TAS2R50)** | rs10772397 | Candidate | Bitterness of quinine was rated. | A total of 600 subjects were enrolled in this study, but 28 were eliminated from the analysis because they (or their co-twin) failed to follow instructions or had a history of alcohol dependence. The remaining 572 subjects were, on average, middle age (range 21–82 years), and most were Caucasian and female. | (58) |
| **SCNN1D** | rs586965 | Candidate | Bitterness of quinine was rated. | A total of 600 subjects were enrolled in this study, but 28 were eliminated from the analysis because they (or their co-twin) failed to follow instructions or had a history of alcohol dependence. The remaining 572 subjects were, on average, middle age (range 21–82 years), and most were Caucasian and female. | (58) |
| **SRPK2** | rs1204064 | Candidate | Responsiveness to a 3.2-mM solution of PROP (MP Biomedicals, OH, USA) was measured using a generalized labeled magnitude scale (gLMS), employing the method described in Bajec and Pickering.. PTS categories were defined based on the cutoff values used by Porubcan and Vickers (2005) and Bajec and Pickering (2010): pNTs, <10.9 mm; pMTs, 10.9–61.5 mm; pSTs, >61.5 mm. | Fifty-three participants were recruited from the students, staff, and faculty of Brock University, and comprised of 36 females and 17 males with a mean age of 26.5 years ±9.7 SD, all of whom were nonsmokers. | (62) |
| **TAS1R1** | rs34160967 | Candidate | Adults used a general labeled magnitude scale (gLMS) to rate the taste intensity of oral stimuli (quinine). As an intensity scale, the gLMS is a vertical scale ranging from “no sensation” (0) at the bottom to “strongest imaginable sensation of any kind” (100) at the top and with other adjectives (“barely detectable,” “weak,” “moderate,” “strong,” and “very strong”) placed in a quasi-logarithmic fashion. | A convenience sample of reportedly healthy, nonsmoking adults was recruited from the University of Connecticut community to participate in an observational study of variation in oral sensation, diet, and health. Exclusion criteria included pregnancy, severe food allergies, and thyroid disease. The study sample included 92 adults, primarily of European ancestry (84.8%), female (76%), and middle aged (mean 40.9±12.2 SD). Other ethnicities represented in the sample were Black (5.4%), Hispanic or Latino (5.4%), Asian (3.3%), and other (1.1%). | (63) |
| **TAS1R1** | rs34160967 | Candidate | Bitterness of quinine was rated. | A total of 600 subjects were enrolled in this study, but 28 were eliminated from the analysis because they (or their co-twin) failed to follow instructions or had a history of alcohol dependence. The remaining 572 subjects were, on average, middle age (range 21–82 years), and most were Caucasian and female. | (58) |
| **TAS1R2** | rs35874116 | Candidate | Bitterness of quinine was rated. | A total of 600 subjects were enrolled in this study, but 28 were eliminated from the analysis because they (or their co-twin) failed to follow instructions or had a history of alcohol dependence. The remaining 572 subjects were, on average, middle age (range 21–82 years), and most were Caucasian and female. | (58) |
| **TAS1R2** | rs9701796 | Candidate | Bitterness of quinine was rated. | A total of 600 subjects were enrolled in this study, but 28 were eliminated from the analysis because they (or their co-twin) failed to follow instructions or had a history of alcohol dependence. The remaining 572 subjects were, on average, middle age (range 21–82 years), and most were Caucasian and female. | (58) |
| **TAS1R3** | rs111615792 | Candidate | Bitterness of quinine was rated. | A total of 600 subjects were enrolled in this study, but 28 were eliminated from the analysis because they (or their co-twin) failed to follow instructions or had a history of alcohol dependence. The remaining 572 subjects were, on average, middle age (range 21–82 years), and most were Caucasian and female. | (58) |
| **TAS1R3** | rs307355 | Candidate | Bitterness of quinine was rated. | A total of 600 subjects were enrolled in this study, but 28 were eliminated from the analysis because they (or their co-twin) failed to follow instructions or had a history of alcohol dependence. The remaining 572 subjects were, on average, middle age (range 21–82 years), and most were Caucasian and female. | (58) |
| **TAS1R3** | rs307377 | Candidate | Bitterness of quinine was rated. | A total of 600 subjects were enrolled in this study, but 28 were eliminated from the analysis because they (or their co-twin) failed to follow instructions or had a history of alcohol dependence. The remaining 572 subjects were, on average, middle age (range 21–82 years), and most were Caucasian and female. | (58) |
| **TAS1R3** | rs35744813 | Candidate | Bitterness of quinine was rated. | A total of 600 subjects were enrolled in this study, but 28 were eliminated from the analysis because they (or their co-twin) failed to follow instructions or had a history of alcohol dependence. The remaining 572 subjects were, on average, middle age (range 21–82 years), and most were Caucasian and female. | (58) |
| **TAS1R3** | rs76755863 | Candidate | Bitterness of quinine was rated. | A total of 600 subjects were enrolled in this study, but 28 were eliminated from the analysis because they (or their co-twin) failed to follow instructions or had a history of alcohol dependence. The remaining 572 subjects were, on average, middle age (range 21–82 years), and most were Caucasian and female. | (58) |
| **TAS2R1** | rs2234233 | Candidate | Bitterness of quinine was rated. | A total of 600 subjects were enrolled in this study, but 28 were eliminated from the analysis because they (or their co-twin) failed to follow instructions or had a history of alcohol dependence. The remaining 572 subjects were, on average, middle age (range 21–82 years), and most were Caucasian and female. | (58) |
| **TAS2R13** | rs1015443 | Candidate | Labeled Magnitude Scale (gLMS) followed by a short practice session. Participants also rated the perceived intensity of five different prototypical tastants presented regionally to each quadrant of the tongue. Participants also rated the perceived intensity of a chemesthetic stimulus (either capsaicin, ethanol or piperine) applied to the left and right circumvallate (CV) papillae, which are located on the posterior tongue, forming a rearward pointing chevron of 8 to 12 dome-shaped structures. Detection thresholds were collected for chemesthetic stimuli using a forced choice method based on ASTM method E-679. As the final stimulus within a session, participants rated the overall intensity of a whole mouth swish-and-spit chemesthetic stimulus | Individuals were recruited from the Pennsylvania State University campus and surrounding community (State College, PA) as part of a larger study on the genetics of oral sensation. All four sessions were completed by 106 participants (40 men) with a mean age of 25.2± 0.63 (SEM). Participants reported ethnicity using the recommended wording from the 1997 OMB Directive 15 guidelines. The majority of participants reported Caucasian ancestry (n=69), followed by Asian (n=15) and African American (n=1); 11 participants chose not to disclose their ancestry. Prior to study enrollment, individuals interested in participating completed an online screening questionnaire. Eligibility criteria included: between 18–45 years old, not pregnant nor breastfeeding, had not smoked in the last 30 days, no known defects of smell nor taste, no oral piercings (lip, cheek or tongue), no history of chronic pain, not currently taking any prescription pain medication, no history of choking or difficulty swallowing and no history of thyroid disease. | (34) |
| **TAS2R13** | rs1015443 | Candidate | Responsiveness to a 3.2-mM solution of PROP (MP Biomedicals, OH, USA) was measured using a generalized labeled magnitude scale (gLMS), employing the method described in Bajec and Pickering.. PTS categories were defined based on the cutoff values used by Porubcan and Vickers (2005) and Bajec and Pickering (2010): pNTs, <10.9 mm; pMTs, 10.9–61.5 mm; pSTs, >61.5 mm. | Fifty-three participants were recruited from the students, staff, and faculty of Brock University, and comprised of 36 females and 17 males with a mean age of 26.5 years ±9.7 SD, all of whom were nonsmokers. | (62) |
|  |  | Candidate | The generalized Labeled Magnitude Scale (gLMS) was used to collect psychophysical ratings for stimuli. This scale ranges from 0 to 100 and asks participants to rate the intensity they experience relative to the ‘strongest imaginable sensation of any kind’ (100). Adjective labels on the scale include: no sensation, barely detectable, weak, moderate, strong, and very strong, located at 0, 1.4, 6, 17, 35, and 51 respectively. The irritants presented in this study consisted of ethanol, piperine, and capsaicin. | Participants, 18 to 45 years old, were recruited from the Pennsylvania State University campus and surrounding area. Those interested in participating completed an online survey to determine if they met inclusion criteria. Qualifications include: not pregnant or breastfeeding, non-smoker, no tongue, cheek or lip piercings, no known smell or taste defect, no hyperactive thyroid, no history of chronic pain, and willingness to provide a  salivary DNA sample. Of the participants who completed sessions 2–4 (total n=130), the majority reported European ancestry (n=93), with 18 reporting Asian ancestry and 2 reporting African ancestry; 17 individuals declined to provide ancestry. Due to potential differences in allele frequencies across ancestry and the possibility of population stratification, all of the results here are restricted to individuals of European ancestry, resulting in a cohort of 58 women and 35 men with a mean age of 25 (±0.69 SEM) years. | (7) |
| **TAS2R16** | rs2233989 | Candidate | Responsiveness to a 3.2-mM solution of PROP (MP Biomedicals, OH, USA) was measured using a generalized labeled magnitude scale (gLMS), employing the method described in Bajec and Pickering.. PTS categories were defined based on the cutoff values used by Porubcan and Vickers (2005) and Bajec and Pickering (2010): pNTs, <10.9 mm; pMTs, 10.9–61.5 mm; pSTs, >61.5 mm. | Fifty-three participants were recruited from the students, staff, and faculty of Brock University, and comprised of 36 females and 17 males with a mean age of 26.5 years ±9.7 SD, all of whom were nonsmokers. | (62) |
| **TAS2R16** | rs978739 | Candidate | Responsiveness to a 3.2-mM solution of PROP (MP Biomedicals, OH, USA) was measured using a generalized labeled magnitude scale (gLMS), employing the method described in Bajec and Pickering.. PTS categories were defined based on the cutoff values used by Porubcan and Vickers (2005) and Bajec and Pickering (2010): pNTs, <10.9 mm; pMTs, 10.9–61.5 mm; pSTs, >61.5 mm. | Fifty-three participants were recruited from the students, staff, and faculty of Brock University, and comprised of 36 females and 17 males with a mean age of 26.5 years ±9.7 SD, all of whom were nonsmokers. | (62) |
| **TAS2R19** | rs12578654 | Candidate | Responsiveness to a 3.2-mM solution of PROP (MP Biomedicals, OH, USA) was measured using a generalized labeled magnitude scale (gLMS), employing the method described in Bajec and Pickering.. PTS categories were defined based on the cutoff values used by Porubcan and Vickers (2005) and Bajec and Pickering (2010): pNTs, <10.9 mm; pMTs, 10.9–61.5 mm; pSTs, >61.5 mm. | Fifty-three participants were recruited from the students, staff, and faculty of Brock University, and comprised of 36 females and 17 males with a mean age of 26.5 years ±9.7 SD, all of whom were nonsmokers. | (62) |
| **TAS2R19** | rs4763235 | Candidate | Responsiveness to a 3.2-mM solution of PROP (MP Biomedicals, OH, USA) was measured using a generalized labeled magnitude scale (gLMS), employing the method described in Bajec and Pickering.. PTS categories were defined based on the cutoff values used by Porubcan and Vickers (2005) and Bajec and Pickering (2010): pNTs, <10.9 mm; pMTs, 10.9–61.5 mm; pSTs, >61.5 mm. | Fifty-three participants were recruited from the students, staff, and faculty of Brock University, and comprised of 36 females and 17 males with a mean age of 26.5 years ±9.7 SD, all of whom were nonsmokers. | (62) |
| **TAS2R3** | rs2270009 | Candidate | Bitterness of quinine was rated. | A total of 600 subjects were enrolled in this study, but 28 were eliminated from the analysis because they (or their co-twin) failed to follow instructions or had a history of alcohol dependence. The remaining 572 subjects were, on average, middle age (range 21–82 years), and most were Caucasian and female. | (58) |
| **TAS2R3** | rs765007 | Candidate | Labeled Magnitude Scale (gLMS) followed by a short practice session. Participants also rated the perceived intensity of five different prototypical tastants presented regionally to each quadrant of the tongue. Participants also rated the perceived intensity of a chemesthetic stimulus (either capsaicin, ethanol or piperine) applied to the left and right circumvallate (CV) papillae, which are located on the posterior tongue, forming a rearward pointing chevron of 8 to 12 dome-shaped structures. Detection thresholds were collected for chemesthetic stimuli using a forced choice method based on ASTM method E-679. As the final stimulus within a session, participants rated the overall intensity of a whole mouth swish-and-spit chemesthetic stimulus | Individuals were recruited from the Pennsylvania State University campus and surrounding community (State College, PA) as part of a larger study on the genetics of oral sensation. All four sessions were completed by 106 participants (40 men) with a mean age of 25.2± 0.63 (SEM). Participants reported ethnicity using the recommended wording from the 1997 OMB Directive 15 guidelines. The majority of participants reported Caucasian ancestry (n=69), followed by Asian (n=15) and African American (n=1); 11 participants chose not to disclose their ancestry. Prior to study enrollment, individuals interested in participating completed an online screening questionnaire. Eligibility criteria included: between 18–45 years old, not pregnant nor breastfeeding, had not smoked in the last 30 days, no known defects of smell nor taste, no oral piercings (lip, cheek or tongue), no history of chronic pain, not currently taking any prescription pain medication, no history of choking or difficulty swallowing and no history of thyroid disease. | (64) |
| **TAS2R39** | rs4726600 | Candidate | Bitterness of quinine was rated. | A total of 600 subjects were enrolled in this study, but 28 were eliminated from the analysis because they (or their co-twin) failed to follow instructions or had a history of alcohol dependence. The remaining 572 subjects were, on average, middle age (range 21–82 years), and most were Caucasian and female. | (58) |
| **TAS2R4** | rs2233998 | Candidate | Responsiveness to a 3.2-mM solution of PROP (MP Biomedicals, OH, USA) was measured using a generalized labeled magnitude scale (gLMS), employing the method described in Bajec and Pickering.. PTS categories were defined based on the cutoff values used by Porubcan and Vickers (2005) and Bajec and Pickering (2010): pNTs, <10.9 mm; pMTs, 10.9–61.5 mm; pSTs, >61.5 mm. | Fifty-three participants were recruited from the students, staff, and faculty of Brock University, and comprised of 36 females and 17 males with a mean age of 26.5 years ±9.7 SD, all of whom were nonsmokers. | (62) |
| **TAS2R4** | rs2234002 | Candidate | Labeled Magnitude Scale (gLMS) followed by a short practice session. Participants also rated the perceived intensity of five different prototypical tastants presented regionally to each quadrant of the tongue. Participants also rated the perceived intensity of a chemesthetic stimulus (either capsaicin, ethanol or piperine) applied to the left and right circumvallate (CV) papillae, which are located on the posterior tongue, forming a rearward pointing chevron of 8 to 12 dome-shaped structures. Detection thresholds were collected for chemesthetic stimuli using a forced choice method based on ASTM method E-679. As the final stimulus within a session, participants rated the overall intensity of a whole mouth swish-and-spit chemesthetic stimulus | Individuals were recruited from the Pennsylvania State University campus and surrounding community (State College, PA) as part of a larger study on the genetics of oral sensation. All four sessions were completed by 106 participants (40 men) with a mean age of 25.2± 0.63 (SEM). Participants reported ethnicity using the recommended wording from the 1997 OMB Directive 15 guidelines. The majority of participants reported Caucasian ancestry (n=69), followed by Asian (n=15) and African American (n=1); 11 participants chose not to disclose their ancestry. Prior to study enrollment, individuals interested in participating completed an online screening questionnaire. Eligibility criteria included: between 18–45 years old, not pregnant nor breastfeeding, had not smoked in the last 30 days, no known defects of smell nor taste, no oral piercings (lip, cheek or tongue), no history of chronic pain, not currently taking any prescription pain medication, no history of choking or difficulty swallowing and no history of thyroid disease. | (64) |
| **TAS2R60** | rs4595035 | Candidate | Bitterness of quinine was rated. | A total of 600 subjects were enrolled in this study, but 28 were eliminated from the analysis because they (or their co-twin) failed to follow instructions or had a history of alcohol dependence. The remaining 572 subjects were, on average, middle age (range 21–82 years), and most were Caucasian and female. | (58) |
| **TRPA1** | rs7827617 | Candidate | Bitterness of quinine was rated. | A total of 600 subjects were enrolled in this study, but 28 were eliminated from the analysis because they (or their co-twin) failed to follow instructions or had a history of alcohol dependence. The remaining 572 subjects were, on average, middle age (range 21–82 years), and most were Caucasian and female. | (58) |
| **TRPA1** | rs11988795 | Candidate | Bitterness of quinine was rated. | A total of 600 subjects were enrolled in this study, but 28 were eliminated from the analysis because they (or their co-twin) failed to follow instructions or had a history of alcohol dependence. The remaining 572 subjects were, on average, middle age (range 21–82 years), and most were Caucasian and female. | (58) |
| **TRPM5** | rs2301699 | Candidate | Bitterness of quinine was rated. | A total of 600 subjects were enrolled in this study, but 28 were eliminated from the analysis because they (or their co-twin) failed to follow instructions or had a history of alcohol dependence. The remaining 572 subjects were, on average, middle age (range 21–82 years), and most were Caucasian and female. | (58) |
| **TRPM8** | rs7593557 | Candidate | Bitterness of quinine was rated. | A total of 600 subjects were enrolled in this study, but 28 were eliminated from the analysis because they (or their co-twin) failed to follow instructions or had a history of alcohol dependence. The remaining 572 subjects were, on average, middle age (range 21–82 years), and most were Caucasian and female. | (58) |
| **TRPV1** | rs4790522 | Candidate | Bitterness of quinine was rated. | A total of 600 subjects were enrolled in this study, but 28 were eliminated from the analysis because they (or their co-twin) failed to follow instructions or had a history of alcohol dependence. The remaining 572 subjects were, on average, middle age (range 21–82 years), and most were Caucasian and female. | (58) |
| **TRPV1** | rs8065080 | Candidate | Bitterness of quinine was rated. | A total of 600 subjects were enrolled in this study, but 28 were eliminated from the analysis because they (or their co-twin) failed to follow instructions or had a history of alcohol dependence. The remaining 572 subjects were, on average, middle age (range 21–82 years), and most were Caucasian and female. | (58) |

**Supplementary Table 5 Study characteristics of genetic association studies related to sweet taste preferences**

FFQ: food frequency questionnaire, iAUC: incremental area under the curve, BMI: body mass index, AUC: area under the curve, AceK: Acesulfame Potassium; FP: fungiform papillae

**Supplementary Table 5 Study characteristics of genetic association studies related to sweet taste preferences**

| **Gene** | **SNP** | **Discovery method** | **Phenotype assessment method** | **Study population characteristics** | **Findings** | **Reference** | **Study characteristics (no association)** | **Reference (no association)** |
| --- | --- | --- | --- | --- | --- | --- | --- | --- |
| **TAS1R2** | rs3935570 | Candidate | All subjects completed a 1-month, 196-item semiquantitative food frequency questionnaire, which has previously been described. Subjects indicated how many times in the past month they consumed a specified portion of each food or beverage, and responses were converted to average daily intake for each item. The average daily intakes of all items were analyzed to compute a total daily intake of all major macroand micronutrients for each subject. Detection thresholds for sucrose were assessed using a three-alternative forced choice up down method. | Subjects were women (n = 524) and men (n = 251) aged 20–29 years from the Toronto Nutrigenomicsand Health Study, a cross-sectional study investigating the effects of gene-diet interactions on biomarkers of chronic disease and genetic determinants of food preferences and intake in a population of young adults. Subjects were excluded from the study if they were pregnant or breastfeeding. Of these individuals, 28 men and 67 women aged (mean ± SE) 23.7 ± 0.6 years were recruited to take part in a sensory test. Individuals who were smokers, had experienced marked weight changes in the last year (>15 pounds), were diagnosed with chronic sinusitis or chronic obstructive bowel disease, had lost their sense of smell, often experienced severe dry mouth, were diagnosed with diabetes or any other chronic disease or were diagnosed with a psychological disorder were excluded from the study BMI <25 BMI ≥25 p  Subjects 542 171  Female 386 (71) 94 (55) <0.0001  Age, years 23.1±0.1 23.6±0.2 0.04  BMI 21.8±0.1 28.2±0.2 | GG or GT vs. TT had significantly higher detection thresholds (and lower suprathreshold sensitivity ratings (iAUC)) but only in individuals with BMI ≥ 25. (No effect on sugar consumption.) | (65) | - | - |
| **TAS1R2** | rs12033832 | Candidate | All subjects completed a 1-month, 196-item semiquantitative food frequency questionnaire, which has previously been described. Subjects indicated how many times in the past month they consumed a specified portion of each food or beverage, and responses were converted to average daily intake for each item. The average daily intakes of all items were analyzed to compute a total daily intake of all major macroand micronutrients for each subject. Detection thresholds for sucrose were assessed using a three-alternative forced choice up down method. | Subjects were women (n = 524) and men (n = 251) aged 20–29 years from the Toronto Nutrigenomicsand Health Study, a cross-sectional study investigating the effects of gene-diet interactions on biomarkers of chronic disease and genetic determinants of food preferences and intake in a population of young adults. Subjects were excluded from the study if they were pregnant or breastfeeding. Of these individuals, 28 men and 67 women aged (mean ± SE) 23.7 ± 0.6 years were recruited to take part in a sensory test. Individuals who were smokers, had experienced marked weight changes in the last year (>15 pounds), were diagnosed with chronic sinusitis or chronic obstructive bowel disease, had lost their sense of smell, often experienced severe dry mouth, were diagnosed with diabetes or any other chronic disease or were diagnosed with a psychological disorder were excluded from the study BMI <25 BMI ≥25 p  Subjects 542 171  Female 386 (71) 94 (55) <0.0001  Age, years 23.1±0.1 23.6±0.2 0.04  BMI 21.8±0.1 28.2±0.2 | Individuals with a BMI ≥ 25: G allele carriers had significantly higher detection and lower suprathreshold sensitivity ratings (iAUC), higher intake of total sugars, sucrose, fructose and glucose.  Individuals with a BMI <25: significantly lower detection thresholds and no effect on suprathreshold taste, lower intake of total sugars, sucrose, fructose, glucose and lactose. | (65) | Our test population consisted of 144 unrelated individuals, who identified themselves as European (n = 92), Asian (n = 37), or African (n = 15). | (66) |
|  |  | Candidate | Subject eating behaviour characteristics were measured by the Three Factor Eating Questionnaire. A modified method from Stewart et al. was used to screen participants for their sweet taste sensitivity. In brief, a triplicate triangle test was performed whereby the subject was presented with three samples (10-ml each) per set: two samples with pure water and one sucrose solution at 9 mM (CSR pure icing sugar, Colonial Sugar Refining Company, Australia; purchased at the local Coles Supermarket). According to previous literature on sucrose detection threshold in young people, and preliminary results from our group, 9 mM was chosen for sucrose concentration where 48 % of the participants could detect the tastant at or below this concentration. Participants were asked not to drink or eat (except water) 2 h before the test. |  |  |  | In all, thirty-two adult volunteers were recruited from students and staff of the University of Queensland. Initial exclusion criteria included: history of taste or olfactory dysfunction; smoker; vegetarian; food allergy; drug or medication use; and pregnancy. One participant dropped out after the first preload study session due to lack of appetite in the morning, and one participant did not finished all the sessions due to personal reason. In all, thirty participants (fourteen females, age range 20–37 years; sixteen males, age range 24–34 years) completed the whole study and their data were analysed. | (67) |
| **TAS1R2** | rs35874116 | Candidate | Subject eating behaviour characteristics were measured by the Three Factor Eating Questionnaire. A modified method from Stewart et al. was used to screen participants for their sweet taste sensitivity. In brief, a triplicate triangle test was performed whereby the subject was presented with three samples (10-ml each) per set: two samples with pure water and one sucrose solution at 9 mM (CSR pure icing sugar, Colonial Sugar Refining Company, Australia; purchased at the local Coles Supermarket). According to previous literature on sucrose detection threshold in young people, and preliminary results from our group, 9 mM was chosen for sucrose concentration where 48 % of the participants could detect the tastant at or below this concentration. Participants were asked not to drink or eat (except water) 2 h before the test. During the test, | In all, thirty-two adult volunteers were recruited from students and staff of the University of Queensland. Initial exclusion criteria included: history of taste or olfactory dysfunction; smoker; vegetarian; food allergy; drug or medication use; and pregnancy. One participant dropped out after the first preload study session due to lack of appetite in the morning, and one participant did not finished all the sessions due to personal reason. In all, thirty participants (fourteen females, age range 20–37 years; sixteen males, age range 24–34 years) completed the whole study and their data were analysed. | CC and CT vs. TT associated with higher intake of sweet foods. | (67) | - | - |
|  |  |  | Two different methods of dietary assessment were used to assess habitual intake of food and beverages. A food-frequency questionnaire (FFQ) was used in population 1, and 2 sets of 3-d food records were used in population 2. The self-administered FFQ used in population 1 was the Toronto-modified Willett questionnaire. The FFQ consisted of 184 food and beverage items and 12 vitamin and dietary supplement items. In addition to total sugars (defined as mono- and disaccharides), the nutrient database also provided information on intakes of sucrose, maltose, lactose, fructose, and glucose. To examine the type of foods consumed that contributed to the intake of sugars, the consumption of daily servings from specific food groups that contained sugars were compared. Each food item response was first converted into daily servings and subsequently summed within its respective food group. Total fruit, which included fruit juice and fruit, and dairy products, corresponded to the original sections of food groups in the FFQ with minor modifications such as excluding avocado and nondairy coffee whitener, respectively, to reflect sugar sources. Sweets included chocolates, candy, jams, baked goods, and ice cream. Sweetened beverages included regular soft drinks, fruit drinks (not fruit juice), and sport drinks. For population 2, each subject was instructed on how to complete a 3-d food record, including 2 weekdays and 1 weekend day, on 2 separate visits. | Population 1 Subjects were participants from the Toronto Nutrigenomics and Health Study, which is a cross-sectional study examining the role of genetics in food intake as well as gene-diet interactions on biomarkers of chronic disease in young men and women between 20 and 29 y of age. Since October 2004, men (n = 391) and women (n = 886) with an average BMI of 23.0 6 6.7 (mean 6 SD) were recruited from the University of Toronto campus. Women who were pregnant or breastfeeding were excluded from the study. For the current analyses, we also excluded subjects who may have underreported (,800 kcal/d) or overreported (.3500 kcal/d for women, .4000 kcal/d for men) their energy intakes (n = 100) or reported consuming a special diet that restricted carbohydrates, fat, or calories (n = 54). We also excluded smokers (n = 79), one subject who had type 1 diabetes, and individuals who had missing data on potential confounders (n = 6). The final sample size consisted of 309 men and 728 women. Subjects were classified by self-reported ethnocultural ancestry and were grouped as white (n = 482), East Asian (n = 362), South Asian (n = 114), or other (n = 79). Population 2 The second population consisted of participants from the Canadian Trial of Carbohydrates in Diabetes multicenter intervention study, described in detail elsewhere (25). Subjects were recruited from 5 centers across Canada (in Edmonton, London, Toronto, Montreal, and Sherbrooke) over a 1-y period (2002–2003), and the baseline data collected was used for this study. All subjects were diagnosed with type 2 diabetes according to the Canadian Diabetes Association (CDA) criteria. Subjects included men and women who had early type 2 diabetes with near-normal glycated hemoglobin (6.2 6 0.6%) and who were considered not to require medications. Therefore, subjects were instructed to follow the CDA dietary guidelines and were excluded if they were using any hypoglycemic, antihyperglycemic, or oral steroid drugs or experienced a major cardiovascular event or surgery in the past 6 mo. Of the 166 subjects recruited, 127 subjects agreed to give a blood sample for genotype analysis, one subject’s genotype remained undetermined, 17 subjects had incomplete baseline dietary data, and 9 individuals had missing data on potential confounders, which left 100 subjects for the final analyses. According to 2 sets of 3-d food records, all subjects reported consuming between 800 and 3500 kcal/d for women and 800 to 4000 kcal/d for men, and therefore, no exclusions were made for possible under- or overreporting. The study consisted of men (n = 49) and women (n = 51) between the ages of 42 and 75 y with a mean (6SD) BMI of 30.6 6 4.2. | Overweight Val carriers consumed less sugars, sucrose, fructose and glucose than Ile homozygotes. | (68) |  |  |
| **TAS1R3** | rs307355 | Candidate | The study analyzed threshold and suprathreshold sensitivity to sucrose by employing the novel use of signal detection analysis and R-index measures for this trait. The quantitative stimuli consisted of 9 different blinded sucrose solutions, which subjects sorted from least sweet to most sweet. For each pairwise sucrose concentration (e.g. 0−0.5%, 0.5−1%, etc) we estimated the classical signal detection measure of sensitivity P(A) - the area under a receiver operating characteristic (ROC) curve - by calculating the R-index . The area under an ROC curve, and hence the R-index, ranges from 0.5 (chance level discrimination) to 1 (perfect discrimination) and measures an individual's ability to discriminate between two stimuli. To obtain a singular measure of sensitivity across the entire sucrose concentration series, the pairwise R-indices were summed to create a concentration-response function for each subject and the area under this curve (AUC) was determined. The AUC, which can theoretically range from 0 (complete non-discrimination) to 9.25 (perfect discrimination) in this testing paradigm, was used as the dependent variable for assessing the effect of genotype on sucrose sensitivity. | Our test population consisted of 144 unrelated individuals, who identified themselves as European (n = 92), Asian (n = 37), or African (n = 15) | Strong association with decreased sucrose AUC scores (reduced taste sensitivity to sucrose associated with T alleles) | (66) | In all, thirty-two adult volunteers were recruited from students and staff of the University of Queensland. Initial exclusion criteria included: history of taste or olfactory dysfunction; smoker; vegetarian; food allergy; drug or medication use; and pregnancy. One participant dropped out after the first preload study session due to lack of appetite in the morning, and one participant did not finished all the sessions due to personal reason. In all, thirty participants (fourteen females, age range 20–37 years; sixteen males, age range 24–34 years) completed the whole study and their data were analysed. | (67) |
| **TAS1R3** | rs35744813 | Candidate | The study analyzed threshold and suprathreshold sensitivity to sucrose by employing the novel use of signal detection analysis and R-index measures for this trait. The quantitative stimuli consisted of 9 different blinded sucrose solutions, which subjects sorted from least sweet to most sweet. For each pairwise sucrose concentration (e.g. 0−0.5%, 0.5−1%, etc) we estimated the classical signal detection measure of sensitivity P(A) - the area under a receiver operating characteristic (ROC) curve - by calculating the R-index . The area under an ROC curve, and hence the R-index, ranges from 0.5 (chance level discrimination) to 1 (perfect discrimination) and measures an individual's ability to discriminate between two stimuli. To obtain a singular measure of sensitivity across the entire sucrose concentration series, the pairwise R-indices were summed to create a concentration-response function for each subject and the area under this curve (AUC) was determined. The AUC, which can theoretically range from 0 (complete non-discrimination) to 9.25 (perfect discrimination) in this testing paradigm, was used as the dependent variable for assessing the effect of genotype on sucrose sensitivity. | Our test population consisted of 144 unrelated individuals, who identified themselves as European (n = 92), Asian (n = 37), or African (n = 15) | Strong association with decreased sucrose AUC scores (reduced taste sensitivity to sucrose associated with T alleles). | (66) | Mothers of healthy children 7–14 years of age were recruited for a “taste study” from local advertisements and from a list of past participants who asked to be notified of future studies Only children who were healthy at the time of testing, with no major medical illness such as diabetes, heart disease, or asthma, were included. the study population consisted of 235 children (female 124, race balck 136, white 46, Asian 2, other/more than one 51; ethnicity (non-Hispanic 219) . For a subset of the subjects tested (n = 96; (female 53, race balck 49, white 16, Asian 2, other/more than one 29; ethnicity (non-Hispanic 84), we obtained additional anthropometric measures and dietary intake data. Detection thresholds were measured by using a two-alternative, forced-choice staircase procedure. | (60) |
|  |  | Candidate |  | The sample included 108 healthy children 5–10 years of age (61 singletons, 19 sibling pairs, 3 sibling triads) and their mothers (n = 83), none of whom were taking prescription medications (except for birth control pills among women). Mothers were queried about race/ethnicity of themselves and their children, highest education level and family yearly income. Seven children did not understand the psychophysical tasks or did not comply with study procedures, and seven mothers did not undergo psychophysical testing because they did not meet the inclusion criteria, thus resulting in a final participant sample of 101 children and 76 adults. Not all subjects completed all tasks: most (95 children, 74 adults) provided complete dietary data; 99 children agreed to provide urine samples, and 80 provided samples that yielded valid NTx data (the other samples either were inadequate or yielded out-of-range values). Genotype: 72 children, 72 mothers. Mothers n=76, Age, years [mean (SEM)] 36.1 (1.0); Race/ethnicity [% (n)]  White 32.9% (25); Black 52.6% (40); Hispanic/Latino/Latina 5.3% (4); Asian 1.3% (1); Other/more than one race 7.9% (6). Children n=101, Age, years [mean (SEM)] 7.8 (0.2);  Race/ethnicity [% (n)]  White 31.7% (32);  Black 42.6% (43);  Hispanic/Latino/Latina 8.9% (9);  Asian 2.0% (2);  Other/more than one race 14.9% (15). | Adults with no T alleles preferred a lower concentration of sucrose than did those with one or two T alleles (no association in children). | (69) | In all, thirty-two adult volunteers were recruited from students and staff of the University of Queensland. Initial exclusion criteria included: history of taste or olfactory dysfunction; smoker; vegetarian; food allergy; drug or medication use; and pregnancy. One participant dropped out after the first preload study session due to lack of appetite in the morning, and one participant did not finished all the sessions due to personal reason. In all, thirty participants (fourteen females, age range 20–37 years; sixteen males, age range 24–34 years) completed the whole study and their data were analysed. | (67) |
| **TAS2R38** | rs713598 | Candidate | Parents completed three-day, weighed dietary records on three days (1 weekend day and 2 week days) at the ages of 12, 24, 36, 48, 60 and 72 months. For custom food items, information on nutrient content was provided from manufacturers. Mono- and disaccharides were considered as sugars. If data on sugar content was not available it was estimated based on comparison with similar products. Food items were categorized into subgroups according to food composition and taste and divided into sweet and non-sweet tasting. Sweet tasting foods comprise pastry products, sweet tasting beverages (fruit juice, soft drinks, fruit drinks), sweetened cereals (sugar >10 g/100 g), desserts, fruit and fruit products, sweetened dairy products, sweets, instant cacao powder, sugar and honey and sweet main dishes.  Fruit and fruit products contain fresh and dried fruits as well as fruit mash or preserves and jam. For further analysis sweet tasting food items were divided into sweet products with high energy density (energy density 200 kcal/100 g) and sweet products with low energy density (energy density < 200 kcal/100 g). | Study of children from five countries across EuropeTAS2R38 genotype and dietary data for at least one time point between 12 and 72 months was available in 691 children (444 or 64.3% genotype PP or PA, 247 or 35.7% genotype AA). (Eligible for study participation were apparently healthy, singleton, term infants who were born between 1 October 2002 and 31 July 2004. Children of mothers with a hormonal or metabolic disease or illicit drug addiction during pregnancy were not included. Children were recruited in 5 countries (Belgium, Germany, Italy, Poland, and Spain). Anthropometric measurements were made at 11 sites: 2 in Germany (Munich and Nuremberg), 2 in Belgium (Liege and Brussels), 4 in Italy (Milano), 1 in Poland (Warsaw), and 2 in Spain (Reus and Tarragona). Subjects with pregnancies involving gestational diabetes, a known familial history of metabolic or hormonal diseases, or any disease interfering with metabolism or growth of the child were excluded. | The PP/PA genotype was associated with a higher intake of (energy dense) sweet tasting foods in children. | (70) | - | - |
|  |  | Candidate | Test-meal procedures. Items served at the test-meal were selected because they were highly palatable and familiar to most children this age. Prior to the experiment, the foods were divided into three categories: sweets (e.g. red licorice, gummies, and sweetened beverages), sweet-fats (e.g. cookies, brownies, doughnuts), and savory-fats (e.g. pizza, mozzarella sticks, chips). Foods in the savory-fat or sweet-fat category had either savory or sweet as their predominant flavor characteristic and contained 20% calories from fat or greater. Items in the sweet food category were primarily sweet tasting and contained less than 1 g of fat per serving. | Children (n = 79) enrolled in this study were between 4 and 6 years old (mean ± SD = 5.04 ± 0.78). Parents self-reported the ethnicity of their children as African-American (42.5%), Hispanic/Latino (31.3%), Caucasian (12.5%), Asian (2.5%) or “other” (11.3%). Approximately 40% of the children were boys. Average BMI z-score for children was 1.00 ± 1.02, corresponding to the 85th BMI-forage percentile. TAS2R38 genotype was unable to be determined for three children due to failures of the test kits or inadequate saliva samples for a failure rate of 4%. Genotype at rs713598 is reported for 76 children. | AP or PP children consumed more chocolate chip cookies at the test-meal than children who had the AA genotype. | (9) | - | - |
| **TAS2R38** | rs713598 | Candidate | Sucrose preferences were determined using a forced-choice, paired-comparison tracking procedure that is sensitive to the cognitive limitations of children. Participans were presented with pairs of solutions of sucrose at differing concentrations (3, 6, 12, 24, and 36 g/dL). They tasted and expectorated each solution and pointed to the one they liked better. The procedure continued until the participant either chose the solution with a given concentration when it was paired with both a higher and a lower concentration or chose the solution with the highest or lowest concentration 2 consecutive times. Children were also queried about their favorite beverages. Specifically, they were asked, “What is your favorite beverage in the whole world?” and “Which beverage do you ask your mom to buy the most?” The sugar content of their favorite beverages was determined from product labels. | The study population consisted of healthy, 3- to 10-year-old children and their mothers who participated in 1 of 5 taste-research studies at the Monell Chemical Senses Center (Philadelphia, Pennsylvania) between 2003 and 2010. Participants were pooled from multiple studies to provide enough power for genotype–phenotype analysis. The total study population included healthy children between the ages of 3 and 19 years; however, for the present analysis, children who were 3 to 10 years of age were selected because age is a key factor in the ability of a child to swallow a pill (the majority of children over the age of 10 years are easily able to swallow a pill). Genotype–phenotype association studies require that subjects be genetically unrelated, so if >1 child from a family was tested, data were included from the first child of the sibling pair from whom data were available. None of the children were excluded from analyses for any other reasons. All of the children underwent genotyping. The sample consisted of 448 children (245 girls, 203 boys; mean [SE] age, 7.8 [0.1] years; black, 52.2%; white, 28.1%; other race/ethnicity, 19.6%). Because of differences among studies, 256 children (57%) underwent both phenotyping for sucrose preference and were queried about medication usage, 111 (25%) underwent phenotyping only, and 81 (18%) provided only retrospective, self-reports of solid medication usage. | PP children preferred higher concentrations of sucrose in water and beverages containing more sugar than AA children (AP intermediate preference). | (71) | Random convenience sampling was used in this study. Booths were set up at Universiti Tunku Abdul Rahman campuses, a primary and a secondary school, around Klang Valley, from October to December, 2008. The ranges of age, sex and race of the samples were not limited. A short introduction of this study was given to subjects who passed by the booth. Subjects were categorized into 3 major races, Malays, Chinese and Indians. Among 215 subjects in this study, the males and females  were almost equally balanced with males comprising 46.5%. The subjects ranged from 10 to 76 years old with the mean age of 21.3 ± 10.4 years old. Nearly half of the total subjects were Chinese (48.9%), followed by Malays (27.5%) and Indians (22.7%). Male (n=100) Female (n=115)  Age (mean±SD); years 21.2±12.4 21.4±8.4. Subjects were asked to indicate how much they liked or disliked a list of 36 mostly local Asian vegetables, 4 soy products and 37 sweet or fat foods, using a ten-point hedonic preference scale which ranged from one (extremely like) to ten (extremely dislike), with a neutral point at five (neither like nor dislike). Subjects were also asked whether they liked to drink green tea or not. The types of vegetables in this study can be divided into 2 categories: 25 bitter vegetables and 11 sweet vegetables. | (12) |
|  |  | Candidate | Participants were administered a food preference questionnaire, proposed by Catanzaro (Catanzaro et al. 2013), consisting of 30 items structured on five-point scale (2=“I hate it very much”; 1 “I hate it”; 0 “indifferent”; 1 =“I like it” 2 =I like it very much”). Food preferences were chosen to provide a comprehensive but non-redundant coverage of the Italian diet. A dietician submitted the questionnaire after taste assessment, and participants were asked not to discuss their food preferences while completing the questionnaire. | This was a two-centre cross-sectional study. Adults aged 18–65 years, with body mass index (BMI) 15–35 kg/m2), who were admitted to the Azienda di Servizi alla Persona di Pavia, University of Pavia and Department of Biology, University of Pisa (Italy), were prospectively enrolled. All subjects had to give complete medical histories, and received a physical examination, with anthropometric assessment and routine laboratory tests. The study was conducted with the approval of the Ethics Committee of the Department of Internal Medicine and Medical Therapy, at the University of Pavia. The starting sample comprised 118 patients (24 men and 94 women). Their mean age was 45.28 ± 12.84 years, with BMI indicating slight overweight (27.38 ± 5.69 kg/m2). | GG subjects did not prefer sweet foods (dessert and chocolate). | (72) |  |  |
|  |  | Candidate | Detection thresholds were measured by using a two-alternative, forced-choice staircase procedure. | Mothers of healthy children 7–14 years of age were recruited for a “taste study” from local advertisements and from a list of past participants who asked to be notified of future studies Only children who were healthy at the time of testing, with no major medical illness such as diabetes, heart disease, or asthma, were included. the study population consisted of 235 children (female 124, race balck 136, white 46, Asian 2, other/more than one 51; ethnicity (non-Hispanic 219) . For a subset of the subjects tested (n = 96; (female 53, race balck 49, white 16, Asian 2, other/more than one 29; ethnicity (non-Hispanic 84), we obtained additional anthropometric measures and dietary intake data. | P allele more common in children with lower sucrose thresholds. | (60) |  |  |
| **TAS2R38** | rs1726866 | Candidate | Detection thresholds were measured by using a two-alternative, forced-choice staircase procedure. | Mothers of healthy children 7–14 years of age were recruited for a “taste study” from local advertisements and from a list of past participants who asked to be notified of future studies Only children who were healthy at the time of testing, with no major medical illness such as diabetes, heart disease, or asthma, were included. the study population consisted of 235 children (female 124, race balck 136, white 46, Asian 2, other/more than one 51; ethnicity (non-Hispanic 219) . For a subset of the subjects tested (n = 96; (female 53, race balck 49, white 16, Asian 2, other/more than one 29; ethnicity (non-Hispanic 84), we obtained additional anthropometric measures and dietary intake data. | Children with one or two bitter-sensitive A alleles had lower detection thresholds (more sensitive to the taste of sucrose). | (60) | Eligibility criteria included the following: between 18 and 45 years old; not pregnant or breastfeeding; nonsmoker (had not smoked in the last 30 days); no known defects of smell or taste; no lip, cheek, or tongue piercings; no history Bitterness of Acesulfame Potassium Varies With TAS2R Polymorphisms 381 of any condition involving chronic pain; not currently taking any prescription pain medication; no reported history of choking or difficulty swallowing; and no history of thyroid disease. Participants also needed to be willing to provide a DNA sample via saliva. DNA samples were available from 147 participants. Race and ethnicity was self-reported using categories provided by the 1997 OMB Directive 15. To minimize potential population stratification, which can potentially cause false negatives and false positives in gene association studies (Hamer and Sirota 2000), individuals with Asian (n = 18), African (n = 5), or unknown (n = 15) ancestry were excluded from the present analyses. Thus, we report data from 108 participants (34 men) of European ancestry, with a mean age of 27.4 (±8.1 SD) years. Results were not substantively different in the mixed ancestry sample, but we report only the results for the European–American participants to facilitate interpretation of the linkage disequilibrium (LD) plots. A general labeled magnitude scale (gLMS) was used to collect perceived intensity of suprathreshold stimuli. This scale ranges from 0 (“no sensation”) to 100 (“the strongest imaginable sensation of any kind”), with intermediate descriptors at 1.4 (“barely detectable”), 6 (“weak”), 17 (“moderate”), 35 (“strong”), and 51 (“very strong”). | (8) (AceK sweetness) |
| **TAS2R38** | rs10246939 | Candidate | Detection thresholds were measured by using a two-alternative, forced-choice staircase procedure. | Mothers of healthy children 7–14 years of age were recruited for a “taste study” from local advertisements and from a list of past participants who asked to be notified of future studies Only children who were healthy at the time of testing, with no major medical illness such as diabetes, heart disease, or asthma, were included. the study population consisted of 235 children (female 124, race balck 136, white 46, Asian 2, other/more than one 51; ethnicity (non-Hispanic 219) . For a subset of the subjects tested (n = 96; (female 53, race balck 49, white 16, Asian 2, other/more than one 29; ethnicity (non-Hispanic 84), we obtained additional anthropometric measures and dietary intake data. | Children with one or two bitter-sensitive V alleles had lower detection thresholds (more sensitive to the taste of sucrose). | (60) | - | - |
| **TAS2R38** | A49P (rs713598), A262V (rs1726866), V296I (rs10246939) | Candidate | The children evaluated the pleasantness of berry samples in each product and rated their liking score u. The verbal descriptors of P&K-scale were translated into the Finnish language. The scale is anchored to a 9-point scale from super bad (1) to super good (9). | The sensory study included 104 healthy children, recruited from five municipal day-care centres and three schools in the area of the city of Turku, Finland. The day-care centres and schools chosen to this study represented various neighbourhoods in Turku. Fifty children from day-care centres and 54 children from schools participated in sensory testing. Genotyping of subjects was performed 1 year after the sensory study, and 73 out of the 104 subjects who participated in the sensory study also gave a sample for the hTAS2R38 gene testing. 39 of the children were from schools and 34 from day-care centres. Before the sensory study, parents filled a background information questionnaire about the health status and dietary habits of the children, which included the usage of vegetables, fruits, and berries. Exclusion criteria for their participation were diabetes, coeliac disease, allergies to milk, cereal, or berry products, and lactose intolerance. Only genotyped subjects that rated all of the berry products were selected to this part (n = 51). At the time of the sensory evaluation, the average age of the children at day-care centres was 5.5 ± 0.7 (mean ± SD) years and 9.5 ± 0.3 years for the school children. The test group was ethnically quite homogenous, consisting of children of mostly Finnish ancestry and of a few immigrants only. | Majority of PAV/PAV and PAV/AVI children, liked the sweetened, dried bilberries with rather high sugar content. | (73) | - | - |
| **TAS2R38** | A49P (rs713598), A262V (rs1726866), V296I (rs10246939) | Candidate | Food consumption was assessed using a modified 131-item food frequency questionnaire (FFQ) developed and validated  by the Finnish National Institute for Health and Welfare. Using nine response categories that ranged from never to [6 portions/day, the participants were asked to report their habitual consumption of selected foods and dishes during the previous 12 months. | The Cardiovascular Risk in Young Finns Study The YFS is a population-based follow-up study of the development of cardiovascular and other metabolic disorders in five study centers in Finland (Turku, Helsinki, Tampere, Kuopio and Oulu). The first cross-sectional survey was conducted in 1980, and 3,596 children and adolescents aged 3, 6, 9, 12, 15 and 18 years were examined according to a standardized protocol. The present study is based on the data obtained at the follow-up study in 2007 when the subjects were 30–45 years of age. All subjects who participated in 1980 and still had a permanent address in Finland were invited, and 2,247 (63 %) participated. The subjects’ weights and heights were measured at the study visit, and the background characteristics were collected by questionnaire. The dietary information from the 2012 subjects was assessed. Questionnaires that were incompletely filled out or were unreliable (n = 16) were excluded, which led to the inclusion of 1,996 subjects. Genotyping was completed for 2,557 subjects. In total, the association analyses between the haplotypes and dietary intakes were carried out for 1,903 subjects, of which 1,055 (55 %) were women. | PAV homozygotic individuals consumed more sweet foods than did the AVI homozygotic subjects. | (74) | - | - |

| **ADIPOQ** | rs822396 | GWAS | We defined the intake score for confectionery as described below, and considered it as a trait in the current GWAS to seek relevant quantitative trait loci. Participants in both stages 1 and 2 studies were asked to report their usual frequency of consumption of 43 food items in a self-administered questionnaire with the following eight possible responses: 1 = almost never; 2 = 1-3 times per month; 3= 1-2 times per week; 4 =3-4 times per week; 5 =5-6 times per week; 6 = once per day; 7 = twice per day; and 8 => 3 times per day. The respondents were requested to circle one of the numbers to provide an answer. Western-style and Japanesestyle confectionery were included as two separate food items in the questionnaire. The responses were then converted into intake scores of 0, 0.1, 0.2, 0.5, 0.8, 1, 2, and 3, respectively, and the sum of the two intake scores was used for association analysis. | In the discovery phase (stage 1), we conducted a GWAS of 977 participants of the Hospital-based Epidemiological Research Program II at Aichi Cancer Center Hospital (HERPACC-II) between January 2001 and September 2005. All participants were enrolled during their first visit to the Aichi Cancer Center Hospital (ACCH; Nagoya, Japan). Briefly, all first-visit outpatients to the ACCH aged 20-79 years were asked to fill in a self-administered questionnaire about their lifestyle and medical factors, and trained interviewers checked their responses. The outpatients were also asked to provide a blood sample. In total, 96.7% of contacted patients completed the questionnaire and about 50% of respondents provided a blood sample. The current analyses were limited to noncancer participants; approximately 35% of the subjects were diagnosed with cancer within 1 year of their first visit. Our previous study showed that the lifestyle patterns of first-visit outpatients without cancer corresponded well with those of individuals who were randomly selected from the general population of Nagoya city. Association signals selected in the stage 1 GWAS were followed-up by genotyping the SNPs in 4,491 participants aged 35-69 years in a cross-sectional study within the Japan Multi-Institutional Collaborative Cohort (J-MICC) Study (replication phase [stage 2]). We previously reported the detailed design of this cross-sectional study and the J-MICC Study as a whole. In brief, participants in the current study completed a questionnaire about lifestyle and medical factors, and donated a blood sample at the time of the J-MICC Study baseline survey. J-MICC Study participants were recruited from 10 areas throughout Japan between 2004 and 2008, and included community citizens, first-visit patients to a cancer hospital, and health check-up examinees. The response rates for the baseline survey by study area varied according to the source population, and were recorded as 7.0-24.0% in the community (recruitment by mailing invitation letters or distributing leaflets), 58.4% in first-visit patients to a cancer hospital, and 14.0-65.5% in health check-up examinees. The respondents for the cross-sectional study comprised 400-600 participants who were enrolled consecutively from each area of the J-MICC Study, with the exception of two areas (Kyoto and Tokushima) where fewer participants were recruited. | Association with confectionery-intake score, but it did not reach genome-wide significance level (GWAS). | (75) | - | - |
| --- | --- | --- | --- | --- | --- | --- | --- | --- |
| **ANKK1** | rs1800497 | Candidate | Using 1 ml disposable syringes 8 samples of sucrose solutions were placed directly in the middle of the tongue. Then, subjects spread samples within their mouths and using VAS rated their intensity (from “0” = very weak to “100” = very strong) and pleasantness (from “-50” = very unpleasant to “50” = very pleasant). | A cohort of 62 adult men who fulfilled the ICD-10 alcohol dependence criteria was studied. All the subjects abstained from alcohol for a minimum of 7 days prior to the study. No withdrawal symptoms were observed. Patients diagnosed with mental diseases or addictions other than AD (apart from nicotine addiction) were excluded from the study. Individuals who within 30 days prior to the study suffered from psychotic disorders, smell and/or taste impairments, exacerbation of somatic disease that required a change of treatment or hospitalisation were also excluded. P | A1 alleles associated with sucrose preference. | (76) | This research protocol was approved by the Yale University Human Investigation Committee. Eighty-one subjects were recruited by advertisements posted around Yale University and the greater New Haven area. Only subjects between the age of 18 and 45 were included. Subjects were excluded if they had any nonremovable metal on their body, were currently or recently taking major medications such as antidepressants, were claustrophobic, had a history of food allergies, diabetes, or any psychiatric disorder or drug abuse. Prior to the experiment, subjects were given a description of the paradigm and provided written informed consent. All subjects completed an initial screening session. Thirty-four subjects were excluded after this session based on ratings falling outside of the target ranges. Those who qualified completed an additional screening and training session on that same day. Data from this session excluded another 12 subjects. Reasons for exclusion included ratings collected in the mock scanner not falling in the target range, discomfort with the scanning environment, or an unwillingness to consume sucrose repeatedly. the general labeled magnitude scale (gLMS) for pleasentness of sucrose. | (77) |
| **DRD2** | rs6277 | Candidate | To assess habitual dietary intake over the past month we used a 196-item self-administered food frequency questionnaire (FFQ) as previously described. The FFQ was modified from the Willett questionnaire, with the addition of 26 food items including 6 fruits, 6 cereals/breads, and 4 beverages. The final FFQ contained 184 food and beverage items and 12 vitamin and dietary supplement items. Additional prompts were added to the questionnaire to clarify beverage serving size, sugar content and whole grain content. | Health Study, which is a cross-sectional study examining the role of genetics in food intake and food selection as well as genediet interactions on biomarkers of chronic disease in young men and women between 20–29 years of age. Since October 2004, freeliving young men (n = 298) and women (n = 663) with an average BMI of 22.7 +/-3.5 (mean 8 SD) were recruited from the University of Toronto campus. Women who were pregnant or breastfeeding were excluded from the study. For the current analyses we also excluded subjects who may have underreported ( ! 800 kcal/ day) or overreported ( 1 3,500 kcal/day female, 1 4,000 kcal/day male) their energy intakes (n = 71) or reported following a special diet that restricted carbohydrates, fat, or calories (n = 46). Smokers (n = 60) and individuals reporting mood disorders or use of antidepressants and/or neuroleptic drugs (n = 94) were excluded from the analysis since these may affect dopaminergic circuits and food intake. One subject who had type 1 diabetes was also excluded. The final sample size consisted of 219 men and 470 women. Subjects were classified by self-reported ethnocultural ancestry and were grouped as Caucasian (n = 313), East Asian (n = 245), South Asian (n = 81), or other (n = 50). | Sucrose consumption was associated with genotypes (CC>CT>TT) among men. Consumption of total sugars, sucrose and fructose was associated with genotypes (CT>TT>CC) among women. | (78) | - | - |
| **FGF21** | rs838133 | Candidate | Profile of macronutrients was retreived from a validated semiquantitative 198-item FFQ (based on diet recall within the last month). | Genetic association studies were performed in the Danish population-based Inter99 study, which is a non-pharmacological intervention study for ischemic heart disease performed at the Research Centre for Prevention and Health, Glostrup, Denmark. A random sample of 13,016 individuals living in Copenhagen County from seven different age groups (30-60 years, grouped with five year intervals) was drawn from the Civil Registration System and 6,784 of these attended the health examination. All participants were Danes by self-report, and 6,514 individuals were eligible for genetic analyses. Subjects were between 18-39 years of age, with a BMI between 19-25 kg/m2 and without any medical conditions. A total of 86 subjects(23 men and 63 women) completed a questionnaire to determine taste preferences and each subject was asked to select images of liked and disliked snacks. | A-allele increased the odds ratio of the consumption of candy. | (79) | - | - |
| **GNAT3** | rs6467192 | Candidate | Measurements of sensitivity to sucrose were performed as described previously (Fushan et al. 2009). A series of preliminary trials empirically determined that solutions of 0, 0.5%, 1%, 2%, 2.4%, 2.8%, 3.2%, 3.6%, and 4% sucrose (Sigma, dissolved in deionized water) produced the best discrimination curves in a representative subpopulation of our subjects. Each concentration is used to calculate a detection threshold for a given sucrose interval (i.e., 0–0.5%, 0.5–1%, etc.). Each subject participated in 1 experiment that consisted of 6 replications performed over 3 sessions. Subjects were asked to complete 2 replications of the ranking test per session with a mandatory 5-min break between replications. Individual sessions were separated by at least a 24-h period. Subjects were presented with 20 ml of each of the solutions in randomized order and could ask for more at any time during the experiment. Panelists were asked to sample each of the solutions and rank them in order from least to most sweet. To minimize adaptation effects, subjects rinsed with water between each sample. Phenotype modeling Data from the 6 replications were pooled for each subject. For each pairwise sucrose concentration (0–0.5%, 0.5–1%, etc.), the R-index (hereafter referred to as Rp-index; see Supplementary Figure 1) was calculated as described in O’Mahony et al. (1992). | Participants were enrolled with written informed consent under National Institutes of Health Combined Neuroscience Institutional Review Board protocol 01-DC-0230 and contained individuals (69 males and 91 females) who identified themselves as Caucasian (n = 103), Asian (n = 41), or African–American (n = 16). African–American individuals had origins in the sub-Saharan racial groups of Africa | Correlation with sucrose AUC scores. | (51) | - | - |
| **GNAT3** | rs1524600 | Candidate | Measurements of sensitivity to sucrose were performed as described previously (Fushan et al. 2009). A series of preliminary trials empirically determined that solutions of 0, 0.5%, 1%, 2%, 2.4%, 2.8%, 3.2%, 3.6%, and 4% sucrose (Sigma, dissolved in deionized water) produced the best discrimination curves in a representative subpopulation of our subjects. Each concentration is used to calculate a detection threshold for a given sucrose interval (i.e., 0–0.5%, 0.5–1%, etc.). Each subject participated in 1 experiment that consisted of 6 replications performed over 3 sessions. Subjects were asked to complete 2 replications of the ranking test per session with a mandatory 5-min break between replications. Individual sessions were separated by at least a 24-h period. Subjects were presented with 20 ml of each of the solutions in randomized order and could ask for more at any time during the experiment. Panelists were asked to sample each of the solutions and rank them in order from least to most sweet. To minimize adaptation effects, subjects rinsed with water between each sample. Phenotype modeling Data from the 6 replications were pooled for each subject. For each pairwise sucrose concentration (0–0.5%, 0.5–1%, etc.), the R-index (hereafter referred to as Rp-index; see Supplementary Figure 1) was calculated as described in O’Mahony et al. (1992). | Participants were enrolled with written informed consent under National Institutes of Health Combined Neuroscience Institutional Review Board protocol 01-DC-0230 and contained individuals (69 males and 91 females) who identified themselves as Caucasian (n = 103), Asian (n = 41), or African–American (n = 16). African–American individuals had origins in the sub-Saharan racial groups of Africa | SNP correlated with sucrose AUC scores and haplotypes (combination of high-sensitivity alleles of rs7792845 and rs1524600), associated with higher sensitivity to sucrose. | (51) | - | - |
| **GNAT3** | rs6467217 | Candidate | Measurements of sensitivity to sucrose were performed as described previously (Fushan et al. 2009). A series of preliminary trials empirically determined that solutions of 0, 0.5%, 1%, 2%, 2.4%, 2.8%, 3.2%, 3.6%, and 4% sucrose (Sigma, dissolved in deionized water) produced the best discrimination curves in a representative subpopulation of our subjects. Each concentration is used to calculate a detection threshold for a given sucrose interval (i.e., 0–0.5%, 0.5–1%, etc.). Each subject participated in 1 experiment that consisted of 6 replications performed over 3 sessions. Subjects were asked to complete 2 replications of the ranking test per session with a mandatory 5-min break between replications. Individual sessions were separated by at least a 24-h period. Subjects were presented with 20 ml of each of the solutions in randomized order and could ask for more at any time during the experiment. Panelists were asked to sample each of the solutions and rank them in order from least to most sweet. To minimize adaptation effects, subjects rinsed with water between each sample. Phenotype modeling Data from the 6 replications were pooled for each subject. For each pairwise sucrose concentration (0–0.5%, 0.5–1%, etc.), the R-index (hereafter referred to as Rp-index; see Supplementary Figure 1) was calculated as described in O’Mahony et al. (1992). | Participants were enrolled with written informed consent under National Institutes of Health Combined Neuroscience Institutional Review Board protocol 01-DC-0230 and contained individuals (69 males and 91 females) who identified themselves as Caucasian (n = 103), Asian (n = 41), or African–American (n = 16). African–American individuals had origins in the sub-Saharan racial groups of Africa | Correlation with sucrose AUC scores. | (51) | - | - |
| **GNAT3** | rs6970109 | Candidate | Measurements of sensitivity to sucrose were performed as described previously (Fushan et al. 2009). A series of preliminary trials empirically determined that solutions of 0, 0.5%, 1%, 2%, 2.4%, 2.8%, 3.2%, 3.6%, and 4% sucrose (Sigma, dissolved in deionized water) produced the best discrimination curves in a representative subpopulation of our subjects. Each concentration is used to calculate a detection threshold for a given sucrose interval (i.e., 0–0.5%, 0.5–1%, etc.). Each subject participated in 1 experiment that consisted of 6 replications performed over 3 sessions. Subjects were asked to complete 2 replications of the ranking test per session with a mandatory 5-min break between replications. Individual sessions were separated by at least a 24-h period. Subjects were presented with 20 ml of each of the solutions in randomized order and could ask for more at any time during the experiment. Panelists were asked to sample each of the solutions and rank them in order from least to most sweet. To minimize adaptation effects, subjects rinsed with water between each sample. Phenotype modeling Data from the 6 replications were pooled for each subject. For each pairwise sucrose concentration (0–0.5%, 0.5–1%, etc.), the R-index (hereafter referred to as Rp-index; see Supplementary Figure 1) was calculated as described in O’Mahony et al. (1992). | Participants were enrolled with written informed consent under National Institutes of Health Combined Neuroscience Institutional Review Board protocol 01-DC-0230 and contained individuals (69 males and 91 females) who identified themselves as Caucasian (n = 103), Asian (n = 41), or African–American (n = 16). African–American individuals had origins in the sub-Saharan racial groups of Africa | Correlation with sucrose AUC scores. | (51) | - | - |
| **GNAT3** | rs6975345 | Candidate | Measurements of sensitivity to sucrose were performed as described previously (Fushan et al. 2009). A series of preliminary trials empirically determined that solutions of 0, 0.5%, 1%, 2%, 2.4%, 2.8%, 3.2%, 3.6%, and 4% sucrose (Sigma, dissolved in deionized water) produced the best discrimination curves in a representative subpopulation of our subjects. Each concentration is used to calculate a detection threshold for a given sucrose interval (i.e., 0–0.5%, 0.5–1%, etc.). Each subject participated in 1 experiment that consisted of 6 replications performed over 3 sessions. Subjects were asked to complete 2 replications of the ranking test per session with a mandatory 5-min break between replications. Individual sessions were separated by at least a 24-h period. Subjects were presented with 20 ml of each of the solutions in randomized order and could ask for more at any time during the experiment. Panelists were asked to sample each of the solutions and rank them in order from least to most sweet. To minimize adaptation effects, subjects rinsed with water between each sample. Phenotype modeling Data from the 6 replications were pooled for each subject. For each pairwise sucrose concentration (0–0.5%, 0.5–1%, etc.), the R-index (hereafter referred to as Rp-index; see Supplementary Figure 1) was calculated as described in O’Mahony et al. (1992). | Participants were enrolled with written informed consent under National Institutes of Health Combined Neuroscience Institutional Review Board protocol 01-DC-0230 and contained individuals (69 males and 91 females) who identified themselves as Caucasian (n = 103), Asian (n = 41), or African–American (n = 16). African–American individuals had origins in the sub-Saharan racial groups of Africa | Correlation with sucrose AUC scores. | (51) | - | - |
| **GNAT3** | rs10242727 | Candidate | Measurements of sensitivity to sucrose were performed as described previously (Fushan et al. 2009). A series of preliminary trials empirically determined that solutions of 0, 0.5%, 1%, 2%, 2.4%, 2.8%, 3.2%, 3.6%, and 4% sucrose (Sigma, dissolved in deionized water) produced the best discrimination curves in a representative subpopulation of our subjects. Each concentration is used to calculate a detection threshold for a given sucrose interval (i.e., 0–0.5%, 0.5–1%, etc.). Each subject participated in 1 experiment that consisted of 6 replications performed over 3 sessions. Subjects were asked to complete 2 replications of the ranking test per session with a mandatory 5-min break between replications. Individual sessions were separated by at least a 24-h period. Subjects were presented with 20 ml of each of the solutions in randomized order and could ask for more at any time during the experiment. Panelists were asked to sample each of the solutions and rank them in order from least to most sweet. To minimize adaptation effects, subjects rinsed with water between each sample. Phenotype modeling Data from the 6 replications were pooled for each subject. For each pairwise sucrose concentration (0–0.5%, 0.5–1%, etc.), the R-index (hereafter referred to as Rp-index; see Supplementary Figure 1) was calculated as described in O’Mahony et al. (1992). | Participants were enrolled with written informed consent under National Institutes of Health Combined Neuroscience Institutional Review Board protocol 01-DC-0230 and contained individuals (69 males and 91 females) who identified themselves as Caucasian (n = 103), Asian (n = 41), or African–American (n = 16). African–American individuals had origins in the sub-Saharan racial groups of Africa | Correlation with sucrose AUC scores. | (51) | - | - |
| **GNAT3** | rs6961082 | Candidate | Measurements of sensitivity to sucrose were performed as described previously (Fushan et al. 2009). A series of preliminary trials empirically determined that solutions of 0, 0.5%, 1%, 2%, 2.4%, 2.8%, 3.2%, 3.6%, and 4% sucrose (Sigma, dissolved in deionized water) produced the best discrimination curves in a representative subpopulation of our subjects. Each concentration is used to calculate a detection threshold for a given sucrose interval (i.e., 0–0.5%, 0.5–1%, etc.). Each subject participated in 1 experiment that consisted of 6 replications performed over 3 sessions. Subjects were asked to complete 2 replications of the ranking test per session with a mandatory 5-min break between replications. Individual sessions were separated by at least a 24-h period. Subjects were presented with 20 ml of each of the solutions in randomized order and could ask for more at any time during the experiment. Panelists were asked to sample each of the solutions and rank them in order from least to most sweet. To minimize adaptation effects, subjects rinsed with water between each sample. Phenotype modeling Data from the 6 replications were pooled for each subject. For each pairwise sucrose concentration (0–0.5%, 0.5–1%, etc.), the R-index (hereafter referred to as Rp-index; see Supplementary Figure 1) was calculated as described in O’Mahony et al. (1992). | Participants were enrolled with written informed consent under National Institutes of Health Combined Neuroscience Institutional Review Board protocol 01-DC-0230 and contained individuals (69 males and 91 females) who identified themselves as Caucasian (n = 103), Asian (n = 41), or African–American (n = 16). African–American individuals had origins in the sub-Saharan racial groups of Africa | Correlation with sucrose AUC scores. | (51) | - | - |
| **GNAT3** | rs6979450 | Candidate | Measurements of sensitivity to sucrose were performed as described previously (Fushan et al. 2009). A series of preliminary trials empirically determined that solutions of 0, 0.5%, 1%, 2%, 2.4%, 2.8%, 3.2%, 3.6%, and 4% sucrose (Sigma, dissolved in deionized water) produced the best discrimination curves in a representative subpopulation of our subjects. Each concentration is used to calculate a detection threshold for a given sucrose interval (i.e., 0–0.5%, 0.5–1%, etc.). Each subject participated in 1 experiment that consisted of 6 replications performed over 3 sessions. Subjects were asked to complete 2 replications of the ranking test per session with a mandatory 5-min break between replications. Individual sessions were separated by at least a 24-h period. Subjects were presented with 20 ml of each of the solutions in randomized order and could ask for more at any time during the experiment. Panelists were asked to sample each of the solutions and rank them in order from least to most sweet. To minimize adaptation effects, subjects rinsed with water between each sample. Phenotype modeling Data from the 6 replications were pooled for each subject. For each pairwise sucrose concentration (0–0.5%, 0.5–1%, etc.), the R-index (hereafter referred to as Rp-index; see Supplementary Figure 1) was calculated as described in O’Mahony et al. (1992). | Participants were enrolled with written informed consent under National Institutes of Health Combined Neuroscience Institutional Review Board protocol 01-DC-0230 and contained individuals (69 males and 91 females) who identified themselves as Caucasian (n = 103), Asian (n = 41), or African–American (n = 16). African–American individuals had origins in the sub-Saharan racial groups of Africa | Correlation with sucrose AUC scores. | (51) | - | - |
| **GNAT3** | rs7776757 | Candidate | Measurements of sensitivity to sucrose were performed as described previously (Fushan et al. 2009). A series of preliminary trials empirically determined that solutions of 0, 0.5%, 1%, 2%, 2.4%, 2.8%, 3.2%, 3.6%, and 4% sucrose (Sigma, dissolved in deionized water) produced the best discrimination curves in a representative subpopulation of our subjects. Each concentration is used to calculate a detection threshold for a given sucrose interval (i.e., 0–0.5%, 0.5–1%, etc.). Each subject participated in 1 experiment that consisted of 6 replications performed over 3 sessions. Subjects were asked to complete 2 replications of the ranking test per session with a mandatory 5-min break between replications. Individual sessions were separated by at least a 24-h period. Subjects were presented with 20 ml of each of the solutions in randomized order and could ask for more at any time during the experiment. Panelists were asked to sample each of the solutions and rank them in order from least to most sweet. To minimize adaptation effects, subjects rinsed with water between each sample. Phenotype modeling Data from the 6 replications were pooled for each subject. For each pairwise sucrose concentration (0–0.5%, 0.5–1%, etc.), the R-index (hereafter referred to as Rp-index; see Supplementary Figure 1) was calculated as described in O’Mahony et al. (1992). | Participants were enrolled with written informed consent under National Institutes of Health Combined Neuroscience Institutional Review Board protocol 01-DC-0230 and contained individuals (69 males and 91 females) who identified themselves as Caucasian (n = 103), Asian (n = 41), or African–American (n = 16). African–American individuals had origins in the sub-Saharan racial groups of Africa | Correlation with sucrose AUC scores. | (51) | - | - |
| **LEP** | rs2167270 | Candidate | Between April 2002 and February 2004, we recruited 3,653 residents who had been participating in a public health physical checkup for citizens living in Suita City, located in the northern area of Osaka, Japan, since 1991. | Semi-Quantification of Sweet Preference A questionnaire designed to measure whether subjects had a “sweet tooth” was administered to each subject. The questionnaire consisted of a single question: “Do you like things that taste sweet?” to which the following responses were available: 1: No, I hate them; 2: No, I don’t like them very much; 3: Neither yes or no; 4: Yes, I like them; and 5: Yes, I like them very much. The written answers were confirmed during an interview. To eliminate crossover, subjects who selected response number 5, above, were considered to have a sweet preference (n=1,751) and those that selected response numbers 1, 2, or 3 were defined as controls (n=869). | Association with sweet preference. | (80) | - | - |
| **LEPR** | rs1137100 | Candidate | Between April 2002 and February 2004, we recruited 3,653 residents who had been participating in a public health physical checkup for citizens living in Suita City, located in the northern area of Osaka, Japan, since 1991. | Semi-Quantification of Sweet Preference A questionnaire designed to measure whether subjects had a “sweet tooth” was administered to each subject. The questionnaire consisted of a single question: “Do you like things that taste sweet?” to which the following responses were available: 1: No, I hate them; 2: No, I don’t like them very much; 3: Neither yes or no; 4: Yes, I like them; and 5: Yes, I like them very much. The written answers were confirmed during an interview. To eliminate crossover, subjects who selected response number 5, above, were considered to have a sweet preference (n=1,751) and those that selected response numbers 1, 2, or 3 were defined as controls (n=869). | Association with sweet preference. | (80) | A total of 185 unrelated Czech Caucasian individuals were recruited for this case-control study and were divided in two groups. All the participants in the study, both obese individuals and the lean controls, were recruited in a mass media campaign addressing the population of the south Moravia region in the Czech Republic. Both the cases and the controls underwent the same diagnostic procedures to avoid possible selection bias. In recruitment, the inclusion and exclusion criteria of Ma et al. were used. The case group consisted of 125 obese individuals (body mass index [BMI] ≥ 30 kg/m2; mean BMI, 37.7 ± 6.38 kg/m2: median age, 50.0 years; range, 18.6–68.9 years). In this group of obese individuals, a subset of 34 morbidly obese patients was identified using the criterion of BMI ≥ 40 kg/m2 (mean BMI, 45.5 ± 3.6 kg/m2: median age, 50.5 years; range, 18.6–67.8 years); all of these 34 patients were available for the plasma leptin and sObR determinations. Participants were advised to complete specific selfadministered demographic questionnaires along with 7-day food records.The control group consisted of 60 healthy, normal-weight, control subjects with no history of childhood obesity (mean BMI, 25.8 ± 3.3 kg/m2: median age, 47.5 years; range, 18.1–67.5 years). Data on personal or family history of obesity, birth weight, age at onset of obesity, eating disorders, age of menarche and menopause in women, family history of sterility, infertility or stillbirth were obtained by a professional using a semi-structured interview. A positive family history for obesity was estimated as one obese relative (BMI ≥ 30 kg/m2 in the close family – siblings, parents and their siblings and grandparents). Both the obese cases and the controls underwent the same examinations on their anthropometric characteristics, dietary intake and genetic background of the individual and the family.  A total of 185 unrelated Czech Caucasian individuals were recruited for this case-control study and were divided in two groups. All the participants in the study, both obese individuals and the lean controls, were recruited in a mass media campaign addressing the population of the south Moravia region in the Czech Republic. Both the cases and the controls underwent the same diagnostic procedures to avoid possible selection bias. In recruitment, the inclusion and exclusion criteria of Ma et al. were used. The case group consisted of 125 obese individuals (body mass index [BMI] ≥ 30 kg/m2; mean BMI, 37.7 ± 6.38 kg/m2: median age, 50.0 years; range, 18.6–68.9 years). In this group of obese individuals, a subset of 34 morbidly obese patients was identified using the criterion of BMI ≥ 40 kg/m2 (mean BMI, 45.5 ± 3.6 kg/m2: median age, 50.5 years; range, 18.6–67.8 years); all of these 34 patients were available for the plasma leptin and sObR determinations. The control group consisted of 60 healthy, normal-weight, control subjects with no history of childhood obesity (mean BMI, 25.8 ± 3.3 kg/m2: median age, 47.5 years; range, 18.1–67.5 years). Data on personal or family history of obesity, birth weight, age at onset of obesity, eating disorders, age of menarche and menopause in women, family history of sterility, infertility or stillbirth were obtained by a professional using a semi-structured interview. A positive family history for obesity was estimated as one obese relative (BMI ≥ 30 kg/m2 in the close family – siblings, parents and their siblings and grandparents). Both the obese cases and the controls underwent the same examinations on their anthropometric characteristics, dietary intake and genetic background of the individual and the family.  A total of 185 unrelated Czech Caucasian individuals were recruited for this case-control study and were divided in two groups. All the participants in the study, both obese individuals and the lean controls, were recruited in a mass media campaign addressing the population of the south Moravia region in the Czech Republic. Both the cases and the controls underwent the same diagnostic procedures to avoid possible selection bias. In recruitment, the inclusion and exclusion criteria of Ma et al. were used. The case group consisted of 125 obese individuals (body mass index [BMI] ≥ 30 kg/m2; mean BMI, 37.7 ± 6.38 kg/m2: median age, 50.0 years; range, 18.6–68.9 years). In this group of obese individuals, a subset of 34 morbidly obese patients was identified using the criterion of BMI ≥ 40 kg/m2 (mean BMI, 45.5 ± 3.6 kg/m2: median age, 50.5 years; range, 18.6–67.8 years); all of these 34 patients were available for the plasma leptin and sObR determinations. The control group consisted of 60 healthy, normal-weight, control subjects with no history of childhood obesity (mean BMI, 25.8 ± 3.3 kg/m2: median age, 47.5 years; range, 18.1–67.5 years). Data on personal or family history of obesity, birth weight, age at onset of obesity, eating disorders, age of menarche and menopause in women, family history of sterility, infertility or stillbirth were obtained by a professional using a semi-structured interview. A positive family history for obesity was estimated as one obese relative (BMI ≥ 30 kg/m2 in the close family – siblings, parents and their siblings and grandparents). Both the obese cases and the controls underwent the same examinations on their anthropometric characteristics, dietary intake and genetic background of the individual and the family. | (81) |
| **LOC107986812** | rs7792845 | Candidate | Measurements of sensitivity to sucrose were performed as described previously (Fushan et al. 2009). A series of preliminary trials empirically determined that solutions of 0, 0.5%, 1%, 2%, 2.4%, 2.8%, 3.2%, 3.6%, and 4% sucrose (Sigma, dissolved in deionized water) produced the best discrimination curves in a representative subpopulation of our subjects. Each concentration is used to calculate a detection threshold for a given sucrose interval (i.e., 0–0.5%, 0.5–1%, etc.). Each subject participated in 1 experiment that consisted of 6 replications performed over 3 sessions. Subjects were asked to complete 2 replications of the ranking test per session with a mandatory 5-min break between replications. Individual sessions were separated by at least a 24-h period. Subjects were presented with 20 ml of each of the solutions in randomized order and could ask for more at any time during the experiment. Panelists were asked to sample each of the solutions and rank them in order from least to most sweet. To minimize adaptation effects, subjects rinsed with water between each sample. Phenotype modeling Data from the 6 replications were pooled for each subject. For each pairwise sucrose concentration (0–0.5%, 0.5–1%, etc.), the R-index (hereafter referred to as Rp-index; see Supplementary Figure 1) was calculated as described in O’Mahony et al. (1992). | Participants were enrolled with written informed consent under National Institutes of Health Combined Neuroscience Institutional Review Board protocol 01-DC-0230 and contained individuals (69 males and 91 females) who identified themselves as Caucasian (n = 103), Asian (n = 41), or African–American (n = 16). African–American individuals had origins in the sub-Saharan racial groups of Africa | Correlation with sucrose AUC scores. | (82) | Mothers of healthy children 7–14 years of age were recruited for a “taste study” from local advertisements and from a list of past participants who asked to be notified of future studies Only children who were healthy at the time of testing, with no major medical illness such as diabetes, heart disease, or asthma, were included. the study population consisted of 235 children (female 124, race balck 136, white 46, Asian 2, other/more than one 51; ethnicity (non-Hispanic 219) . For a subset of the subjects tested (n = 96; (female 53, race balck 49, white 16, Asian 2, other/more than one 29; ethnicity (non-Hispanic 84), we obtained additional anthropometric measures and dietary intake data. | (60) |
| **LOC107986812** | rs940541 | Candidate | Measurements of sensitivity to sucrose were performed as described previously (Fushan et al. 2009). A series of preliminary trials empirically determined that solutions of 0, 0.5%, 1%, 2%, 2.4%, 2.8%, 3.2%, 3.6%, and 4% sucrose (Sigma, dissolved in deionized water) produced the best discrimination curves in a representative subpopulation of our subjects. Each concentration is used to calculate a detection threshold for a given sucrose interval (i.e., 0–0.5%, 0.5–1%, etc.). Each subject participated in 1 experiment that consisted of 6 replications performed over 3 sessions. Subjects were asked to complete 2 replications of the ranking test per session with a mandatory 5-min break between replications. Individual sessions were separated by at least a 24-h period. Subjects were presented with 20 ml of each of the solutions in randomized order and could ask for more at any time during the experiment. Panelists were asked to sample each of the solutions and rank them in order from least to most sweet. To minimize adaptation effects, subjects rinsed with water between each sample. Phenotype modeling Data from the 6 replications were pooled for each subject. For each pairwise sucrose concentration (0–0.5%, 0.5–1%, etc.), the R-index (hereafter referred to as Rp-index; see Supplementary Figure 1) was calculated as described in O’Mahony et al. (1992). | Participants were enrolled with written informed consent under National Institutes of Health Combined Neuroscience Institutional Review Board protocol 01-DC-0230 and contained individuals (69 males and 91 females) who identified themselves as Caucasian (n = 103), Asian (n = 41), or African–American (n = 16). African–American individuals had origins in the sub-Saharan racial groups of Africa | Correlation with sucrose AUC scores. | (82) | - | - |
| **LOC107986812** | rs1107660 | Candidate | Measurements of sensitivity to sucrose were performed as described previously (Fushan et al. 2009). A series of preliminary trials empirically determined that solutions of 0, 0.5%, 1%, 2%, 2.4%, 2.8%, 3.2%, 3.6%, and 4% sucrose (Sigma, dissolved in deionized water) produced the best discrimination curves in a representative subpopulation of our subjects. Each concentration is used to calculate a detection threshold for a given sucrose interval (i.e., 0–0.5%, 0.5–1%, etc.). Each subject participated in 1 experiment that consisted of 6 replications performed over 3 sessions. Subjects were asked to complete 2 replications of the ranking test per session with a mandatory 5-min break between replications. Individual sessions were separated by at least a 24-h period. Subjects were presented with 20 ml of each of the solutions in randomized order and could ask for more at any time during the experiment. Panelists were asked to sample each of the solutions and rank them in order from least to most sweet. To minimize adaptation effects, subjects rinsed with water between each sample. Phenotype modeling Data from the 6 replications were pooled for each subject. For each pairwise sucrose concentration (0–0.5%, 0.5–1%, etc.), the R-index (hereafter referred to as Rp-index; see Supplementary Figure 1) was calculated as described in O’Mahony et al. (1992). | Participants were enrolled with written informed consent under National Institutes of Health Combined Neuroscience Institutional Review Board protocol 01-DC-0230 and contained individuals (69 males and 91 females) who identified themselves as Caucasian (n = 103), Asian (n = 41), or African–American (n = 16). African–American individuals had origins in the sub-Saharan racial groups of Africa | Correlation with sucrose AUC scores. | (82) | - | - |
| **LOC107986812** | rs1107657 | Candidate | Measurements of sensitivity to sucrose were performed as described previously (Fushan et al. 2009). A series of preliminary trials empirically determined that solutions of 0, 0.5%, 1%, 2%, 2.4%, 2.8%, 3.2%, 3.6%, and 4% sucrose (Sigma, dissolved in deionized water) produced the best discrimination curves in a representative subpopulation of our subjects. Each concentration is used to calculate a detection threshold for a given sucrose interval (i.e., 0–0.5%, 0.5–1%, etc.). Each subject participated in 1 experiment that consisted of 6 replications performed over 3 sessions. Subjects were asked to complete 2 replications of the ranking test per session with a mandatory 5-min break between replications. Individual sessions were separated by at least a 24-h period. Subjects were presented with 20 ml of each of the solutions in randomized order and could ask for more at any time during the experiment. Panelists were asked to sample each of the solutions and rank them in order from least to most sweet. To minimize adaptation effects, subjects rinsed with water between each sample. Phenotype modeling Data from the 6 replications were pooled for each subject. For each pairwise sucrose concentration (0–0.5%, 0.5–1%, etc.), the R-index (hereafter referred to as Rp-index; see Supplementary Figure 1) was calculated as described in O’Mahony et al. (1992). | Participants were enrolled with written informed consent under National Institutes of Health Combined Neuroscience Institutional Review Board protocol 01-DC-0230 and contained individuals (69 males and 91 females) who identified themselves as Caucasian (n = 103), Asian (n = 41), or African–American (n = 16). African–American individuals had origins in the sub-Saharan racial groups of Africa | Correlation with sucrose AUC scores. | (82) | - | - |
| **LOC107986812** | rs2012380 | Candidate | Measurements of sensitivity to sucrose were performed as described previously (Fushan et al. 2009). A series of preliminary trials empirically determined that solutions of 0, 0.5%, 1%, 2%, 2.4%, 2.8%, 3.2%, 3.6%, and 4% sucrose (Sigma, dissolved in deionized water) produced the best discrimination curves in a representative subpopulation of our subjects. Each concentration is used to calculate a detection threshold for a given sucrose interval (i.e., 0–0.5%, 0.5–1%, etc.). Each subject participated in 1 experiment that consisted of 6 replications performed over 3 sessions. Subjects were asked to complete 2 replications of the ranking test per session with a mandatory 5-min break between replications. Individual sessions were separated by at least a 24-h period. Subjects were presented with 20 ml of each of the solutions in randomized order and could ask for more at any time during the experiment. Panelists were asked to sample each of the solutions and rank them in order from least to most sweet. To minimize adaptation effects, subjects rinsed with water between each sample. Phenotype modeling Data from the 6 replications were pooled for each subject. For each pairwise sucrose concentration (0–0.5%, 0.5–1%, etc.), the R-index (hereafter referred to as Rp-index; see Supplementary Figure 1) was calculated as described in O’Mahony et al. (1992). | Participants were enrolled with written informed consent under National Institutes of Health Combined Neuroscience Institutional Review Board protocol 01-DC-0230 and contained individuals (69 males and 91 females) who identified themselves as Caucasian (n = 103), Asian (n = 41), or African–American (n = 16). African–American individuals had origins in the sub-Saharan racial groups of Africa | Correlation with sucrose AUC scores. | (82) | - | - |
| **NA** | rs17724320 | Candidate | Validated food frequency questionnaire (FFQ) on their dietary intake, which is designed to estimate regular intake of 178 food items in the year before enrolment. Scientists calculated and assigned the values (grams/day) for total energy, fat, carbohydrates, protein and alcohol for each food item in the FFQ. Energy-adjusted intake was calculated using the nutrient-density method. | Hamlet study. The Hamlet study is a cross-sectional, singlecenter study in 400 men aged 40 to 80 years living independently. In brief, participants visited the study center twice for physical examinations, including drawing of blood, and filled in a validated food frequency questionnaire (FFQ) on their dietary intake, which is designed to estimate regular intake of 178 food items in the year before enrolment. Data collection took place between March 2001 and April 2002. (HapMap. A total of 270 people are included in the HapMap database (Phase II) [36]: 30 trios of US residents with Northern and Western European ancestry (CEU), 30 trios of Yoruba people from Ibadan, Nigeria (YRI), 45 unrelated Japanese individuals from the Tokyo area (ASN), and 45 unrelated Chinese individuals from Beijing (ASN) | Dose-response relationship with mono- and disaccharide intake as for total carbohydrates for the derived allele. | (83) | - | - |
| **NPY1R** | rs11100489 | rs17724320 | Candidate | Validated food frequency questionnaire (FFQ) on their dietary intake, which is designed to estimate regular intake of 178 food items in the year before enrolment. Scientists calculated and assigned the values (grams/day) for total energy, fat, carbohydrates, protein and alcohol for each food item in the FFQ. Energy-adjusted intake was calculated using the nutrient-density method. | Dose-response relationship with mono- and disaccharide intake as for total carbohydrates for the derived allele. | (83) | - | - |
| **NPY1R** | rs12507653 | rs17724320 | Candidate | Validated food frequency questionnaire (FFQ) on their dietary intake, which is designed to estimate regular intake of 178 food items in the year before enrolment. Scientists calculated and assigned the values (grams/day) for total energy, fat, carbohydrates, protein and alcohol for each food item in the FFQ. Energy-adjusted intake was calculated using the nutrient-density method. | AA or AT vs. TT men consumed less mono- and disaccharides. | (83) | - | - |
| **NPY1R** | rs4234955 | rs17724320 | Candidate | Validated food frequency questionnaire (FFQ) on their dietary intake, which is designed to estimate regular intake of 178 food items in the year before enrolment. Scientists calculated and assigned the values (grams/day) for total energy, fat, carbohydrates, protein and alcohol for each food item in the FFQ. Energy-adjusted intake was calculated using the nutrient-density method. | Dose-response relationship with mono- and disaccharide intake as for total carbohydrates for the derived allele. | (83) | - | - |
| **NPY2R** | rs12507396 | rs17724320 | Candidate | Validated food frequency questionnaire (FFQ) on their dietary intake, which is designed to estimate regular intake of 178 food items in the year before enrolment. Scientists calculated and assigned the values (grams/day) for total energy, fat, carbohydrates, protein and alcohol for each food item in the FFQ. Energy-adjusted intake was calculated using the nutrient-density method. | Dose-response relationship with mono- and disaccharide intake as for total carbohydrates for the derived allele. | (83) | - | - |
| **OPRM1** | rs1799971 | Candidate | Three variables from the Food Preference Questionnaire were used in the analyses. This 72-item scale was designed as a 2 (FAT: high vs low) by 3 (CARBOHYDRATE: high simple, high complex, low carbohydrate/high protein) measure of preference for various kinds of macronutrients. Respondents indicate their preference for each food on a ninepoint Likert scale. The high fat preference score comprises the mean of 36 items (for example, onion rings and barbeque chicken); the high sugar preference score the mean of 24 items (for example, canned pears and dried dates); and the high fat and high sugar preference score the mean of 12 items (for example, chocolate layer cake and pecan pie) | Three hundred women (n = 238) and men (n =62) between the ages of 24 and 50 years took part in the study. Participants were required to be fluent in English and to have lived in North America for at least 5 years before their enrolment. All female participants were also pre-menopausal as identified by the self-reporting of regular menstrual cycles. Exclusion criteria included a current diagnosis of any psychotic disorder, substance abuse, alcoholism or a serious medical/physical illness, such as cancer, heart disease or paralysis. Female participants with a pregnancy within the previous 6 months were also ineligible. The sample comprised a broad range of BMI values, with a distribution representative of the general adult population. In all, 35% had a BMI of 25 kg m2 while the remainder was classified as overweight or obese according to the World Health Organization criteria. In total, 80% of the sample was Caucasian and 13% were of African descent. (Women mean age 33.5 SD=6.6, men mean age 35.2 SD=6.7) | GG associated with stronger sweet preference. | (84) | - | - |
| **OPRM1** | rs495491 | Candidate | Three variables from the Food Preference Questionnaire were used in the analyses. This 72-item scale was designed as a 2 (FAT: high vs low) by 3 (CARBOHYDRATE: high simple, high complex, low carbohydrate/high protein) measure of preference for various kinds of macronutrients. Respondents indicate their preference for each food on a ninepoint Likert scale. The high fat preference score comprises the mean of 36 items (for example, onion rings and barbeque chicken); the high sugar preference score the mean of 24 items (for example, canned pears and dried dates); and the high fat and high sugar preference score the mean of 12 items (for example, chocolate layer cake and pecan pie) | Three hundred women (n = 238) and men (n =62) between the ages of 24 and 50 years took part in the study. Participants were required to be fluent in English and to have lived in North America for at least 5 years before their enrolment. All female participants were also pre-menopausal as identified by the self-reporting of regular menstrual cycles. Exclusion criteria included a current diagnosis of any psychotic disorder, substance abuse, alcoholism or a serious medical/physical illness, such as cancer, heart disease or paralysis. Female participants with a pregnancy within the previous 6 months were also ineligible. The sample comprised a broad range of BMI values, with a distribution representative of the general adult population. In all, 35% had a BMI of 25 kg m2 while the remainder was classified as overweight or obese according to the World Health Organization criteria. In total, 80% of the sample was Caucasian and 13% were of African descent. (Women mean age 33.5 SD=6.6, men mean age 35.2 SD=6.7) | GA reported a lower sweet preference. The A–C haplotype (rs495491, rs563649) was associated with stronger sweet preference than the other two common haplotypes. | (84) | - | - |
| **OPRM1** | rs563649 | Candidate | Three variables from the Food Preference Questionnaire were used in the analyses. This 72-item scale was designed as a 2 (FAT: high vs low) by 3 (CARBOHYDRATE: high simple, high complex, low carbohydrate/high protein) measure of preference for various kinds of macronutrients. Respondents indicate their preference for each food on a ninepoint Likert scale. The high fat preference score comprises the mean of 36 items (for example, onion rings and barbeque chicken); the high sugar preference score the mean of 24 items (for example, canned pears and dried dates); and the high fat and high sugar preference score the mean of 12 items (for example, chocolate layer cake and pecan pie) | Three hundred women (n = 238) and men (n =62) between the ages of 24 and 50 years took part in the study. Participants were required to be fluent in English and to have lived in North America for at least 5 years before their enrolment. All female participants were also pre-menopausal as identified by the self-reporting of regular menstrual cycles. Exclusion criteria included a current diagnosis of any psychotic disorder, substance abuse, alcoholism or a serious medical/physical illness, such as cancer, heart disease or paralysis. Female participants with a pregnancy within the previous 6 months were also ineligible. The sample comprised a broad range of BMI values, with a distribution representative of the general adult population. In all, 35% had a BMI of 25 kg m2 while the remainder was classified as overweight or obese according to the World Health Organization criteria. In total, 80% of the sample was Caucasian and 13% were of African descent. (Women mean age 33.5 SD=6.6, men mean age 35.2 SD=6.7) | The A–C haplotype (rs495491, rs563649) was associated with stronger sweet preference than the other two common haplotypes. | (84) | - | - |
| **OXTR** | rs2268494 | Candidate | Food Preference Questionnaire: This questionnaire provides hedonic ratings for 72 common food items arranged according to a 2 (FAT: high vs low) x 3 (OTHER MACRONUTRIENT: high simple sugar, high complex carbohydrate, and high protein) matrix with 12 items in each cell. In the current study, High Sugar and High Fat Foods is reflected by the 12 items from the high fat/high simple sugar cell; High Sugar Foods includes the 12 items from the High Simple Sugar/Low Fat cell and the High Fat Foods is the total (24 items) of the High Fat/High Carbohydrate and High Fat/High Protein subscales. The 72 food items of the Food Preference Questionnaire vary systematically and significantly with respect to their macronutrient content. | The sample consisted of 460 adults (females = 346) between 24 and 50 years of age. Inclusion criteria were residence in North America for at least 5 years before enrollment in the study, and fluency in written and spoken English. Women were also required to be premenopausal as indicated by the self-report of a regular menstrual cycle. Exclusion criteria included serious medical illnesses such as cancer or diabetes, or severe physical disabilities such as cerebral palsy. Those with a current axis I diagnosed disorder, with the exception of unipolar depression, were also excluded. In addition, women with a pregnancy in the previous 6 months, or who were lactating, were not included. The majority of the sample was Caucasian (79%), with 15% identifying as African descent and the remainder as ‘Other’. The sample represented a broad range of body mass index (BMI) values (17.8–75.2 kg m2). Participants comprised a community-based sample and were recruited from posters, newspaper advertisements and online sites such as Craigslist and Kijiji in a large Canadian city. Females age 33.4 (SD= 6.6); males age 34.3 (SD=7.0) | AA or AT vs. TT associated with stronger preference for sweet and fatty foods. | (85) | - | - |
| **SLC2A2** | rs5400 | Candidate | Two different methods of dietary assessment were used to assess habitual intake of food and beverages. A food-frequency questionnaire (FFQ) was used in population 1, and 2 sets of 3-d food records were used in population 2. The self-administered FFQ used in population 1 was the Toronto-modified Willett questionnaire. The FFQ consisted of 184 food and beverage items and 12 vitamin and dietary supplement items. In addition to total sugars (defined as mono- and disaccharides), the nutrient database also provided information on intakes of sucrose, maltose, lactose, fructose, and glucose. To examine the type of foods consumed that contributed to the intake of sugars, the consumption of daily servings from specific food groups that contained sugars were compared. Each food item response was first converted into daily servings and subsequently summed within its respective food group. Total fruit, which included fruit juice and fruit, and dairy products, corresponded to the original sections of food groups in the FFQ with minor modifications such as excluding avocado and nondairy coffee whitener, respectively, to reflect sugar sources. Sweets included chocolates, candy, jams, baked goods, and ice cream. Sweetened beverages included regular soft drinks, fruit drinks (not fruit juice), and sport drinks. For population 2, each subject was instructed on how to complete a 3-d food record, including 2 weekdays and 1 weekend day, on 2 separate visits. | Population 1 Subjects were participants from the Toronto Nutrigenomics and Health Study, which is a cross-sectional study examining the role of genetics in food intake as well as gene-diet interactions on biomarkers of chronic disease in young men and women between 20 and 29 y of age. Since October 2004, men (n = 391) and women (n = 886) with an average BMI of 23.0 6 6.7 (mean 6 SD) were recruited from the University of Toronto campus. Women who were pregnant or breastfeeding were excluded from the study. For the current analyses, we also excluded subjects who may have underreported (,800 kcal/d) or overreported (.3500 kcal/d for women, .4000 kcal/d for men) their energy intakes (n = 100) or reported consuming a special diet that restricted carbohydrates, fat, or calories (n = 54). We also excluded smokers (n = 79), one subject who had type 1 diabetes, and individuals who had missing data on potential confounders (n = 6). The final sample size consisted of 309 men and 728 women. Subjects were classified by self-reported ethnocultural ancestry and were grouped as white (n = 482), East Asian (n = 362), South Asian (n = 114), or other (n = 79). Population 2 The second population consisted of participants from the Canadian Trial of Carbohydrates in Diabetes multicenter intervention study, described in detail elsewhere (25). Subjects were recruited from 5 centers across Canada (in Edmonton, London, Toronto, Montreal, and Sherbrooke) over a 1-y period (2002–2003), and the baseline data collected was used for this study. All subjects were diagnosed with type 2 diabetes according to the Canadian Diabetes Association (CDA) criteria. Subjects included men and women who had early type 2 diabetes with near-normal glycated hemoglobin (6.2 6 0.6%) and who were considered not to require medications. Therefore, subjects were instructed to follow the CDA dietary guidelines and were excluded if they were using any hypoglycemic, antihyperglycemic, or oral steroid drugs or experienced a major cardiovascular event or surgery in the past 6 mo. Of the 166 subjects recruited, 127 subjects agreed to give a blood sample for genotype analysis, one subject’s genotype remained undetermined, 17 subjects had incomplete baseline dietary data, and 9 individuals had missing data on potential confounders, which left 100 subjects for the final analyses. According to 2 sets of 3-d food records, all subjects reported consuming between 800 and 3500 kcal/d for women and 800 to 4000 kcal/d for men, and therefore, no exclusions were made for possible under- or overreporting. The study consisted of men (n = 49) and women (n = 51) between the ages of 42 and 75 y with a mean (6SD) BMI of 30.6 6 4.2. | Intake of sugars, sucrose, fructose, glucose, sweetened beverages was greater among carriers of the Ile allele compared with those with the Thr/Thr genotype. | (86) | - | - |
| **TAS1R1** | rs17492553 | Candidate | Adults used a general labeled magnitude scale (gLMS) to rate the taste intensity of oral stimuli.of 1M NaCl. | A convenience sample of reportedly healthy, nonsmoking adults was recruited from the University of Connecticut community to participate in an observational study of variation in oral sensation, diet, and health. Exclusion criteria included pregnancy, severe food allergies, and thyroid disease. The study sample included 92 adults, primarily of European ancestry (84.8%), female (76%), and middle aged (mean 40.9±12.2 SD). Other ethnicities represented in the sample were Black (5.4%), Hispanic or Latino (5.4%), Asian (3.3%), and other (1.1%). | TT associated with lower intensities than CC (regional application on FP). TT, CT reported lower intensities than CC (regional application on CP). | (53) | - | - |
| **TAS1R1** | rs34160967 | Candidate | Adults used a general labeled magnitude scale (gLMS) to rate the taste intensity of oral stimuli.of 1M NaCl. | A convenience sample of reportedly healthy, nonsmoking adults was recruited from the University of Connecticut community to participate in an observational study of variation in oral sensation, diet, and health. Exclusion criteria included pregnancy, severe food allergies, and thyroid disease. The study sample included 92 adults, primarily of European ancestry (84.8%), female (76%), and middle aged (mean 40.9±12.2 SD). Other ethnicities represented in the sample were Black (5.4%), Hispanic or Latino (5.4%), Asian (3.3%), and other (1.1%). | AA/AG associated with lower intensities than GG homozygotes (regional application on CP). | (53) | - | - |
| **TRPV1** | rs8065080 | Candidate | Preferences for food tastes like sweetness, were asked about as ‘Do you like food with (sweet/salty/etc.) taste?’ The preference for food taste was given as ‘very dislike’ (1), ‘dislike’ (2), ‘moderate’ (3), ‘like’ (4), or ‘very like’ (5). Preferences were categorized into two groups as ‘low preference’, including ‘very dislike’ and ‘dislike’, and as ‘high preference’, including the rest of the scale for each taste. | A total of 4,183 men and 4,659 women aged 40–69 years (8,842 participants) were recruited from two community-based epidemiological cohorts, the rural community of Anseong city and the urban community of Ansan city. Subjects from both cohorts were sources of the genetic information that was collected in 2001 as part of the KoGES, and the part containing genetic information was called the Korean Association Resource (KARE) study. All participants had lived within the survey area for at least 6 months and were mentally and physically healthy. | Minor allele positively associated with sweet preference. | (64) | - | - |
| **TRPV1** | rs161364 | Candidate | Preferences for food tastes like sweetness, were asked about as ‘Do you like food with (sweet/salty/etc.) taste?’ The preference for food taste was given as ‘very dislike’ (1), ‘dislike’ (2), ‘moderate’ (3), ‘like’ (4), or ‘very like’ (5). Preferences were categorized into two groups as ‘low preference’, including ‘very dislike’ and ‘dislike’, and as ‘high preference’, including the rest of the scale for each taste. | A total of 4,183 men and 4,659 women aged 40–69 years (8,842 participants) were recruited from two community-based epidemiological cohorts, the rural community of Anseong city and the urban community of Ansan city. Subjects from both cohorts were sources of the genetic information that was collected in 2001 as part of the KoGES, and the part containing genetic information was called the Korean Association Resource (KARE) study. All participants had lived within the survey area for at least 6 months and were mentally and physically healthy. | Minor allele positively associated with sweet preference. | (64) | - | - |

**Supplementary Table 6 Study characteristics of genetic association studies related to sweet taste preferences (no associations)**

**Supplementary Table 6 Study characteristics of genetic association studies related to sweet taste preferences (no associations)**

| **Gene** | **SNP** | **Discovery method** | **Phenotype assessment method** | **Study population characteristics** | **Reference (no association)** |
| --- | --- | --- | --- | --- | --- |
| **TAS1R2** | rs35874116 | Candidate | The study analyzed threshold and suprathreshold sensitivity to sucrose by employing the novel use of signal detection analysis and R-index measures for this trait. The quantitative stimuli consisted of 9 different blinded sucrose solutions, which subjects sorted from least sweet to most sweet. For each pairwise sucrose concentration (e.g. 0−0.5%, 0.5−1%, etc) we estimated the classical signal detection measure of sensitivity P(A) - the area under a receiver operating characteristic (ROC) curve - by calculating the R-index . The area under an ROC curve, and hence the R-index, ranges from 0.5 (chance level discrimination) to 1 (perfect discrimination) and measures an individual's ability to discriminate between two stimuli. To obtain a singular measure of sensitivity across the entire sucrose concentration series, the pairwise R-indices were summed to create a concentration-response function for each subject and the area under this curve (AUC) was determined. The AUC, which can theoretically range from 0 (complete non-discrimination) to 9.25 (perfect discrimination) in this testing paradigm, was used as the dependent variable for assessing the effect of genotype on sucrose sensitivity. | Our test population consisted of 144 unrelated individuals, who identified themselves as European (n = 92), Asian (n = 37), or African (n = 15) | (66) |
| **TAS2R31** | rs10772423 | Candidate | Intensity ratings (test samples: sucrose, gentiobiose, aspartame, rebaudioside A and D). Participants reported perceived intensity of the test stimuli by rating on a general Labeled Magnitude Scale (gLMS). The gLMS anchors are 0 (‘no sensation’) to 100 (’the strongest imaginable sensation of any kind’), with descriptors at 1.4 (‘barely detectable’), 6 (‘weak’), 17 (‘moderate’), 35 (‘strong’) and 51 (‘very strong’). | Here, we present data from a follow-up study to a larger project on the genetics of oral  sensation (Project GIANT-CS). Participants who had completed the main study and had been genotyped were invited to return to our laboratory to taste a variety of tastants not originally included in the main study, including multiple non-nutritive sweeteners and disaccharides. 122 participants returned to complete the follow-up study. The study was conducted in the Sensory Evaluation Center at the Pennsylvania State University in individual testing test booths under white light. After participants were re-consented in writing for the follow-up study, they participated in a 3- minute training session in a multifunction space in our facility prior to entering the test booths. In isolated testing booths, participants rated bitter, sweet, and metallic sensations on a computerized general Labeled Magnitude Scale (gLMS). Participants were screened prior to the start of the main study. Eligibility criteria included: between 18–45 years old, not pregnant or breastfeeding, non-smoker (had not smoked in the last 30 days), no known defects of smell or taste, no lip, cheek or tongue piercings, no history of any condition involving chronic pain, not currently taking any prescription pain medication, no reported history of choking or difficulty swallowing and no history of thyroid disease. Here, we report data from 122 participants (44 men), with a mean age 27.7 (±7.89) years. Self-reported race and ethnicity was collected based on criteria provided by the 1997 OMB Directive 15. This population is largely of European ancestry (n=88), with marginal representation from other ancestry, African (n=2) and Asian (n=21), with 9 individuals choosing to not disclose their ancestry. | (45) |
|  |  | Candidate | A general labeled magnitude scale (gLMS) was used to collect perceived intensity of suprathreshold stimuli (AceK sweetness). This scale ranges from 0 (“no sensation”) to 100 (“the strongest imaginable sensation of any kind”), with intermediate descriptors at 1.4 (“barely detectable”), 6 (“weak”), 17 (“moderate”), 35 (“strong”), and 51 (“very strong”). | Eligibility criteria included the following: between 18 and 45 years old; not pregnant or breastfeeding; nonsmoker (had not smoked in the last 30 days); no known defects of smell or taste; no lip, cheek, or tongue piercings; no history Bitterness of Acesulfame Potassium Varies With TAS2R Polymorphisms 381 of any condition involving chronic pain; not currently taking any prescription pain medication; no reported history of choking or difficulty swallowing; and no history of thyroid disease. Participants also needed to be willing to provide a DNA sample via saliva. DNA samples were available from 147 participants. Race and ethnicity was self-reported using categories provided by the 1997 OMB Directive 15. To minimize potential population stratification, which can potentially cause false negatives and false positives in gene association studies (Hamer and Sirota 2000), individuals with Asian (n = 18), African (n = 5), or unknown (n = 15) ancestry were excluded from the present analyses. Thus, we report data from 108 participants (34 men) of European ancestry, with a mean age of 27.4 (±8.1 SD) years. Results were not substantively different in the mixed ancestry sample, but we report only the results for the European–American participants to facilitate interpretation of the linkage disequilibrium (LD) plots. | (8) |
| **TAS2R4** | rs2234001 | Candidate | Intensity ratings (test samples: sucrose, gentiobiose, aspartame, rebaudioside A and D). Participants reported perceived intensity of the test stimuli by rating on a general Labeled Magnitude Scale (gLMS). The gLMS anchors are 0 (‘no sensation’) to 100 (’the strongest imaginable sensation of any kind’), with descriptors at 1.4 (‘barely detectable’), 6 (‘weak’), 17 (‘moderate’), 35 (‘strong’) and 51 (‘very strong’). | Here, we present data from a follow-up study to a larger project on the genetics of oral sensation (Project GIANT-CS). Participants who had completed the main study and had been genotyped were invited to return to our laboratory to taste a variety of tastants not originally included in the main study, including multiple non-nutritive sweeteners and disaccharides. 122 participants returned to complete the follow-up study. The study was conducted in the Sensory Evaluation Center at the Pennsylvania State University in individual testing test booths under white light. After participants were re-consented in writing for the follow-up study, they participated in a 3- minute training session in a multifunction space in our facility prior to entering the test booths. In isolated testing booths, participants rated bitter, sweet, and metallic sensations on a computerized general Labeled Magnitude Scale (gLMS). Participants were screened prior to the start of the main study. Eligibility criteria included: between 18–45 years old, not pregnant or breastfeeding, non-smoker (had not smoked in the last 30 days), no known defects of smell or taste, no lip, cheek or tongue piercings, no history of any condition involving chronic pain, not currently taking any prescription pain medication, no reported history of choking or difficulty swallowing and no history of thyroid disease. Here, we report data from 122 participants (44 men), with a mean age 27.7 (±7.89) years. Self-reported race and ethnicity was collected based on criteria provided by the 1997 OMB Directive 15. This population is largely of European ancestry (n=88), with marginal representation from other ancestry, African (n=2) and Asian (n=21), with 9 individuals choosing to not disclose their ancestry. | (45) |
| **TAS2R9** | rs3741845 | Candidate | A general labeled magnitude scale (gLMS) was used to collect perceived intensity of suprathreshold stimuli (AceK sweetness). This scale ranges from 0 (“no sensation”) to 100 (“the strongest imaginable sensation of any kind”), with intermediate descriptors at 1.4 (“barely detectable”), 6 (“weak”), 17 (“moderate”), 35 (“strong”), and 51 (“very strong”). | Eligibility criteria included the following: between 18 and 45 years old; not pregnant or breastfeeding; nonsmoker (had not smoked in the last 30 days); no known defects of smell or taste; no lip, cheek, or tongue piercings; no history Bitterness of Acesulfame Potassium Varies With TAS2R Polymorphisms 381 of any condition involving chronic pain; not currently taking any prescription pain medication; no reported history of choking or difficulty swallowing; and no history of thyroid disease. Participants also needed to be willing to provide a DNA sample via saliva. DNA samples were available from 147 participants. Race and ethnicity was self-reported using categories provided by the 1997 OMB Directive 15. To minimize potential population stratification, which can potentially cause false negatives and false positives in gene association studies (Hamer and Sirota 2000), individuals with Asian (n = 18), African (n = 5), or unknown (n = 15) ancestry were excluded from the present analyses. Thus, we report data from 108 participants (34 men) of European ancestry, with a mean age of 27.4 (±8.1 SD) years. Results were not substantively different in the mixed ancestry sample, but we report only the results for the European–American participants to facilitate interpretation of the linkage disequilibrium (LD) plots. | (8) |

**Supplementary Table 7 Study characteristics of genetic association studies related to fat taste preferences**

MUFA: monounsaturated fatty acid, PUFA: [polyunsaturated fatty acid, GWAS](https://en.wikipedia.org/wiki/Polyunsaturated_fatty_acid): genome-wide association study

**Supplementary Table 7 Study characteristics of genetic association studies related to fat taste preferences**

| **Gene** | **SNP** | **Discovery method** | **Phenotype assessment method** | **Study characteristics** | **Findings** | **Reference** | **Study characteristics (no association)** | **Reference (no association)** |
| --- | --- | --- | --- | --- | --- | --- | --- | --- |
| **CD36** | rs1761667 | Candidate | The threshold for oleic acid multimodal oral perception was assessed in each subject, in the absence of nose clips, by a modification of the staircase method implemented in a three-alternative forced-choice procedure, where stimuli were presented to subjects by means of filter paper disks (1.5 cm diameter). Filter paper disks were impregnated with 10 μL of a mixture of oleic acid and mineral oil, with oleic acid ranging from 0.0015 to 10 μL (pure). Each subject was presented with three samples: two contained only mineral oil (control) and one the amount of oleic acid under evaluation. The oleic acid concentration presented was increased after a single incorrect response and reduced after two correct responses in a row. A reversal was considered to have occurred at points where the concentration sequence changed direction. The procedure was terminated when four reversals occurred. The threshold concentration was calculated as the mean value of the four reversals. | Sixty-four non-smoking Caucasian subjects (23 males, 41 females, age 27.6 ± 0.85 years) from Sardinia, Italy were recruited. All were normal weight with a body mass index (BMI) ranging from 18.6 to 25.3 kg/m2, had maintained a stable weight in the previous. 3 months, and did not follow a diet or take medications that might interfere with taste function. Subjects with extreme scores for restraint and/or disinhibition and/or perceived hunger, assessed by theThree-Factor Eating Questionnaire, were excluded from the study. 36 for tested for sensitivity to oleic acid esterified with glycerol (triolein). | GG vs. AA linked to lower threshold for oleic acid. | (29) | 165 male and female adolescents wre recruited from Constantine district in Algeria. All the participants belonged to Arab-Berber ethnicity. The exclusion criteria for participants were any history of a chronic pathology such as cardiovascular disease, diabetes, liver, or kidney disease. The smokers were also excluded from the study. The alternative-forced choice (AFC) method was used for oleic acid sensitivity analysis. | (87) |
|  |  | Candidate | Taste emulsions containing food grade oleic acid were prepared. Taste preference tests for dietary lipids were performed by employing oleic acid at different ascending concentrations (0·018, 0·18, 0·37, 0·75, 1·5, 3, 6 and 12 mmol/l) by using a three-alternative forced choice (3-AFC) method. The threshold concentration was calculated as mean of log values for the last four reversals. | Obese women (n=203) were recruited from the group of patients who visited the gynaecology outpatient department of Farhat Hached University Hospital, Sousse (Tunisia), in 2012 and 2013 for a general health check-up. The studied women were between 38 and 43 years old.  The exclusion criteria included smoking, diabetes, breastfeeding, pregnancy-related complications, a history of gestational diabetes, the use of oral contraception, chronic illness such as hypertension or any other inflammatory pathology, any autoimmune disease, any lipid-lowering medication, recent weight loss, dieting and the use of any medications known to affect taste. The inclusion criterion constituted a normal glucose tolerance test and electrocardiograms. | GG vs. AA linked to lower threshold for oleic acid. | (88) |  |  |
|  |  | Candidate | Children underwent the OA (oleic acid) taste sensitivity analysis at different ascending concentrations of OA (0.018, 0.18, 0.37, 0.75, 1.5, 3, 6 and 12 mmol l − 1 ) as per three alternative-forced choice method. | One hundred sixteen (n = 116) school children, aged from 7 to 8 years, from Constantine district in Algeria by employing a multi-stage cluster random sampling method were recruited. The average age of the children was 8±0.5 years. The number of boys and girls was 57 and 59, respectively. | Threshold higher in A-allele (obese) children than in G-allele children. | (89) |  |  |
|  |  | Candidate | Participants completed taste testing studies on 2 separate days (day 1 and day 2) approximately 1 week apart. For 10 participants, fat taste perception was assessed in the presence of orlistat on day 1 and without orlistat, control day, on day 2. The remaining participants were assessed in the reverse order (i.e., control on day 1 and orlistat on day 2). The type of fat used as the fi rst taste stimuli to measure detection thresholds (i.e., oleic acid or triolein) was counterbalanced within the groups. | Twenty-one (females n=18,; males n=3; race: African American n=19, Caucasian n=2) obese subjects participated in this study. Potential subjects who smoked cigarettes in the last 6 months, had chronic sinus problems, previous malabsorptive or restrictive intestinal surgery, diabetes, or who were pregnant, breastfeeding, or taking any medication that might affect taste perception were excluded | G-allele carriers were more sensitive  in detecting oleic acid and triolein than subjects homozygous for the A-allele | (90) |  |  |
| **CD36** | rs1761667 | Candidate | Oral fat perception was assessed using Italian salad dressings prepared with varying amounts of canola oil. Participants were presented with the 5, 35, and 55% fat salad dressings, served in a randomized order in black. Participants self-reported acceptability of 83 fat containing foods as well. | Three hundred and seventeen (n = 317) African-American males (n = 137) and females (n = 180), ages 18–65 (mean ± SD = 35.5 ± 11.3), participated. Participants were recruited by placing advertisements on popular internet websites and through flyers posted around the hospitalstudy site. Screening for potential participants was conducted on the phone. Exclusion criteria were: food allergies; major medical conditions such as diabetes, hypertension, or diagnosed metabolic syndrome; recent weight loss or dieting; or use of any medications known to affect taste, body weight, or appetite. Participants were also excluded if they were not African-American, as defined by self-report of two African-American biological | AA vs. GA or GG perceived more creaminess (regardless of fat concentration), associated with acceptance of added fats and oils but no differences in perceived oiliness were reported. | (91) | - | - |
|  |  | Candidate | Participants were presented with four increasing oil (fat) content by-weight custards and low-fat/regular versions of commercially-available milk, mayonnaise and cream crackers. and then completed the perceived oiliness, perceived fat content and perceived creaminess ratings on a 170 mm visual analogue scale (VAS) anchored on the ends with “extremely low” and “extremely high”. Participants were instructed to mark a vertical line on the scale. Following the ratings, a ruler was used to measure the distance between the left anchor and the participant's response | Students were recruited of Universiti Tunku Abdul Rahman, Kampar Campus, Malaysia. The exclusion criteria included those with food allergies, major medical conditions such as diabetes, hypertension or diagnosed metabolic syndrome, recent weight loss or dieting, use of any medications known to affect taste, body weight or appetite, smoking more than one pack per week, chronic sinus problems, previous malabsorptive or restrictive intestinal surgery, pregnant and breastfeeding. The final sample comprised 313 healthy and unrelated subjects (male n = 118 mean age: 20.73 ± 1.55, ethnicity: Chinese n=113, Indian n=5; female n = 195, mean age: 20.74 ± 1.49, , ethnicity: Chinese n=180, Indian n=15 ) | AA lowest perceived ratings of fat content. | (92) | - | - |
| **CD36** | rs1527483 | Candidate | Oral fat perception was assessed using Italian salad dressings prepared with varying amounts of canola oil. Participants were presented with the 5, 35, and 55% fat salad dressings, served in a randomized order in black. | Three hundred and seventeen (n = 317) African-American males (n = 137) and females (n = 180), ages 18–65 (mean ± SD = 35.5 ± 11.3), participated. Participants were recruited by placing advertisements on popular internet websites and through flyers posted around the hospitalstudy site. Screening for potential participants was conducted on the phone. Exclusion criteria were: food allergies; major medical conditions such as diabetes, hypertension, or diagnosed metabolic syndrome; recent weight loss or dieting; or use of any medications known to affect taste, body weight, or appetite. Participants were also excluded if they were not African-American, as defined by self-report of two African-American biological parents. In addition, moderate to heavy smokers (defined as those smoking more than one pack per week) were excluded. | C/T or T/T perceived greater creaminess, oiliness and fat content of salad dressing. | (91) | Sixty-four non-smoking Caucasian subjects (23 males, 41 females, age 27.6 ± 0.85 years) from Sardinia, Italy were recruited. All were normal weight with a body mass index (BMI) ranging from 18.6 to 25.3 kg/m2, had maintained a stable weight in the previous. 3 months, and did not follow a diet or take medications that might interfere with taste function. Subjects with extreme scores for restraint and/or disinhibition and/or perceived hunger, assessed by theThree-Factor Eating Questionnaire, were excluded from the study. 36 for tested for sensitivity to oleic acid esterified with glycerol (triolein). | (29) |
|  |  | Candidate | Participants were presented with four increasing oil (fat) content by-weight custards and low-fat/regular versions of commercially-available milk, mayonnaise and cream crackers. and then completed the perceived oiliness, perceived fat content and perceived creaminess ratings on a 170 mm visual analogue scale (VAS) anchored on the ends with “extremely low” and “extremely high”. Participants were instructed to mark a vertical line on the scale. Following the ratings, a ruler was used to measure the distance between the left anchor and the participant's response. | Students were recruited of Universiti Tunku Abdul Rahman, Kampar Campus, Malaysia. The exclusion criteria included those with food allergies, major medical conditions such as diabetes, hypertension or diagnosed metabolic syndrome, recent weight loss or dieting, use of any medications known to affect taste, body weight or appetite, smoking more than one pack per week, chronic sinus problems, previous malabsorptive or restrictive intestinal surgery, pregnant and breastfeeding. The final sample comprised 313 healthy and unrelated subjects (male n = 118 mean age: 20.73 ± 1.55, ethnicity: Chinese n=113, Indian n=5; female n = 195, mean age: 20.74 ± 1.49, , ethnicity: Chinese n=180, Indian n=15 ) | C/T or T/T perceived greater creaminess, oiliness and fat content of crackers. | (92) |  |  |
| **IZUMO1** | rs838145 | Candidate | Profile of macronutrients was retreived from a validated semiquantitative 198-item FFQ (based on diet recall within the last month). | Genetic association studies were performed in the Danish population-based Inter99 study, which is a non-pharmacological intervention study for ischemic heart disease performed at the Research Centre for Prevention and Health, Glostrup, Denmark. A random sample of 13,016 individuals living in Copenhagen County from seven different age groups (30-60 years, grouped with five year intervals) was drawn from the Civil Registration System and 6,784 of these attended the health examination. All participants were Danes by self-report, and 6,514 individuals were eligible for genetic analyses. Subjects were between 18-39 years of age, with a BMI between 19-25 kg/m2 and without any medical conditions. A total of 86 subjects(23 men and 63 women) completed a questionnaire to determine taste preferences and each subject was asked to select images of liked and disliked snacks. | Tendency toward decreased total fat intake (A allele carriers), (MUFAs, PUFAs, omega-3 fatty acids). | (79) | - | - |
|  |  | GWAS | Macronutrients were assessed by using food-frequency questionnaires and analyzed as percentages of total energy consumption from total fat, protein, and carbohydrate. | A 2-stage genome-wide association (GWA) meta-analysis of macronutrient intake was performed in populations of European descent. | The variant associated with lower fat consumption. | (93) | - | - |

| **ADRB3** | rs4994 | Candidate | To assess food intake, the established FFQg available for clinical investigation was used. A trained dietitian inquired about the portion size and the frequency of food eaten for 1 week. | Fifty-two normal female subjects 21 or 22 y old (mean 21.6 y) were recruited for the study. The subjects were fourth-year university students. All were nonsmokers. The subjects’ height was 158.6 +/-0.5 cm, and their weight was 50.0 +/-6.9 kg. All subjects were non-obese (body mass index 19.8 2.1 kg/m2) and were found to be healthy at the annual medical checkups provided by the university. All subjects had been born and grew up in the vicinity of Tokyo or in the eastern part of Honshu Main Island. | T/C; TT vs. CC/TC linked to higher lipid intakes, including fatty acids and cholesterol. | (93) | - | - |
| --- | --- | --- | --- | --- | --- | --- | --- | --- |
| **APOA2** | rs5082 | Candidate | Dietary intake was estimated by using of the diet history questionnaire (DHQ). | The study sample consisted of 514 men and 564 women who participated in the Genetics of Lipid Lowering Drugs and Diet Network (GOLDN) study. GOLDN is part of the Program for Genetic Interactions Network and is funded by the NIH through the University of Alabama at Birmingham and in collaboration with the University of Utah, Washington University, Tufts University, University of Texas, University of Michigan, University of Minnesota, and Fairview-University of Minnesota Medical Center. The majority of participants were re-recruited from 3-generational pedigrees from 2 National Heart, Lung, and Blood Institute Family Heart Study field centers (Minneapolis, MN, and Salt Lake City, UT). Nearly all individuals were of European ancestry. Exclusion criteria were age <18 years; fasting triglycerides 16.5 mmol/L; recent history (6 months) of myocardial infarction; history of liver, kidney, pancreas, or gall bladder disease; history of malabsorption of nutrients; current use of insulin; abnormal renal or hepatic function; and pregnancy or nursing in women. Individuals who reported current use of hypolipidemic drugs or dietary supplements known to influence lipids were required to consult their physician for approval to discontinue these lipid-lowering agents for 4 weeks before study participation. The initially estimated sample size for the GOLDN study was 1200 individuals. Complete fasting and postprandial data were obtained from 1118 individuals. Individuals with inconsistent dietary data (total daily energy intake outside the range of 800 –5500 kcal in men or 600 – 4500 in women) were excluded, resulting in a final sample size of 1078 individuals (514 men and 564 women). Age, years, men 49.1 (16.1) women 48.1 (16.3) | CC vs TC/TT linked to higher total fat intake expressed as percentage of daily energy intake. | (94) | - | - |
| **OPRM1** | rs1799971 | Candidate | Three variables from the Food Preference Questionnaire were used in the analyses. This 72-item scale was designed as a 2 (FAT: high vs low) by 3 (CARBOHYDRATE: high simple, high complex, low carbohydrate/high protein) measure of preference for various kinds of macronutrients. Respondents indicate their preference for each food on a ninepoint Likert scale. The high fat preference score comprises the mean of 36 items (for example, onion rings and barbeque chicken); the high sugar preference score the mean of 24 items (for example, canned pears and dried dates); and the high fat and high sugar preference score the mean of 12 items (for example, chocolate layer cake and pecan pie) | Three hundred women (n = 238) and men (n =62) between the ages of 24 and 50 years took part in the study. Participants were required to be fluent in English and to have lived in North America for at least 5 years before their enrolment. All female participants were also pre-menopausal as identified by the self-reporting of regular menstrual cycles. Exclusion criteria included a current diagnosis of any psychotic disorder, substance abuse, alcoholism or a serious medical/physical illness, such as cancer, heart disease or paralysis. Female participants with a pregnancy within the previous 6 months were also ineligible. The sample comprised a broad range of BMI values, with a distribution representative of the general adult population. In all, 35% had a BMI of 25 kg m2 while the remainder was classified as overweight or obese according to the World Health Organization criteria. In total, 80% of the sample was Caucasian and 13% were of African descent. (Women mean age 33.5 SD=6.6, men mean age 35.2 SD=6.7) | GG stronger fat preferences. | (84) | - | - |
| **OPRM1** | rs495491 | Candidate | Three variables from the Food Preference Questionnaire were used in the analyses. This 72-item scale was designed as a 2 (FAT: high vs low) by 3 (CARBOHYDRATE: high simple, high complex, low carbohydrate/high protein) measure of preference for various kinds of macronutrients. Respondents indicate their preference for each food on a ninepoint Likert scale. The high fat preference score comprises the mean of 36 items (for example, onion rings and barbeque chicken); the high sugar preference score the mean of 24 items (for example, canned pears and dried dates); and the high fat and high sugar preference score the mean of 12 items (for example, chocolate layer cake and pecan pie) | Three hundred women (n = 238) and men (n =62) between the ages of 24 and 50 years took part in the study. Participants were required to be fluent in English and to have lived in North America for at least 5 years before their enrolment. All female participants were also pre-menopausal as identified by the self-reporting of regular menstrual cycles. Exclusion criteria included a current diagnosis of any psychotic disorder, substance abuse, alcoholism or a serious medical/physical illness, such as cancer, heart disease or paralysis. Female participants with a pregnancy within the previous 6 months were also ineligible. The sample comprised a broad range of BMI values, with a distribution representative of the general adult population. In all, 35% had a BMI of 25 kg m2 while the remainder was classified as overweight or obese according to the World Health Organization criteria. In total, 80% of the sample was Caucasian and 13% were of African descent. (Women mean age 33.5 SD=6.6, men mean age 35.2 SD=6.7) | GG vs. GA and AA reported lower fat preference. | (84) | - | - |
| **RGS6** | rs847330 | Candidate | A food frequency questionnaire was administered by interview at the follow-up visit. This questionnaire contains a 70-item food list. | The IRASFS was designed to explore genetic and epidemiologic contributions to abdominal adiposity and glucose homeostasis traits among Hispanic and African Americans using a family-based design. Large families were recruited from 2000 to 2002 at study centers in San Antonio, TX (Hispanics); San Luis Valley, CO (Hispanics); and Los Angeles, CA (African Americans); with probands identified from both the parent study IRAS as well as the general population. Families were recruited based upon family size, not disease or phenotype status. A follow-up examination was conducted ~5 years after the baseline examination, during which dietary intake data were collected. As the RGS6 genotyping was only done in Hispanics the current study only includes the Hispanic cohort of the IRASFS. Individuals with diabetes remained in our dataset for analysis. Total (n) 932;  Gender (% female) 61.9%;  Age (years) 47.8 ± 14.1;  BMI (kg/m2) 29.8 ± 6.2. | Associated with the frequency of servings of fats/oils/sweets and modestly associated with total fat and saturated fat intake. | (95) | - | - |
| **RGS6** | rs847328 | Candidate | A food frequency questionnaire was administered by interview at the follow-up visit. This questionnaire contains a 70-item food list. | The IRASFS was designed to explore genetic and epidemiologic contributions to abdominal adiposity and glucose homeostasis traits among Hispanic and African Americans using a family-based design. Large families were recruited from 2000 to 2002 at study centers in San Antonio, TX (Hispanics); San Luis Valley, CO (Hispanics); and Los Angeles, CA (African Americans); with probands identified from both the parent study IRAS as well as the general population. Families were recruited based upon family size, not disease or phenotype status. A follow-up examination was conducted ~5 years after the baseline examination, during which dietary intake data were collected. As the RGS6 genotyping was only done in Hispanics the current study only includes the Hispanic cohort of the IRASFS. Individuals with diabetes remained in our dataset for  analysis. Total (n) 932;  Gender (% female) 61.9%;  Age (years) 47.8 ± 14.1;  BMI (kg/m2) 29.8 ± 6.2. | Associated with the frequency of servings of fats/oils/sweets. | (95) | - | - |
| **RGS6** | rs769148 | Candidate | A food frequency questionnaire was administered by interview at the follow-up visit. This questionnaire contains a 70-item food list. | The IRASFS was designed to explore genetic and epidemiologic contributions to abdominal adiposity and glucose homeostasis traits among Hispanic and African Americans using a family-based design. Large families were recruited from 2000 to 2002 at study centers in San Antonio, TX (Hispanics); San Luis Valley, CO (Hispanics); and Los Angeles, CA (African Americans); with probands identified from both the parent study IRAS as well as the general population. Families were recruited based upon family size, not disease or phenotype status. A follow-up examination was conducted ~5 years after the baseline examination, during which dietary intake data were collected. As the RGS6 genotyping was only done in Hispanics the current study only includes the Hispanic cohort of the IRASFS. Individuals with diabetes remained in our dataset for  analysis. Total (n) 932;  Gender (% female) 61.9%;  Age (years) 47.8 ± 14.1;  BMI (kg/m2) 29.8 ± 6.2. | Associated with the frequency of servings of fats/oils/sweets. | (95) | - | - |
| **AGT** | rs699 | Candidate | To assess food intake, the established FFQg available for clinical investigation was used. A trained dietitian inquired about the portion size and the frequency of food eaten for 1 week. | Fifty-two normal female subjects 21 or 22 y old (mean 21.6 y) were recruited for the study. The subjects were fourth-year university students. All were nonsmokers. The subjects’ height was 158.6 +/-0.5 cm, and their weight was 50.0 +/-6.9 kg. All subjects were non-obese (body mass index 19.8 2.1 kg/m2) and were found to be healthy at the annual medical checkups provided by the university. All subjects had been born and grew up in the vicinity of Tokyo or in the eastern part of Honshu Main Island. | MM/MT vs. TT associated with higher intake of total lipids. | (93) | - | - |
| **BPNT1** | rs6661761 | GWAS | Liking/disliking for 20 foods were ascertained through a questionnaire administered by an operator. The foods can be conceptually grouped into 4 categories: vegetables (artichokes, broccoli, chicory, spinach, and mushrooms), fatty (bacon ham, lamb meat, oil or butter on bread, and pork chops), dairy (goat cheese, blue cheese, ice cream, mozzarella, plain yogurt and whole milk) and bitter (dark beer, dark chocolate, liver and coffee).  Participants were asked to rate their liking for each food on a scale ranging from 1 (dislike extremely) to 9 (like extremely) or to indicate never having tasted the particular food. The SR population was in excepption because a 5-point facial hedonic scale was used. | Participants have been collected from Europe and Central Asia, including: 381 individuals from INGI-CARL recruited from Carlantino, a small village located in Puglia (Southern Italy); 744 from INGI-FVG, recruited from 6 villages situated in the Friuli-Venezia Region in Northern-Eastern Italy, and 1115 from INGI-VB, recruited from the Val Borbera Valley in Northern-Western Italy. DNA from 1261 individuals was collected in the Erasmus Rucphen Family (ERF) study, a cross-sectional cohort including 3000 living descendants of 22 couples who had at least 6 children baptized in the community church around 1850–1900. Finally, DNA samples from 335 individuals was obtained from the Silk Road (SR) cohort of ~1000 individuals from 20 communities located along the Silk Road (Armenia, Azerbaijan, Georgia, Uzbekistan, Tajikistan and Kazakhstan). | Association with oil or butter on bread liking. | (47) | - | - |
| **CNR1** | rs1049353 | Candidate | Validated semi-quantitative food frequency questionnaire (FFQ, self-reported): intake of 124 foods and drinks clustered into 96 food items, organized into 12 sections of homogeneous food groups, considering caloric contribution and nutritional supply, consumed during the 12 months before enrolment in the study. | Study Design and Patients This work is part of the MICOL study, a 25-year longitudinal population study on gallstones, which began in 1985 in a small town in southern Italy (Castellana, Province of Bari, in the Apulia region). Briefly, between May 1985 and June 1986, 3,500 individuals (2,000 men and 1,500 women aged 30–69 years) were randomly selected from the electoral register of the town (the register includes all residents of the town over 18 years of age, and therefore old enough to vote). Of these, 2,472 subjects (70.6% respondence rate, 1,429 men and 1,043 women) participated in the survey. Between May 1992 and June 1993, 2,175 of these subjects were re-examined (88% respondence rate). After agreeing to participate, all subjects were asked to complete a validated semi-quantitative food frequency questionnaire (FFQ) and to respond to questions on sociodemographic variables, lifestyle, and medical history all reported in the previous year, and underwent standardized measurement of height, weight, and blood pressure. In this report, we present data obtained from a random subsample of 118 elderly subjects at the second examination (1992–1993), already previously examined for the CB1-R 1359 G/A polymorphism, who have filled out their FFQ. Allsubjects were older than 65 years and younger than 75 years. | The SNP inversely associated with the intake of dietary cholesterol and saturated fats. | (59) | - | - |
| **CNTN5** | rs140738262 | GWAS | Liking/disliking for 20 foods were ascertained through a questionnaire administered by an operator. The foods can be conceptually grouped into 4 categories: vegetables (artichokes, broccoli, chicory, spinach, and mushrooms), fatty (bacon ham, lamb meat, oil or butter on bread, and pork chops), dairy (goat cheese, blue cheese, ice cream, mozzarella, plain yogurt and whole milk) and bitter (dark beer, dark chocolate, liver and coffee).  Participants were asked to rate their liking for each food on a scale ranging from 1 (dislike extremely) to 9 (like extremely) or to indicate never having tasted the particular food. The SR population was in excepption because a 5-point facial hedonic scale was used. | Participants have been collected from Europe and Central Asia, including: 381 individuals from INGI-CARL recruited from Carlantino, a small village located in Puglia (Southern Italy); 744 from INGI-FVG, recruited from 6 villages situated in the Friuli-Venezia Region in Northern-Eastern Italy, and 1115 from INGI-VB, recruited from the Val Borbera Valley in Northern-Western Italy. DNA from 1261 individuals was collected in the Erasmus Rucphen Family (ERF) study, a cross-sectional cohort including 3000 living descendants of 22 couples who had at least 6 children baptized in the community church around 1850–1900. Finally, DNA samples from 335 individuals was obtained from the Silk Road (SR) cohort of ~1000 individuals from 20 communities located along the Silk Road (Armenia, Azerbaijan, Georgia, Uzbekistan, Tajikistan and Kazakhstan). | Association with bacon liking. | (47) | - | - |
| **FGF21** | rs838133 | Candidate | Profile of macronutrients was retreived from a validated semiquantitative 198-item FFQ (based on diet recall within the last month). | Genetic association studies were performed in the Danish population-based Inter99 study, which is a non-pharmacological intervention study for ischemic heart disease performed at the Research Centre for Prevention and Health, Glostrup, Denmark. A random sample of 13,016 individuals living in Copenhagen County from seven different age groups (30-60 years, grouped with five year intervals) was drawn from the Civil Registration System and 6,784 of these attended the health examination. All participants were Danes by self-report, and 6,514 individuals were eligible for genetic analyses. Subjects were between 18-39 years of age, with a BMI between 19-25 kg/m2 and without any medical conditions. A total of 86 subjects(23 men and 63 women) completed a questionnaire to determine taste preferences and each subject was asked to select images of liked and disliked snacks. | Tendency toward decreased total fat intake (A allele carriers), (MUFAs, PUFAs, omega-3 fatty acids). | (79) | - | - |
| **OXTR** | rs2268494 | Candidate | Food Preference Questionnaire: This questionnaire provides hedonic ratings for 72 common food items arranged according to a 2 (FAT: high vs low) x 3 (OTHER MACRONUTRIENT: high simple sugar, high complex carbohydrate, and high protein) matrix with 12 items in each cell. In the current study, High Sugar and High Fat Foods is reflected by the 12 items from the high fat/high simple sugar cell; High Sugar Foods includes the 12 items from the High Simple Sugar/Low Fat cell and the High Fat Foods is the total (24 items) of the High Fat/High Carbohydrate and High Fat/High Protein subscales. The 72 food items of the Food Preference Questionnaire vary systematically and significantly with respect to their macronutrient content. | The sample consisted of 460 adults (females = 346) between 24 and 50 years of age. Inclusion criteria were residence in North America for at least 5 years before enrollment in the study, and fluency in written and spoken English. Women were also required to be premenopausal as indicated by the self-report of a regular menstrual cycle. Exclusion criteria included serious medical illnesses such as cancer or diabetes, or severe physical disabilities such as cerebral palsy. Those with a current axis I diagnosed disorder, with the exception of unipolar depression, were also excluded. In addition, women with a pregnancy in the previous 6 months, or who were lactating, were not included. The majority of the sample was Caucasian (79%), with 15% identifying as African descent and the remainder as ‘Other’. The sample represented a broad range of body mass index (BMI) values (17.8–75.2 kg m2). Participants comprised a community-based sample and were recruited from posters, newspaper advertisements and online sites such as Craigslist and Kijiji in a large Canadian city. Females age 33.4 (SD= 6.6); males age 34.3 (SD=7.0) | A allele carriers stronger preference for fatty foods. | (85) | - | - |
| **RGS6** | rs847354 | Candidate | A food frequency questionnaire was administered by interview at the follow-up visit. This questionnaire contains a 70-item food list. | The IRASFS was designed to explore genetic and epidemiologic contributions to abdominal adiposity and glucose homeostasis traits among Hispanic and African Americans using a family-based design. Large families were recruited from 2000 to 2002 at study centers in San Antonio, TX (Hispanics); San Luis Valley, CO (Hispanics); and Los Angeles, CA (African Americans); with probands identified from both the parent study IRAS as well as the general population. Families were recruited based upon family size, not disease or phenotype status. A follow-up examination was conducted ~5 years after the baseline examination, during which dietary intake data were collected. As the RGS6 genotyping was only done in Hispanics the current study only includes the Hispanic cohort of the IRASFS. Individuals with diabetes remained in our dataset for  analysis. Total (n) 932;  Gender (% female) 61.9%;  Age (years) 47.8 ± 14.1;  BMI (kg/m2) 29.8 ± 6.2. | Modestly associated with intake frequency of fats/oils/sweets, total fat and saturated fat intake. | (95) | - | - |
| **RGS6** | rs2239247 | Candidate | A food frequency questionnaire was administered by interview at the follow-up visit. This questionnaire contains a 70-item food list. | The IRASFS was designed to explore genetic and epidemiologic contributions to abdominal adiposity and glucose homeostasis traits among Hispanic and African Americans using a family-based design. Large families were recruited from 2000 to 2002 at study centers in San Antonio, TX (Hispanics); San Luis Valley, CO (Hispanics); and Los Angeles, CA (African Americans); with probands identified from both the parent study IRAS as well as the general population. Families were recruited based upon family size, not disease or phenotype status. A follow-up examination was conducted ~5 years after the baseline examination, during which dietary intake data were collected. As the RGS6 genotyping was only done in Hispanics the current study only includes the Hispanic cohort of the IRASFS. Individuals with diabetes remained in our dataset for  analysis. Total (n) 932;  Gender (% female) 61.9%;  Age (years) 47.8 ± 14.1;  BMI (kg/m2) 29.8 ± 6.2. | Associated with intake frequency of fats/oils/ sweets (servings/day). | (95) | 0 | - |
| **RGS6** | rs1402064 | Candidate | A food frequency questionnaire was administered by interview at the follow-up visit. This questionnaire contains a 70-item food list. | The IRASFS was designed to explore genetic and epidemiologic contributions to abdominal adiposity and glucose homeostasis traits among Hispanic and African Americans using a family-based design. Large families were recruited from 2000 to 2002 at study centers in San Antonio, TX (Hispanics); San Luis Valley, CO (Hispanics); and Los Angeles, CA (African Americans); with probands identified from both the parent study IRAS as well as the general population. Families were recruited based upon family size, not disease or phenotype status. A follow-up examination was conducted ~5 years after the baseline examination, during which dietary intake data were collected. As the RGS6 genotyping was only done in Hispanics the current study only includes the Hispanic cohort of the IRASFS. Individuals with diabetes remained in our dataset for  analysis. Total (n) 932;  Gender (% female) 61.9%;  Age (years) 47.8 ± 14.1;  BMI (kg/m2) 29.8 ± 6.2. | Associated with the intake frequency of servings of fats/oils/sweets and total fat intake, saturated fat intake, percent of calories from fat. | (95) | 0 | - |
| **SH2B1** | rs7498665 | Candidate | The food frequency questionnaire contained questions on the usual frequency of consumption of 77 main food items. | The study population consisted of Dutch female participants in the European Prospective Investigation into Cancer and Nutrition (EPIC), conducted in Utrecht, Netherlands (Prospect-EPIC). Between 1993 and 1997, 17,357 women aged 49–70 y and residing in or near Utrecht were recruited through a regional, population-based, breast cancer screening program. At recruitment, each participant filled in a general questionnaire on lifestyle factors, gynecologic and obstetric history, and past and current morbidity as well as a validated, semiquantitative, food-frequency questionnaire (FFQ) with the aim of capturing the habitual diet during the year preceding enrollment. A random sample of 1736 (10%) women was selected for biochemical analyses. Buffy coat samples were missing for 36 women; therefore, our study population comprised 1700 women. For the analyses of energy and macronutrient intakes, we exclude women who did not fill in the dietary questionnaire (n = 11). In addition, we excluded women with an implausibly low total energy intake of ,800 kcal/d (n = 9). Age (y) 57.22 +/- 6.06. | Associated with fat and saturated fat intakes. | (96) | 0 | - |
| **TAS2R38** | A49P (rs713598), A262V (rs1726866), V296I (rs10246939) | Candidate | The threshold for oleic acid multimodal oral perception was assessed in each subject, in the absence of nose clips, by a modification of the staircase method implemented in a three-alternative forced-choice procedure, where stimuli were presented to subjects by means of filter paper disks (1.5 cm diameter). Filter paper disks were impregnated with 10 μL of a mixture of oleic acid and mineral oil, with oleic acid ranging from 0.0015 to 10 μL (pure). Each subject was presented with three samples: two contained only mineral oil (control) and one the amount of oleic acid under evaluation. The oleic acid concentration presented was increased after a single incorrect response and reduced after two correct responses in a row. A reversal was considered to have occurred at points where the concentration sequence changed direction. The procedure was terminated when four reversals occurred. The threshold concentration was calculated as the mean value of the four reversals. | Sixty-four non-smoking Caucasian subjects (23 males, 41 females, age 27.6 ± 0.85 years) from Sardinia, Italy were recruited. All were normal weight with a body mass index (BMI) ranging from 18.6 to 25.3 kg/m2, had maintained a stable weight in the previous. 3 months, and did not follow a diet or take medications that might interfere with taste function. Subjects with extreme scores for restraint and/or disinhibition and/or perceived hunger, assessed by theThree-Factor Eating Questionnaire, were excluded from the study. 36 for tested for sensitivity to oleic acid esterified with glycerol (triolein). | In interaction with rs1761667 and rs1527483. | (29) | 0 | - |

**Supplementary Table 8 Study characteristics of genetic association studies related to fat taste preferences (no associations)**

**Supplementary Table 8 Study characteristics of genetic association studies related to fat taste preferences (no associations)**

| **Gene** | **SNP** | **Discovery method** | **Phenotype assessment method** | **Study population characteristics** | **Reference (no association)** |
| --- | --- | --- | --- | --- | --- |
| **ADIPOQ-AS1** | rs2241766 | Candidate | Native 7-day food records were collected. | Czech Caucasians (n=138). | (97) |
| **BDNF-AS** | rs1488830 | Candidate | The food frequency questionnaire contained questions on the usual frequency of consumption of 77 main food items. | The study population consisted of Dutch female participants in the European Prospective Investigation into Cancer and Nutrition (EPIC), conducted in Utrecht, Netherlands (Prospect-EPIC). Between 1993 and 1997, 17,357 women aged 49–70 y and residing in or near Utrecht were recruited through a regional, population-based, breast cancer screening program. At recruitment, each participant filled in a general questionnaire on lifestyle factors, gynecologic and obstetric history, and past and current morbidity as well as a validated, semiquantitative, food-frequency questionnaire (FFQ) with the aim of capturing the habitual diet during the year preceding enrollment. A random sample of 1736 (10%) women was selected for biochemical analyses. Buffy coat samples were missing for 36 women; therefore, our study population comprised 1700 women. For the analyses of energy and macronutrient intakes, we exclude women who did not fill in the dietary questionnaire (n = 11). In addition, we excluded women with an implausibly low total energy intake of ,800 kcal/d (n = 9). Age (y) 57.22 +/- 6.06. | (96) |
| **BDNF-AS** | rs925946 | Candidate | The food frequency questionnaire contained questions on the usual frequency of consumption of 77 main food items. | The study population consisted of Dutch female participants in the European Prospective Investigation into Cancer and Nutrition (EPIC), conducted in Utrecht, Netherlands (Prospect-EPIC). Between 1993 and 1997, 17,357 women aged 49–70 y and residing in or near Utrecht were recruited through a regional, population-based, breast cancer screening program. At recruitment, each participant filled in a general questionnaire on lifestyle factors, gynecologic and obstetric history, and past and current morbidity as well as a validated, semiquantitative, food-frequency questionnaire (FFQ) with the aim of capturing the habitual diet during the year preceding enrollment. A random sample of 1736 (10%) women was selected for biochemical analyses. Buffy coat samples were missing for 36 women; therefore, our study population comprised 1700 women. For the analyses of energy and macronutrient intakes, we exclude women who did not fill in the dietary questionnaire (n = 11). In addition, we excluded women with an implausibly low total energy intake of ,800 kcal/d (n = 9). Age (y) 57.22 +/- 6.06. | (96) |
| **CAV3** | rs237878 | Candidate | Food Preference Questionnaire: This questionnaire provides hedonic ratings for 72 common food items arranged according to a 2 (FAT: high vs low) x 3 (OTHER MACRONUTRIENT: high simple sugar, high complex carbohydrate, and high protein) matrix with 12 items in each cell. In the current study, High Sugar and High Fat Foods is reflected by the 12 items from the high fat/high simple sugar cell; High Sugar Foods includes the 12 items from the High Simple Sugar/Low Fat cell and the High Fat Foods is the total (24 items) of the High Fat/High Carbohydrate and High Fat/High Protein subscales. The 72 food items of the Food Preference Questionnaire vary systematically and significantly with respect to their macronutrient content. | The sample consisted of 460 adults (females = 346) between 24 and 50 years of age. Inclusion criteria were residence in North America for at least 5 years before enrollment in the study, and fluency in written and spoken English. Women were also required to be premenopausal as indicated by the self-report of a regular menstrual cycle. Exclusion criteria included serious medical illnesses such as cancer or diabetes, or severe physical disabilities such as cerebral palsy. Those with a current axis I diagnosed disorder, with the exception of unipolar depression, were also excluded. In addition, women with a pregnancy in the previous 6 months, or who were lactating, were not included. The majority of the sample was Caucasian (79%), with 15% identifying as African descent and the remainder as ‘Other’. The sample represented a broad range of body mass index (BMI) values (17.8–75.2 kg m2). Participants comprised a community-based sample and were recruited from posters, newspaper advertisements and online sites such as Craigslist and Kijiji in a large Canadian city. Females age 33.4 (SD= 6.6); males age 34.3 (SD=7.0) | (85) |
| **CD36** | rs1984112 | Candidate | Frequency of consumption of foods comprising four areas of interest was assessed using a food frequency questionnaire (FFQ). | Participants were between 18 and 30 years old, non-obese (BMI ⩽30 kg/m2), non-smokers who were physically active. A total of 56 participants (28 males, 28 females; 24.9 ± 3.3 years; BMI = 23.7 ± 2.7 kg/m2) volunteered for the study from November 2014 to June 2015. | (98) |
| **CD36** | rs1984112 | Candidate | Oral fat perception was assessed using Italian salad dressings prepared with varying amounts of canola oil. Participants were presented with the 5, 35, and 55% fat salad dressings, served in a randomized order in black. Participants self-reported acceptability of 83 fat containing foods as well. | Three hundred and seventeen (n = 317) African-American males (n = 137) and females (n = 180), ages 18–65 (mean ± SD = 35.5 ± 11.3), participated. Participants were recruited by placing advertisements on popular internet websites and through flyers posted around the hospitalstudy site. Screening for potential participants was conducted on the phone. Exclusion criteria were: food allergies; major medical conditions such as diabetes, hypertension, or diagnosed metabolic syndrome; recent weight loss or dieting; or use of any medications known to affect taste, body weight, or appetite. Participants were also excluded if they were not African-American, as defined by self-report of two African-American biological | (99) |
| **CD36** | rs1527479 | Candidate | Frequency of consumption of foods comprising four areas of interest was assessed using a food frequency questionnaire (FFQ). | Participants were between 18 and 30 years old, non-obese (BMI ⩽30 kg/m2), non-smokers who were physically active. A total of 56 participants (28 males, 28 females; 24.9 ± 3.3 years; BMI = 23.7 ± 2.7 kg/m2) volunteered for the study from November 2014 to June 2015. | (98) |
| **CD36** | rs1049673 | Candidate | Oral fat perception was assessed using Italian salad dressings prepared with varying amounts of canola oil. Participants were presented with the 5, 35, and 55% fat salad dressings, served in a randomized order in black. Participants self-reported acceptability of 83 fat containing foods as well. | Three hundred and seventeen (n = 317) African-American males (n = 137) and females (n = 180), ages 18–65 (mean ± SD = 35.5 ± 11.3), participated. Participants were recruited by placing advertisements on popular internet websites and through flyers posted around the hospitalstudy site. Screening for potential participants was conducted on the phone. Exclusion criteria were: food allergies; major medical conditions such as diabetes, hypertension, or diagnosed metabolic syndrome; recent weight loss or dieting; or use of any medications known to affect taste, body weight, or appetite. Participants were also excluded if they were not African-American, as defined by self-report of two African-American biological | (99) |
| **CD36** | rs3840546 | Candidate | Oral fat perception was assessed using Italian salad dressings prepared with varying amounts of canola oil. Participants were presented with the 5, 35, and 55% fat salad dressings, served in a randomized order in black. Participants self-reported acceptability of 83 fat containing foods as well. | Three hundred and seventeen (n = 317) African-American males (n = 137) and females (n = 180), ages 18–65 (mean ± SD = 35.5 ± 11.3), participated. Participants were recruited by placing advertisements on popular internet websites and through flyers posted around the hospitalstudy site. Screening for potential participants was conducted on the phone. Exclusion criteria were: food allergies; major medical conditions such as diabetes, hypertension, or diagnosed metabolic syndrome; recent weight loss or dieting; or use of any medications known to affect taste, body weight, or appetite. Participants were also excluded if they were not African-American, as defined by self-report of two African-American biological | (96) |
| **FTO** | rs1121980 | Candidate | The food frequency questionnaire contained questions on the usual frequency of consumption of 77 main food items. | The study population consisted of Dutch female participants in the European Prospective Investigation into Cancer and Nutrition (EPIC), conducted in Utrecht, Netherlands (Prospect-EPIC). Between 1993 and 1997, 17,357 women aged 49–70 y and residing in or near Utrecht were recruited through a regional, population-based, breast cancer screening program. At recruitment, each participant filled in a general questionnaire on lifestyle factors, gynecologic and obstetric history, and past and current morbidity as well as a validated, semiquantitative, food-frequency questionnaire (FFQ) with the aim of capturing the habitual diet during the year preceding enrollment. A random sample of 1736 (10%) women was selected for biochemical analyses. Buffy coat samples were missing for 36 women; therefore, our study population comprised 1700 women. For the analyses of energy and macronutrient intakes, we exclude women who did not fill in the dietary questionnaire (n = 11). In addition, we excluded women with an implausibly low total energy intake of ,800 kcal/d (n = 9). Age (y) 57.22 +/- 6.06. | (96) |
| **FTO** | rs17782313 | Candidate | The food frequency questionnaire contained questions on the usual frequency of consumption of 77 main food items. | The study population consisted of Dutch female participants in the European Prospective Investigation into Cancer and Nutrition (EPIC), conducted in Utrecht, Netherlands (Prospect-EPIC). Between 1993 and 1997, 17,357 women aged 49–70 y and residing in or near Utrecht were recruited through a regional, population-based, breast cancer screening program. At recruitment, each participant filled in a general questionnaire on lifestyle factors, gynecologic and obstetric history, and past and current morbidity as well as a validated, semiquantitative, food-frequency questionnaire (FFQ) with the aim of capturing the habitual diet during the year preceding enrollment. A random sample of 1736 (10%) women was selected for biochemical analyses. Buffy coat samples were missing for 36 women; therefore, our study population comprised 1700 women. For the analyses of energy and macronutrient intakes, we exclude women who did not fill in the dietary questionnaire (n = 11). In addition, we excluded women with an implausibly low total energy intake of ,800 kcal/d (n = 9). Age (y) 57.22 +/- 6.06. | (96) |
| **GHRL** | rs696217 | Candidate | Participants were advised to complete specific selfadministered demographic questionnaires along with 7-day food records. | A total of 185 unrelated Czech Caucasian individuals were recruited for this case-control study and were divided in two groups. All the participants in the study, both obese individuals and the lean controls, were recruited in a mass media campaign addressing the population of the south Moravia region in the Czech Republic. Both the cases and the controls underwent the same diagnostic procedures to avoid possible selection bias. In recruitment, the inclusion and exclusion criteria of Ma et al. were used. The case group consisted of 125 obese individuals (body mass index [BMI] ≥ 30 kg/m2; mean BMI, 37.7 ± 6.38 kg/m2: median age, 50.0 years; range, 18.6–68.9 years). In this group of obese individuals, a subset of 34 morbidly obese patients was identified using the criterion of BMI ≥ 40 kg/m2 (mean BMI, 45.5 ± 3.6 kg/m2: median age, 50.5 years; range, 18.6–67.8 years); all of these 34 patients were available for the plasma leptin and sObR determinations. The control group consisted of 60 healthy, normal-weight, control subjects with no history of childhood obesity (mean BMI, 25.8 ± 3.3 kg/m2: median age, 47.5 years; range, 18.1–67.5 years). Data on personal or family history of obesity, birth weight, age at onset of obesity, eating disorders, age of menarche and menopause in women, family history of sterility, infertility or stillbirth were obtained by a professional using a semi-structured interview. A positive family history for obesity was estimated as one obese relative (BMI ≥ 30 kg/m2 in the close family – siblings, parents and their siblings and grandparents). Both the obese cases and the controls underwent the same examinations on their anthropometric characteristics, dietary intake and genetic background of the individual and the family. | (81) |
| **LEPR** | rs1137101 | Candidate | Participants were advised to complete specific selfadministered demographic questionnaires along with 7-day food records. | A total of 185 unrelated Czech Caucasian individuals were recruited for this case-control study and were divided in two groups. All the participants in the study, both obese individuals and the lean controls, were recruited in a mass media campaign addressing the population of the south Moravia region in the Czech Republic. Both the cases and the controls underwent the same diagnostic procedures to avoid possible selection bias. In recruitment, the inclusion and exclusion criteria of Ma et al. were used. The case group consisted of 125 obese individuals (body mass index [BMI] ≥ 30 kg/m2; mean BMI, 37.7 ± 6.38 kg/m2: median age, 50.0 years; range, 18.6–68.9 years). In this group of obese individuals, a subset of 34 morbidly obese patients was identified using the criterion of BMI ≥ 40 kg/m2 (mean BMI, 45.5 ± 3.6 kg/m2: median age, 50.5 years; range, 18.6–67.8 years); all of these 34 patients were available for the plasma leptin and sObR determinations. The control group consisted of 60 healthy, normal-weight, control subjects with no history of childhood obesity (mean BMI, 25.8 ± 3.3 kg/m2: median age, 47.5 years; range, 18.1–67.5 years). Data on personal or family history of obesity, birth weight, age at onset of obesity, eating disorders, age of menarche and menopause in women, family history of sterility, infertility or stillbirth were obtained by a professional using a semi-structured interview. A positive family history for obesity was estimated as one obese relative (BMI ≥ 30 kg/m2 in the close family – siblings, parents and their siblings and grandparents). Both the obese cases and the controls underwent the same examinations on their anthropometric characteristics, dietary intake and genetic background of the individual and the family. | (81) |
| **LOC105375494** | rs7799039 | Candidate | Participants were advised to complete specific selfadministered demographic questionnaires along with 7-day food records. | A total of 185 unrelated Czech Caucasian individuals were recruited for this case-control study and were divided in two groups. All the participants in the study, both obese individuals and the lean controls, were recruited in a mass media campaign addressing the population of the south Moravia region in the Czech Republic. Both the cases and the controls underwent the same diagnostic procedures to avoid possible selection bias. In recruitment, the inclusion and exclusion criteria of Ma et al. were used. The case group consisted of 125 obese individuals (body mass index [BMI] ≥ 30 kg/m2; mean BMI, 37.7 ± 6.38 kg/m2: median age, 50.0 years; range, 18.6–68.9 years). In this group of obese individuals, a subset of 34 morbidly obese patients was identified using the criterion of BMI ≥ 40 kg/m2 (mean BMI, 45.5 ± 3.6 kg/m2: median age, 50.5 years; range, 18.6–67.8 years); all of these 34 patients were available for the plasma leptin and sObR determinations. The control group consisted of 60 healthy, normal-weight, control subjects with no history of childhood obesity (mean BMI, 25.8 ± 3.3 kg/m2: median age, 47.5 years; range, 18.1–67.5 years). Data on personal or family history of obesity, birth weight, age at onset of obesity, eating disorders, age of menarche and menopause in women, family history of sterility, infertility or stillbirth were obtained by a professional using a semi-structured interview. A positive family history for obesity was estimated as one obese relative (BMI ≥ 30 kg/m2 in the close family – siblings, parents and their siblings and grandparents). Both the obese cases and the controls underwent the same examinations on their anthropometric characteristics, dietary intake and genetic background of the individual and the family. | (81) |
| **LOC105378797** | rs2568958 | Candidate | The food frequency questionnaire contained questions on the usual frequency of consumption of 77 main food items. | The study population consisted of Dutch female participants in the European Prospective Investigation into Cancer and Nutrition (EPIC), conducted in Utrecht, Netherlands (Prospect-EPIC). Between 1993 and 1997, 17,357 women aged 49–70 y and residing in or near Utrecht were recruited through a regional, population-based, breast cancer screening program. At recruitment, each participant filled in a general questionnaire on lifestyle factors, gynecologic and obstetric history, and past and current morbidity as well as a validated, semiquantitative, food-frequency questionnaire (FFQ) with the aim of capturing the habitual diet during the year preceding enrollment. A random sample of 1736 (10%) women was selected for biochemical analyses. Buffy coat samples were missing for 36 women; therefore, our study population comprised 1700 women. For the analyses of energy and macronutrient intakes, we exclude women who did not fill in the dietary questionnaire (n = 11). In addition, we excluded women with an implausibly low total energy intake of ,800 kcal/d (n = 9). Age (y) 57.22 +/- 6.06. | (96) |
| **MTCH2** | rs10838738 | Candidate | The food frequency questionnaire contained questions on the usual frequency of consumption of 77 main food items. | The study population consisted of Dutch female participants in the European Prospective Investigation into Cancer and Nutrition (EPIC), conducted in Utrecht, Netherlands (Prospect-EPIC). Between 1993 and 1997, 17,357 women aged 49–70 y and residing in or near Utrecht were recruited through a regional, population-based, breast cancer screening program. At recruitment, each participant filled in a general questionnaire on lifestyle factors, gynecologic and obstetric history, and past and current morbidity as well as a validated, semiquantitative, food-frequency questionnaire (FFQ) with the aim of capturing the habitual diet during the year preceding enrollment. A random sample of 1736 (10%) women was selected for biochemical analyses. Buffy coat samples were missing for 36 women; therefore, our study population comprised 1700 women. For the analyses of energy and macronutrient intakes, we exclude women who did not fill in the dietary questionnaire (n = 11). In addition, we excluded women with an implausibly low total energy intake of ,800 kcal/d (n = 9). Age (y) 57.22 +/- 6.06. | (96) |
| **NA** | rs17700633 | Candidate | The food frequency questionnaire contained questions on the usual frequency of consumption of 77 main food items. | The study population consisted of Dutch female participants in the European Prospective Investigation into Cancer and Nutrition (EPIC), conducted in Utrecht, Netherlands (Prospect-EPIC). Between 1993 and 1997, 17,357 women aged 49–70 y and residing in or near Utrecht were recruited through a regional, population-based, breast cancer screening program. At recruitment, each participant filled in a general questionnaire on lifestyle factors, gynecologic and obstetric history, and past and current morbidity as well as a validated, semiquantitative, food-frequency questionnaire (FFQ) with the aim of capturing the habitual diet during the year preceding enrollment. A random sample of 1736 (10%) women was selected for biochemical analyses. Buffy coat samples were missing for 36 women; therefore, our study population comprised 1700 women. For the analyses of energy and macronutrient intakes, we exclude women who did not fill in the dietary questionnaire (n = 11). In addition, we excluded women with an implausibly low total energy intake of ,800 kcal/d (n = 9). Age (y) 57.22 +/- 6.06. | (96) |
| **NA** | rs6548238 | Candidate | The food frequency questionnaire contained questions on the usual frequency of consumption of 77 main food items. | The study population consisted of Dutch female participants in the European Prospective Investigation into Cancer and Nutrition (EPIC), conducted in Utrecht, Netherlands (Prospect-EPIC). Between 1993 and 1997, 17,357 women aged 49–70 y and residing in or near Utrecht were recruited through a regional, population-based, breast cancer screening program. At recruitment, each participant filled in a general questionnaire on lifestyle factors, gynecologic and obstetric history, and past and current morbidity as well as a validated, semiquantitative, food-frequency questionnaire (FFQ) with the aim of capturing the habitual diet during the year preceding enrollment. A random sample of 1736 (10%) women was selected for biochemical analyses. Buffy coat samples were missing for 36 women; therefore, our study population comprised 1700 women. For the analyses of energy and macronutrient intakes, we exclude women who did not fill in the dietary questionnaire (n = 11). In addition, we excluded women with an implausibly low total energy intake of ,800 kcal/d (n = 9). Age (y) 57.22 +/- 6.06. | (96) |
| **NA** | rs10938397 | Candidate | The food frequency questionnaire contained questions on the usual frequency of consumption of 77 main food items. | The study population consisted of Dutch female participants in the European Prospective Investigation into Cancer and Nutrition (EPIC), conducted in Utrecht, Netherlands (Prospect-EPIC). Between 1993 and 1997, 17,357 women aged 49–70 y and residing in or near Utrecht were recruited through a regional, population-based, breast cancer screening program. At recruitment, each participant filled in a general questionnaire on lifestyle factors, gynecologic and obstetric history, and past and current morbidity as well as a validated, semiquantitative, food-frequency questionnaire (FFQ) with the aim of capturing the habitual diet during the year preceding enrollment. A random sample of 1736 (10%) women was selected for biochemical analyses. Buffy coat samples were missing for 36 women; therefore, our study population comprised 1700 women. For the analyses of energy and macronutrient intakes, we exclude women who did not fill in the dietary questionnaire (n = 11). In addition, we excluded women with an implausibly low total energy intake of ,800 kcal/d (n = 9). Age (y) 57.22 +/- 6.06. | (96) |
| **NA** | rs368794 | Candidate | The food frequency questionnaire contained questions on the usual frequency of consumption of 77 main food items. | The study population consisted of Dutch female participants in the European Prospective Investigation into Cancer and Nutrition (EPIC), conducted in Utrecht, Netherlands (Prospect-EPIC). Between 1993 and 1997, 17,357 women aged 49–70 y and residing in or near Utrecht were recruited through a regional, population-based, breast cancer screening program. At recruitment, each participant filled in a general questionnaire on lifestyle factors, gynecologic and obstetric history, and past and current morbidity as well as a validated, semiquantitative, food-frequency questionnaire (FFQ) with the aim of capturing the habitual diet during the year preceding enrollment. A random sample of 1736 (10%) women was selected for biochemical analyses. Buffy coat samples were missing for 36 women; therefore, our study population comprised 1700 women. For the analyses of energy and macronutrient intakes, we exclude women who did not fill in the dietary questionnaire (n = 11). In addition, we excluded women with an implausibly low total energy intake of ,800 kcal/d (n = 9). Age (y) 57.22 +/- 6.06. | (96) |
| **NA** | rs7647305 | Candidate | The food frequency questionnaire contained questions on the usual frequency of consumption of 77 main food items. | The study population consisted of Dutch female participants in the European Prospective Investigation into Cancer and Nutrition (EPIC), conducted in Utrecht, Netherlands (Prospect-EPIC). Between 1993 and 1997, 17,357 women aged 49–70 y and residing in or near Utrecht were recruited through a regional, population-based, breast cancer screening program. At recruitment, each participant filled in a general questionnaire on lifestyle factors, gynecologic and obstetric history, and past and current morbidity as well as a validated, semiquantitative, food-frequency questionnaire (FFQ) with the aim of capturing the habitual diet during the year preceding enrollment. A random sample of 1736 (10%) women was selected for biochemical analyses. Buffy coat samples were missing for 36 women; therefore, our study population comprised 1700 women. For the analyses of energy and macronutrient intakes, we exclude women who did not fill in the dietary questionnaire (n = 11). In addition, we excluded women with an implausibly low total energy intake of ,800 kcal/d (n = 9). Age (y) 57.22 +/- 6.06. | (96) |
| **NA** | rs2844479 | Candidate | The food frequency questionnaire contained questions on the usual frequency of consumption of 77 main food items. | The study population consisted of Dutch female participants in the European Prospective Investigation into Cancer and Nutrition (EPIC), conducted in Utrecht, Netherlands (Prospect-EPIC). Between 1993 and 1997, 17,357 women aged 49–70 y and residing in or near Utrecht were recruited through a regional, population-based, breast cancer screening program. At recruitment, each participant filled in a general questionnaire on lifestyle factors, gynecologic and obstetric history, and past and current morbidity as well as a validated, semiquantitative, food-frequency questionnaire (FFQ) with the aim of capturing the habitual diet during the year preceding enrollment. A random sample of 1736 (10%) women was selected for biochemical analyses. Buffy coat samples were missing for 36 women; therefore, our study population comprised 1700 women. For the analyses of energy and macronutrient intakes, we exclude women who did not fill in the dietary questionnaire (n = 11). In addition, we excluded women with an implausibly low total energy intake of ,800 kcal/d (n = 9). Age (y) 57.22 +/- 6.06. | (96) |
| **OPRM1** | rs510769 | Candidate | Three variables from the Food Preference Questionnaire were used in the analyses. This 72-item scale was designed as a 2 (FAT: high vs low) by 3 (CARBOHYDRATE: high simple, high complex, low carbohydrate/high protein) measure of preference for various kinds of macronutrients. Respondents indicate their preference for each food on a ninepoint Likert scale. The high fat preference score comprises the mean of 36 items (for example, onion rings and barbeque chicken); the high sugar preference score the mean of 24 items (for example, canned pears and dried dates); and the high fat and high sugar preference score the mean of 12 items (for example, chocolate layer cake and pecan pie) | Three hundred women (n = 238) and men (n =62) between the ages of 24 and 50 years took part in the study. Participants were required to be fluent in English and to have lived in North America for at least 5 years before their enrolment. All female participants were also pre-menopausal as identified by the self-reporting of regular menstrual cycles. Exclusion criteria included a current diagnosis of any psychotic disorder, substance abuse, alcoholism or a serious medical/physical illness, such as cancer, heart disease or paralysis. Female participants with a pregnancy within the previous 6 months were also ineligible. The sample comprised a broad range of BMI values, with a distribution representative of the general adult population. In all, 35% had a BMI of 25 kg m2 while the remainder was classified as overweight or obese according to the World Health Organization criteria. In total, 80% of the sample was Caucasian and 13% were of African descent. (Women mean age 33.5 SD=6.6, men mean age 35.2 SD=6.7) | (84) |
| **OPRM1** | rs563649 | Candidate | Three variables from the Food Preference Questionnaire were used in the analyses. This 72-item scale was designed as a 2 (FAT: high vs low) by 3 (CARBOHYDRATE: high simple, high complex, low carbohydrate/high protein) measure of preference for various kinds of macronutrients. Respondents indicate their preference for each food on a ninepoint Likert scale. The high fat preference score comprises the mean of 36 items (for example, onion rings and barbeque chicken); the high sugar preference score the mean of 24 items (for example, canned pears and dried dates); and the high fat and high sugar preference score the mean of 12 items (for example, chocolate layer cake and pecan pie) | Three hundred women (n = 238) and men (n =62) between the ages of 24 and 50 years took part in the study. Participants were required to be fluent in English and to have lived in North America for at least 5 years before their enrolment. All female participants were also pre-menopausal as identified by the self-reporting of regular menstrual cycles. Exclusion criteria included a current diagnosis of any psychotic disorder, substance abuse, alcoholism or a serious medical/physical illness, such as cancer, heart disease or paralysis. Female participants with a pregnancy within the previous 6 months were also ineligible. The sample comprised a broad range of BMI values, with a distribution representative of the general adult population. In all, 35% had a BMI of 25 kg m2 while the remainder was classified as overweight or obese according to the World Health Organization criteria. In total, 80% of the sample was Caucasian and 13% were of African descent. (Women mean age 33.5 SD=6.6, men mean age 35.2 SD=6.7) | (84) |
| **OPRM1** | rs675026 | Candidate | Three variables from the Food Preference Questionnaire were used in the analyses. This 72-item scale was designed as a 2 (FAT: high vs low) by 3 (CARBOHYDRATE: high simple, high complex, low carbohydrate/high protein) measure of preference for various kinds of macronutrients. Respondents indicate their preference for each food on a ninepoint Likert scale. The high fat preference score comprises the mean of 36 items (for example, onion rings and barbeque chicken); the high sugar preference score the mean of 24 items (for example, canned pears and dried dates); and the high fat and high sugar preference score the mean of 12 items (for example, chocolate layer cake and pecan pie) | Three hundred women (n = 238) and men (n =62) between the ages of 24 and 50 years took part in the study. Participants were required to be fluent in English and to have lived in North America for at least 5 years before their enrolment. All female participants were also pre-menopausal as identified by the self-reporting of regular menstrual cycles. Exclusion criteria included a current diagnosis of any psychotic disorder, substance abuse, alcoholism or a serious medical/physical illness, such as cancer, heart disease or paralysis. Female participants with a pregnancy within the previous 6 months were also ineligible. The sample comprised a broad range of BMI values, with a distribution representative of the general adult population. In all, 35% had a BMI of 25 kg m2 while the remainder was classified as overweight or obese according to the World Health Organization criteria. In total, 80% of the sample was Caucasian and 13% were of African descent. (Women mean age 33.5 SD=6.6, men mean age 35.2 SD=6.7) | (84) |
| **OPRM1** | rs9322447 | Candidate | Three variables from the Food Preference Questionnaire were used in the analyses. This 72-item scale was designed as a 2 (FAT: high vs low) by 3 (CARBOHYDRATE: high simple, high complex, low carbohydrate/high protein) measure of preference for various kinds of macronutrients. Respondents indicate their preference for each food on a ninepoint Likert scale. The high fat preference score comprises the mean of 36 items (for example, onion rings and barbeque chicken); the high sugar preference score the mean of 24 items (for example, canned pears and dried dates); and the high fat and high sugar preference score the mean of 12 items (for example, chocolate layer cake and pecan pie) | Three hundred women (n = 238) and men (n =62) between the ages of 24 and 50 years took part in the study. Participants were required to be fluent in English and to have lived in North America for at least 5 years before their enrolment. All female participants were also pre-menopausal as identified by the self-reporting of regular menstrual cycles. Exclusion criteria included a current diagnosis of any psychotic disorder, substance abuse, alcoholism or a serious medical/physical illness, such as cancer, heart disease or paralysis. Female participants with a pregnancy within the previous 6 months were also ineligible. The sample comprised a broad range of BMI values, with a distribution representative of the general adult population. In all, 35% had a BMI of 25 kg m2 while the remainder was classified as overweight or obese according to the World Health Organization criteria. In total, 80% of the sample was Caucasian and 13% were of African descent. (Women mean age 33.5 SD=6.6, men mean age 35.2 SD=6.7) | (84) |
| **OPRM1** | rs558948 | Candidate | Three variables from the Food Preference Questionnaire were used in the analyses. This 72-item scale was designed as a 2 (FAT: high vs low) by 3 (CARBOHYDRATE: high simple, high complex, low carbohydrate/high protein) measure of preference for various kinds of macronutrients. Respondents indicate their preference for each food on a ninepoint Likert scale. The high fat preference score comprises the mean of 36 items (for example, onion rings and barbeque chicken); the high sugar preference score the mean of 24 items (for example, canned pears and dried dates); and the high fat and high sugar preference score the mean of 12 items (for example, chocolate layer cake and pecan pie) | Three hundred women (n = 238) and men (n =62) between the ages of 24 and 50 years took part in the study. Participants were required to be fluent in English and to have lived in North America for at least 5 years before their enrolment. All female participants were also pre-menopausal as identified by the self-reporting of regular menstrual cycles. Exclusion criteria included a current diagnosis of any psychotic disorder, substance abuse, alcoholism or a serious medical/physical illness, such as cancer, heart disease or paralysis. Female participants with a pregnancy within the previous 6 months were also ineligible. The sample comprised a broad range of BMI values, with a distribution representative of the general adult population. In all, 35% had a BMI of 25 kg m2 while the remainder was classified as overweight or obese according to the World Health Organization criteria. In total, 80% of the sample was Caucasian and 13% were of African descent. (Women mean age 33.5 SD=6.6, men mean age 35.2 SD=6.7) | (84) |
| **OXTR** | rs53576 | Candidate | Food Preference Questionnaire: This questionnaire provides hedonic ratings for 72 common food items arranged according to a 2 (FAT: high vs low) x 3 (OTHER MACRONUTRIENT: high simple sugar, high complex carbohydrate, and high protein) matrix with 12 items in each cell. In the current study, High Sugar and High Fat Foods is reflected by the 12 items from the high fat/high simple sugar cell; High Sugar Foods includes the 12 items from the High Simple Sugar/Low Fat cell and the High Fat Foods is the total (24 items) of the High Fat/High Carbohydrate and High Fat/High Protein subscales. The 72 food items of the Food Preference Questionnaire vary systematically and significantly with respect to their macronutrient content. | The sample consisted of 460 adults (females = 346) between 24 and 50 years of age. Inclusion criteria were residence in North America for at least 5 years before enrollment in the study, and fluency in written and spoken English. Women were also required to be premenopausal as indicated by the self-report of a regular menstrual cycle. Exclusion criteria included serious medical illnesses such as cancer or diabetes, or severe physical disabilities such as cerebral palsy. Those with a current axis I diagnosed disorder, with the exception of unipolar depression, were also excluded. In addition, women with a pregnancy in the previous 6 months, or who were lactating, were not included. The majority of the sample was Caucasian (79%), with 15% identifying as African descent and the remainder as ‘Other’. The sample represented a broad range of body mass index (BMI) values (17.8–75.2 kg m2). Participants comprised a community-based sample and were recruited from posters, newspaper advertisements and online sites such as Craigslist and Kijiji in a large Canadian city. Females age 33.4 (SD= 6.6); males age 34.3 (SD=7.0) | (85) |
| **OXTR** | rs2268493 | Candidate | Food Preference Questionnaire: This questionnaire provides hedonic ratings for 72 common food items arranged according to a 2 (FAT: high vs low) x 3 (OTHER MACRONUTRIENT: high simple sugar, high complex carbohydrate, and high protein) matrix with 12 items in each cell. In the current study, High Sugar and High Fat Foods is reflected by the 12 items from the high fat/high simple sugar cell; High Sugar Foods includes the 12 items from the High Simple Sugar/Low Fat cell and the High Fat Foods is the total (24 items) of the High Fat/High Carbohydrate and High Fat/High Protein subscales. The 72 food items of the Food Preference Questionnaire vary systematically and significantly with respect to their macronutrient content. | The sample consisted of 460 adults (females = 346) between 24 and 50 years of age. Inclusion criteria were residence in North America for at least 5 years before enrollment in the study, and fluency in written and spoken English. Women were also required to be premenopausal as indicated by the self-report of a regular menstrual cycle. Exclusion criteria included serious medical illnesses such as cancer or diabetes, or severe physical disabilities such as cerebral palsy. Those with a current axis I diagnosed disorder, with the exception of unipolar depression, were also excluded. In addition, women with a pregnancy in the previous 6 months, or who were lactating, were not included. The majority of the sample was Caucasian (79%), with 15% identifying as African descent and the remainder as ‘Other’. The sample represented a broad range of body mass index (BMI) values (17.8–75.2 kg m2). Participants comprised a community-based sample and were recruited from posters, newspaper advertisements and online sites such as Craigslist and Kijiji in a large Canadian city. Females age 33.4 (SD= 6.6); males age 34.3 (SD=7.0) | (85) |
| **OXTR** | rs237885 | Candidate | Food Preference Questionnaire: This questionnaire provides hedonic ratings for 72 common food items arranged according to a 2 (FAT: high vs low) x 3 (OTHER MACRONUTRIENT: high simple sugar, high complex carbohydrate, and high protein) matrix with 12 items in each cell. In the current study, High Sugar and High Fat Foods is reflected by the 12 items from the high fat/high simple sugar cell; High Sugar Foods includes the 12 items from the High Simple Sugar/Low Fat cell and the High Fat Foods is the total (24 items) of the High Fat/High Carbohydrate and High Fat/High Protein subscales. The 72 food items of the Food Preference Questionnaire vary systematically and significantly with respect to their macronutrient content. | The sample consisted of 460 adults (females = 346) between 24 and 50 years of age. Inclusion criteria were residence in North America for at least 5 years before enrollment in the study, and fluency in written and spoken English. Women were also required to be premenopausal as indicated by the self-report of a regular menstrual cycle. Exclusion criteria included serious medical illnesses such as cancer or diabetes, or severe physical disabilities such as cerebral palsy. Those with a current axis I diagnosed disorder, with the exception of unipolar depression, were also excluded. In addition, women with a pregnancy in the previous 6 months, or who were lactating, were not included. The majority of the sample was Caucasian (79%), with 15% identifying as African descent and the remainder as ‘Other’. The sample represented a broad range of body mass index (BMI) values (17.8–75.2 kg m2). Participants comprised a community-based sample and were recruited from posters, newspaper advertisements and online sites such as Craigslist and Kijiji in a large Canadian city. Females age 33.4 (SD= 6.6); males age 34.3 (SD=7.0) | (85) |
| **OXTR** | rs2254298 | Candidate | Food Preference Questionnaire: This questionnaire provides hedonic ratings for 72 common food items arranged according to a 2 (FAT: high vs low) x 3 (OTHER MACRONUTRIENT: high simple sugar, high complex carbohydrate, and high protein) matrix with 12 items in each cell. In the current study, High Sugar and High Fat Foods is reflected by the 12 items from the high fat/high simple sugar cell; High Sugar Foods includes the 12 items from the High Simple Sugar/Low Fat cell and the High Fat Foods is the total (24 items) of the High Fat/High Carbohydrate and High Fat/High Protein subscales. The 72 food items of the Food Preference Questionnaire vary systematically and significantly with respect to their macronutrient content. | The sample consisted of 460 adults (females = 346) between 24 and 50 years of age. Inclusion criteria were residence in North America for at least 5 years before enrollment in the study, and fluency in written and spoken English. Women were also required to be premenopausal as indicated by the self-report of a regular menstrual cycle. Exclusion criteria included serious medical illnesses such as cancer or diabetes, or severe physical disabilities such as cerebral palsy. Those with a current axis I diagnosed disorder, with the exception of unipolar depression, were also excluded. In addition, women with a pregnancy in the previous 6 months, or who were lactating, were not included. The majority of the sample was Caucasian (79%), with 15% identifying as African descent and the remainder as ‘Other’. The sample represented a broad range of body mass index (BMI) values (17.8–75.2 kg m2). Participants comprised a community-based sample and were recruited from posters, newspaper advertisements and online sites such as Craigslist and Kijiji in a large Canadian city. Females age 33.4 (SD= 6.6); males age 34.3 (SD=7.0) | (85) |
| **OXTR** | rs2268498 | Candidate | Food Preference Questionnaire: This questionnaire provides hedonic ratings for 72 common food items arranged according to a 2 (FAT: high vs low) x 3 (OTHER MACRONUTRIENT: high simple sugar, high complex carbohydrate, and high protein) matrix with 12 items in each cell. In the current study, High Sugar and High Fat Foods is reflected by the 12 items from the high fat/high simple sugar cell; High Sugar Foods includes the 12 items from the High Simple Sugar/Low Fat cell and the High Fat Foods is the total (24 items) of the High Fat/High Carbohydrate and High Fat/High Protein subscales. The 72 food items of the Food Preference Questionnaire vary systematically and significantly with respect to their macronutrient content. | The sample consisted of 460 adults (females = 346) between 24 and 50 years of age. Inclusion criteria were residence in North America for at least 5 years before enrollment in the study, and fluency in written and spoken English. Women were also required to be premenopausal as indicated by the self-report of a regular menstrual cycle. Exclusion criteria included serious medical illnesses such as cancer or diabetes, or severe physical disabilities such as cerebral palsy. Those with a current axis I diagnosed disorder, with the exception of unipolar depression, were also excluded. In addition, women with a pregnancy in the previous 6 months, or who were lactating, were not included. The majority of the sample was Caucasian (79%), with 15% identifying as African descent and the remainder as ‘Other’. The sample represented a broad range of body mass index (BMI) values (17.8–75.2 kg m2). Participants comprised a community-based sample and were recruited from posters, newspaper advertisements and online sites such as Craigslist and Kijiji in a large Canadian city. Females age 33.4 (SD= 6.6); males age 34.3 (SD=7.0) | (85) |
| **POMC** | rs3754860 | Candidate | Participants were advised to complete specific selfadministered demographic questionnaires along with 7-day food records. | A total of 185 unrelated Czech Caucasian individuals were recruited for this case-control study and were divided in two groups. All the participants in the study, both obese individuals and the lean controls, were recruited in a mass media campaign addressing the population of the south Moravia region in the Czech Republic. Both the cases and the controls underwent the same diagnostic procedures to avoid possible selection bias. In recruitment, the inclusion and exclusion criteria of Ma et al. were used. The case group consisted of 125 obese individuals (body mass index [BMI] ≥ 30 kg/m2; mean BMI, 37.7 ± 6.38 kg/m2: median age, 50.0 years; range, 18.6–68.9 years). In this group of obese individuals, a subset of 34 morbidly obese patients was identified using the criterion of BMI ≥ 40 kg/m2 (mean BMI, 45.5 ± 3.6 kg/m2: median age, 50.5 years; range, 18.6–67.8 years); all of these 34 patients were available for the plasma leptin and sObR determinations. The control group consisted of 60 healthy, normal-weight, control subjects with no history of childhood obesity (mean BMI, 25.8 ± 3.3 kg/m2: median age, 47.5 years; range, 18.1–67.5 years). Data on personal or family history of obesity, birth weight, age at onset of obesity, eating disorders, age of menarche and menopause in women, family history of sterility, infertility or stillbirth were obtained by a professional using a semi-structured interview. A positive family history for obesity was estimated as one obese relative (BMI ≥ 30 kg/m2 in the close family – siblings, parents and their siblings and grandparents). Both the obese cases and the controls underwent the same examinations on their anthropometric characteristics, dietary intake and genetic background of the individual and the family. | (81) |
| **POMC** | rs1009388 | Candidate | Participants were advised to complete specific selfadministered demographic questionnaires along with 7-day food records. | A total of 185 unrelated Czech Caucasian individuals were recruited for this case-control study and were divided in two groups. All the participants in the study, both obese individuals and the lean controls, were recruited in a mass media campaign addressing the population of the south Moravia region in the Czech Republic. Both the cases and the controls underwent the same diagnostic procedures to avoid possible selection bias. In recruitment, the inclusion and exclusion criteria of Ma et al. were used. The case group consisted of 125 obese individuals (body mass index [BMI] ≥ 30 kg/m2; mean BMI, 37.7 ± 6.38 kg/m2: median age, 50.0 years; range, 18.6–68.9 years). In this group of obese individuals, a subset of 34 morbidly obese patients was identified using the criterion of BMI ≥ 40 kg/m2 (mean BMI, 45.5 ± 3.6 kg/m2: median age, 50.5 years; range, 18.6–67.8 years); all of these 34 patients were available for the plasma leptin and sObR determinations. The control group consisted of 60 healthy, normal-weight, control subjects with no history of childhood obesity (mean BMI, 25.8 ± 3.3 kg/m2: median age, 47.5 years; range, 18.1–67.5 years). Data on personal or family history of obesity, birth weight, age at onset of obesity, eating disorders, age of menarche and menopause in women, family history of sterility, infertility or stillbirth were obtained by a professional using a semi-structured interview. A positive family history for obesity was estimated as one obese relative (BMI ≥ 30 kg/m2 in the close family – siblings, parents and their siblings and grandparents). Both the obese cases and the controls underwent the same examinations on their anthropometric characteristics, dietary intake and genetic background of the individual and the family. | (81) |
| **RGS6** | rs860195 |  | A food frequency questionnaire was administered by interview at the follow-up visit. This questionnaire contains a 70-item food list. | The IRASFS was designed to explore genetic and epidemiologic contributions to abdominal adiposity and glucose homeostasis traits among Hispanic and African Americans using a family-based design. Large families were recruited from 2000 to 2002 at study centers in San Antonio, TX (Hispanics); San Luis Valley, CO (Hispanics); and Los Angeles, CA (African Americans); with probands identified from both the parent study IRAS as well as the general population. Families were recruited based upon family size, not disease or phenotype status. A follow-up examination was conducted ~5 years after the baseline examination, during which dietary intake data were collected. As the RGS6 genotyping was only done in Hispanics the current study only includes the Hispanic cohort of the IRASFS. Individuals with diabetes remained in our dataset for  analysis. Total (n) 932;  Gender (% female) 61.9%;  Age (years) 47.8 ± 14.1;  BMI (kg/m2) 29.8 ± 6.2. | (95) |
| **RGS6** | rs2283394 |  | A food frequency questionnaire was administered by interview at the follow-up visit. This questionnaire contains a 70-item food list. | The IRASFS was designed to explore genetic and epidemiologic contributions to abdominal adiposity and glucose homeostasis traits among Hispanic and African Americans using a family-based design. Large families were recruited from 2000 to 2002 at study centers in San Antonio, TX (Hispanics); San Luis Valley, CO (Hispanics); and Los Angeles, CA (African Americans); with probands identified from both the parent study IRAS as well as the general population. Families were recruited based upon family size, not disease or phenotype status. A follow-up examination was conducted ~5 years after the baseline examination, during which dietary intake data were collected. As the RGS6 genotyping was only done in Hispanics the current study only includes the Hispanic cohort of the IRASFS. Individuals with diabetes remained in our dataset for  analysis. Total (n) 932;  Gender (% female) 61.9%;  Age (years) 47.8 ± 14.1;  BMI (kg/m2) 29.8 ± 6.2. | (95) |
| **RGS6** | rs847352 |  | A food frequency questionnaire was administered by interview at the follow-up visit. This questionnaire contains a 70-item food list. | The IRASFS was designed to explore genetic and epidemiologic contributions to abdominal adiposity and glucose homeostasis traits among Hispanic and African Americans using a family-based design. Large families were recruited from 2000 to 2002 at study centers in San Antonio, TX (Hispanics); San Luis Valley, CO (Hispanics); and Los Angeles, CA (African Americans); with probands identified from both the parent study IRAS as well as the general population. Families were recruited based upon family size, not disease or phenotype status. A follow-up examination was conducted ~5 years after the baseline examination, during which dietary intake data were collected. As the RGS6 genotyping was only done in Hispanics the current study only includes the Hispanic cohort of the IRASFS. Individuals with diabetes remained in our dataset for  analysis. Total (n) 932;  Gender (% female) 61.9%;  Age (years) 47.8 ± 14.1;  BMI (kg/m2) 29.8 ± 6.2. | (95) |
| **RGS6** | rs2239250 |  | A food frequency questionnaire was administered by interview at the follow-up visit. This questionnaire contains a 70-item food list. | The IRASFS was designed to explore genetic and epidemiologic contributions to abdominal adiposity and glucose homeostasis traits among Hispanic and African Americans using a family-based design. Large families were recruited from 2000 to 2002 at study centers in San Antonio, TX (Hispanics); San Luis Valley, CO (Hispanics); and Los Angeles, CA (African Americans); with probands identified from both the parent study IRAS as well as the general population. Families were recruited based upon family size, not disease or phenotype status. A follow-up examination was conducted ~5 years after the baseline examination, during which dietary intake data were collected. As the RGS6 genotyping was only done in Hispanics the current study only includes the Hispanic cohort of the IRASFS. Individuals with diabetes remained in our dataset for  analysis. Total (n) 932;  Gender (% female) 61.9%;  Age (years) 47.8 ± 14.1;  BMI (kg/m2) 29.8 ± 6.2. | (95) |
| **RGS6** | rs8018927 |  | A food frequency questionnaire was administered by interview at the follow-up visit. This questionnaire contains a 70-item food list. | The IRASFS was designed to explore genetic and epidemiologic contributions to abdominal adiposity and glucose homeostasis traits among Hispanic and African Americans using a family-based design. Large families were recruited from 2000 to 2002 at study centers in San Antonio, TX (Hispanics); San Luis Valley, CO (Hispanics); and Los Angeles, CA (African Americans); with probands identified from both the parent study IRAS as well as the general population. Families were recruited based upon family size, not disease or phenotype status. A follow-up examination was conducted ~5 years after the baseline examination, during which dietary intake data were collected. As the RGS6 genotyping was only done in Hispanics the current study only includes the Hispanic cohort of the IRASFS. Individuals with diabetes remained in our dataset for  analysis. Total (n) 932;  Gender (% female) 61.9%;  Age (years) 47.8 ± 14.1;  BMI (kg/m2) 29.8 ± 6.2. | (95) |
| **RGS6** | rs7147236 |  | A food frequency questionnaire was administered by interview at the follow-up visit. This questionnaire contains a 70-item food list. | The IRASFS was designed to explore genetic and epidemiologic contributions to abdominal adiposity and glucose homeostasis traits among Hispanic and African Americans using a family-based design. Large families were recruited from 2000 to 2002 at study centers in San Antonio, TX (Hispanics); San Luis Valley, CO (Hispanics); and Los Angeles, CA (African Americans); with probands identified from both the parent study IRAS as well as the general population. Families were recruited based upon family size, not disease or phenotype status. A follow-up examination was conducted ~5 years after the baseline examination, during which dietary intake data were collected. As the RGS6 genotyping was only done in Hispanics the current study only includes the Hispanic cohort of the IRASFS. Individuals with diabetes remained in our dataset for  analysis. Total (n) 932;  Gender (% female) 61.9%;  Age (years) 47.8 ± 14.1;  BMI (kg/m2) 29.8 ± 6.2. | (95) |
| **RGS6** | rs2238199 |  | A food frequency questionnaire was administered by interview at the follow-up visit. This questionnaire contains a 70-item food list. | The IRASFS was designed to explore genetic and epidemiologic contributions to abdominal adiposity and glucose homeostasis traits among Hispanic and African Americans using a family-based design. Large families were recruited from 2000 to 2002 at study centers in San Antonio, TX (Hispanics); San Luis Valley, CO (Hispanics); and Los Angeles, CA (African Americans); with probands identified from both the parent study IRAS as well as the general population. Families were recruited based upon family size, not disease or phenotype status. A follow-up examination was conducted ~5 years after the baseline examination, during which dietary intake data were collected. As the RGS6 genotyping was only done in Hispanics the current study only includes the Hispanic cohort of the IRASFS. Individuals with diabetes remained in our dataset for  analysis. Total (n) 932;  Gender (% female) 61.9%;  Age (years) 47.8 ± 14.1;  BMI (kg/m2) 29.8 ± 6.2. | (95) |
| **RGS6** | rs2681749 |  | A food frequency questionnaire was administered by interview at the follow-up visit. This questionnaire contains a 70-item food list. | The IRASFS was designed to explore genetic and epidemiologic contributions to abdominal adiposity and glucose homeostasis traits among Hispanic and African Americans using a family-based design. Large families were recruited from 2000 to 2002 at study centers in San Antonio, TX (Hispanics); San Luis Valley, CO (Hispanics); and Los Angeles, CA (African Americans); with probands identified from both the parent study IRAS as well as the general population. Families were recruited based upon family size, not disease or phenotype status. A follow-up examination was conducted ~5 years after the baseline examination, during which dietary intake data were collected. As the RGS6 genotyping was only done in Hispanics the current study only includes the Hispanic cohort of the IRASFS. Individuals with diabetes remained in our dataset for  analysis. Total (n) 932;  Gender (% female) 61.9%;  Age (years) 47.8 ± 14.1;  BMI (kg/m2) 29.8 ± 6.2. | (95) |
| **RGS6** | rs12892244 |  | A food frequency questionnaire was administered by interview at the follow-up visit. This questionnaire contains a 70-item food list. | The IRASFS was designed to explore genetic and epidemiologic contributions to abdominal adiposity and glucose homeostasis traits among Hispanic and African Americans using a family-based design. Large families were recruited from 2000 to 2002 at study centers in San Antonio, TX (Hispanics); San Luis Valley, CO (Hispanics); and Los Angeles, CA (African Americans); with probands identified from both the parent study IRAS as well as the general population. Families were recruited based upon family size, not disease or phenotype status. A follow-up examination was conducted ~5 years after the baseline examination, during which dietary intake data were collected. As the RGS6 genotyping was only done in Hispanics the current study only includes the Hispanic cohort of the IRASFS. Individuals with diabetes remained in our dataset for  analysis. Total (n) 932;  Gender (% female) 61.9%;  Age (years) 47.8 ± 14.1;  BMI (kg/m2) 29.8 ± 6.2. | (95) |
| **RGS6** | rs847334 |  | A food frequency questionnaire was administered by interview at the follow-up visit. This questionnaire contains a 70-item food list. | The IRASFS was designed to explore genetic and epidemiologic contributions to abdominal adiposity and glucose homeostasis traits among Hispanic and African Americans using a family-based design. Large families were recruited from 2000 to 2002 at study centers in San Antonio, TX (Hispanics); San Luis Valley, CO (Hispanics); and Los Angeles, CA (African Americans); with probands identified from both the parent study IRAS as well as the general population. Families were recruited based upon family size, not disease or phenotype status. A follow-up examination was conducted ~5 years after the baseline examination, during which dietary intake data were collected. As the RGS6 genotyping was only done in Hispanics the current study only includes the Hispanic cohort of the IRASFS. Individuals with diabetes remained in our dataset for  analysis. Total (n) 932;  Gender (% female) 61.9%;  Age (years) 47.8 ± 14.1;  BMI (kg/m2) 29.8 ± 6.2. | (95) |
| **RGS6** | rs2239227 |  | A food frequency questionnaire was administered by interview at the follow-up visit. This questionnaire contains a 70-item food list. | The IRASFS was designed to explore genetic and epidemiologic contributions to abdominal adiposity and glucose homeostasis traits among Hispanic and African Americans using a family-based design. Large families were recruited from 2000 to 2002 at study centers in San Antonio, TX (Hispanics); San Luis Valley, CO (Hispanics); and Los Angeles, CA (African Americans); with probands identified from both the parent study IRAS as well as the general population. Families were recruited based upon family size, not disease or phenotype status. A follow-up examination was conducted ~5 years after the baseline examination, during which dietary intake data were collected. As the RGS6 genotyping was only done in Hispanics the current study only includes the Hispanic cohort of the IRASFS. Individuals with diabetes remained in our dataset for  analysis. Total (n) 932;  Gender (% female) 61.9%;  Age (years) 47.8 ± 14.1;  BMI (kg/m2) 29.8 ± 6.2. | (95) |
| **RGS6** | rs4903013 |  | A food frequency questionnaire was administered by interview at the follow-up visit. This questionnaire contains a 70-item food list. | The IRASFS was designed to explore genetic and epidemiologic contributions to abdominal adiposity and glucose homeostasis traits among Hispanic and African Americans using a family-based design. Large families were recruited from 2000 to 2002 at study centers in San Antonio, TX (Hispanics); San Luis Valley, CO (Hispanics); and Los Angeles, CA (African Americans); with probands identified from both the parent study IRAS as well as the general population. Families were recruited based upon family size, not disease or phenotype status. A follow-up examination was conducted ~5 years after the baseline examination, during which dietary intake data were collected. As the RGS6 genotyping was only done in Hispanics the current study only includes the Hispanic cohort of the IRASFS. Individuals with diabetes remained in our dataset for  analysis. Total (n) 932;  Gender (% female) 61.9%;  Age (years) 47.8 ± 14.1;  BMI (kg/m2) 29.8 ± 6.2. | (95) |
| **RGS6** | rs10149207 |  | A food frequency questionnaire was administered by interview at the follow-up visit. This questionnaire contains a 70-item food list. | The IRASFS was designed to explore genetic and epidemiologic contributions to abdominal adiposity and glucose homeostasis traits among Hispanic and African Americans using a family-based design. Large families were recruited from 2000 to 2002 at study centers in San Antonio, TX (Hispanics); San Luis Valley, CO (Hispanics); and Los Angeles, CA (African Americans); with probands identified from both the parent study IRAS as well as the general population. Families were recruited based upon family size, not disease or phenotype status. A follow-up examination was conducted ~5 years after the baseline examination, during which dietary intake data were collected. As the RGS6 genotyping was only done in Hispanics the current study only includes the Hispanic cohort of the IRASFS. Individuals with diabetes remained in our dataset for  analysis. Total (n) 932;  Gender (% female) 61.9%;  Age (years) 47.8 ± 14.1;  BMI (kg/m2) 29.8 ± 6.2. | (95) |
| **RGS6** | rs6574069 |  | A food frequency questionnaire was administered by interview at the follow-up visit. This questionnaire contains a 70-item food list. | The IRASFS was designed to explore genetic and epidemiologic contributions to abdominal adiposity and glucose homeostasis traits among Hispanic and African Americans using a family-based design. Large families were recruited from 2000 to 2002 at study centers in San Antonio, TX (Hispanics); San Luis Valley, CO (Hispanics); and Los Angeles, CA (African Americans); with probands identified from both the parent study IRAS as well as the general population. Families were recruited based upon family size, not disease or phenotype status. A follow-up examination was conducted ~5 years after the baseline examination, during which dietary intake data were collected. As the RGS6 genotyping was only done in Hispanics the current study only includes the Hispanic cohort of the IRASFS. Individuals with diabetes remained in our dataset for  analysis. Total (n) 932;  Gender (% female) 61.9%;  Age (years) 47.8 ± 14.1;  BMI (kg/m2) 29.8 ± 6.2. | (95) |
| **RGS6** | rs2239223 |  | A food frequency questionnaire was administered by interview at the follow-up visit. This questionnaire contains a 70-item food list. | The IRASFS was designed to explore genetic and epidemiologic contributions to abdominal adiposity and glucose homeostasis traits among Hispanic and African Americans using a family-based design. Large families were recruited from 2000 to 2002 at study centers in San Antonio, TX (Hispanics); San Luis Valley, CO (Hispanics); and Los Angeles, CA (African Americans); with probands identified from both the parent study IRAS as well as the general population. Families were recruited based upon family size, not disease or phenotype status. A follow-up examination was conducted ~5 years after the baseline examination, during which dietary intake data were collected. As the RGS6 genotyping was only done in Hispanics the current study only includes the Hispanic cohort of the IRASFS. Individuals with diabetes remained in our dataset for  analysis. Total (n) 932;  Gender (% female) 61.9%;  Age (years) 47.8 ± 14.1;  BMI (kg/m2) 29.8 ± 6.2. | (95) |
| **RGS6** | rs2239219 |  | A food frequency questionnaire was administered by interview at the follow-up visit. This questionnaire contains a 70-item food list. | The IRASFS was designed to explore genetic and epidemiologic contributions to abdominal adiposity and glucose homeostasis traits among Hispanic and African Americans using a family-based design. Large families were recruited from 2000 to 2002 at study centers in San Antonio, TX (Hispanics); San Luis Valley, CO (Hispanics); and Los Angeles, CA (African Americans); with probands identified from both the parent study IRAS as well as the general population. Families were recruited based upon family size, not disease or phenotype status. A follow-up examination was conducted ~5 years after the baseline examination, during which dietary intake data were collected. As the RGS6 genotyping was only done in Hispanics the current study only includes the Hispanic cohort of the IRASFS. Individuals with diabetes remained in our dataset for  analysis. Total (n) 932;  Gender (% female) 61.9%;  Age (years) 47.8 ± 14.1;  BMI (kg/m2) 29.8 ± 6.2. | (95) |
| **RGS6** | rs10149848 |  | A food frequency questionnaire was administered by interview at the follow-up visit. This questionnaire contains a 70-item food list. | The IRASFS was designed to explore genetic and epidemiologic contributions to abdominal adiposity and glucose homeostasis traits among Hispanic and African Americans using a family-based design. Large families were recruited from 2000 to 2002 at study centers in San Antonio, TX (Hispanics); San Luis Valley, CO (Hispanics); and Los Angeles, CA (African Americans); with probands identified from both the parent study IRAS as well as the general population. Families were recruited based upon family size, not disease or phenotype status. A follow-up examination was conducted ~5 years after the baseline examination, during which dietary intake data were collected. As the RGS6 genotyping was only done in Hispanics the current study only includes the Hispanic cohort of the IRASFS. Individuals with diabetes remained in our dataset for  analysis. Total (n) 932;  Gender (% female) 61.9%;  Age (years) 47.8 ± 14.1;  BMI (kg/m2) 29.8 ± 6.2. | (95) |
| **TAS2R38** | rs713598 | Candidate | Test-meal procedures. Items served at the test-meal were selected because they were highly palatable and familiar to most children this age. Prior to the experiment, the foods were divided into three categories: sweets (e.g. red licorice, gummies, and sweetened beverages), sweet-fats (e.g. cookies, brownies, doughnuts), and savory-fats (e.g. pizza, mozzarella sticks, chips). Foods in the savory-fat or sweet-fat category had either savory or sweet as their predominant flavor characteristic and contained 20% calories from fat or greater. Items in the sweet food category were primarily sweet tasting and contained less than 1 g of fat per serving. | Children (n = 79) enrolled in this study were between 4 and 6 years old (mean ± SD = 5.04 ± 0.78). Parents self-reported the ethnicity of their children as African-American (42.5%), Hispanic/Latino (31.3%), Caucasian (12.5%), Asian (2.5%) or “other” (11.3%). Approximately 40% of the children were boys. Average BMI z-score for children was 1.00 ± 1.02, corresponding to the 85th BMI-forage percentile. TAS2R38 genotype was unable to be determined for three children due to failures of the test kits or inadequate saliva samples for a failure rate of 4%. Genotype at rs713598 is reported for 76 children. | (9) |
| **TAS2R38** | A49P (rs713598), V296I (rs10246939) | Candidate | Dietary assessment: A subset (registered for nutrition education classes, 47) completed food records of each meal, including weights fot 3 consecutive weeks in June | 87 female college students, age 18-22, recruited from university community, born 1987-91. | (59) |
| **TRPV1** | rs161364 | Candidate | Preferences oiliness were asked about as ‘Do you like food with (sweet/salty/etc.) taste?’ The preference for food taste was given as ‘very dislike’ (1), ‘dislike’ (2), ‘moderate’ (3), ‘like’ (4), or ‘very like’ (5). Preferences were categorized into two groups as ‘low preference’, including ‘very dislike’ and ‘dislike’, and as ‘high preference’, including the rest of the scale for each taste. Nutrient Intake The usual food intake was determined by a semi-quantitative food frequency questionnaire (SQFFQ).] | A total of 4,183 men and 4,659 women aged 40–69 years (8,842 participants) were recruited from two community-based epidemiological cohorts, the rural community of Anseong city and the urban community of Ansan city. Subjects from both cohorts were sources of the genetic information that was collected in 2001 as part of the KoGES, and the part containing genetic information was called the Korean Association Resource (KARE) study. All participants had lived within the survey area for at least 6 months and were mentally and physically healthy | (64) |
| **TRPV1** | rs8065080 | Candidate | Preferences oiliness were asked about as ‘Do you like food with (sweet/salty/etc.) taste?’ The preference for food taste was given as ‘very dislike’ (1), ‘dislike’ (2), ‘moderate’ (3), ‘like’ (4), or ‘very like’ (5). Preferences were categorized into two groups as ‘low preference’, including ‘very dislike’ and ‘dislike’, and as ‘high preference’, including the rest of the scale for each taste. Nutrient Intake The usual food intake was determined by a semi-quantitative food frequency questionnaire (SQFFQ).] | A total of 4,183 men and 4,659 women aged 40–69 years (8,842 participants) were recruited from two community-based epidemiological cohorts, the rural community of Anseong city and the urban community of Ansan city. Subjects from both cohorts were sources of the genetic information that was collected in 2001 as part of the KoGES, and the part containing genetic information was called the Korean Association Resource (KARE) study. All participants had lived within the survey area for at least 6 months and were mentally and physically healthy | (64) |

**Supplementary Table 9 Study characteristics of genetic association studies related to umami taste preferences**

IMP: inosine monophosphate, MPG: monopotassium glutamate, MSG: monosodium glutamate, M+I: MSG in the presence of IMP.

**Supplementary Table 9 Study characteristics of genetic association studies related to umami taste preferences**

| **Gene** | **SNP** | **Discovery method** | **Phenotype assessment method** | | **Study characteristics** | **Findings** | **Reference** | **Study characteristics (no association)** | **Reference (no association)** |
| --- | --- | --- | --- | --- | --- | --- | --- | --- | --- |
| **TAS1R3** | rs307377 | Candidate | | Recognition thresholds were measured for five qualities of taste using different concentrations of MSG (0.2,400 mM), inosine 59- monophosphate (IMP, 0.02,40 mM), MSG in the presence of 0.5 mM IMP (M+I, 0.003,12.5 mM), sucrose (0.2,400 mM), NaCl (0.2,400 mM), HCl (0.02,40 mM) and PTC (0.001,2.0 mM). The testing procedure was the staircase-method. The procedure was terminated after five reversals, and the threshold was calculated as the mean of the concentration values of the last four reversals. | A total of 254 healthy, non-obese, non-diabetic Japanese volunteers [male/female: 141/113, age: 20–36, body mass index (BMI); 16.5–24.8] participated in the study. Inclusion criteria were satisfactory state of oral hygiene, nonsmoking, regular work, sleep, and meal schedules. | Significant associations between allele frequency and recognition threshold for IMP. | (61) | - | - |
|  |  | Candidate | | The following methods were applied: Test measuring the stimulus concentration perceived as intense as the 29 mmol NaCl/L reference.  Forced-choice paired comparisons to identify the stronger concentration. Questionnaire based paired comparisons. The hedonic value was assessed for each tube on a 210 to 10 scale.  Ranking test. of subjects according to perceived intensity. | The study group consisted of 142 white, French, genetically unrelated subjects (55 men and 87 women). | The mutation was less frequent in tasters than expected. | (100) |  |  |
|  |  | Candidate | | The general labeled magnitude scale (gLMS) was used it to rate the intensity of the sensation and the taste quality of taste stimuli (MPG/L). | Human genomic DNA was obtained from a population of 87 US subjects, mostly of Dutch ancestry. The youngest subjects in this population were fourth-generation Dutch-American immigrants and the oldest were second generation. All of the subjects were healthy individuals (44% men) recruited from Michigan with a mean age (+/-SD) of 35 +/- 19 y (age range: 14–89 y). | CT subjects rated MPG/L twice as did those with CC genotype. | (63) | - | - |
| **TAS1R3** | rs76755863 | Candidate | | The following methods were applied: Test measuring the stimulus concentration perceived as intense as the 29 mmol NaCl/L reference.  Forced-choice paired comparisons to identify the stronger concentration. Questionnaire based paired comparisons. The hedonic value was assessed for each tube on a 210 to 10 scale.  Ranking test. of subjects according to perceived intensity. | The study group consisted of 142 white, French, genetically unrelated subjects (55 men and 87 women). | The mutation G13A was associated with non-tasters and hypotasters. | (100) | - | - |
|  |  | Candidate | | The general labeled magnitude scale (gLMS) was used it to rate the intensity of the sensation and the taste quality of taste stimuli (MPG/L). | Human genomic DNA was obtained from a population of 87 US subjects, mostly of Dutch ancestry. The youngest subjects in this population were fourth-generation Dutch-American immigrants and the oldest were second generation. All of the subjects were healthy individuals (44% men) recruited from Michigan with a mean age (+/-SD) of 35 +/- 19 y (age range: 14–89 y). | For the rare allele doubling of umami taste intensity ratings. | (63) | - | - |
| **TAS1R3** | rs111615792 | Candidate | | The general labeled magnitude scale (gLMS) was used it to rate the intensity of the sensation and the taste quality of taste stimuli (MPG/L). | Human genomic DNA was obtained from a population of 87 US subjects, mostly of Dutch ancestry. The youngest subjects in this population were fourth-generation Dutch-American immigrants and the oldest were second generation. All of the subjects were healthy individuals (44% men) recruited from Michigan with a mean age (+/-SD) of 35 +/- 19 y (age range: 14–89 y). | For the rare allele doubling of umami taste intensity ratings. | (63) | - | - |
| **TAS1R1** | rs34160967 | Candidate | | Recognition thresholds were measured for five qualities of taste using different concentrations of MSG (0.2,400 mM), inosine 59- monophosphate (IMP, 0.02,40 mM), MSG in the presence of 0.5 mM IMP (M+I, 0.003,12.5 mM), sucrose (0.2,400 mM), NaCl (0.2,400 mM), HCl (0.02,40 mM) and PTC (0.001,2.0 mM). The testing procedure was the staircase-method. The procedure was terminated after five reversals, and the threshold was calculated as the mean of the concentration values of the last four reversals. | A total of 254 healthy, non-obese, non-diabetic Japanese volunteers [male/female: 141/113, age: 20–36, body mass index (BMI); 16.5–24.8] participated in the study. Inclusion criteria were satisfactory state of oral hygiene, nonsmoking, regular work, sleep, and meal schedules | Significant associations between genotypes and recognition thresholds for MSG and M+I. | (61) | Human genomic DNA was obtained from a population of 87 US subjects, mostly of Dutch ancestry. The youngest subjects in this population were fourth-generation Dutch-American immigrants and the oldest were second generation. All of the subjects were healthy individuals (44% men) recruited from Michigan with a mean age (+/-SD) of 35 +/- 19 y (age range: 14–89 y). | (63) |
|  |  | Candidate | | The following methods were applied: Test measuring the stimulus concentration perceived as intense as the 29 mmol NaCl/L reference.  Forced-choice paired comparisons to identify the stronger concentration. Questionnaire based paired comparisons. The hedonic value was assessed for each tube on a 210 to 10 scale.  Ranking test. of subjects according to perceived intensity. | The study group consisted of 142 white, French, genetically unrelated subjects (55 men and 87 women). | The SNP was more frequent in tasters than expected. | (100) | An overall number of 183 individuals (belonging to four different geographical regions and highly diversiﬁed in terms of both genetic background and food habits: Italy, North Europe, Maghreb and Sri Lanka.) were recruited and enrolled in the study. Subjects (81 females and 102 males with an average age of 42.71 ± 15.89) did not report any food allergies, were not following any prescribed diet or using drugs that might interfere with taste perception. Most of the participants (N=111) were Italians, with the remaining subjects coming from the Maghreb region (N =18), Sri Lanka (N =26) and Northern Europe (N=28) but recruited in Italy. | (11) |
| **TAS1R1** | rs41278020 | Candidate | | The following methods were applied: Test measuring the stimulus concentration perceived as intense as the 29 mmol NaCl/L reference.  Forced-choice paired comparisons to identify the stronger concentration. Questionnaire based paired comparisons. The hedonic value was assessed for each tube on a 210 to 10 scale.  Ranking test. of subjects according to perceived intensity. | The study group consisted of 142 white, French, genetically unrelated subjects (55 men and 87 women). | The mutation was more frequent in non-tasters than expected. | (100) | Human genomic DNA was obtained from a population of 87 US subjects, mostly of Dutch ancestry. The youngest subjects in this population were fourth-generation Dutch-American immigrants and the oldest were second generation. All of the subjects were healthy individuals (44% men) recruited from Michigan with a mean age (+/-SD) of 35 +/- 19 y (age range: 14–89 y). | (63) |
| **TAS1R1** | rs35118458 | Candidate | | The following methods were applied: Test measuring the stimulus concentration perceived as intense as the 29 mmol NaCl/L reference.  Forced-choice paired comparisons to identify the stronger concentration. Questionnaire based paired comparisons. The hedonic value was assessed for each tube on a 210 to 10 scale.  Ranking test. of subjects according to perceived intensity. | The study group consisted of 142 white, French, genetically unrelated subjects (55 men and 87 women). | The mutation tended to be more frequent in non-tasters. | (100) | - | - |
| **GRM1** | rs2814863 | Candidate | | The following methods were applied: Test measuring the stimulus concentration perceived as intense as the 29 mmol NaCl/L reference.  Forced-choice paired comparisons to identify the stronger concentration. Questionnaire based paired comparisons. The hedonic value was assessed for each tube on a 210 to 10 scale.  Ranking test of subjects according to perceived intensity. | The study group consisted of 142 white, French, genetically unrelated subjects (55 men and 87 women). | The mutation tended to be associated with the non-taster phenotype. | (100) | - | - |

**Supplementary Table 10 Study characteristics of genetic association studies related to umami taste preferences (no associations)**

| **Gene** | **SNP** | **Discovery method** | **Phenotype assessment method** | **Study population characteristics** | **Reference (no association)** |
| --- | --- | --- | --- | --- | --- |
| **TAS1R3** | rs3813210 | Candidate | Recognition thresholds were measured for five qualities of taste using different concentrations of MSG (0.2,400 mM), inosine 59- monophosphate (IMP, 0.02,40 mM), MSG in the presence of 0.5 mM IMP (M+I, 0.003,12.5 mM), sucrose (0.2,400 mM), NaCl (0.2,400 mM), HCl (0.02,40 mM) and PTC (0.001,2.0 mM). The testing procedure was the staircase-method. The procedure was terminated after five reversals, and the threshold was calculated as the mean of the concentration values of the last four reversals. | A total of 254 healthy, non-obese, non-diabetic Japanese volunteers [male/female: 141/113, age: 20–36, body mass index (BMI); 16.5–24.8] participated in the study. Inclusion criteria were satisfactory state of oral hygiene, nonsmoking, regular work, sleep, and meal schedules | (61) |
|  |  | Candidate | The general labeled magnitude scale (gLMS) was used it to rate the intensity of the sensation and the taste quality of taste stimuli (MPG/L). | Human genomic DNA was obtained from a population of 87 US subjects, mostly of Dutch ancestry. The youngest subjects in this population were fourth-generation Dutch-American immigrants and the oldest were second generation. All of the subjects were healthy individuals (44% men) recruited from Michigan with a mean age (+/-SD) of 35 +/- 19 y (age range: 14–89 y). | (63) |
| **TAS1R3** | rs35424002 | Candidate | Rating the perceived intensity of umami solutions on multiple Labeled Magnitude Scales. | An overall number of 183 individuals (belonging to four different geographical regions and highly diversiﬁed in terms of both genetic background and food habits: Italy, North Europe, Maghreb and Sri Lanka.) were recruited and enrolled in the study. Subjects (81 females and 102 males with an average age of 42.71 ± 15.89) did not report any food allergies, were not following any prescribed diet or using drugs that might interfere with taste perception. Most of the participants (N=111) were Italians, with the remaining subjects coming from the Maghreb region (N =18), Sri Lanka (N =26) and Northern Europe (N=28) but recruited in Italy. | (11) |
| **GNAT3** | rs6467192 | Candidate | Rating the perceived intensity of umami solutions on multiple Labeled Magnitude Scales. | An overall number of 183 individuals (belonging to four different geographical regions and highly diversiﬁed in terms of both genetic background and food habits: Italy, North Europe, Maghreb and Sri Lanka.) were recruited and enrolled in the study. Subjects (81 females and 102 males with an average age of 42.71 ± 15.89) did not report any food allergies, were not following any prescribed diet or using drugs that might interfere with taste perception. Most of the participants (N=111) were Italians, with the remaining subjects coming from the Maghreb region (N =18), Sri Lanka (N =26) and Northern Europe (N=28) but recruited in Italy. | (11) |

**Supplementary Table 11 Study characteristics of genetic association studies related to salt taste preferences**

NaCl: sodium chloride, KCl: potassium chloride, iAUC: incremental area under the curve

**Supplementary Table 11 Study characteristics of genetic association studies related to salty taste preferences**

| **Gene** | **SNP** | **Discovery method** | **Phenotype assessment method** | **Study population characteristics** | **Findings** | **Reference (association)** | **Study characteristics (no association)** | **Reference (no association)** |
| --- | --- | --- | --- | --- | --- | --- | --- | --- |
| **TRPV1** | rs8065080 | Candidate | Suprathreshold taste sensitivity to NaCl was assessed using general labeled magnitude scales. Five solutions 0.5 log cycles apart in concentration, ranging from 0.01 to 1.0mol/L NaCl.. Individual ratings of the intensity of suprathreshold solutions were plotted and the incremental area under the curve (iAUC) for each taste sensitivity was computed. | Subjects were participants from the Toronto Nutrigenomics and Health Study. Young men and women. Men (n = 28) and women (n = 67) between the ages of 21 and 31 years were enrolled. All subjects were Caucasian and individuals who were smokers, pregnant or breast marked weight changes in the last year (greater than 15 pounds), were diagnosed with chronic sinusitis or chronic obstructive bowel disease, lost their sense of smell, often experienced severe dry mouth, were diagnosed with diabetes or any other chronic disease, or were diagnosed with a psychological disorder were excluded from the study | CC genotype significantly lower iAUCs. | (101) | - | - |
| **SCNN1B** | rs239345 | Candidate | Suprathreshold taste sensitivity to NaCl was assessed using general labeled magnitude scales. Five solutions 0.5 log cycles apart in concentration, ranging from 0.01 to 1.0mol/L NaCl.. Individual ratings of the intensity of suprathreshold solutions were plotted and the incremental area under the curve (iAUC) for each taste sensitivity was computed | Subjects were participants from the Toronto Nutrigenomics and Health Study. Young men and women. Men (n = 28) and women (n = 67) between the ages of 21 and 31 years were enrolled. All subjects were Caucasian and individuals who were smokers, pregnant or breast marked weight changes in the last year (greater than 15 pounds), were diagnosed with chronic sinusitis or chronic obstructive bowel disease, lost their sense of smell, often experienced severe dry mouth, were diagnosed with diabetes or any other chronic disease, or were diagnosed with a psychological disorder were excluded from the study | AA  genotype significantly lower iAUCs. | (101) | - | - |
| **SCNN1B** | rs3785368 | Candidate | Suprathreshold taste sensitivity to NaCl was assessed using general labeled magnitude scales. Five solutions 0.5 log cycles apart in concentration, ranging from 0.01 to 1.0mol/L NaCl.. Individual ratings of the intensity of suprathreshold solutions were plotted and the incremental area under the curve (iAUC) for each taste sensitivity was computed. | Subjects were participants from the Toronto Nutrigenomics and Health Study. Young men and women. Men (n = 28) and women (n = 67) between the ages of 21 and 31 years were enrolled. All subjects were Caucasian and individuals who were smokers, pregnant or breast marked weight changes in the last year (greater than 15 pounds), were diagnosed with chronic sinusitis or chronic obstructive bowel disease, lost their sense of smell, often experienced severe dry mouth, were diagnosed with diabetes or any other chronic disease, or were diagnosed with a psychological disorder were excluded from the study | TT genotype significantly lower iAUCs. | (101) | - | - |
| **CA6** | rs3737665 | Candidate | Participants were asked to rate the intensity, salty, sensations on generalized Labeled Magnitude Scales. | 243 healthy adults (146 women), aged 18–45 were recruited from the Pennsylvania State University campus and surrounding area. | The SNP associated with differences in the perceived intensity of NaCl and KCl saltiness. | (102) | - | - |
| **CA6** | rs3765964 | Candidate | Participants were asked to rate the intensity, salty, sensations on generalized Labeled Magnitude Scales. | 243 healthy adults (146 women), aged 18–45 were recruited from the Pennsylvania State University campus and surrounding area. | The SNP associated with differences in the perceived intensity of NaCl. | (102) | - | - |
| **CA6** | rs2274333 | Candidate | Participants were asked to rate the intensity, salty, sensations on generalized Labeled Magnitude Scales. | 243 healthy adults (146 women), aged 18–45 were recruited from the Pennsylvania State University campus and surrounding area. | The SNP associated with differences in KCl saltiness. | (102) | - | - |
| **TAS1R1** | rs17492553 | Candidate | Adults used a general labeled magnitude scale (gLMS) to rate the taste intensity of oral stimuli.of 1M NaCl. | A convenience sample of reportedly healthy, nonsmoking adults was recruited from the University of Connecticut community to participate in an observational study of variation in oral sensation, diet, and health. Exclusion criteria included pregnancy, severe food allergies, and thyroid disease. The study sample included 92 adults, primarily of European ancestry (84.8%), female (76%), and middle aged (mean 40.9±12.2 SD). Other ethnicities represented in the sample were Black (5.4%), Hispanic or Latino (5.4%), Asian (3.3%), and other (1.1%). | T (allele, genotype) lower intensities. | (53) | - | - |
| **TAS1R1** | rs34160967 | Candidate | Adults used a general labeled magnitude scale (gLMS) to rate the taste intensity of oral stimuli.of 1M NaCl. | A convenience sample of reportedly healthy, nonsmoking adults was recruited from the University of Connecticut community to participate in an observational study of variation in oral sensation, diet, and health. Exclusion criteria included pregnancy, severe food allergies, and thyroid disease. The study sample included 92 adults, primarily of European ancestry (84.8%), female (76%), and middle aged (mean 40.9±12.2 SD). Other ethnicities represented in the sample were Black (5.4%), Hispanic or Latino (5.4%), Asian (3.3%), and other (1.1%). | A (allele, genotype) lower intensities. | (53) | - | - |
| **TAS2R38** | A49P (rs713598), A262V (rs1726866), V296I (rs10246939) |  | The stimuli was 0.1 mmol/l of NaCl solution, which was kept in the mouth for 10 s, and then rate the perceived intensity. | The study population consisted of individuals (n = 393; 212 males,181 females, mean age 35.9 ± 12.0 years) at least 19 years of ager, ecruited as a convenience sample from Institute of ChemicalTechnology, Mumbai, India. Individuals, who were smokers, visited the dentist 3 days prior to the test, sinus problems, using medications that might aﬀect sensory (taste/smell) perception, having food allergies or cold and women (pregnant or lactating) were excluded. All individuals were in good health at the time of sampling. For TAS2R38 polymorphisms: the sample consisted of Indian population [n = 393; 212 (53.9%)men and 181 (46.1%) women with a mean age of 27.3 ± 6.7 years]. | PAV/PAV higher ratings for saltiness intensity. | (27) | A sample of 198 reportedly healthy nonsmokers were recruited from the areas surrounding the University of Connecticut campus. Twenty individuals with rare genotypes were excluded from analysis unless stated otherwise, leaving Self- identified ethnic group: Asian Black Caucasian Hispanic Native American Other: 18, 14, 334, 22, 4, 2. Mean age was 38 years (±13 standard deviation), ranging from 21 to 60. | (20) |

**Supplementary Table 12 Study characteristics of genetic association studies related to sour taste preferences**

**Supplementary Table 12 Study characteristics of genetic association studies related to sour taste preferences**

| **Gene** | **SNP** | **Discovery method** | **Phenotype assessment method** | **Study population characteristics** | **Findings** | **Reference (association)** | **Reference (no association)** |
| --- | --- | --- | --- | --- | --- | --- | --- |
| **TAS1R1** | rs17492553 | Candidate | Adults used a general labeled magnitude scale (gLMS) to rate the taste intensity of oral stimulus of 3.2mM citric acid. | A convenience sample of reportedly healthy, nonsmoking adults was recruited from the University of Connecticut community to participate in an observational study of variation in oral sensation, diet, and health. Exclusion criteria included pregnancy, severe food allergies, and thyroid disease. The study sample included 92 adults, primarily of European ancestry (84.8%), female (76%), and middle aged (mean 40.9±12.2 SD). Other ethnicities represented in the sample were Black (5.4%), Hispanic or Latino (5.4%), Asian (3.3%), and other (1.1%). | T (allele, genotype) associated with lower intensities. | (53) | - |
| **TAS1R1** | rs34160967 | Candidate | Adults used a general labeled magnitude scale (gLMS) to rate the taste intensity of oral stimulus of 3.2mM citric acid. | A convenience sample of reportedly healthy, nonsmoking adults was recruited from the University of Connecticut community to participate in an observational study of variation in oral sensation, diet, and health. Exclusion criteria included pregnancy, severe food allergies, and thyroid disease. The study sample included 92 adults, primarily of European ancestry (84.8%), female (76%), and middle aged (mean 40.9±12.2 SD). Other ethnicities represented in the sample were Black (5.4%), Hispanic or Latino (5.4%), Asian (3.3%), and other (1.1%). | A (allele, genotype) associated with lower intensities. | (53) | - |
| **TAS2R38** | A49P (rs713598), A262V (rs1726866), V296I (rs10246939) | Candidate | Subjects used the general Labeled Magnitude Scale (gLMS) to report the intensity of the samples (3.2 mM citric acid). | A sample of 198 reportedly healthy, nonsmokers, who were recruited from the areas surrounding the University of Connecticut campus. Twenty individuals with rare genotypes were excluded from analysis unless stated otherwise, leaving Self-identified ethnic group: Asian Black Caucasian Hispanic Native American Other: 18, 14, 334, 22, 4, 2. Mean age was 38 years (±13 standard deviation), ranging from 21 to 60. | - | - | (20) |
| **TAS2R38** | A49P (rs713598), A262V (rs1726866), V296I (rs10246939) | Candidate | The liking of odor, appearance, and flavor (sourness of berry juice samples and extracts) was measured using a nine-point balanced hedonic scale (from 1 = dislike extremely to 9 = like extremely). | A total of 41 Finnish voluntary subjects (32 females and 9 males) between ages 20 and 60 (low 20–30, n = 13; medium 31–40, n = 16; high 41–60, n = 12) were recruited. They were prescreened for their hTAS2R38 genotype and selected according to their willingness, availability, motivation, and previously demonstrated capability to work as part of a sensory panel. Subjects were University staff and also local citizens, who had previously participated in our studies. | AVI/AVI rated sourness higher than the PAV/PAV subjects. | (36) | - |
| **NA** | rs6466849 | Candidate | Participants were asked to indicate the degree of wine sourness using a visual analogic scale (VAS) from 0 (not detectable) to 10 (strong taste impression). | A sample of 599 individuals of legal drinking age composed by students, university staff and blood donors, was recruited in two different European countries: 299 at the Institute of Experimental Medicine, Czech Academy of Sciences, Prague, Czech Republic and 300 at the Biology Department, Pisa University, Italy. Volunteers were enrolled to obtain two groups with similar mean age (40±15 years), and an equal male: female ratio. The final number of participants, who were eligible for association studies between  genotype and phenotype (fully characterized by the questionnaire, stimuli perception and genotyping), included 272 Czechs (average age 43.4±11.7; male 58%, female 42%) and 235 Italians (average age 37.7±15.2; male 47%, female 53%). The association analyses were carried out using 27 SNPs belonging to 20 genes in a total number of 507 individuals. | Variant allele associated with wine sourness. | (103) | - |

**References**

1. Colares-Bento FC, Souza VC, Toledo JO, Moraes CF, Alho CS, Lima RM, et al. Implication of the G145C polymorphism (rs713598) of the TAS2r38 gene on food consumption by Brazilian older women. Arch Gerontol Geriatr (2012) 54(2):e13-8. Epub 2011/07/19. doi: 10.1016/j.archger.2011.05.019.

2. Lucock M, Ng X, Boyd L, Skinner V, Wai R, Tang S, et al. TAS2R38 bitter taste genetics, dietary vitamin C, and both natural and synthetic dietary folic acid predict folate status, a key micronutrient in the pathoaetiology of adenomatous polyps. Food Funct (2011) 2(8):457-65.

3. Bering AB, Pickering G, Liang P. TAS2R38 single nucleotide polymorphisms are associated with PROP-but not thermal-tasting: a pilot study. Chem Percept (2014) 7:23.

4. Wooding S, Gunn H, Ramos P, Thalmann S, Xing C, Meyerhof W. Genetics and bitter taste responses to goitrin, a plant toxin found in vegetables. Chem Senses (2010) 35(8):685-92. Epub 2010/06/17. doi: 10.1093/chemse/bjq061. PubMed PMID: 20551074.

5. Carrai M, Campa D, Vodicka P, Flamini R, Martelli I, Slyskova J, et al. Association between taste receptor (TAS) genes and the perception of wine characteristics. Sci Rep (2017) 7(1):9239.

6. Kim U, Jorgenson E, Coon H, Leppert M, Risch N, Drayna D.. Positional cloning of the human quantitative trait locus underlying taste sensitivity to phenylthiocarbamide. Science (New York, NY) (2003) 299(5610):1221-5. Epub 2003/02/22. doi: 10.1126/science.1080190.

7. Allen AL, McGeary JE, Hayes JE. Polymorphisms in TRPV1 and TAS2Rs Associate with Sensations from Sampled Ethanol. Alcohol Clin Exp Res (2014) 38(10):2550-60. doi: 10.1111/acer.12527.

8. Allen AL, McGeary JE, Knopik VS, Hayes JE. Bitterness of the non-nutritive Sweetener Acesulfame Potassium varies with polymorphisms in TAS2R9 and TAS2R31. Chem Senses (2013) 38(5):379-89. doi: 10.1093/chemse/bjt017.

9. Keller KL, Olsen A, Cravener TL, Bloom R, Chung WK, Deng L. Bitter taste phenotype and body weight predict children's selection of sweet and savory foods at a palatable test-meal. Appetite (2014) 77:113-21. Epub 2014/03/13. doi: 10.1016/j.appet.2014.02.019.

10. Mennella JA, Reed DR, Roberts KM, Mathew PS, Mansfield CJ. Age-related differences in bitter taste and efficacy of bitter blockers. PloS One (2014) 9(7):e103107. doi: 10.1371/journal.pone.0103107.

11. Risso DS, Giuliani C, Antinucci M, Morini G, Garagnani P, Tofanelli S, et al. A bio-cultural approach to the study of food choice: The contribution of taste genetics, population and culture. Appetite (2017) 114:240-7. Epub 2017/04/04. doi: 10.1016/j.appet.2017.03.046.

12. Ooi SX, Lee PL, Law HY, Say YH. Bitter receptor gene (TAS2R38) P49A genotypes and their associations with aversion to vegetables and sweet/fat foods in Malaysian subjects. Asia Pac J Clin Nutr (2010) 19(4):491-8. Epub 2010/12/15. PubMed PMID: 21147709.

13. Behrens M, Gunn HC, Ramos PC, Meyerhof W, Wooding SP. Genetic, functional, and phenotypic diversity in TAS2R38-mediated bitter taste perception. Chem Senses (2013) 38(6):475-84.

14. Mennella JA, Pepino MY, Duke FF, Reed DR. Psychophysical dissection of genotype effects on human bitter perception. Chem Senses (2011) 36(2):161-7. Epub 2010/10/29. doi: 10.1093/chemse/bjq106.

15. Robino A, Mezzavilla M, Pirastu N, La Bianca M, Gasparini P, Carlino D, et al. Understanding the role of personality and alexithymia in food preferences and PROP taste perception. Physiol Behav (2016) 157:72-8. Epub 2016/01/26. doi: 10.1016/j.physbeh.2016.01.022.

16. Ledda M, Kutalik Z, Souza Destito MC, Souza MM, Cirillo CA, Zamboni A, et al. GWAS of human bitter taste perception identifies new loci and reveals additional complexity of bitter taste genetics. Hum Mol Genet (2014) 23(1):259-67. doi: 10.1093/hmg/ddt404.

17. Timpson NJ, Heron J, Day IN, Ring SM, Bartoshuk LM, Horwood J, et al. Refining associations between TAS2R38 diplotypes and the 6-n-propylthiouracil (PROP) taste test: findings from the Avon Longitudinal Study of Parents and Children. BMC Genet (2007) 8:51.

18. Sacerdote C, Guarrera S, Smith G, Grioni S, Krogh V, Masala G, et al. Lactase persistence and bitter taste response: instrumental variables and mendelian randomization in epidemiologic studies of dietary factors and cancer risk. Am J Epidemiol (2007) 166(5):576-81. Epub 2007/06/29. doi: 10.1093/aje/kwm113.

19. Cabras T, Melis M, Castagnola M, Padiglia A, Tepper BJ, Messana I, et al. Responsiveness to 6-n-propylthiouracil (PROP) is associated with salivary levels of two specific basic proline-rich proteins in humans. PloS One (2012) 7(2). doi: 10.1371/journal.pone.0030962.

20. Hayes JE, Bartoshuk LM, Kidd JR, Duffy VB. Supertasting and PROP bitterness depends on more than the TAS2R38 gene. Chem Senses (2008) 33(3):255-65. Epub 2008/01/23. doi: 10.1093/chemse/bjm084.

21. Duffy VB, Davidson AC, Kidd JR, Kidd KK, Speed WC, Pakstis AJ, et al. Bitter receptor gene (TAS2R38), 6-n-propylthiouracil (PROP) bitterness and alcohol intake. Alcohol Clin Exp Res (2004) 28(11):1629-37. doi: 10.1097/01.ALC.0000145789.55183.D4.

22. Feeney EL, O’Brien SA, Scannell AG, Markey A, Gibney ER. Genetic and environmental influences on liking and reported intakes of vegetables in Irish children. ‎Food Qual Prefer (2014) 32:253-63. doi: 10.1016/j.foodqual.2013.09.009.

23. Calò C, Padiglia A, Zonza A, Corrias L, Contu P, Tepper BJ, et al. Polymorphisms in TAS2R38 and the taste bud trophic factor, gustin gene co-operate in modulating PROP taste phenotype. Physiol Behav (2011) 5(104):1065-71.

24. Negri R, Di Feola M, Di Domenico S, Scala MG, Artesi G, Valente S, et al. Taste perception and food choices. J Pediatr Gastroenterol Nutr (2012) 54(5):624-9. Epub 2011/12/27. doi: 10.1097/MPG.0b013e3182473308.

25. Melis M, Atzori E, Cabras S, Zonza A, Calo C, Muroni P, et al. The gustin (CA6) gene polymorphism, rs2274333 (A/G), as a mechanistic link between PROP tasting and fungiform taste papilla density and maintenance. PloS One (2013) 8(9):e74151. Epub 2013/09/17. doi: 10.1371/journal.pone.0074151.

26. Campbell MC, Ranciaro A, Froment A, Hirbo J, Omar S, Bodo JM, et al. Evolution of functionally diverse alleles associated with PTC bitter taste sensitivity in Africa. Mol Biol Evol (2012) 29(4):1141-53. Epub 2011/12/02. doi: 10.1093/molbev/msr293.

27. Deshaware S, Singhal R. Genetic variation in bitter taste receptor gene TAS2R38, PROP taster status and their association with body mass index and food preferences in Indian population. Gene (2017) 627:363-8. Epub 2017/06/28. doi: 10.1016/j.gene.2017.06.047. PubMed PMID: 28652185.

28. Mennella JA, Pepino MY, Reed DR. Genetic and environmental determinants of bitter perception and sweet preferences. Pediatrics (2005) 115(2):e216. doi: 10.1542/peds.2004-1582.

29. Melis M, Sollai G, Muroni P, Crnjar R, Barbarossa IT. Associations between orosensory perception of oleic acid, the common single nucleotide polymorphisms (rs1761667 and rs1527483) in the CD36 gene, and 6-n-propylthiouracil (PROP) tasting. Nutrients (2015) 7(3):2068-84. Epub 2015/03/25. doi: 10.3390/nu7032068.

30. Duffy VB, Hayes JE, Davidson AC, Kidd JR, Kidd KK, Bartoshuk LM. Vegetable intake in college-aged adults is explained by oral sensory phenotypes and TAS2R38 genotype. Chemosens Percept (2010) 3(3-4):137-48.

31. Garneau NL, Nuessle TM, Sloan MM, Santorico SA, Coughlin BC, Hayes JE. Crowdsourcing taste research: genetic and phenotypic predictors of bitter taste perception as a model. Front Integr Neurosci (2014) 8(33). doi: 10.3389/fnint.2014.00033.

32. Robino A, Mezzavilla M, Pirastu N, Dognini M, Tepper BJ, Gasparini P. A population-based approach to study the impact of PROP perception on food liking in populations along the Silk Road. PloS One (2014) 9(3):e91716. doi: 10.1371/journal.pone.0091716.

33. Sandell MA, Breslin PA. Variability in a taste-receptor gene determines whether we taste toxins in food. Curr Biol (2006) 16(18):R792-4.

34. Nolden AA MJ,  Hayes JE. Differential bitterness in capsaicin, piperine, and ethanol associates with polymorphisms in multiple bitter taste receptor genes. Physiol Behav (2016) 156:117-27.

35. Bella L, Methven L, Wagstaff C. The influence of phytochemical composition and resulting sensory attributes on preference for salad rocket (Eruca sativa) accessions by consumers of varying TAS2R38 diplotype. Food chemistry (2017) 222:6-17.

36. Laaksonen O, Ahola J, Sandell M. Explaining and predicting individually experienced liking of berry fractions by the hTAS2R38 taste receptor genotype. Appetite (2013) 61(1):85-96. Epub 2012/11/22. doi: 10.1016/j.appet.2012.10.023.

37. Feeney E, O'Brien S, Scannell A, Markey A, Gibney ER. Genetic variation in taste perception: does it have a role in healthy eating? Proc Nutr Soc (2011) 70(1):135-43. Epub 2010/11/26. doi: 10.1017/s0029665110003976.

38. Knaapila A, Hwang LD, Lysenko A, Duke FF, Fesi B, Khoshnevisan A, et al. Genetic analysis of chemosensory traits in human twins. Chem Senses (2012) 37(9):869-81.

39. Hayes JE, Feeney EL, Nolden AA, McGeary JE. Quinine bitterness and grapefruit liking associate with allelic variants in TAS2R31. Chem Senses (2015) 40(6):437-43. doi: 10.1093/chemse/bjv027.

40. Reed DR, Zhu G, Breslin PA, Duke FF, Henders AK, Campbell MJ, et al. The perception of quinine taste intensity is associated with common genetic variants in a bitter receptor cluster on chromosome 12. Hum Mol Genet (2010) 19(21):4278-85.

41. Roudnitzky N, Behrens M, Engel A, Kohl S, Thalmann S, Hübner S, et al. Receptor polymorphism and genomic structure interact to shape bitter taste perception. PLoS Genet (2015) 11(9):e1005530.

42. Hayes JE, Wallace MR, Knopik VS, Herbstman DM, Bartoshuk LM, Duffy VB, et al. Allelic variation in TAS2R bitter receptor genes associates with variation in sensations from and ingestive behaviors toward common bitter beverages in adults. Chem Senses (2011) 36(3):311-9. Epub 2010/12/18. doi: 10.1093/chemse/bjq132.

43. Roudnitzky N, Bufe B, Thalmann S, Kuhn C, Gunn HC, Xing C, et al. Genomic, genetic and functional dissection of bitter taste responses to artificial sweeteners. Hum Mol Genet (2011) 20(17):3437-49. doi: 10.1093/hmg/ddr252.

44. Pronin AN, Xu H, Tang H, Zhang L, Li Q, Li X. Specific alleles of bitter receptor genes influence human sensitivity to the bitterness of aloin and saccharin. Curr Biol (2007) 17(16):1403-8. doi: 10.1016/j.cub.2007.07.046.

45. Allen AL MJ, Hayes JE. Rebaudioside A and Rebaudioside D bitterness do not covary with Acesulfame K bitterness or polymorphisms in TAS2R9 and TAS2R31. Chemosens Percept (2013) 6:3.

46. Padiglia A, Zonza A, Atzori E, Chillotti C, Calo C, Tepper BJ, et al. Sensitivity to 6-n-propylthiouracil is associated with gustin (carbonic anhydrase VI) gene polymorphism, salivary zinc, and body mass index in humans. Am J Clin Nutr (2010) 92(3):539-45. Epub 2010/07/16. doi: 10.3945/ajcn.2010.29418.

47. Pirastu N, Kooyman M, Traglia M, Robino A, Willems SM, Pistis G, et al. A genome-wide association study in isolated populations reveals new genes associated to common food likings. Reviews in Endocrine and Metabolic Disorders (2016) 17(2):209-19. doi: 10.1007/s11154-016-9354-3.

48. Reed DR, Knaapila A. Genetics of taste and smell: poisons and pleasures. Prog Mol Biol Transl Sci (2010) 94:213–40.

49. Robino A. Genetic variation in taste perception and its role in food liking and health status. Triest: Universita’ degli Studi di Trieste (2014).

50. Tomassini Barbarossa I, Ozdener MH, Melis M, Love-Gregory L, Mitreva M, Abumrad NA, et al. Variant in a common odorant-binding protein gene is associated with bitter sensitivity in people. Behavioural brain research (2017) 329:200-4. Epub 2017/05/11. doi: 10.1016/j.bbr.2017.05.015.

51. Pirastu N , Kooyman M, Robino A, van der Spek A, Navarini L, Amin N, et al. Non-additive genome-wide association scan reveals a new gene associated with habitual coffee consumption. Sci Rep (2016) 6(31590).

52. Ledda M, Kutalik Z, Souza Destito MC, Souza MM, Cirillo CA, Zamboni A, et al. GWAS of human bitter taste perception identifies new loci and reveals additional complexity of bitter taste genetics. Hum Mol Genet (2014) 23(1):259-67. Epub 2013/08/24. doi: 10.1093/hmg/ddt404.

53. Rawal S, Hayes JE, Wallace MR, Bartoshuk LM, Duffy VB. Do polymorphisms in the TAS1R1 gene contribute to broader differences in human taste intensity? Chem Senses (2013) 38(8):719-28.

54. Ramos-Lopez O, Panduro A, Martinez-Lopez E, Roman S. Sweet taste receptor TAS1R2 polymorphism (Val191Val) is associated with a higher carbohydrate intake and hypertriglyceridemia among the population of West Mexico. Nutrients (2016) 8(2):101.

55. Campbell MC, Ranciaro A, Zinshteyn D, Rawlings-Goss R, Hirbo J, Thompson S, et al. Origin and differential selection of allelic variation at TAS2R16 associated with salicin bitter taste sensitivity in Africa. Molecular biology and evolution (2014) 31(2):288-302. Epub 2013/11/02. doi: 10.1093/molbev/mst211.

56. Pirastu N, Kooyman M, Traglia M, Robino A, Willems SM, Pistis G, et al. Association analysis of bitter receptor genes in five isolated populations identifies a significant correlation between TAS2R43 variants and coffee liking. PLoS One (2014) 9(3):e92065.

57. Roudnitzky N, Bufe B, Thalmann S, Kuhn C, Gunn HC, Xing C, et al. Genomic, genetic and functional dissection of bitter taste responses to artificial sweeteners. Hum Mol Genet (2011) 20(17):3437-49.

58. Caruso MG, Gazzerro P, Notarnicola M, Cisternino AM, Guerra V, Misciagna G, et al. Cannabinoid type 1 receptor gene polymorphism and macronutrient intake. Journal of nutrigenetics and nutrigenomics (2012) 5(6):305-13. Epub 2012/12/05. doi: 10.1159/000343563. PubMed PMID: 23207972.

59. Inoue H, Yamakawa-Kobayashi K, Suzuki Y, Nakano T, Hayashi H, Kuwano T. A case study on the association of variation of bitter-taste receptor gene TAS2R38 with the height, weight and energy intake in Japanese female college students. J Nutr Sci Vitaminol (Tokyo) (2013) 59(1):16-21.

60. Joseph PV, Reed DR, Mennella JA. Individual differences among children in sucrose detection thresholds: relationship with age, gender, and bitter taste genotype. Nursing Research (2016) 65(1):3-12. Epub 2015/12/04. doi: 10.1097/nnr.0000000000000138.

61. Shigemura N, Shirosaki S, Sanematsu K, Yoshida R, Ninomiya Y. Genetic and molecular basis of individual differences in human umami taste perception. PloS One (2009) 4(8):e6717. Epub 2009/08/22. doi: 10.1371/journal.pone.0006717.

62. Robino A. Genetic variation in taste perception and its role in food liking and health status. Trieste, Italy: Università degli studi di Trieste (2014).

63. Chen QY, Alarcon S, Tharp A, Ahmed OM, Estrella NL, Greene TA, et al. Perceptual variation in umami taste and polymorphisms in TAS1R taste receptor genes. Am J Clin Nutr (2009) 90(3):770S-9S. Epub 2009/07/10. doi: 10.3945/ajcn.2009.27462N.

64. Park S, Zhang X, Lee NR, Jin HS. TRPV1 Gene Polymorphisms Are Associated with Type 2 Diabetes by Their Interaction with Fat Consumption in the Korean Genome Epidemiology Study. J Nutrigenet Nutrigenomics (2016) 9(1):47-61. Epub 2016/06/12. doi: 10.1159/000446499.

65. Dias AG, Eny KM, Cockburn M, Chiu W, Nielsen DE, Duizer L, et al. Variation in the TAS1R2 gene, sweet taste perception and intake of sugars. J Nutrigenet Nutrigenomics (2015) 8(2):81-90. Epub 2015/08/19. doi: 10.1159/000430886. PubMed PMID: 26279452.

66. Fushan AA, Simons CT, Slack JP, Manichaikul A, Drayna D. Allelic polymorphism within the TAS1R3 promoter is associated with human taste sensitivity to sucrose. Curr Biol (2009) 19(15):1288-93. Epub 2009/06/30. doi: 10.1016/j.cub.2009.06.015.

67. Han P, Keast RSJ, Roura E. Salivary leptin and TAS1R2/TAS1R3 polymorphisms are related to sweet taste sensitivity and carbohydrate intake from a buffet meal in healthy young adults. The British journal of nutrition (2017) 118(10):763-70. Epub 2017/11/08. doi: 10.1017/s0007114517002872.

68. Eny KM, Wolever TM, Corey PN, El-Sohemy A. Genetic variation in TAS1R2 (Ile191Val) is associated with consumption of sugars in overweight and obese individuals in 2 distinct populations. Am J Clin Nutr (2010) 92(6):1501-10.

69. Mennella JA, Finkbeiner S, Lipchock SV, Hwang LD, Reed DR. Preferences for salty and sweet tastes are elevated and related to each other during childhood. PloS one (2014) 9(3):e92201. Epub 2014/03/19. doi: 10.1371/journal.pone.0092201.

70. Pawellek I, Grote V, Rzehak P, Xhonneux A, Verduci E, et al. Association of TAS2R38 variants with sweet food intake in children aged 1-6 years. Appetite (2016) 107:126-34. Epub 2016/08/01. doi: 10.1016/j.appet.2016.07.034.

71. Lipchock SV, Reed DR, Mennella JA. Relationship between bitter-taste receptor genotype and solid medication formulation usage among young children: a retrospective analysis. Clinical therapeutics (2012) 34(3):728-33. Epub 2012/03/24. doi: 10.1016/j.clinthera.2012.02.006.

72. Perna S, Riva A, Nicosanti G, Carrai M, Barale R, et al. Association of the bitter taste receptor gene TAS2R38 (polymorphism RS713598) with sensory responsiveness, food preferences, biochemical parameters and body-composition markers. A cross-sectional study in Italy. Int J of Food Sci Nut (2018) 69(2):245-52. doi: 10.1080/09637486.2017.1353954.

73. Suomela JP, Vaarno J, Sandell M, Lehtonen HM, Tahvonen R, Viikari J, et al. Children's hedonic response to berry products: Effect of chemical composition of berries and hTAS2R38 genotype on liking. Food Chemistry (2012) 135(3):1210-9. Epub 2012/09/08. doi: 10.1016/j.foodchem.2012.05.079.

74. Sandell M, Hoppu U, Mikkilä V, Mononen N, Kähönen M, Männistö S, et al. Genetic variation in the hTAS2R38 taste receptor and food consumption among Finnish adults. Genes Nutr (2014) 9(6):433. doi: doi: 10.1007/s12263-014-0433-3.

75. Wakai K, Matsuo K, Matsuda F, Yamada R, Takahashi M, Kawaguchi T, et al. Genome-wide association study of genetic factors related to confectionery intake: Potential roles of the ADIPOQ gene. Obesity (2013) 21(11):2413-9. doi: 10.1002/oby.20316.

76. Jablonski M, Jasiewicz A, Kucharska-Mazur J, Samochowiec J, Bienkowski P, Mierzejewski P, et al. The effect of selected polymorphisms of the dopamine receptor gene DRD2 and the ANKK-1 on the preference of concentrations of sucrose solutions in men with alcohol dependence. Psychiatr Danub (2013) 25(4):371-8. Epub 2013/11/20.

77. Pérusse L, Tremblay A, Leblanc C, Cloninger CR, Reich T, Rice J, et al. Familial resemblance in energy intake: contribution of genetic and environmental factors. Am J Clin Nutr (1988) 47(4):629-35.

78. Eny KM CP, El-Sohemy A. Dopamine D2 receptor genotype (C957T) and habitual consumption of sugars in a free-living population of men and women. J Nutrigenet Nutrigenomics (2009) 2(4-5):235-42.

79. Søberg S, Sandholt CH, Jespersen NZ, Toft U, Madsen AL, von Holstein-Rathlou S, et al. FGF21 is a sugar-induced hormone associated with sweet intake and preference in humans. (2017) 25(5):1045-53.e6. doi: 10.1016/j.cmet.2017.04.009

80. Mizuta E, Kokubo Y, Yamanaka I, Miyamoto Y, Okayama A, Yoshimasa Y, et al. Leptin gene and leptin receptor gene polymorphisms are associated with sweet preference and obesity. Hypertens Res (2008) 31(6):1069-77. Epub 2008/08/22. doi: 10.1291/hypres.31.1069.

81. Bienertova-Vasku J, Bienert P, Tomandl J, Forejt M, Vavrina M, Kudelkova J, et al. No association of defined variability in leptin, leptin receptor, adiponectin, proopiomelanocortin and ghrelin gene with food preferences in the Czech population. Nutritional neuroscience (2008) 11(1):2-8. Epub 2008/05/31. doi: 10.1179/147683008x301379.

82. Fushan AA, Simons CT, Slack JP, Drayna D. Association between common variation in genes encoding sweet taste signaling components and human sucrose perception. Chem Senses (2010) 35(7):579-92.

83. Elbers CC, de Kovel CGF, van der Schouw YT, Meijboom JR, Bauer F, Grobbee DE, et al. Variants in neuropeptide Y receptor 1 and 5 are associated with nutrient-specific food intake and are under recent selection in Europeans. (2009) 4(9). doi: 10.1371/journal.pone.0007070

84. Davis C, Zai C, Levitan RD, Kaplan AS, Carter JC, Reid-Westoby C, et al. Opiates, overeating and obesity: a psychogenetic analysis. Int J Obes (Lond). (2011) 35(10):1347-54. Epub 2011/01/27. doi: 10.1038/ijo.2010.276.

85. Davis C, Patte K, Zai C, Kennedy JL. Polymorphisms of the oxytocin receptor gene and overeating: the intermediary role of endophenotypic risk factors. Nutrition & diabetes (2017) 7(5):e279. Epub 2017/05/23. doi: 10.1038/nutd.2017.24.

86. Eny KM, Wolever TM, Fontaine-Bisson B, El-Sohemy A. Genetic variant in the glucose transporter type 2 is associated with higher intakes of sugars in two distinct populations. Physiol Genomics (2008) 33(3):355-60.

87. Daoudi H, Plesnik J, Sayed A, Sery O, Rouabah A, Rouabah L, et al. Oral fat sensing and CD36 gene polymorphism in Algerian lean and obese teenagers. Nutrients (2015) 7(11):9096-104. Epub 2015/11/12. doi: 10.3390/nu7115455.

88. Mrizak I, Sery O, Plesnik J, Arfa A, Fekih M, Bouslema A, et al. The A allele of cluster of differentiation 36 (CD36) SNP 1761667 associates with decreased lipid taste perception in obese Tunisian women. Br J Nutr. (2015) 113(8):1330-7. Epub 2015/03/31. doi: 10.1017/s0007114515000343.

89. Sayed A, Šerý O, Plesnik J, Daoudi H, Rouabah A, Rouabah L, et al. CD36 AA genotype is associated with decreased lipid taste perception in young obese, but not lean, children. Int J Obes (London) (2015) 39(6):920-4. doi: 10.1038/ijo.2015.20.

90. Pepino MY, Love-Gregory L, Klein S, Abumrad NA. The fatty acid translocase gene CD36 and lingual lipase influence oral sensitivity to fat in obese subjects. Journal of lipid research (2012) 53(3):561-6. Epub 2012/01/03. doi: 10.1194/jlr.M021873.

91. Keller K, Liang L, Sakimura J, May D, van Belle C, et al. Common variants in the CD36 gene are associated with oral fat perception, fat preferences, and obesity in African Americans. Obesity (2012) 20(5):1066-73. Epub 2012/01/14. doi: 10.1038/oby.2011.374. PubMed PMID: 22240721

92. Ong HH, Tan YN, Say YH. Fatty acid translocase gene CD36 rs1527483 variant influences oral fat perception in Malaysian subjects. Physiol Behav (2017) 168:128-37.

93. Sasaki M, Yamada K, Namba H, Yoshinaga M, Du D, Uehara Y. Angiotensinogen gene polymorphisms and food-intake behavior in young, normal female subjects in Japan. Nutrition (2013) 29(1):60-5. Epub 2012/08/04. doi: 10.1016/j.nut.2012.03.013.

94. Corella D, Amett DK, Tsai MY, Kabagambe EK, Peacock JM, Hixson JE, et al. The 256TC polymorphism in the apolipoprotein A-II gene promoter is associated with body mass index and food intake in the Genetics of Lipid Lowering Drugs and Diet Network Study. Clin Chem (2007) 53(6):1144 –52.

95. Sibbel SP, Talbert ME, Bowden DW, Haffner SM, Taylor KD, Chen YD, et al. RGS6 variants are associated with dietary fat intake in Hispanics: the IRAS Family Study. Obesity (Silver Spring) (2011) 19(7):1433-8. Epub 2011/01/15. doi: 10.1038/oby.2010.333.

96. Bauer F, Elbers CC, Adan RA, Loos RJ, Onland-Moret NC, Grobbee DE, et al. Obesity genes identified in genome-wide association studies are associated with adiposity measures and potentially with nutrient-specific food preference. Am J Clin Nutr (2009) 90(4):951-9. Epub 2009/08/21. doi: 10.3945/ajcn.2009.27781.

97. Bienertova-Vasku J, Bienert P, Tomandl J, Forejt M, Vasku A. Relation between adiponectin 45 T/G polymorphism and dietary composition in the Czech population. Diabetes research and clinical practice (2009) 84(3):329-31. Epub 2009/03/31. doi: 10.1016/j.diabres.2009.02.023.

98. Jayewardene AF, Mavros Y, Hancock DP, Gwinn T, Rooney KB. Associations between CD36 gene polymorphisms, fat tolerance and oral fat preference in a young-adult population. Eur J Clin Nutr (2016) 70(11):1325-31. Epub 2016/11/03. doi: 10.1038/ejcn.2016.132.

99. Keller KL, Liang LC, Sakimura J, May D, van Belle C, Breen C et al. Common variants in the CD36 gene are associated with oral fat perception, fat preferences, and obesity in African Americans. Obesity (Silver Spring) (2012) 20(5):1066-73. Epub 2012/01/14. doi: 10.1038/oby.2011.374.

100. Raliou M, Wiencis A, Pillias AM, Planchais A, Eloit C, Boucher Y, et al. Nonsynonymous single nucleotide polymorphisms in human tas1r1, tas1r3, and mGluR1 and individual taste sensitivity to glutamate. Am J Clin Nutr (2009) 90(3):789S-99S. Epub 2009/07/03. doi: 10.3945/ajcn.2009.27462P.

101. Dias AG, Rousseau D, Duizer L, Cockburn M, Chiu W, Nielsen D, et al. Genetic variation in putative salt taste receptors and salt taste perception in humans. Chem Senses (2013) 38(2):137-45. Epub 2012/11/03. doi: 10.1093/chemse/bjs090.

102. Feeney EL, Hayes JE. Exploring associations between taste perception, oral anatomy and polymorphisms in the carbonic anhydrase (gustin) gene CA6. Physiol Behav (2014) 128:148-54.

103. Carrai M, Campa D, Vodicka P, Flamini R, Martelli I, Slyskova J, et al. Association between taste receptor (TAS) genes and the perception of wine characteristics. Sci Rep (2017) 7(1):9239.
